# Supplementary material for: Enantioselective [3+3] atroposelective annulation catalyzed by N-heterocyclic carbenes
Source: Nat Commun. 2018 Feb 9;9:611. doi: 10.1038/s41467-018-02952-3 (PMC5807440; doi:10.1038/s41467-018-02952-3)
Supplement: Supplementary file 1 — Supplementary Information [file 41467_2018_2952_MOESM1_ESM.pdf]

Enantioselective [3+3] Atroposelective Annulation Catalyzed by N-heterocyclic Carbenes

Zhao et al.

Supplementary Figures

Supplementary Figure 1. ^{1}H NMR Spectrum of 3aa

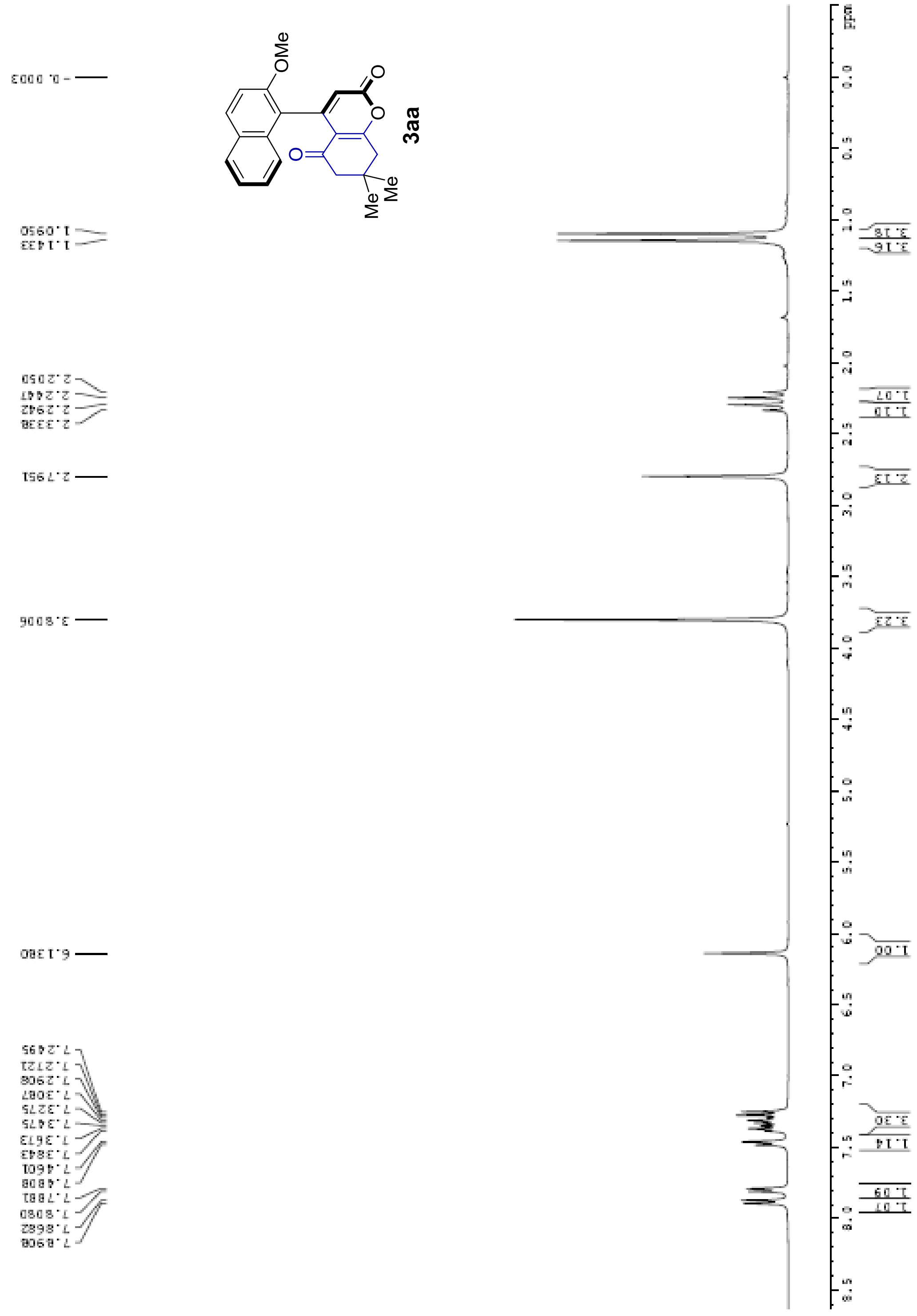

Supplementary Figure 2. ^{13}C NMR Spectrum of 3aa

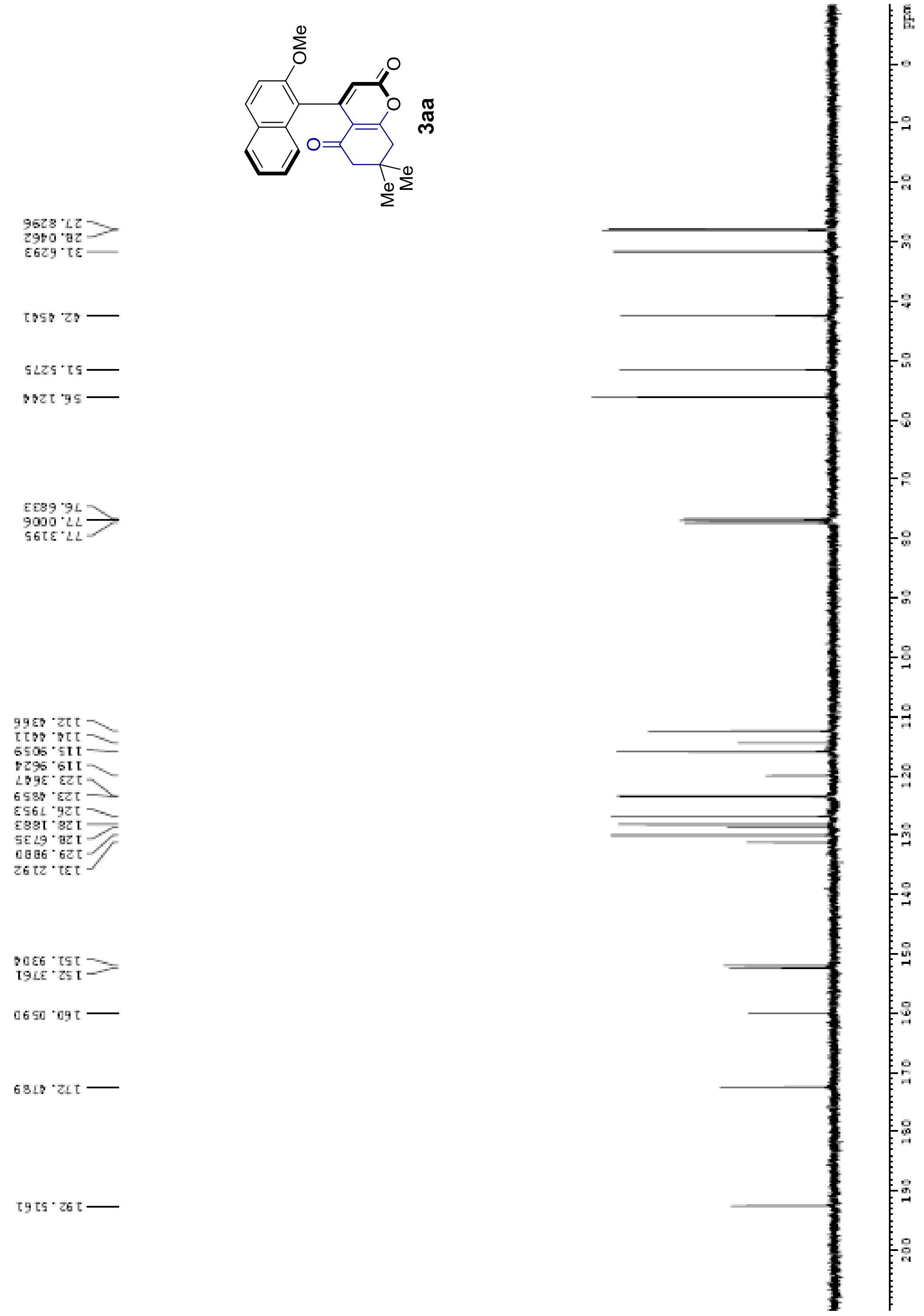

Supplementary Figure 3. ^{13}C NMR Spectrum of 3aa

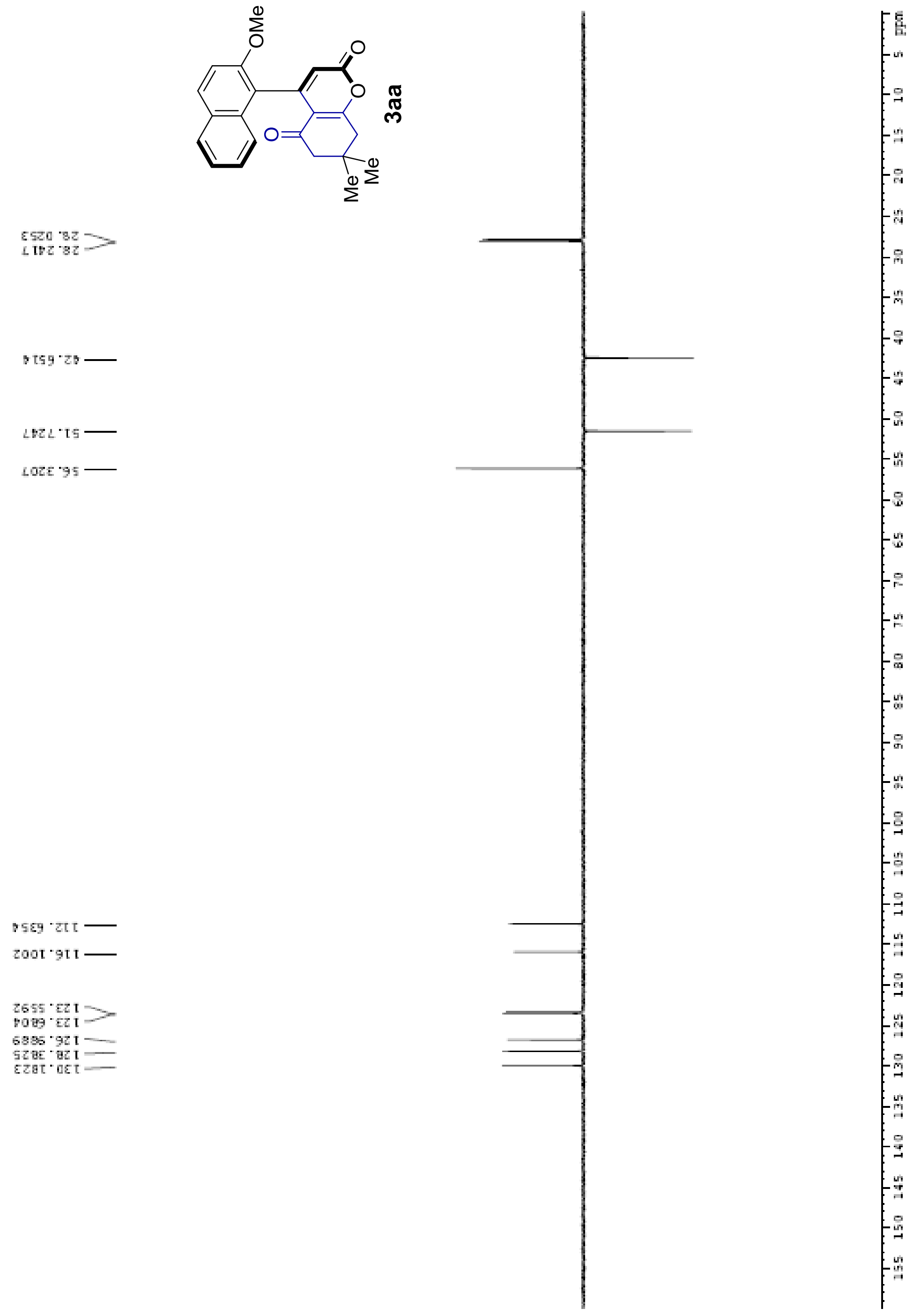

Supplementary Figure 4. HPLC Spectrum of racemic 3aa

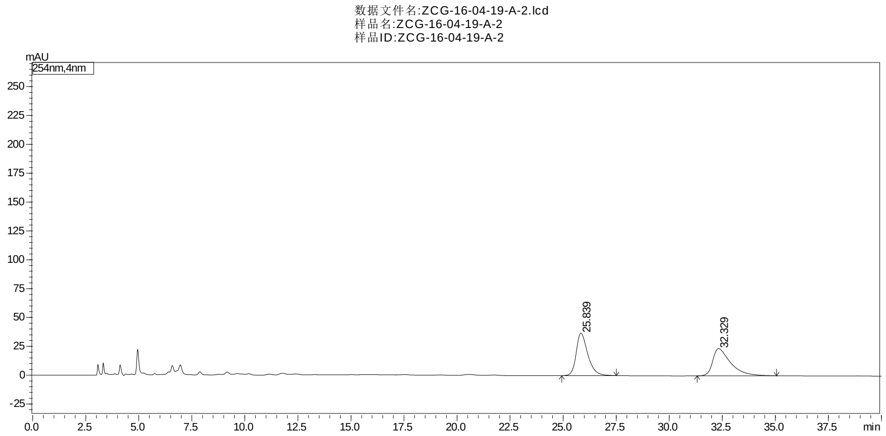

Peak#	Ret. Time	Area	Height	Area%	Height%
1	25.839	1400665	36773	50.361	60.927
2	32.329	1380602	23583	49.639	39.073
Total		2781267	60356	100.000	100.000

Supplementary Figure 5. HPLC Spectrum of 3aa

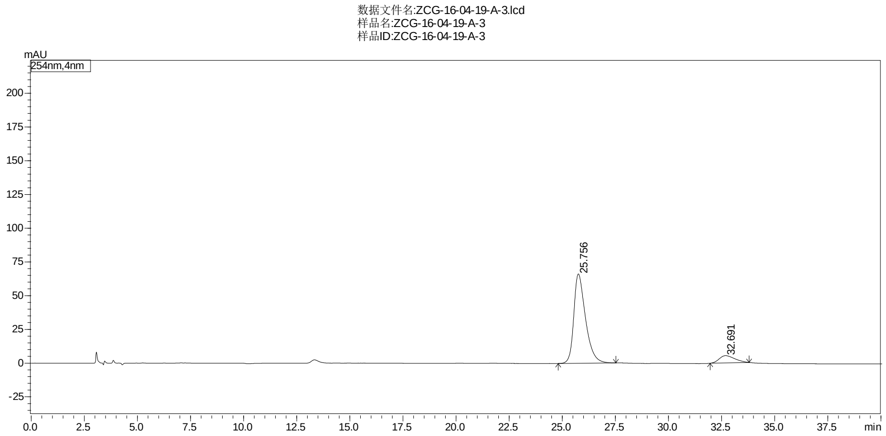

Peak#	Ret. Time	Area	Height	Area%	Height%
1	25.756	2538794	66218	90.251	92.489
2	32.691	274241	5378	9.749	7.511
Total		2813035	71596	100.000	100.000

Supplementary Figure 6. ^{1}H NMR Spectrum of 3ab

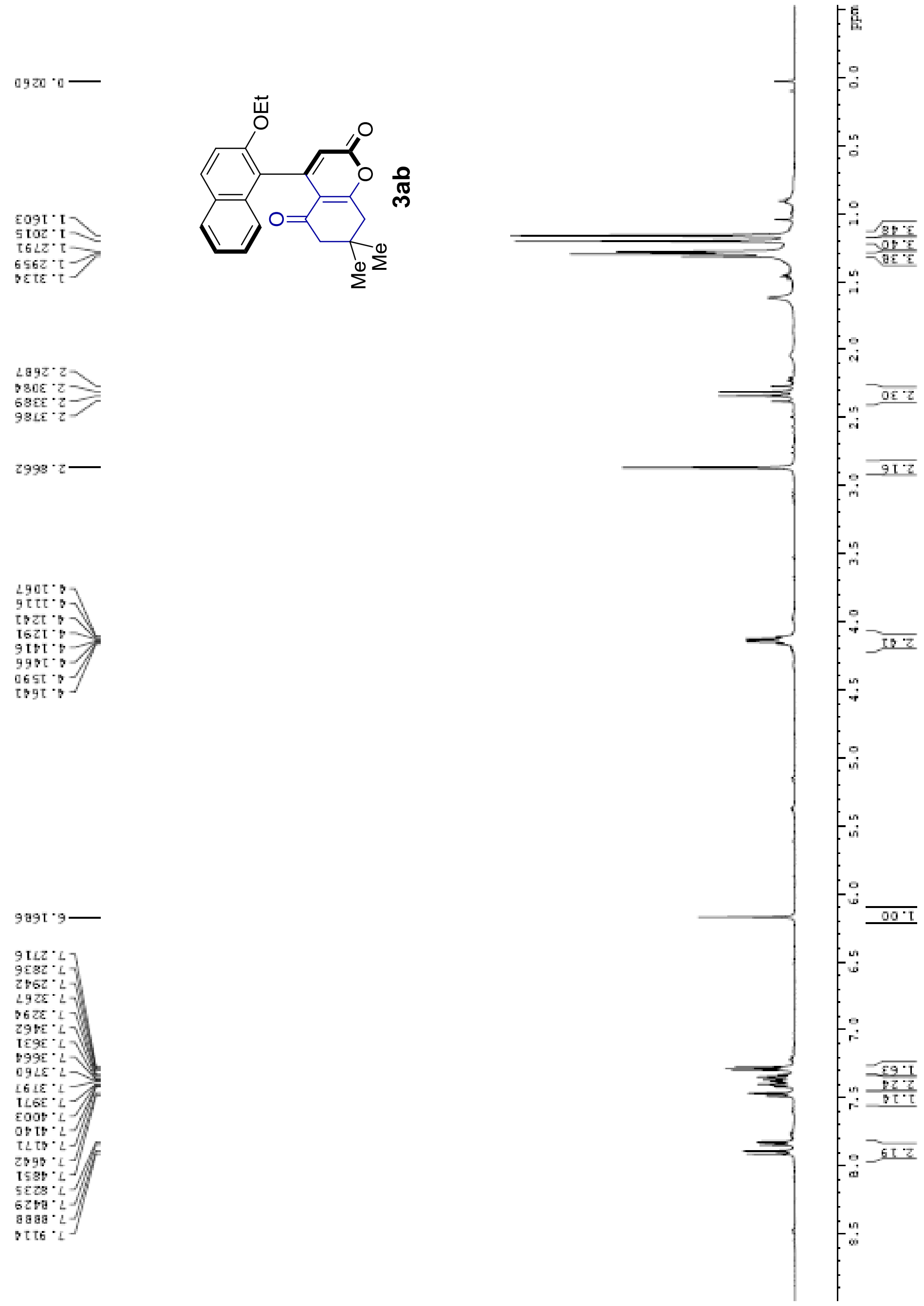

Supplementary Figure 7. ^{13}C NMR Spectrum of 3ab

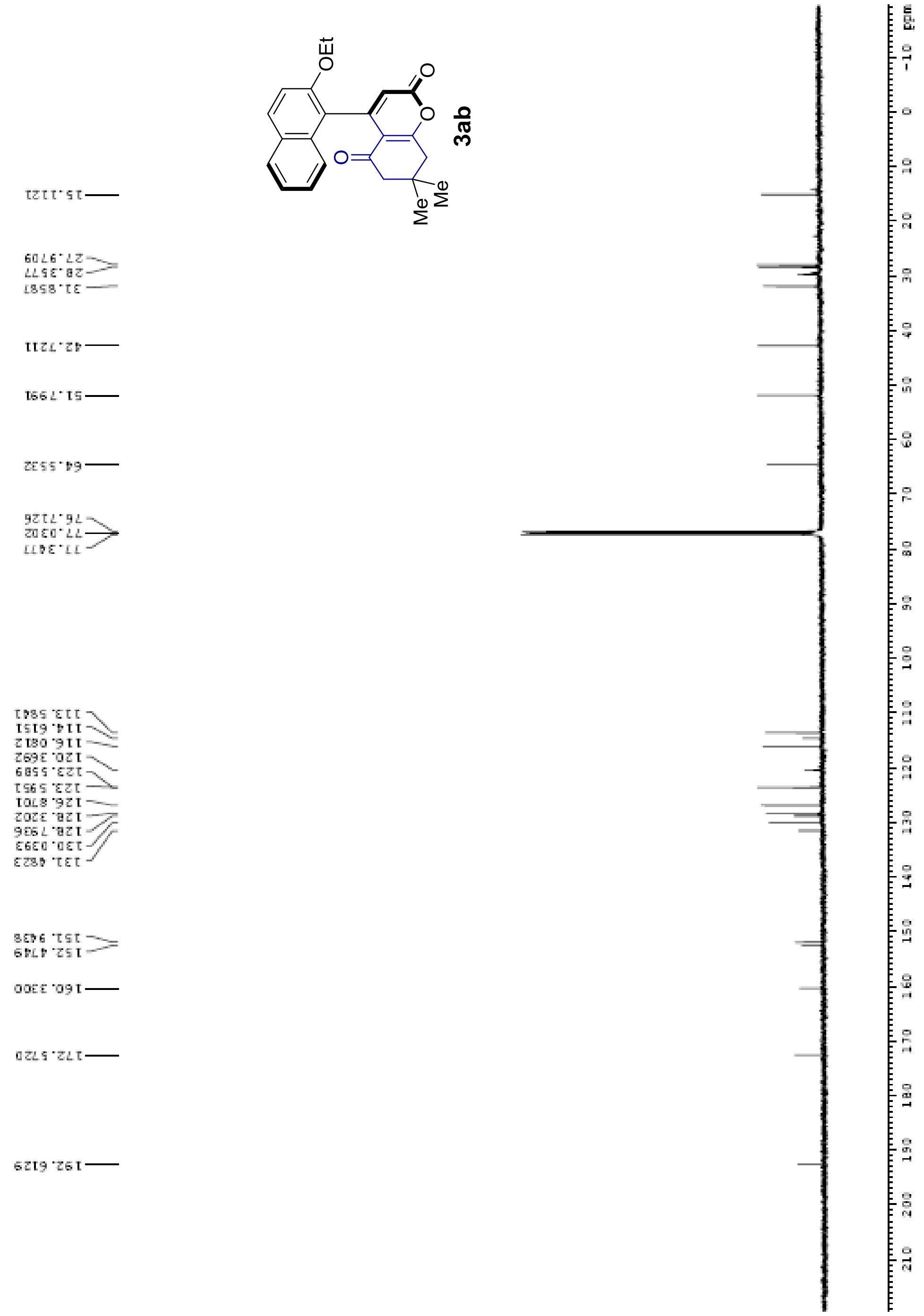

Supplementary Figure 8. HPLC Spectrum of racemic 3ab

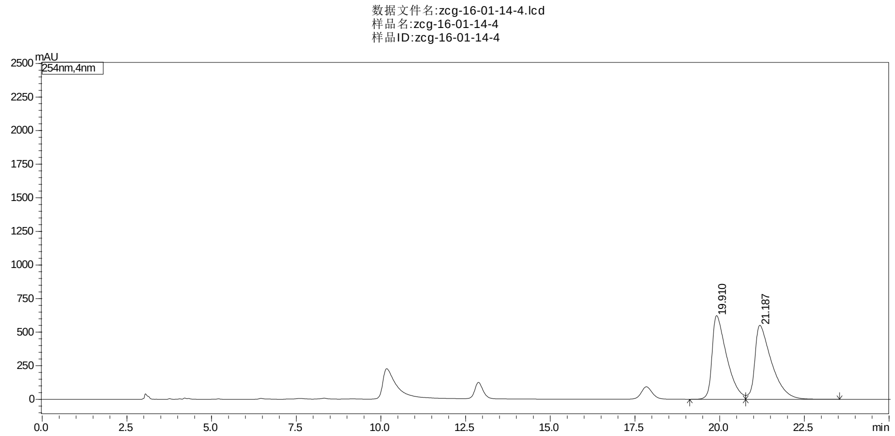

Peak#	Ret. Time	Area	Height	Area%	Height%
1	19.910	18726073	622115	49.638	53.056
2	21.187	18999274	550444	50.362	46.944
Total		37725347	1172559	100.000	100.000

Supplementary Figure 9. HPLC Spectrum of 3ab

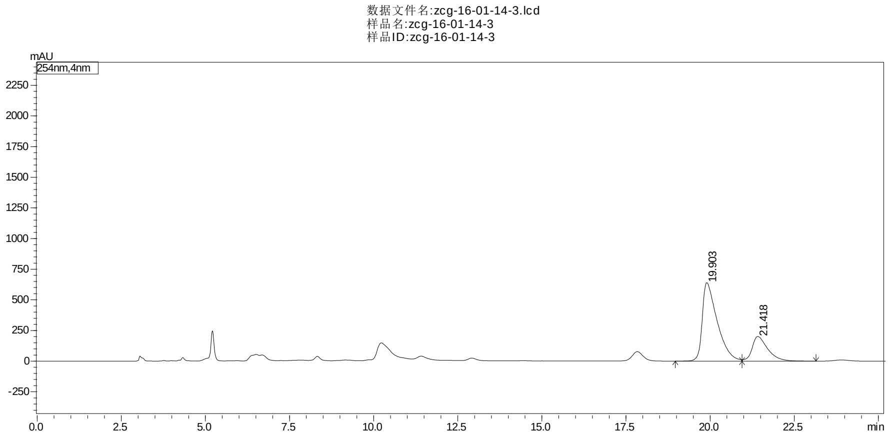

Peak#	Ret. Time	Area	Height	Area%	Height%
1	19.903	19775527	639391	75.218	76.039
2	21.418	6515522	201484	24.782	23.961
Total		26291049	840875	100.000	100.000

Supplementary Figure 10. ^{1}H NMR Spectrum of 3ac

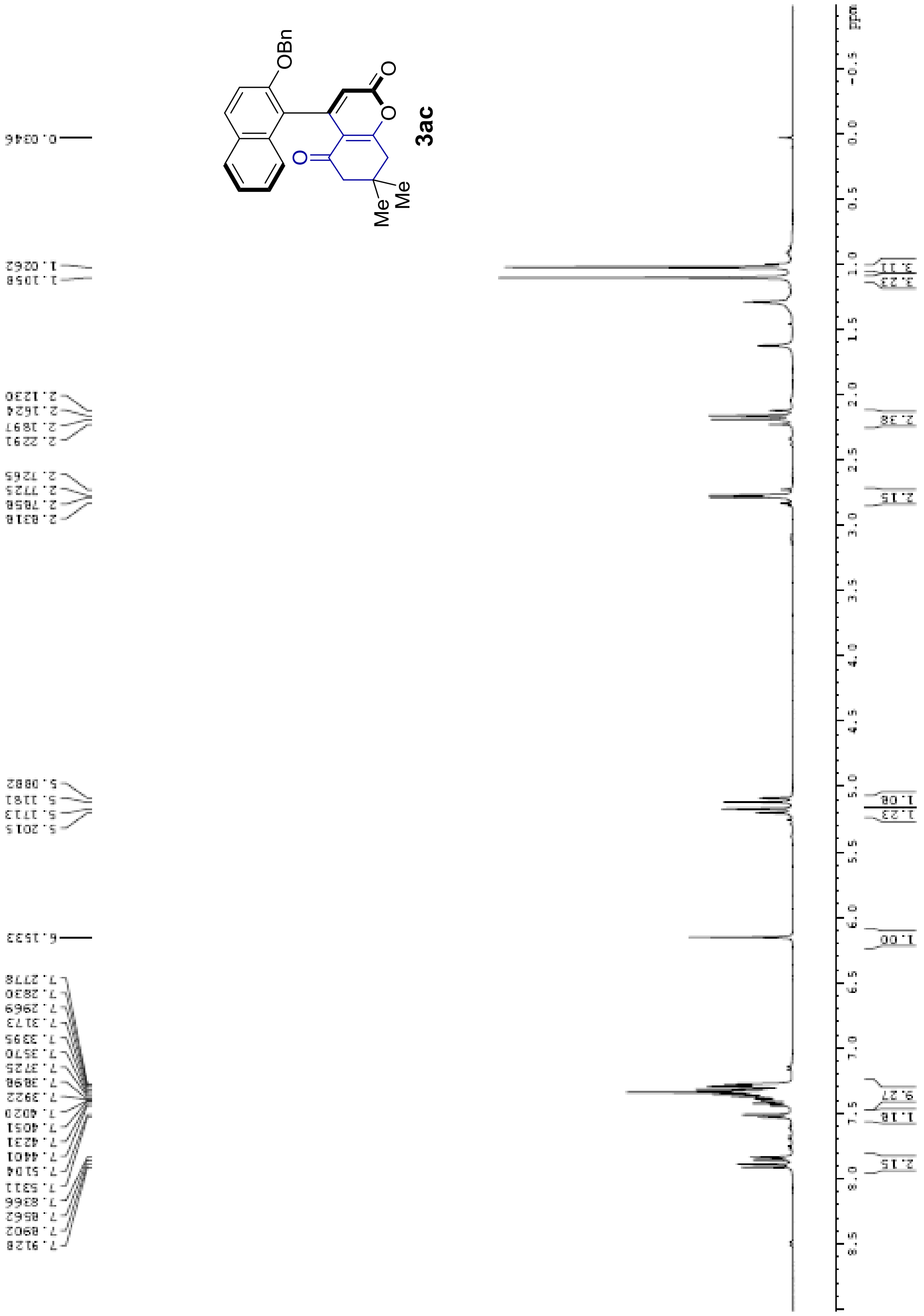

Supplementary Figure 11. ^{13}C NMR Spectrum of 3ac

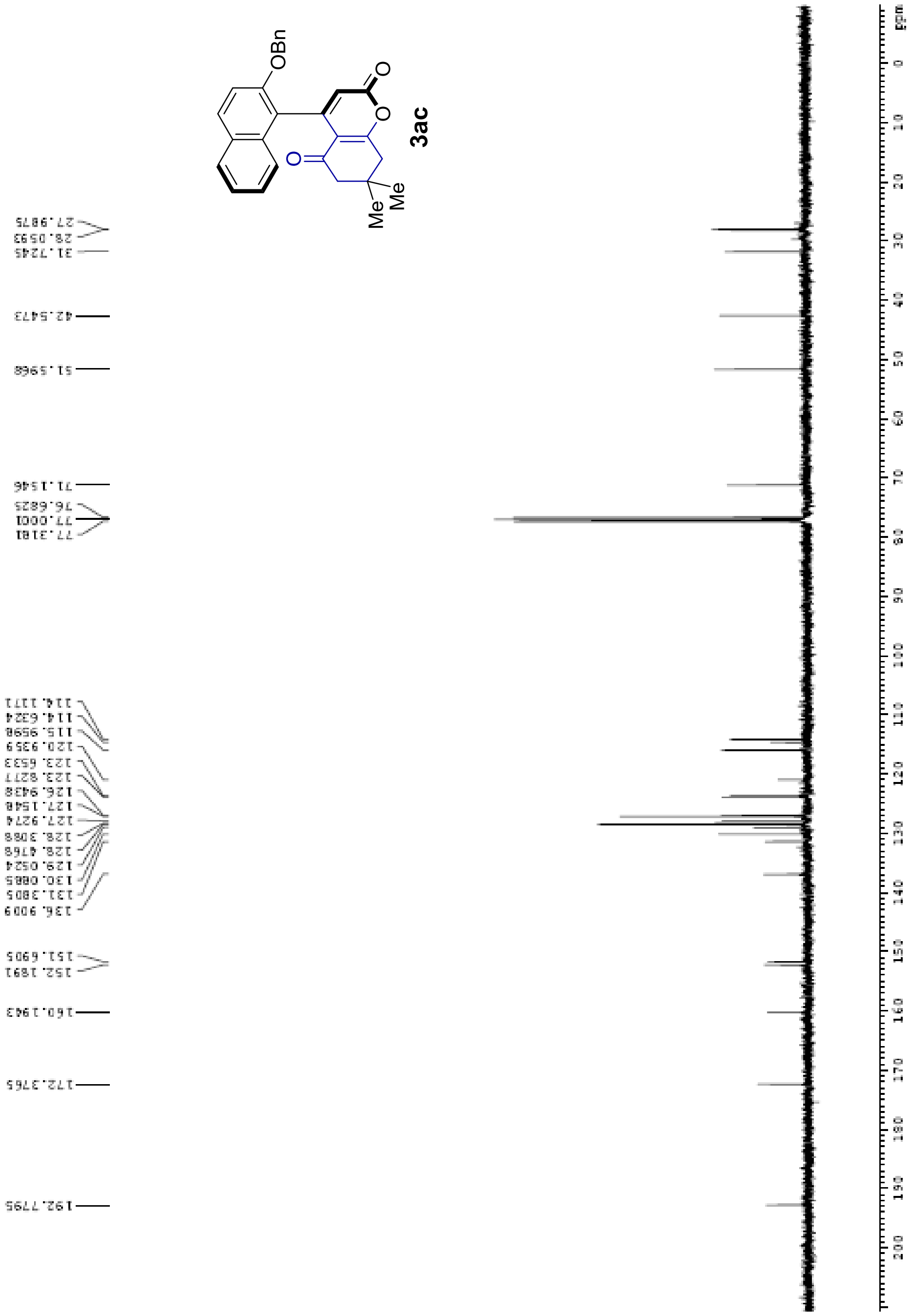

Supplementary Figure 12. HPLC Spectrum of racemic 3ac

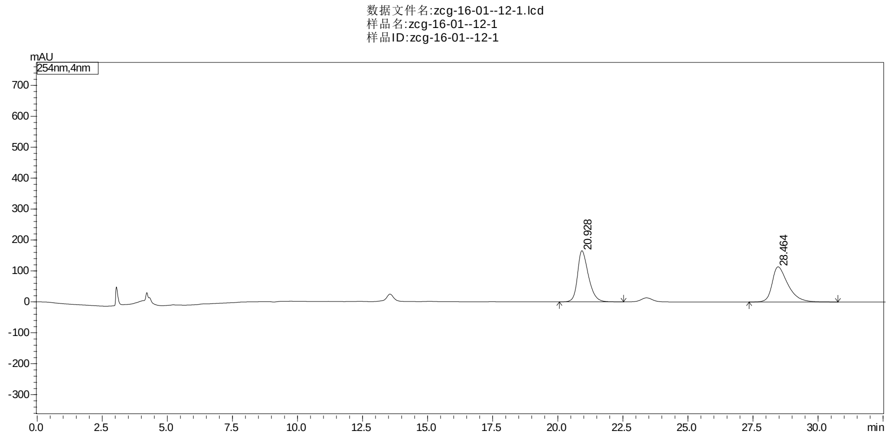

Peak#	Ret. Time	Area	Height	Area%	Height%
1	20.928	4829699	165349	50.083	59.224
2	28.464	4813697	113844	49.917	40.776
Total		9643396	279193	100.000	100.000

Supplementary Figure 13. HPLC Spectrum of 3ac

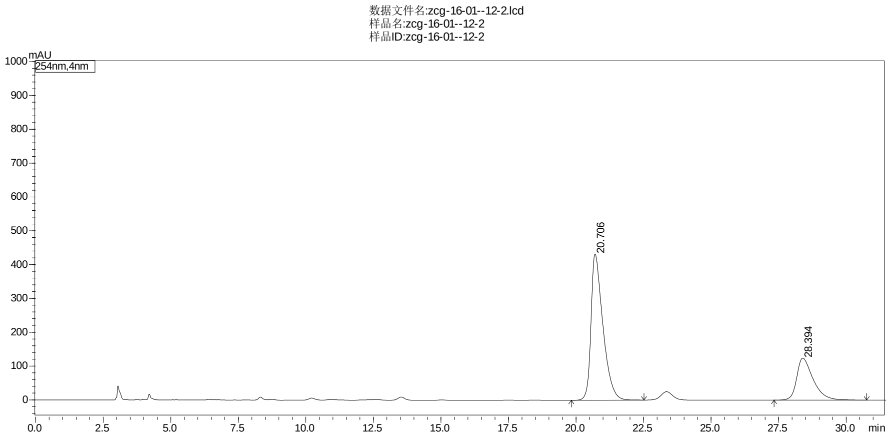

Peak#	Ret. Time	Area	Height	Area%	Height%
1	20.706	13282557	432700	71.569	77.683
2	28.394	5276502	124307	28.431	22.317
Total		18559059	557007	100.000	100.000

Supplementary Figure 14. ^{1}H NMR Spectrum of 3ba

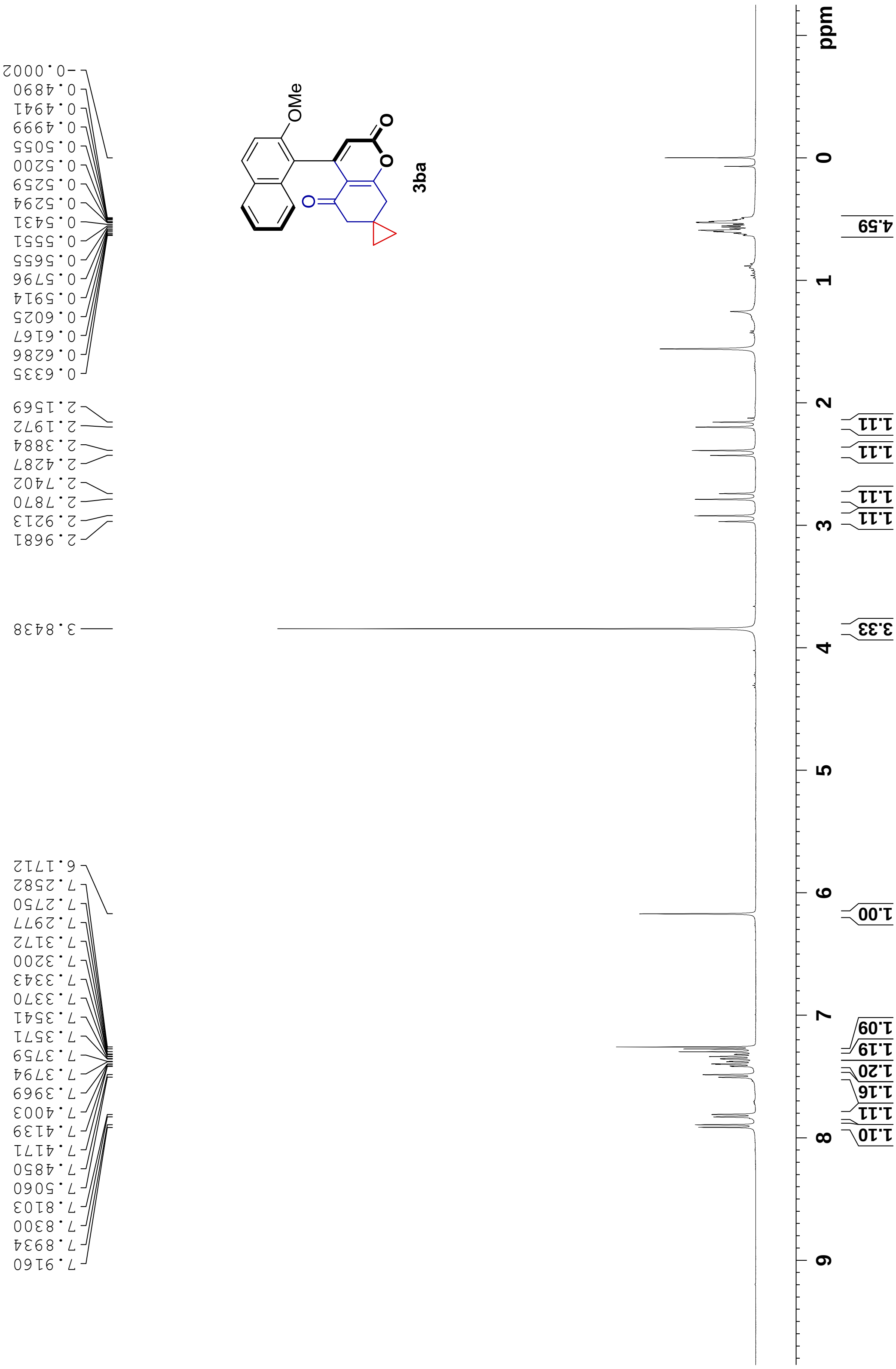

Supplementary Figure 15. ^{13}C NMR Spectrum of 3ba

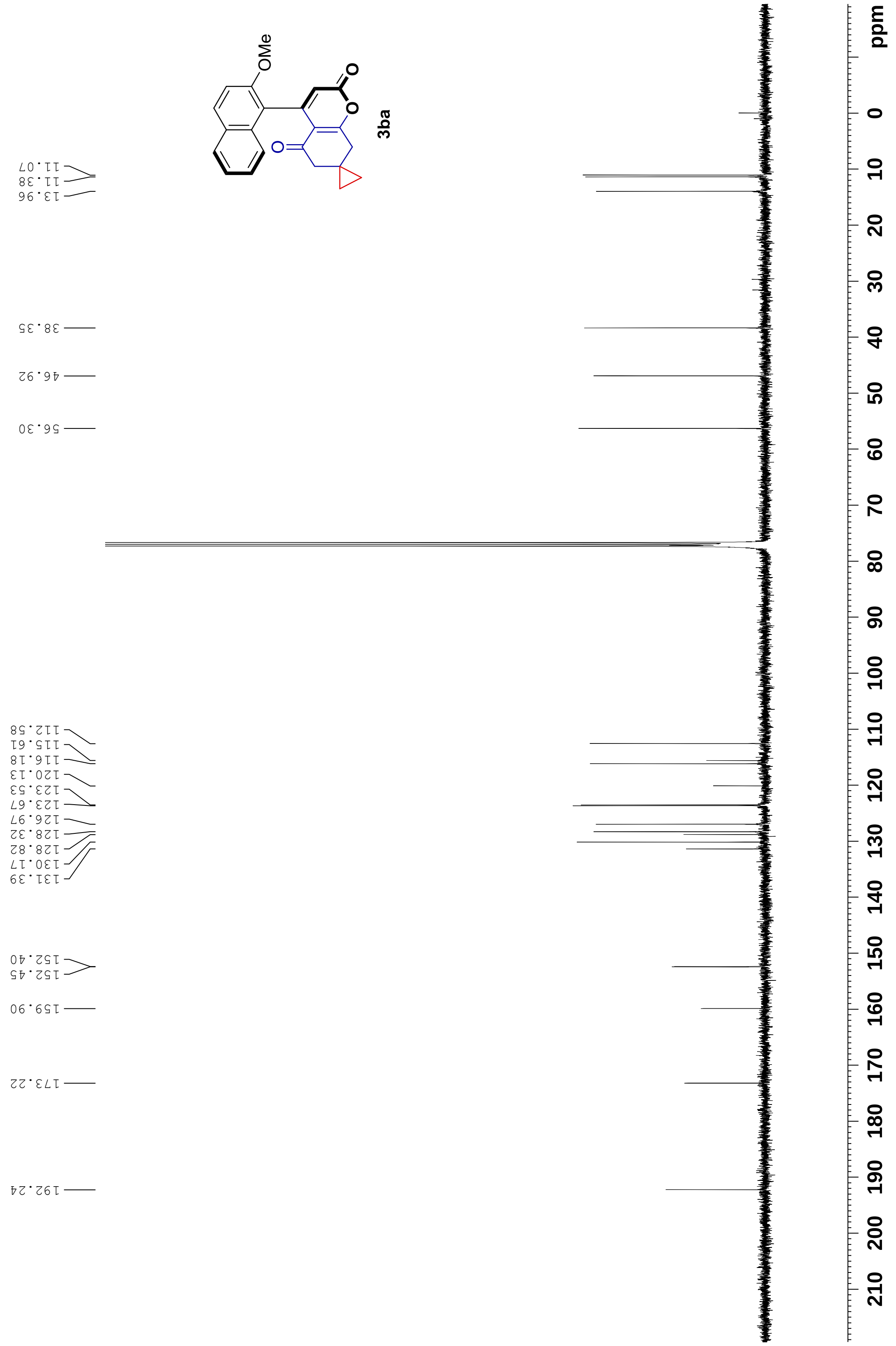

Supplementary Figure 16. HPLC Spectrum of racemic 3ba

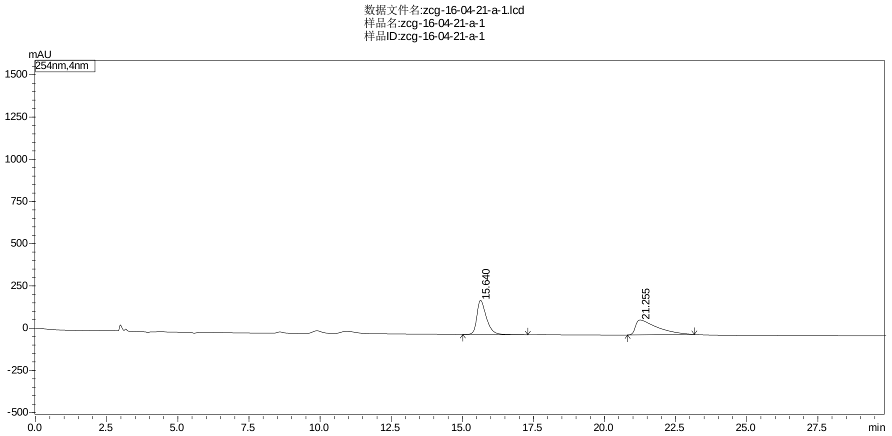

Peak#	Ret. Time	Area	Height	Area%	Height%
1	15.640	4700329	202588	50.087	69.689
2	21.255	4684062	88115	49.913	30.311
Total		9384391	290703	100.000	100.000

Supplementary Figure 17. HPLC Spectrum of 3ba

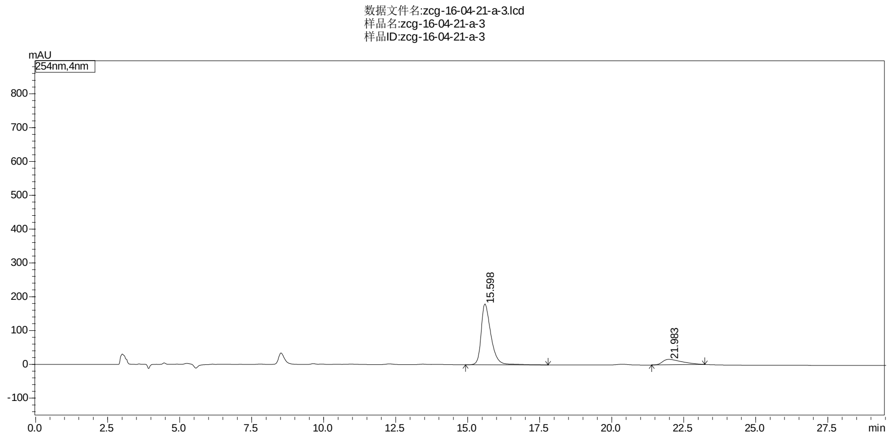

Peak#	Ret. Time	Area	Height	Area%	Height%
1	15.598	4248253	179868	84.786	91.778
2	21.983	762314	16114	15.214	8.222
Total		5010567	195981	100.000	100.000

Supplementary Figure 18. ^{1}H NMR Spectrum of 3ca

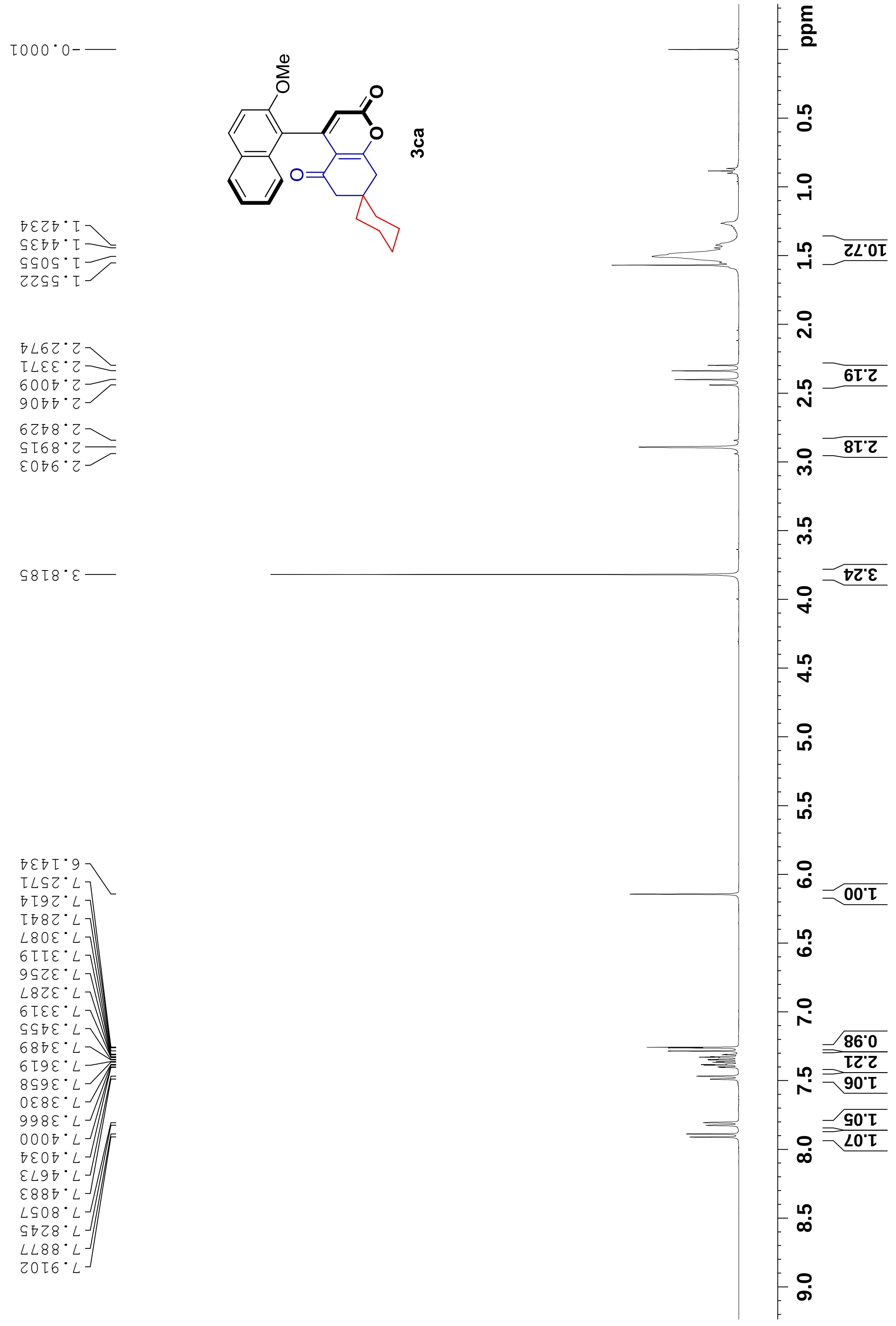

Supplementary Figure 19. ^{13}C NMR Spectrum of 3ca

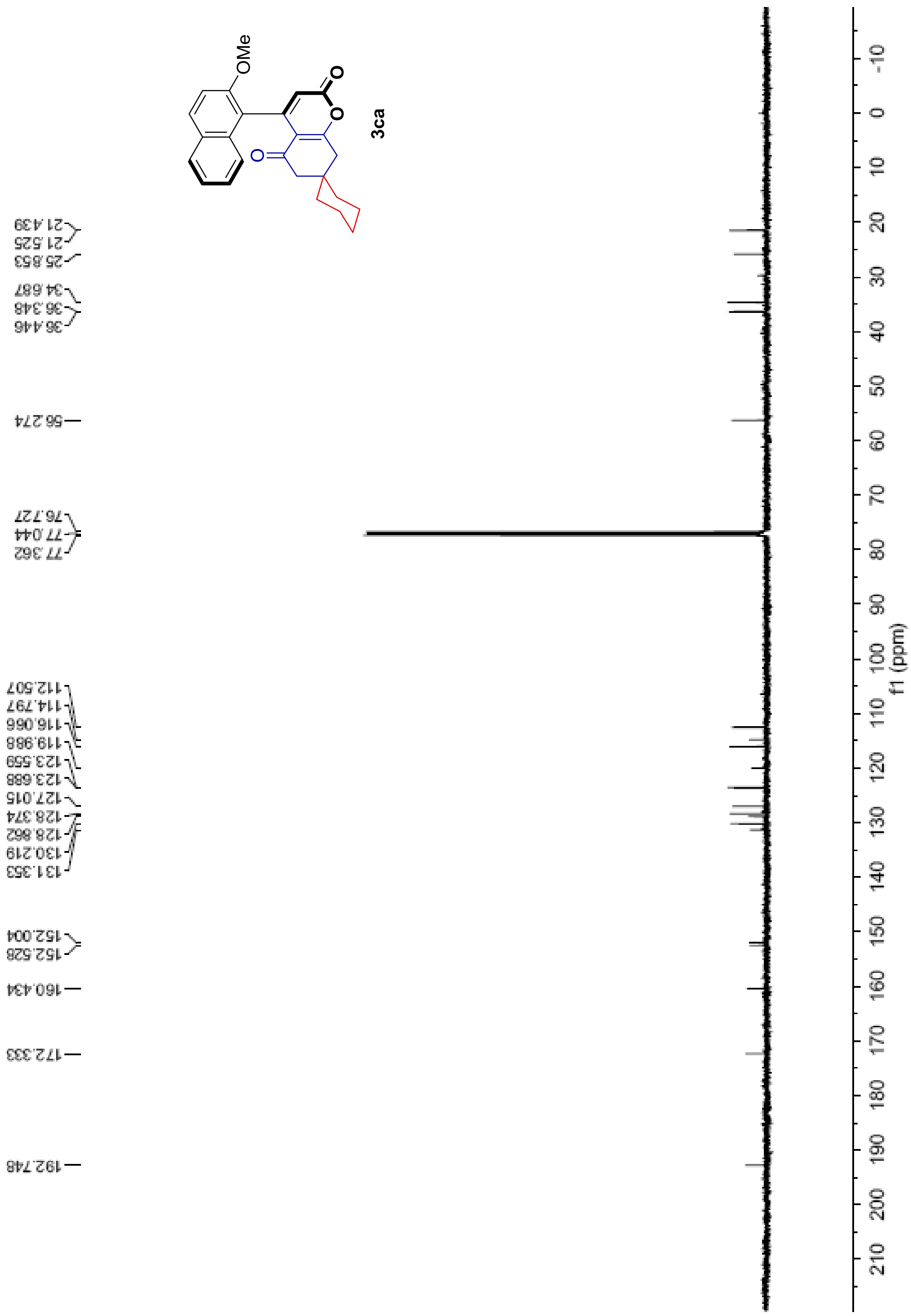

Supplementary Figure 20. HPLC Spectrum of racemic 3ca

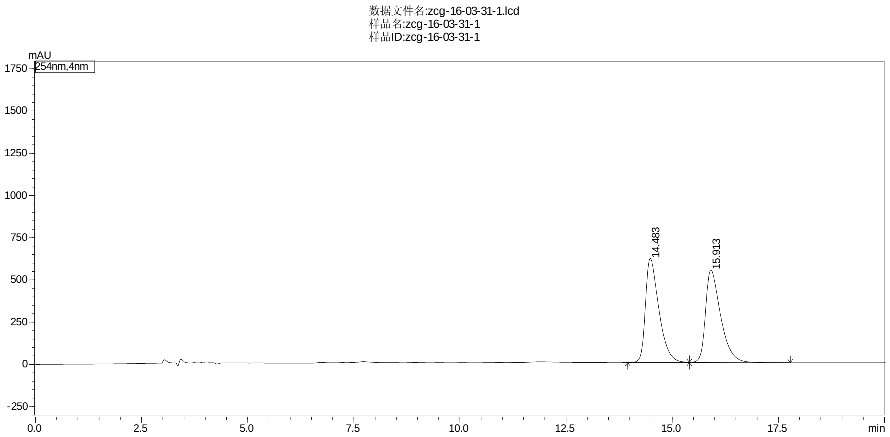

Peak#	Ret. Time	Area	Height	Area%	Height%
1	14.483	13549587	615635	49.891	52.885
2	15.913	13608595	548458	50.109	47.115
Total		27158182	1164093	100.000	100.000

Supplementary Figure 21. HPLC Spectrum of 3ca

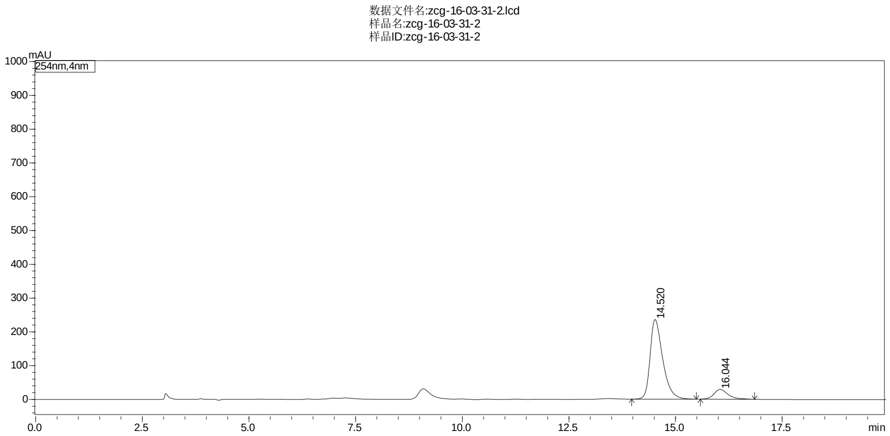

Peak#	Ret. Time	Area	Height	Area%	Height%
1	14.520	4850380	235835	87.890	89.067
2	16.044	668331	28948	12.110	10.933
Total		5518711	264783	100.000	100.000

Supplementary Figure 22. ^{1}H NMR Spectrum of 3da

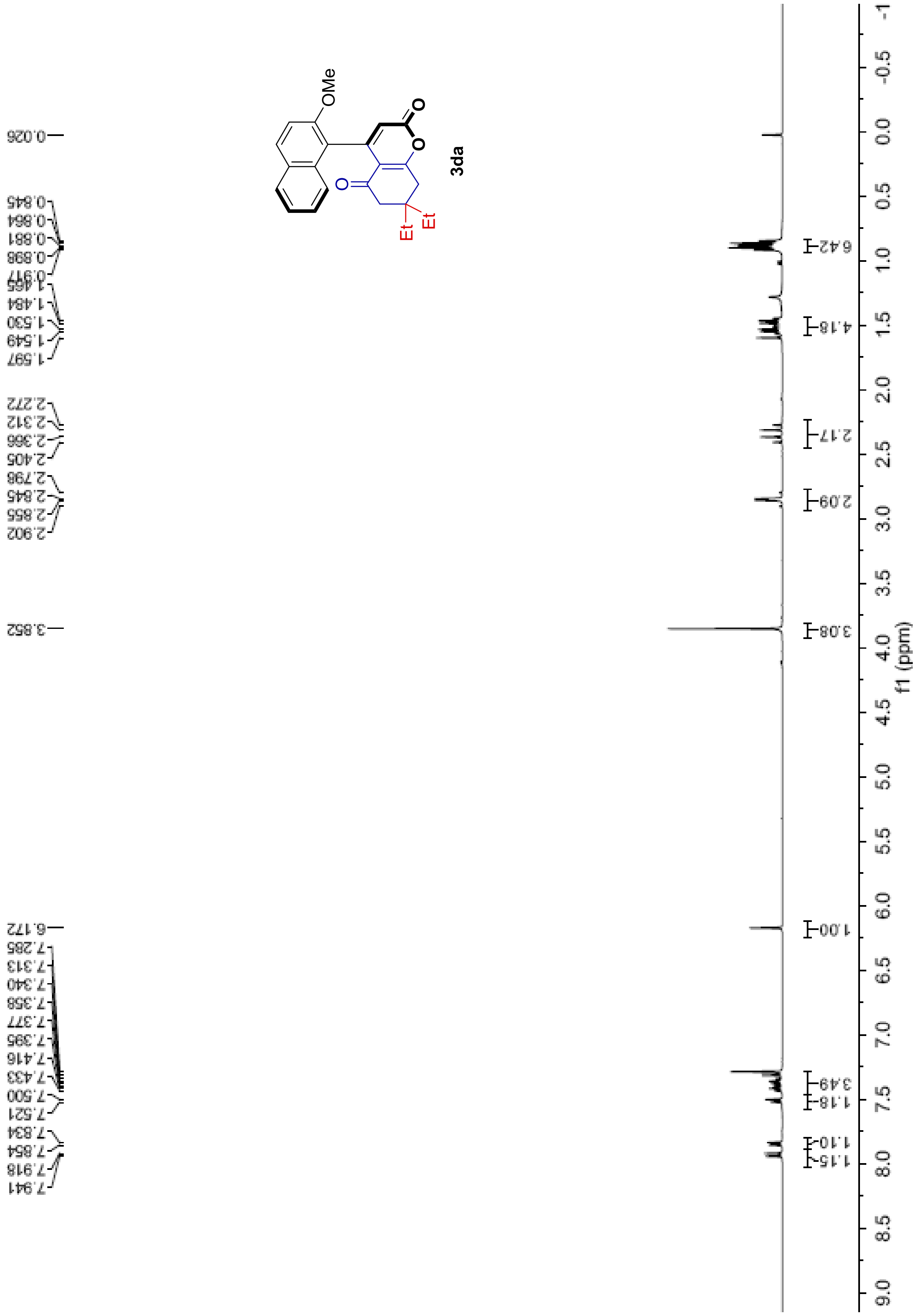

Supplementary Figure 23. ^{13}C NMR Spectrum of 3da

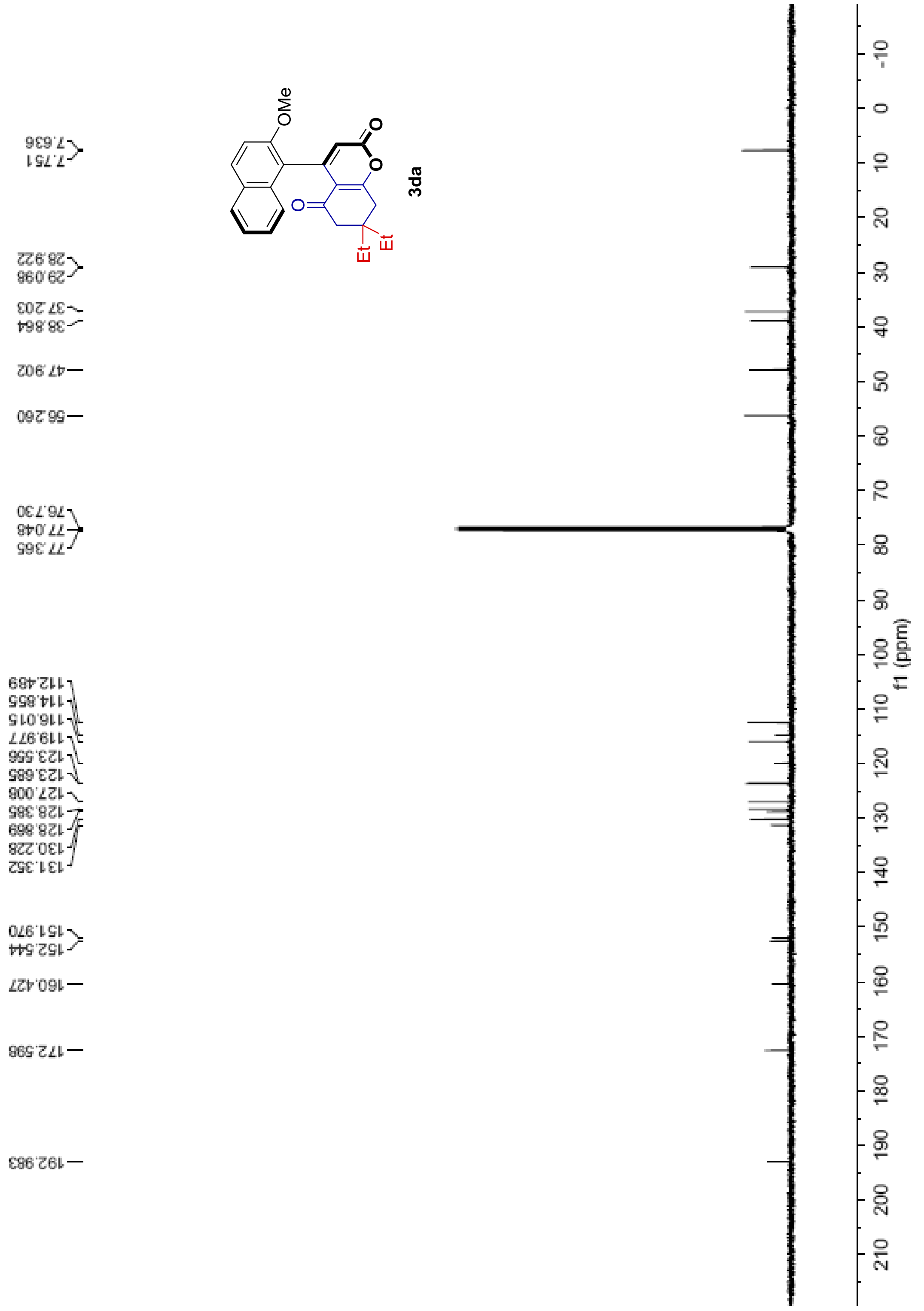

Supplementary Figure 24. HPLC Spectrum of racemic 3da

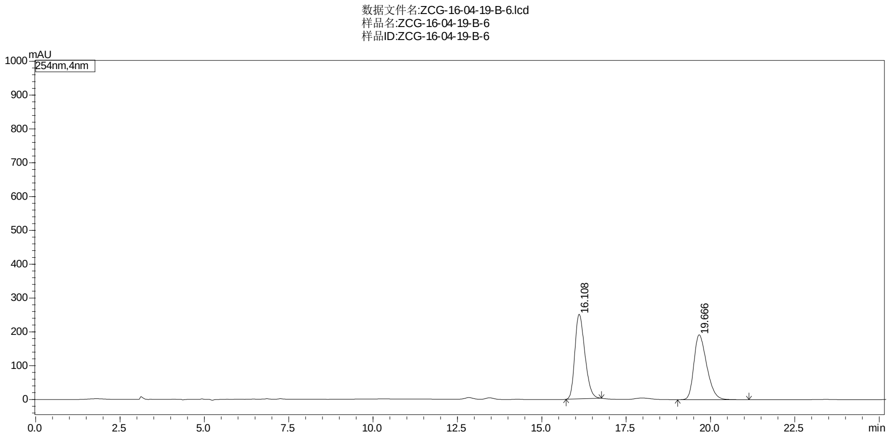

Peak#	Ret. Time	Area	Height	Area%	Height%
1	16.108	5162585	250179	50.401	56.606
2	19.666	5080481	191788	49.599	43.394
Total		10243066	441968	100.000	100.000

Supplementary Figure 25. HPLC Spectrum of 3da

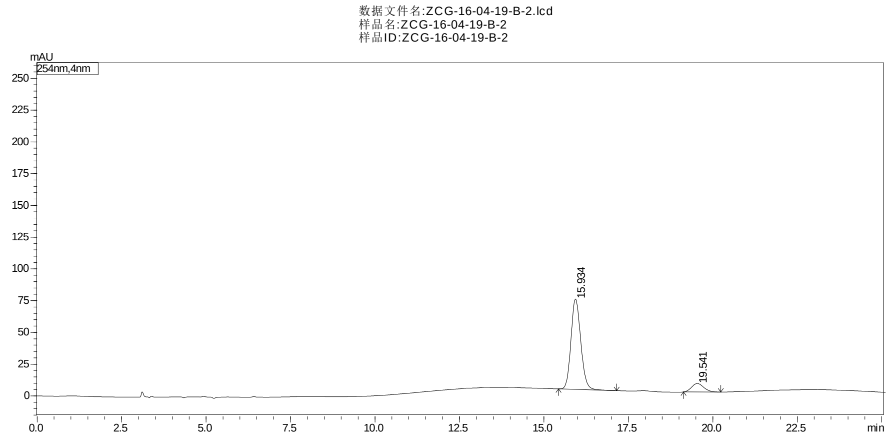

Peak#	Ret. Time	Area	Height	Area%	Height%
1	15.934	1438230	71230	89.734	91.454
2	19.541	164532	6656	10.266	8.546
Total		1602762	77887	100.000	100.000

Supplementary Figure 26. ^{1}H NMR Spectrum of 3ea

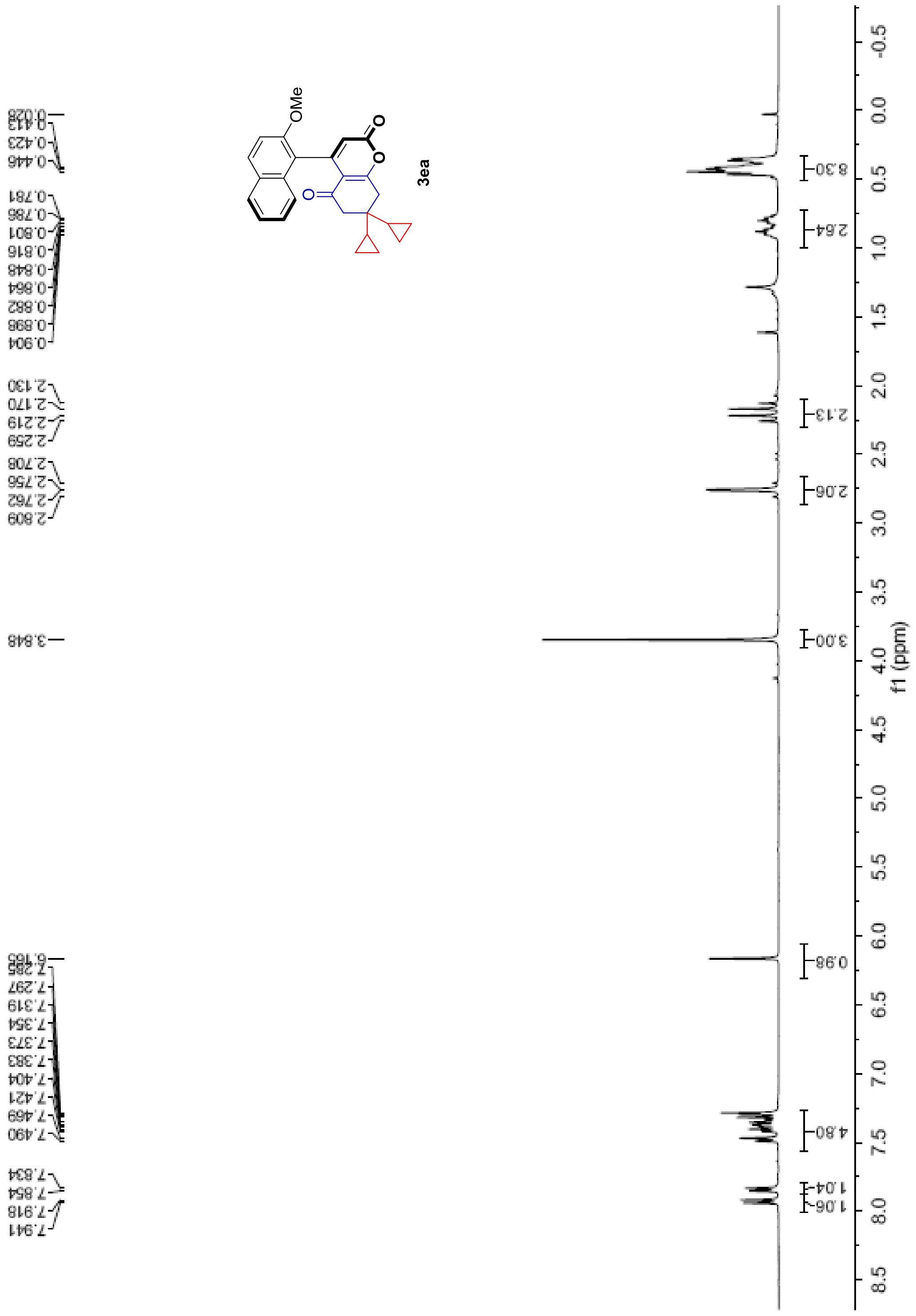

Supplementary Figure 27. ^{13}C NMR Spectrum of 3ea

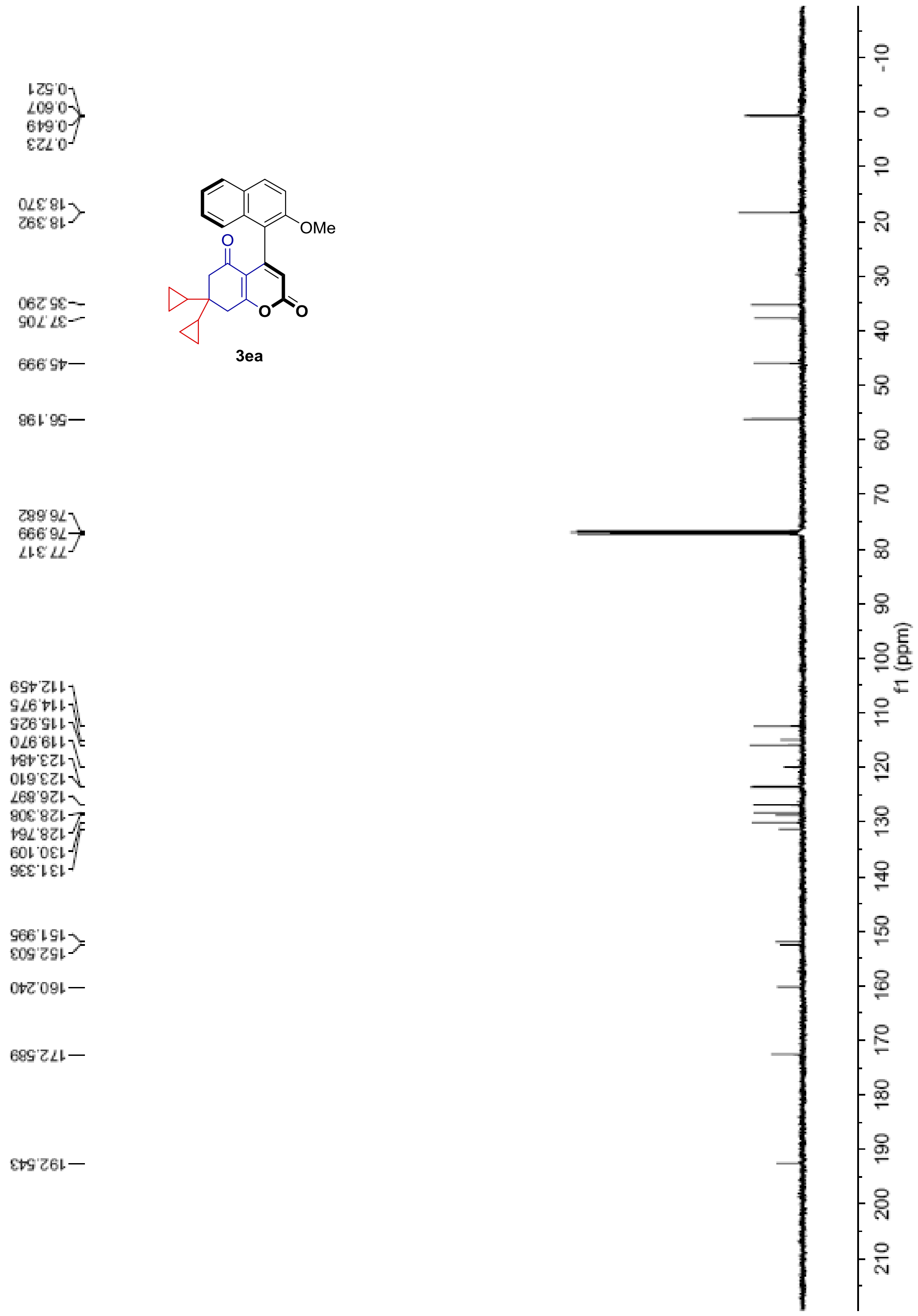

Supplementary Figure 28. HPLC Spectrum of racemic 3ea

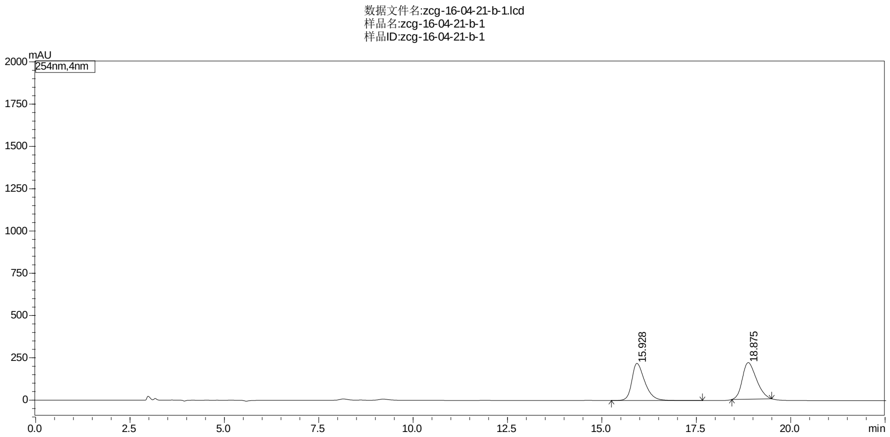

Peak#	Ret. Time	Area	Height	Area%	Height%
1	15.928	5282308	219899	48.496	50.406
2	18.875	5609880	216358	51.504	49.594
Total		10892188	436258	100.000	100.000

Supplementary Figure 29. HPLC Spectrum of 3ea

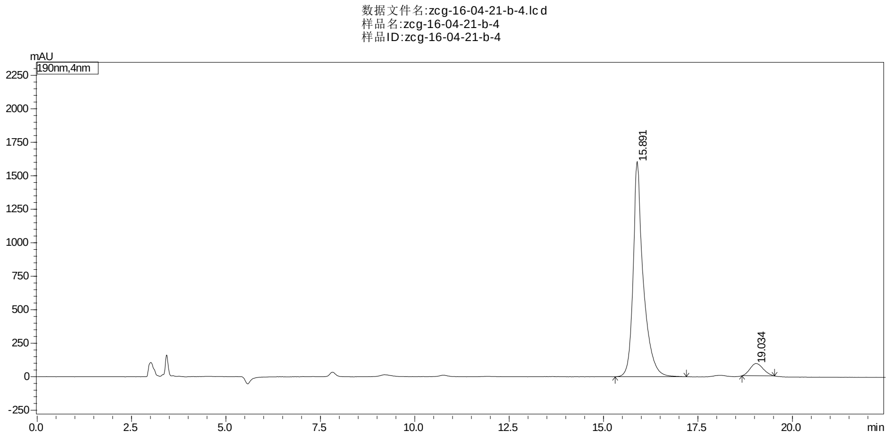

Peak#	Ret. Time	Area	Height	Area%	Height%
1	15.891	29942799	1607886	93.062	94.601
2	19.034	2232152	91764	6.938	5.399
Total		32174951	1699649	100.000	100.000

Supplementary Figure 30. ^{1}H NMR Spectrum of 3fa

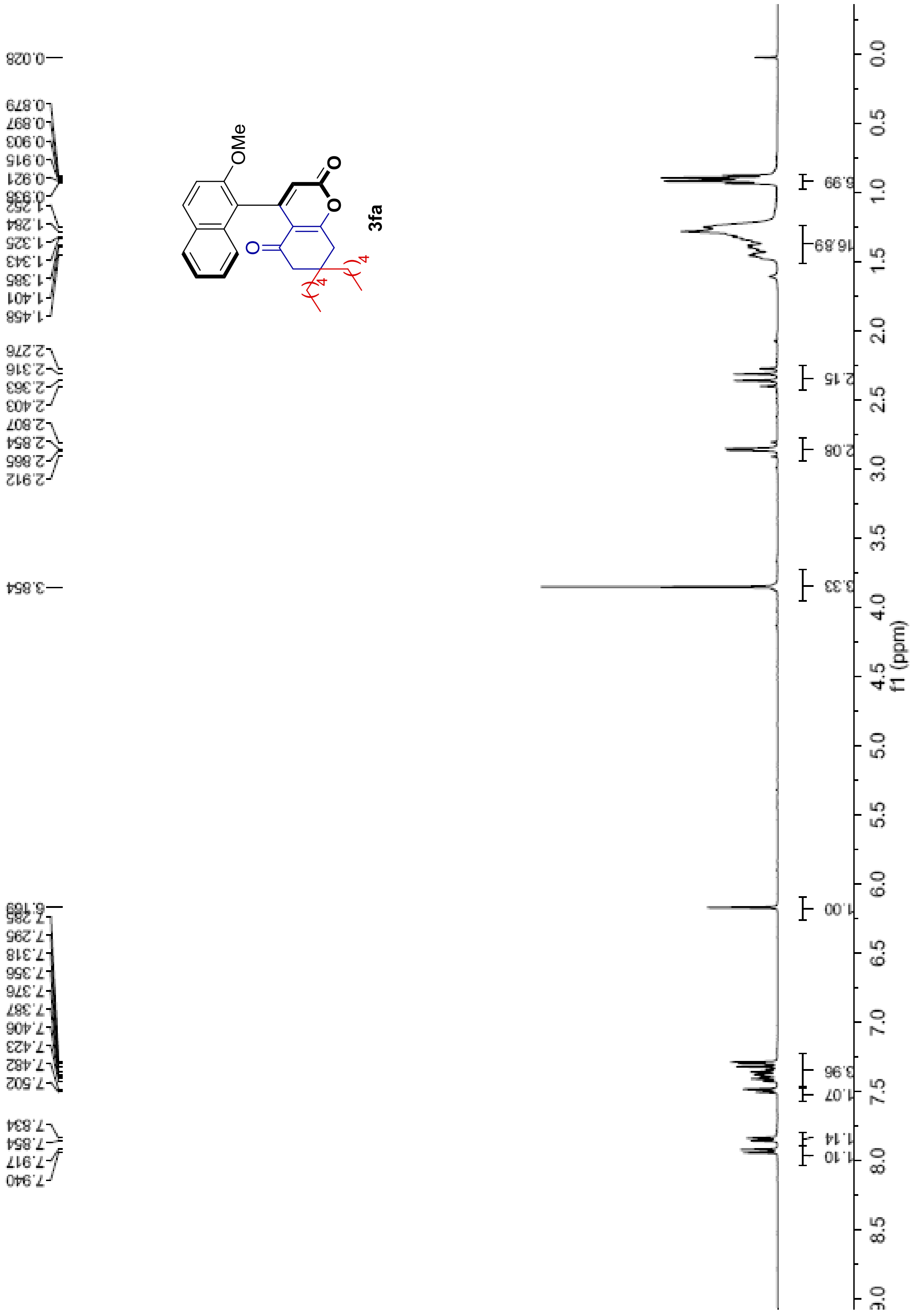

Supplementary Figure 31. ^{13}C NMR Spectrum of 3fa

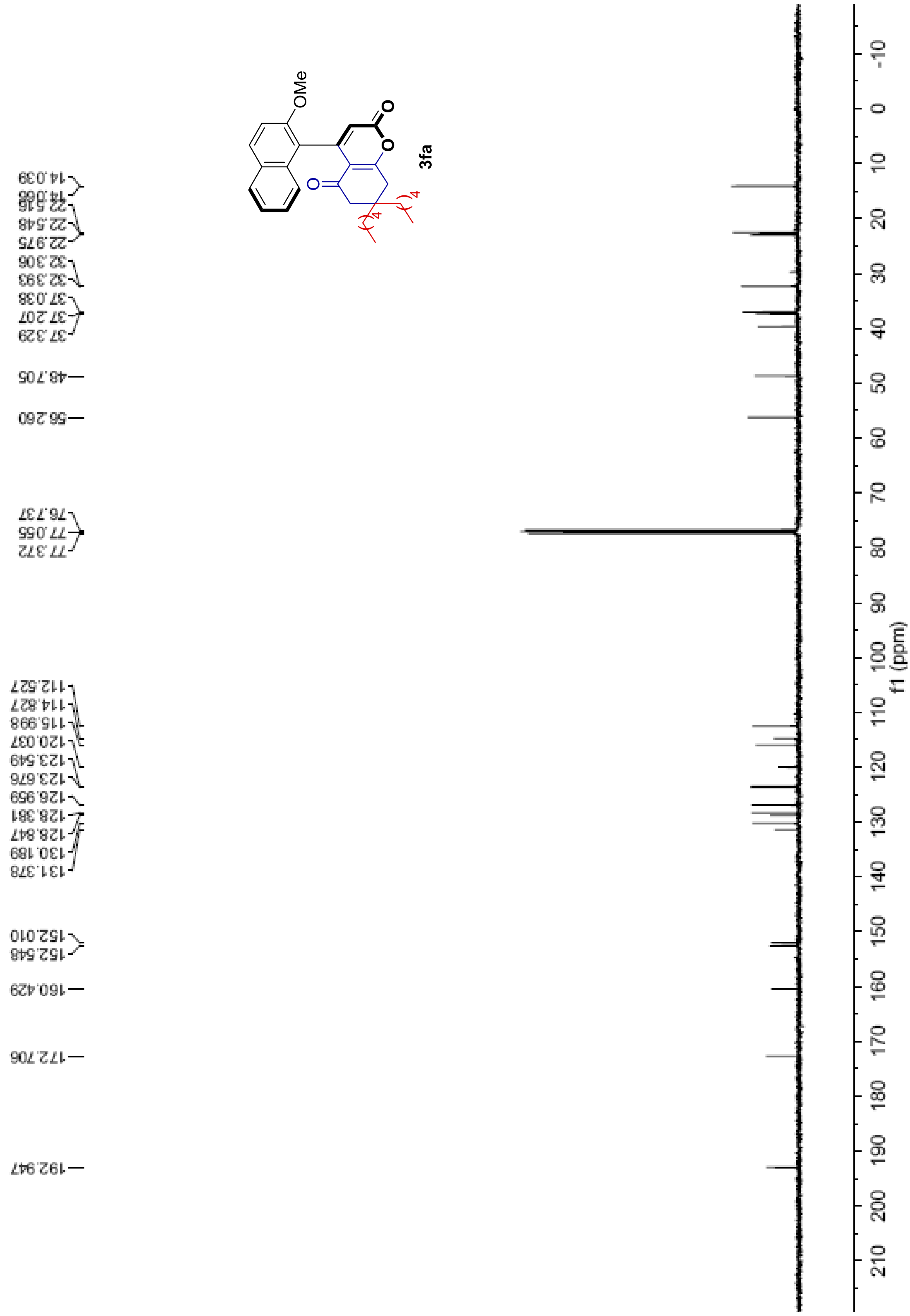

Supplementary Figure 32. HPLC Spectrum of racemic 3fa

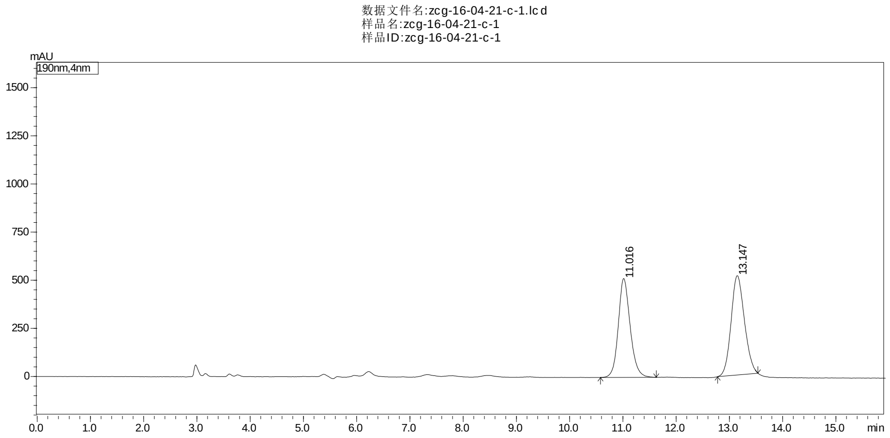

Peak#	Ret. Time	Area	Height	Area%	Height%
1	11.017	2524199	159427	47.733	50.165
2	13.149	2763949	158377	52.267	49.835
Total		5288147	317803	100.000	100.000

Supplementary Figure 33. HPLC Spectrum of 3fa

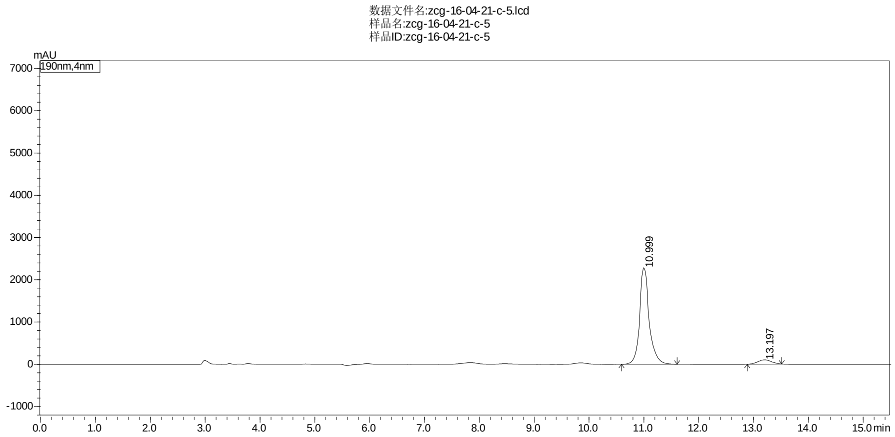

Peak#	Ret. Time	Area	Height	Area%	Height%
1	10.999	27720307	2286103	94.079	95.728
2	13.197	1744652	102032	5.921	4.272
Total		29464960	2388135	100.000	100.000

Supplementary Figure 34. ^{1}H NMR Spectrum of 3ga

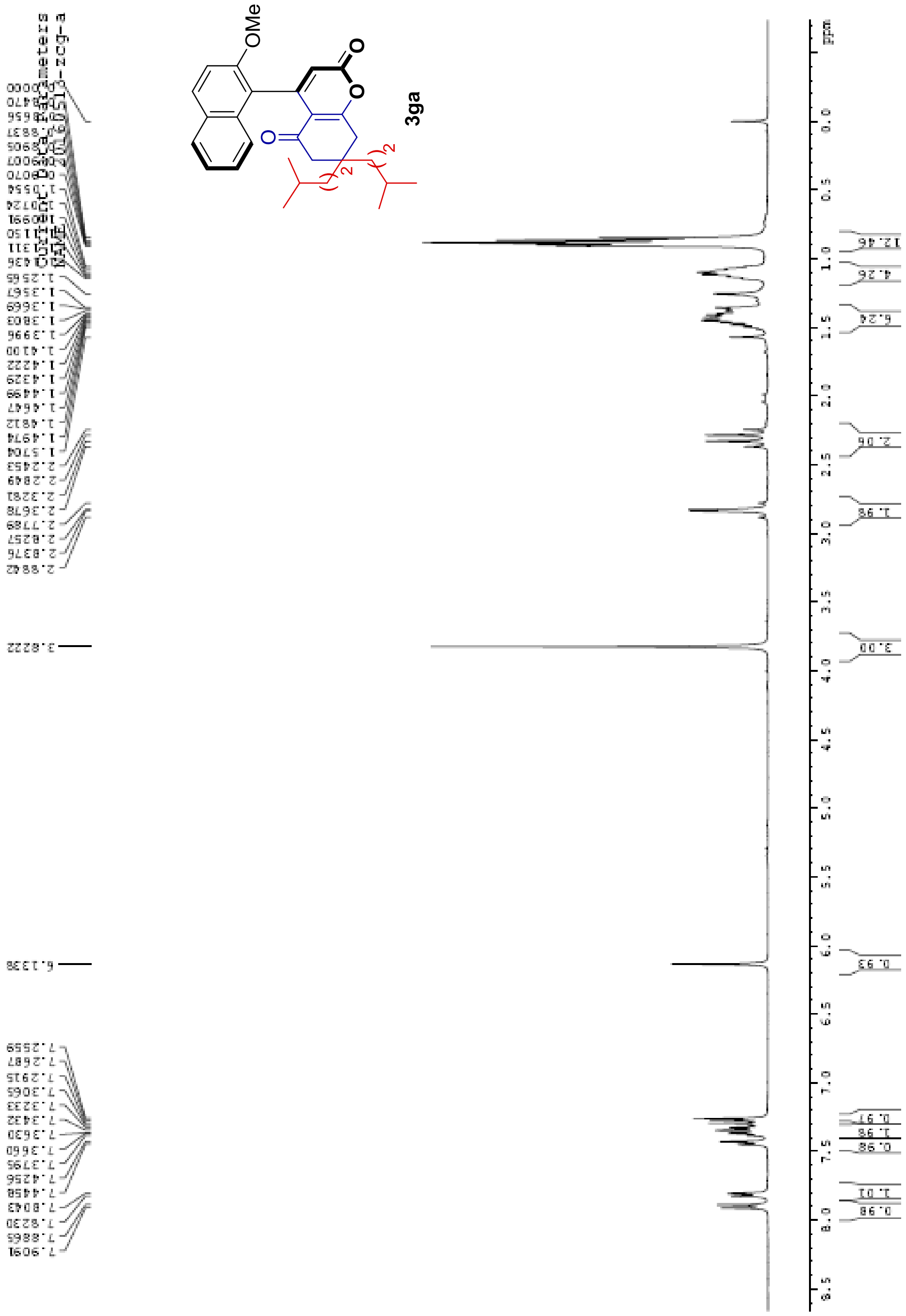

Supplementary Figure 35. ^{13}C NMR Spectrum of 3ga

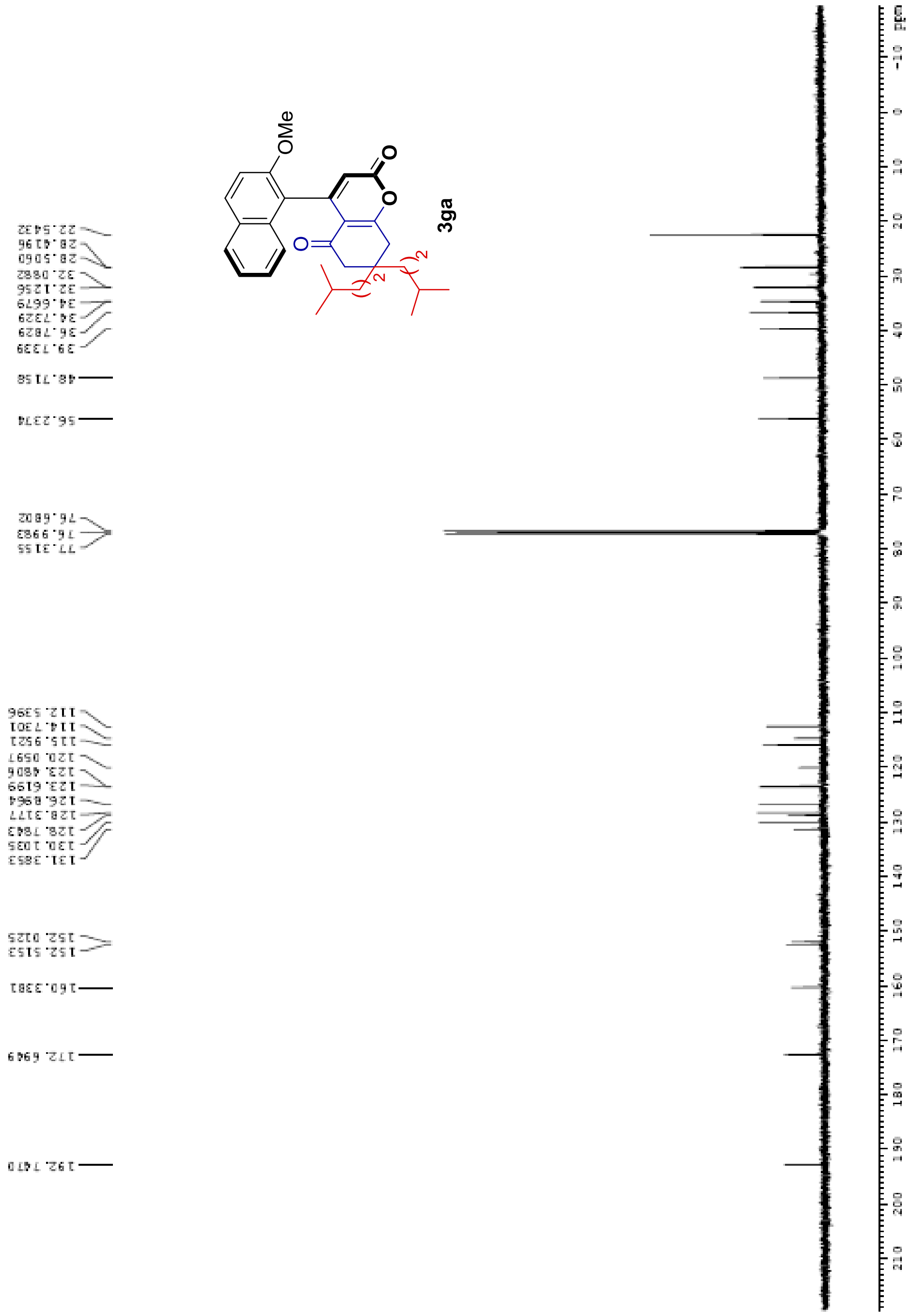

Supplementary Figure 36. HPLC Spectrum of racemic 3ga

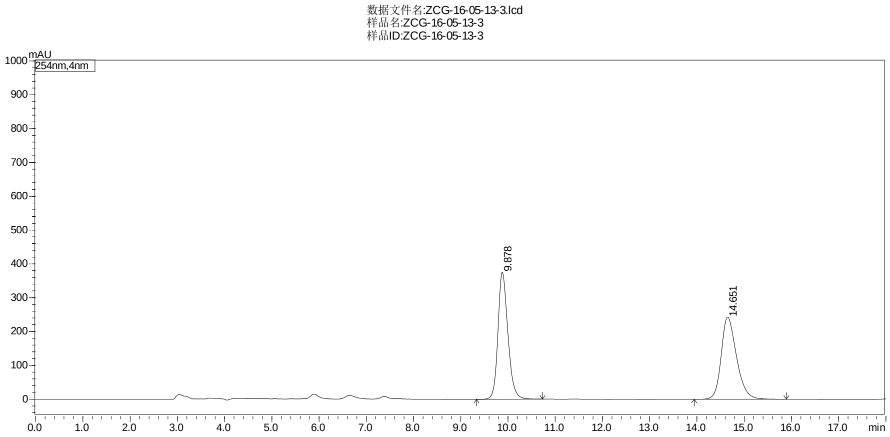

Peak#	Ret. Time	Area	Height	Area%	Height%
1	9.878	5504169	375739	50.168	60.712
2	14.651	5467217	243148	49.832	39.288
Total		10971386	618887	100.000	100.000

Supplementary Figure 37. HPLC Spectrum of 3ga

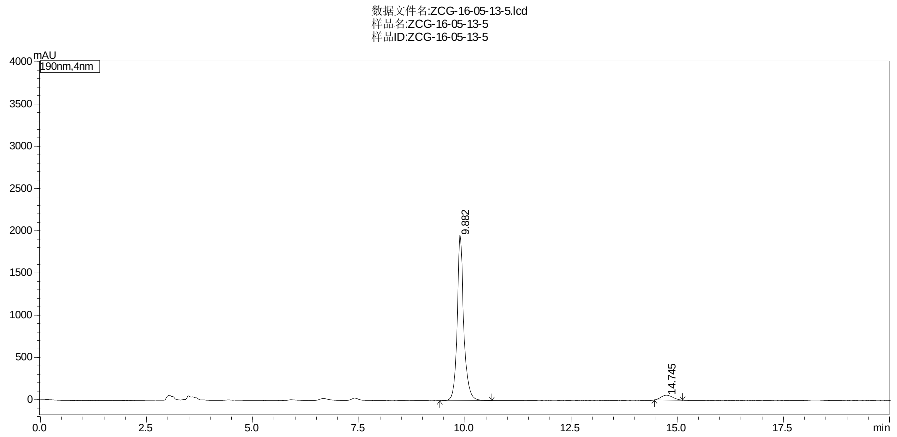

Peak#	Ret. Time	Area	Height	Area%	Height%
1	9.882	21124512	1957896	95.042	97.144
2	14.745	1102025	57569	4.958	2.856
Total		22226537	2015465	100.000	100.000

Supplementary Figure 38. ^{1}H NMR Spectrum of 3gd

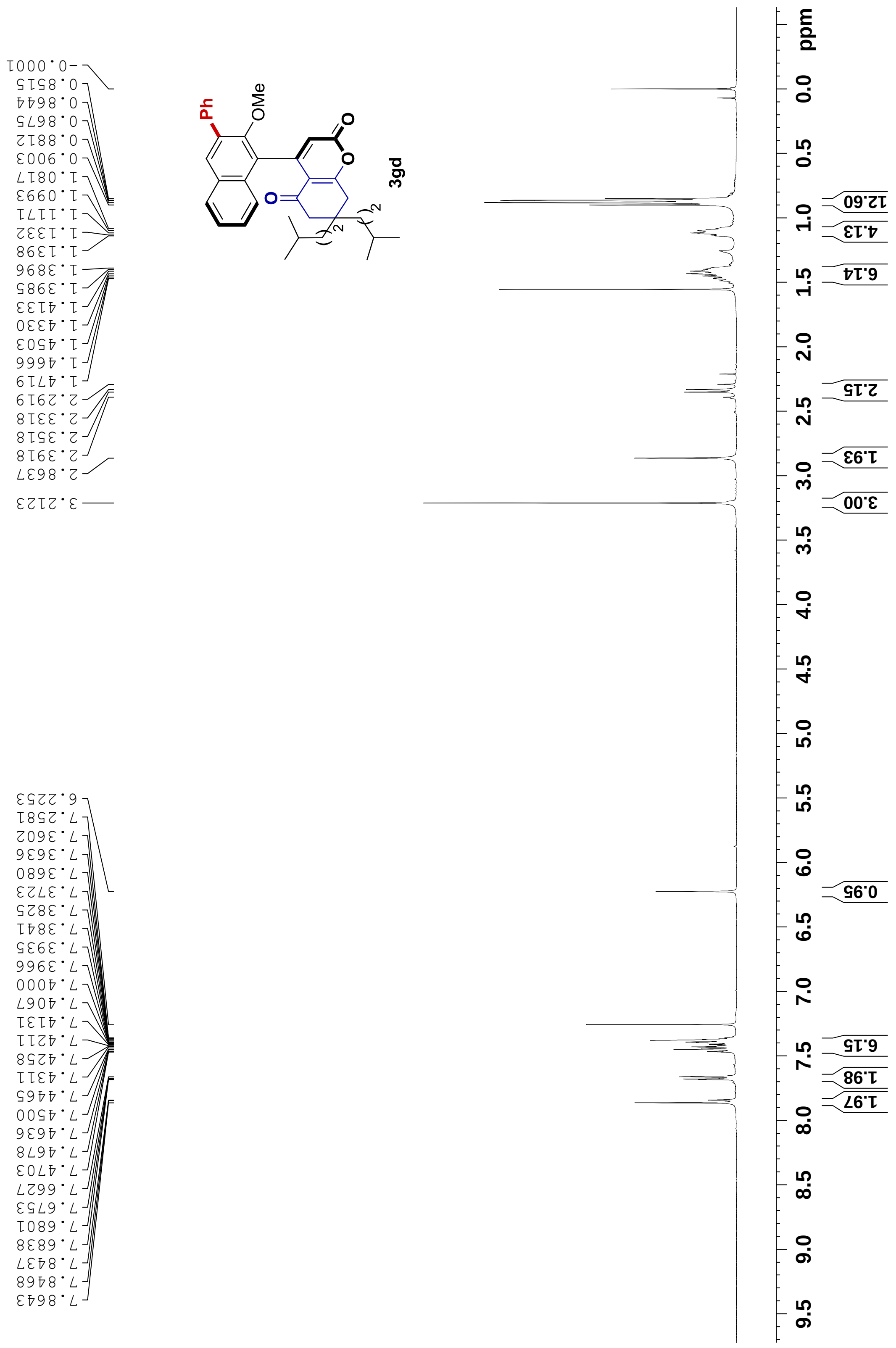

Supplementary Figure 39. ^{13}C NMR Spectrum of 3gd

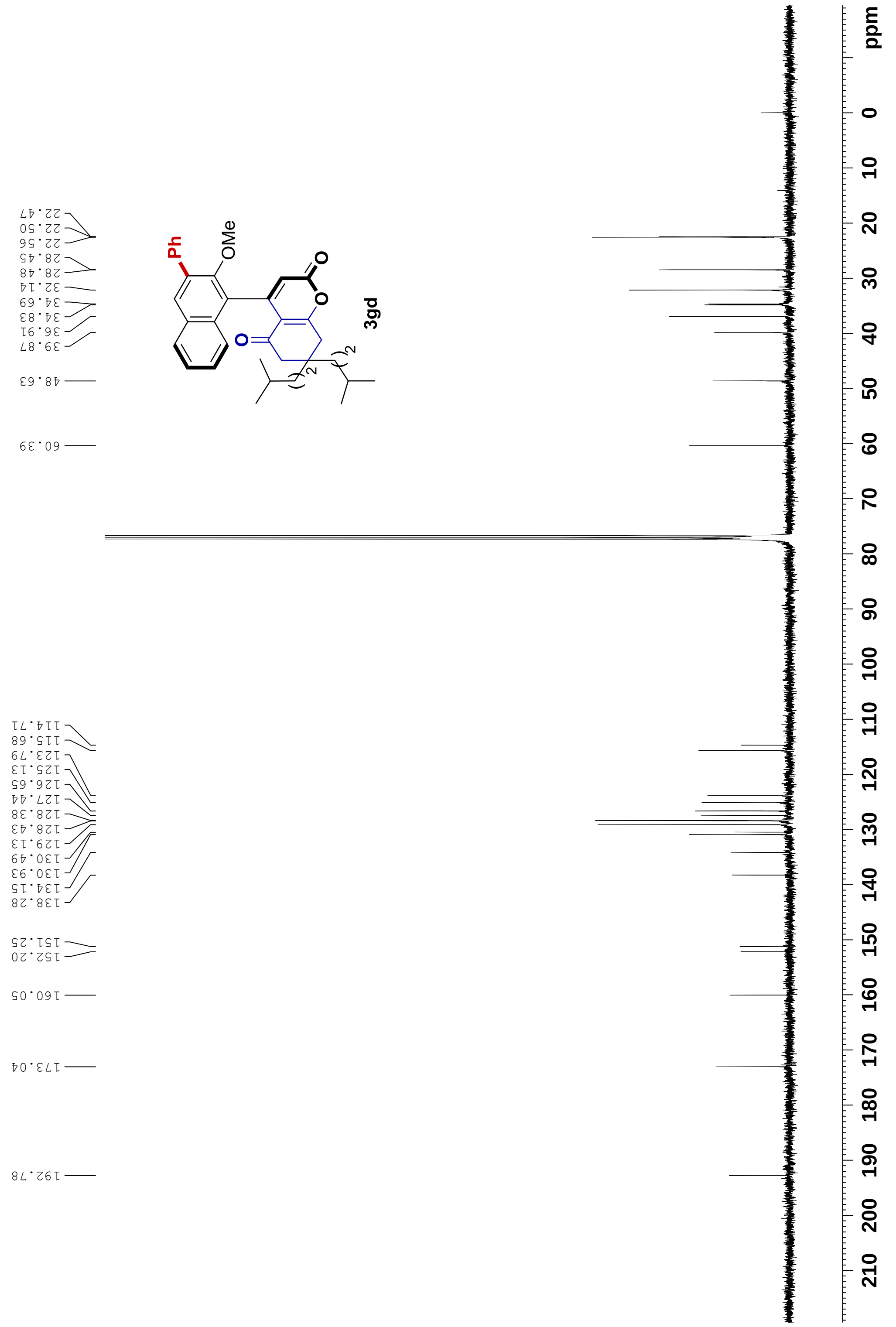

Supplementary Figure 40. HPLC Spectrum of racemic 3gd

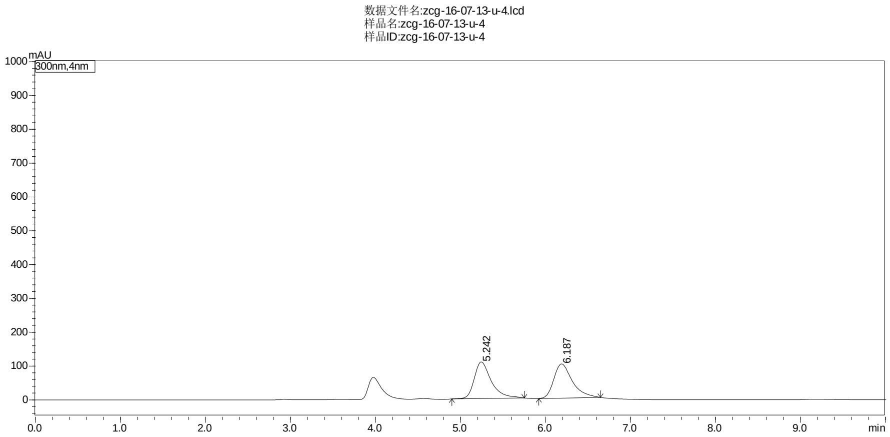

Peak#	Ret. Time	Area	Height	Area%	Height%
1	5.242	1509264	108000	49.565	51.638
2	6.187	1535751	101147	50.435	48.362
Total		3045016	209147	100.000	100.000

Supplementary Figure 41. HPLC Spectrum of 3gd

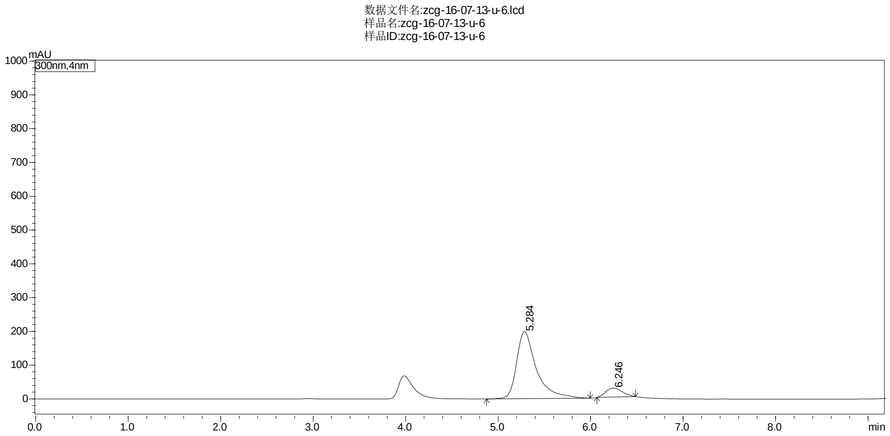

Peak#	Ret. Time	Area	Height	Area%	Height%
1	5.284	2945838	198470	89.871	88.161
2	6.246	332027	26652	10.129	11.839
Total		3277865	225123	100.000	100.000

Supplementary Figure 42. ^{1}H NMR Spectrum of 3ge

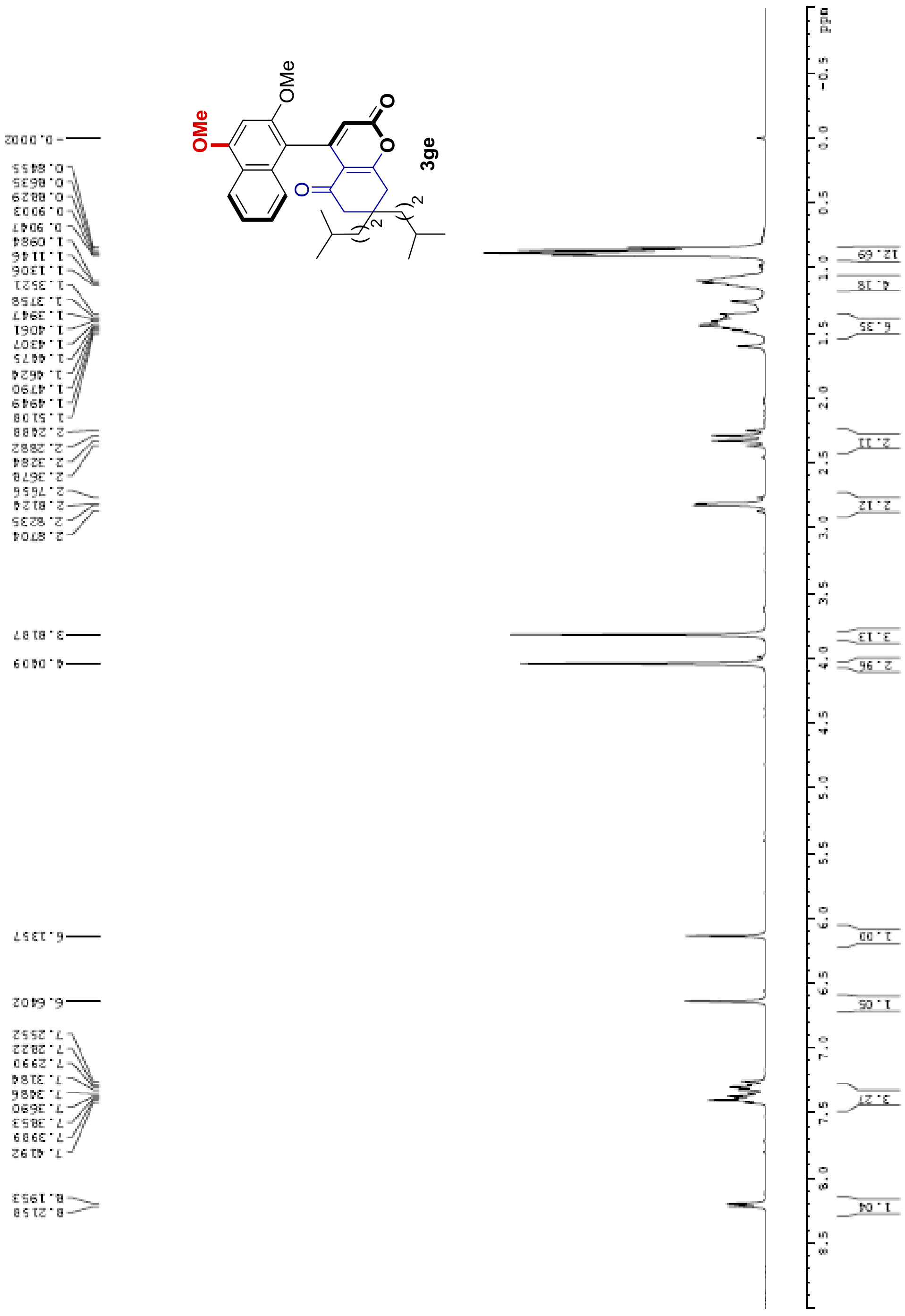

Supplementary Figure 43. ^{13}C NMR Spectrum of 3ge

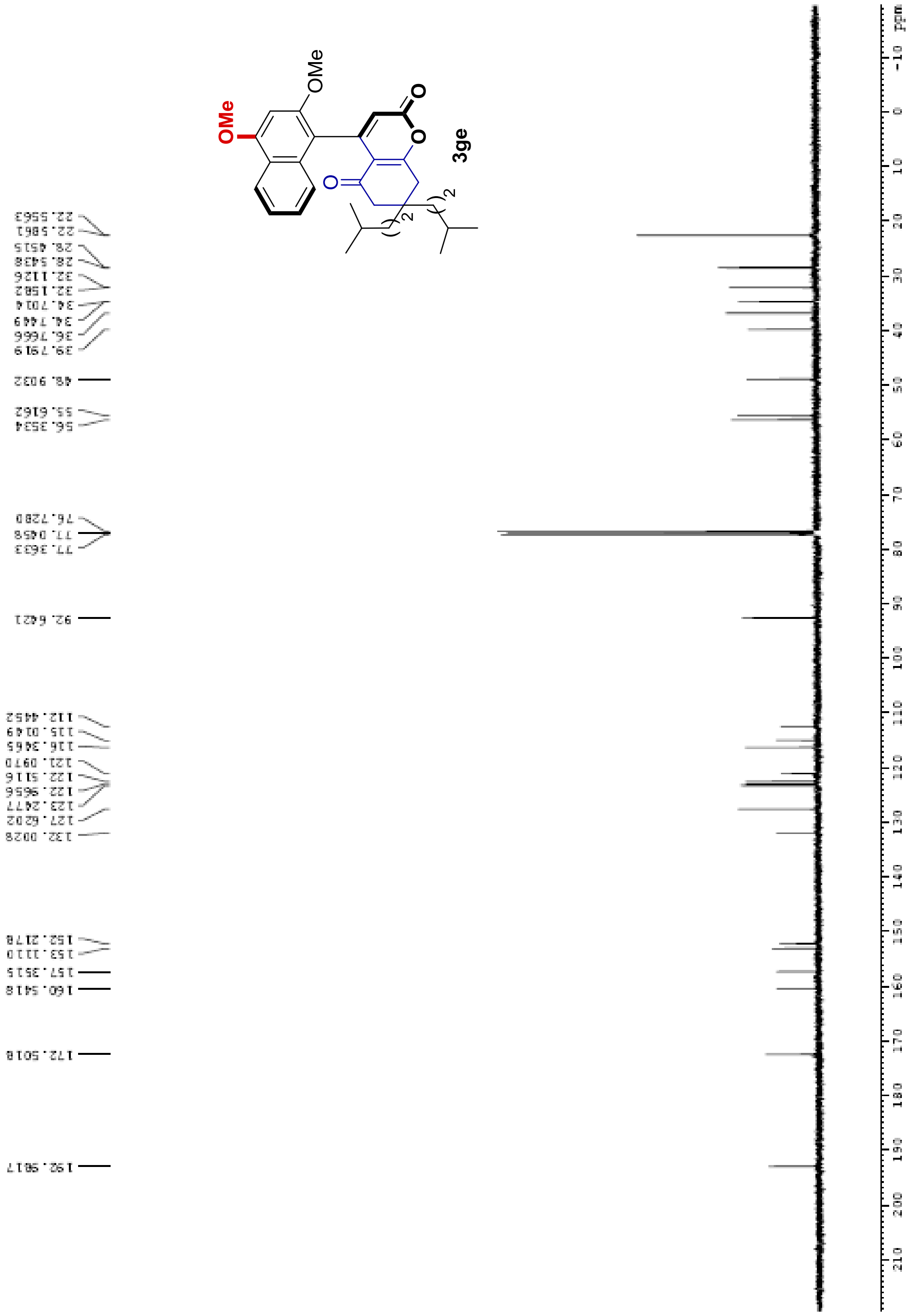

Supplementary Figure 44. ^{13}C NMR Spectrum of 3ge

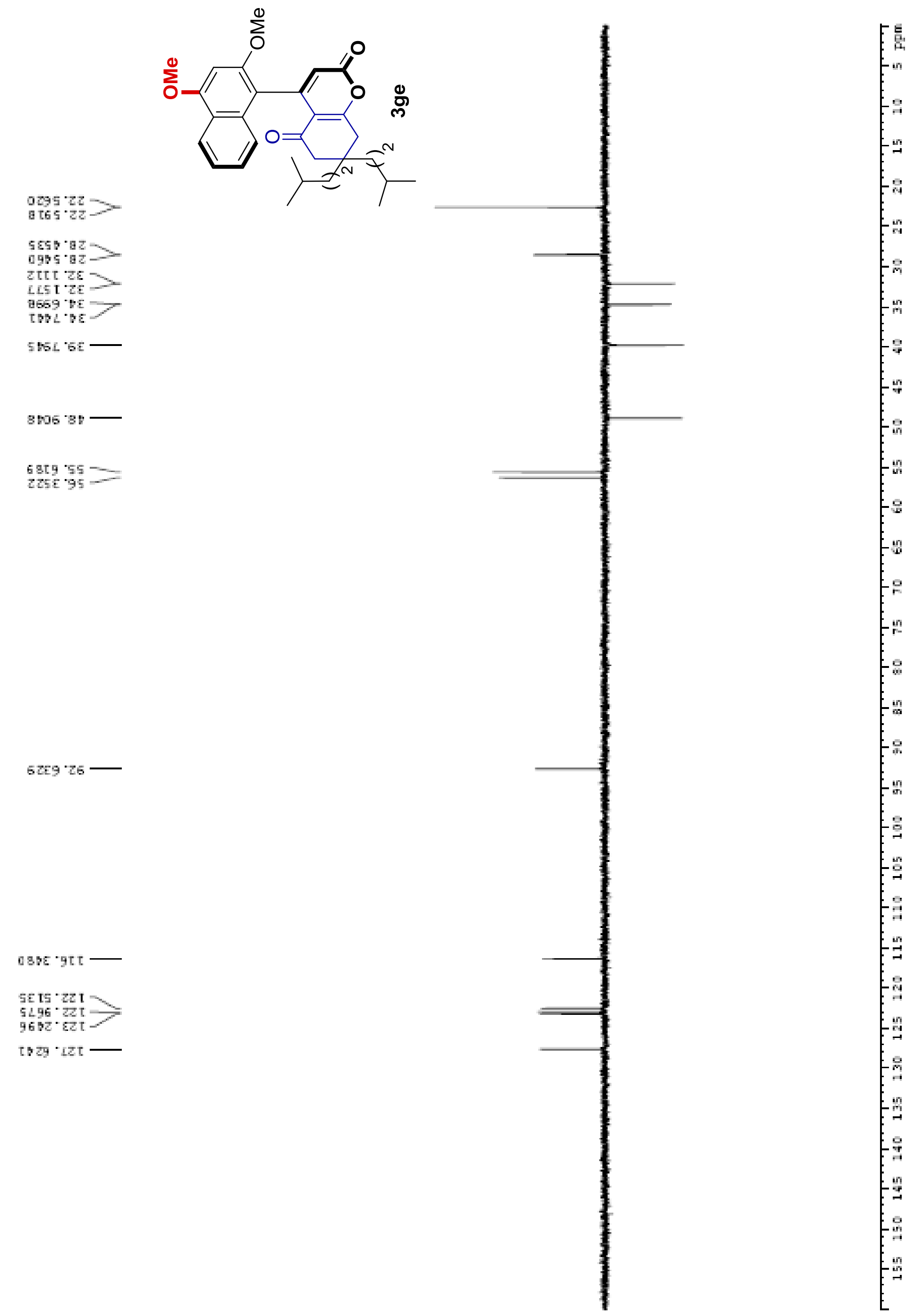

Supplementary Figure 45. HPLC Spectrum of racemic 3ge

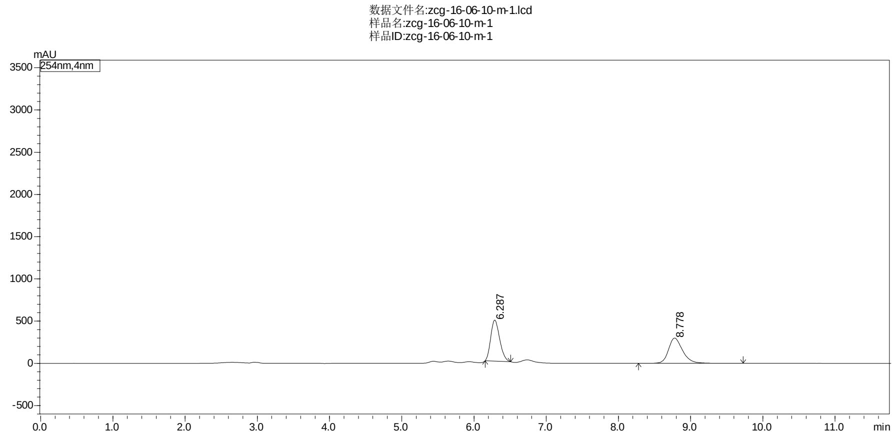

Peak#	Ret. Time	Area	Height	Area%	Height%
1	6.287	4205010	486577	50.790	61.901
2	8.778	4074138	299485	49.210	38.099
Total		8279148	786062	100.000	100.000

Supplementary Figure 46. HPLC Spectrum of 3ge

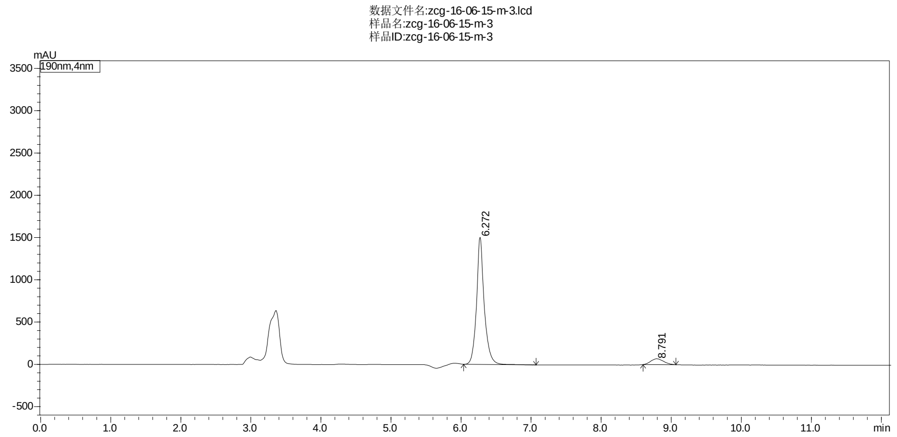

Peak#	Ret. Time	Area	Height	Area%	Height%
1	6.272	11518515	1504360	92.945	95.619
2	8.791	874269	68921	7.055	4.381
Total		12392783	1573281	100.000	100.000

Supplementary Figure 47. ^{1}H NMR Spectrum of 3gf

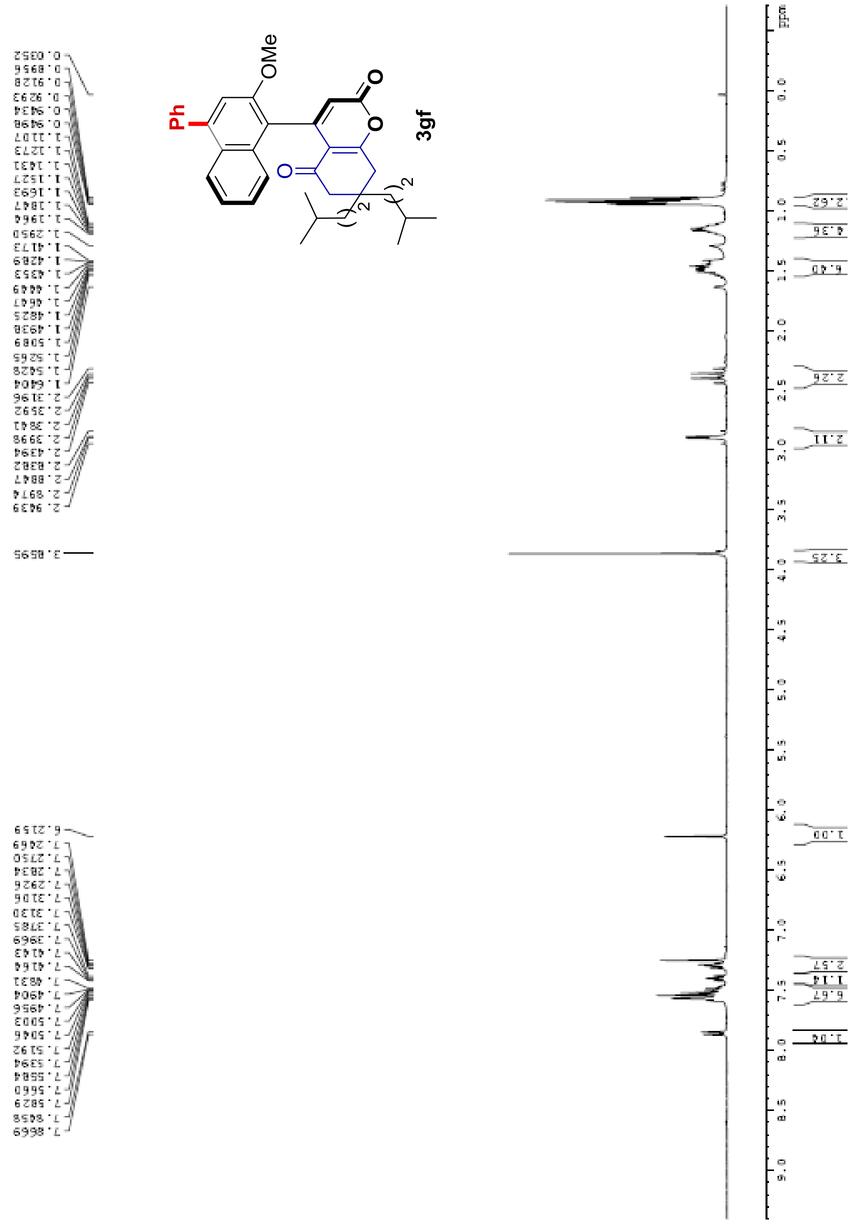

Supplementary Figure 48. ^{13}C NMR Spectrum of 3gf

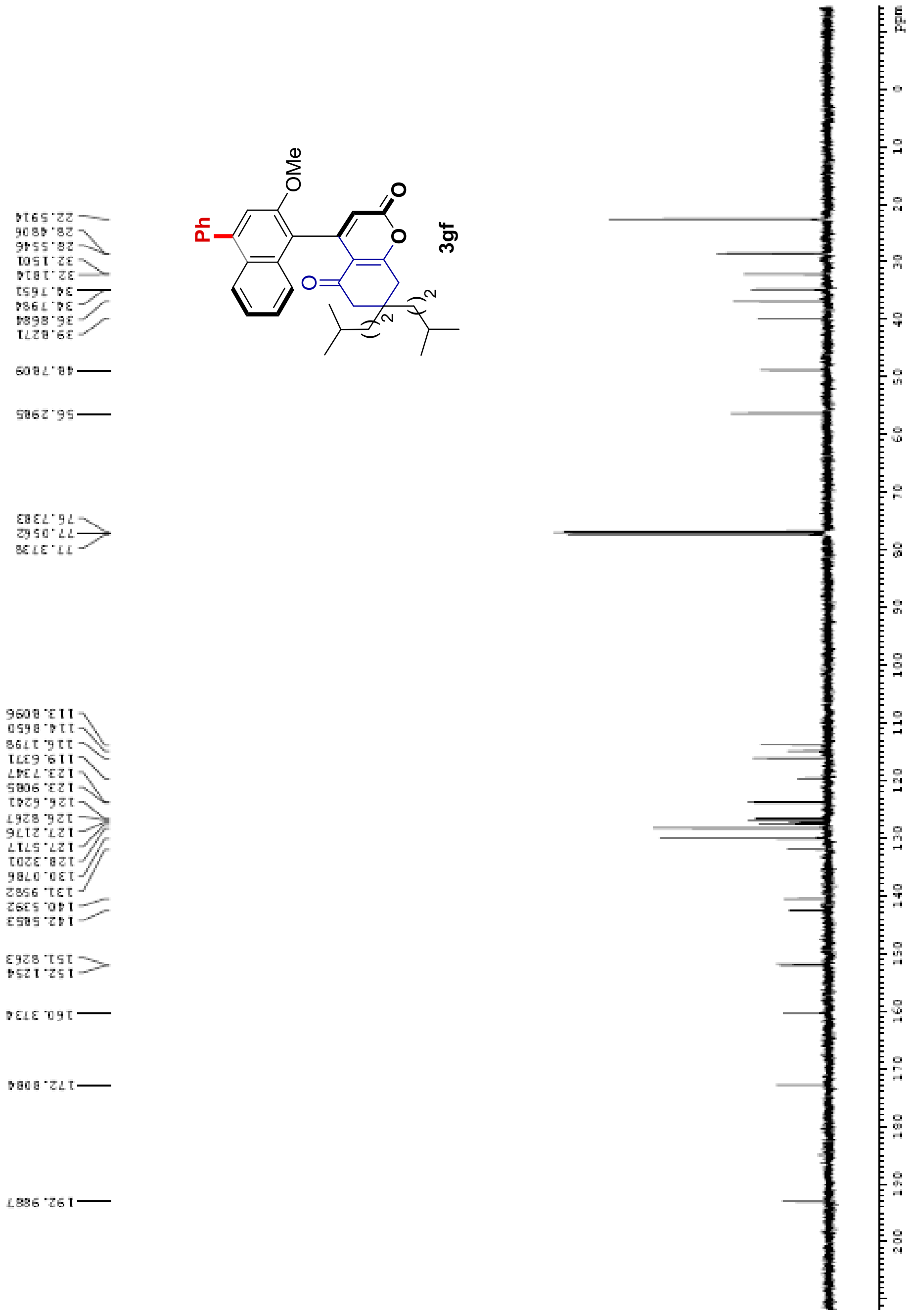

Supplementary Figure 49. ^{13}C NMR Spectrum of 3gf

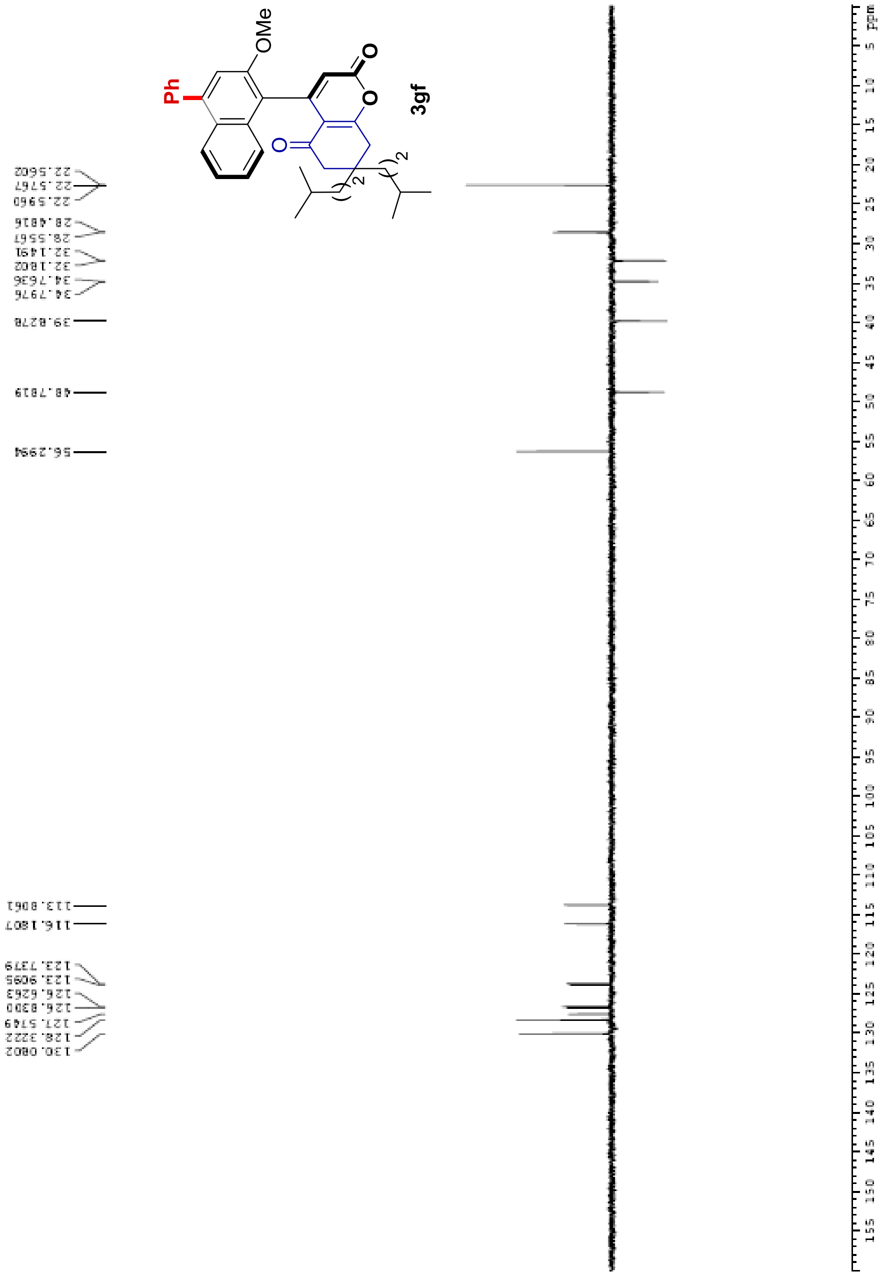

Supplementary Figure 50. HPLC Spectrum of racemic 3gf

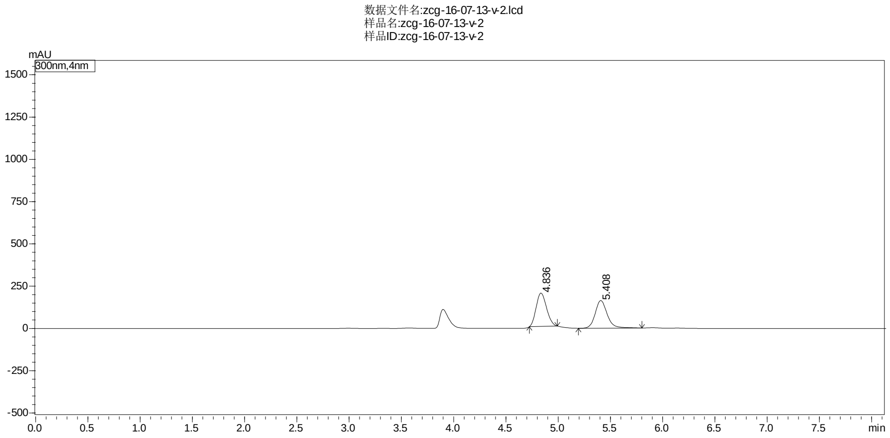

Peak#	Ret. Time	Area	Height	Area%	Height%
1	4.836	1365001	197201	50.914	54.517
2	5.408	1316015	164522	49.086	45.483
Total		2681015	361724	100.000	100.000

Supplementary Figure 51. HPLC Spectrum of 3gf

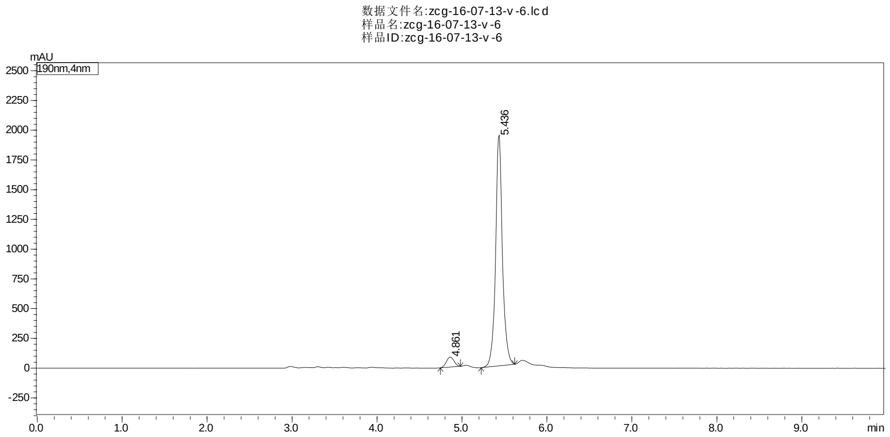

Peak#	Ret. Time	Area	Height	Area%	Height%
1	4.861	526963	83904	4.630	4.144
2	5.436	10855714	1940907	95.370	95.856
Total		11382677	2024811	100.000	100.000

Supplementary Figure 52. ^{1}H NMR Spectrum of 3gg

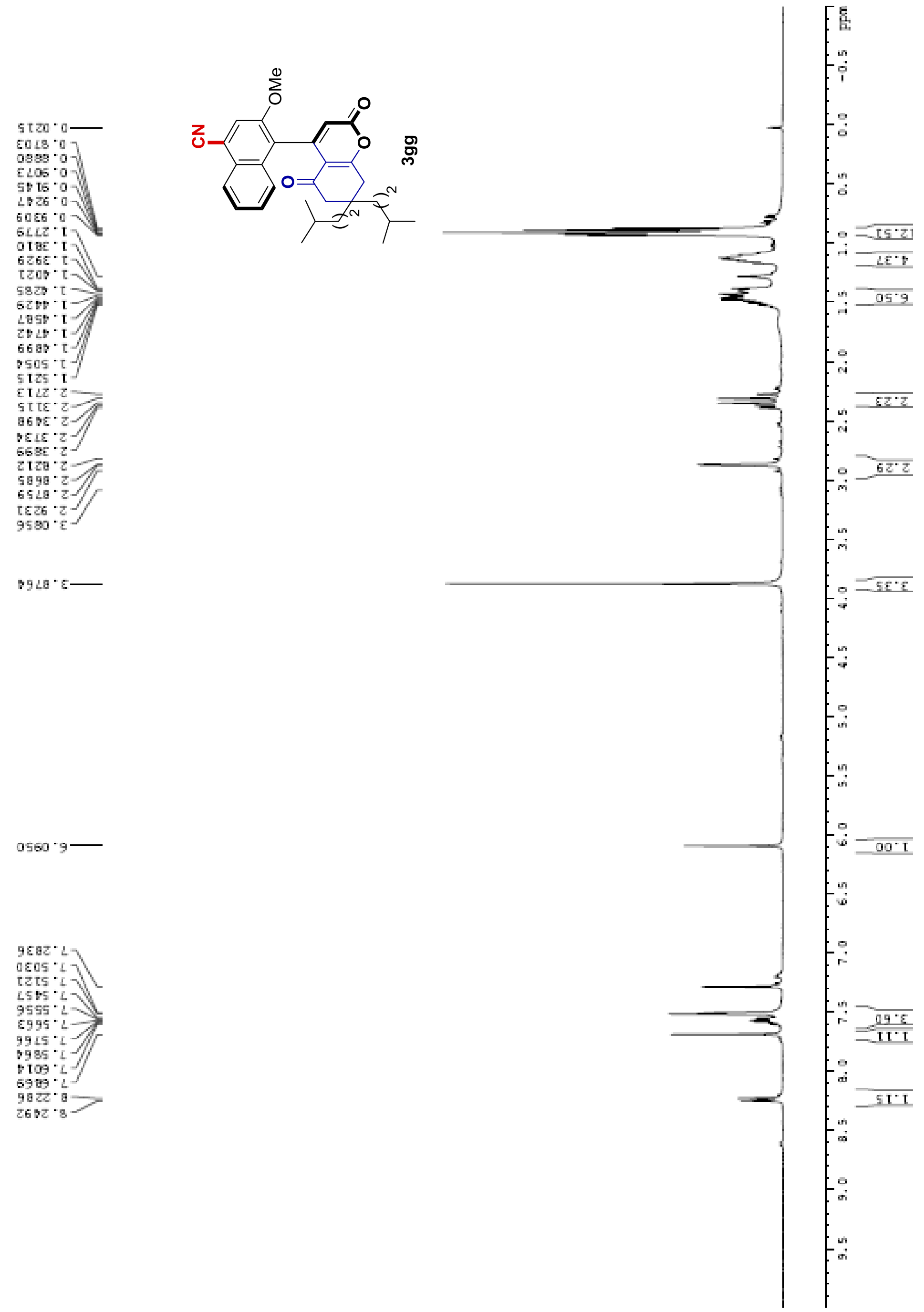

Supplementary Figure 53. ^{13}C NMR Spectrum of 3gg

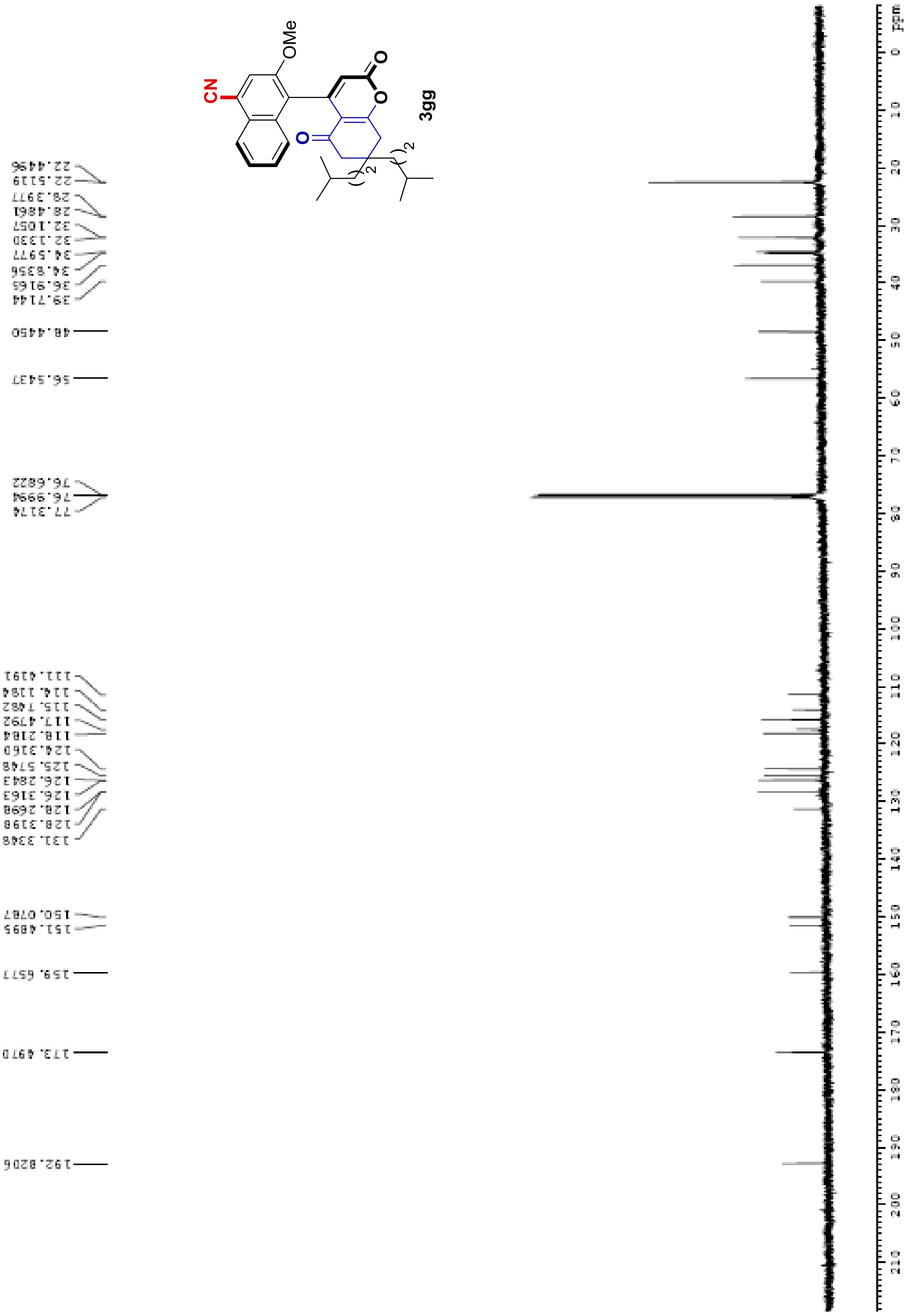

Supplementary Figure 54. HPLC Spectrum of racemic 3gg

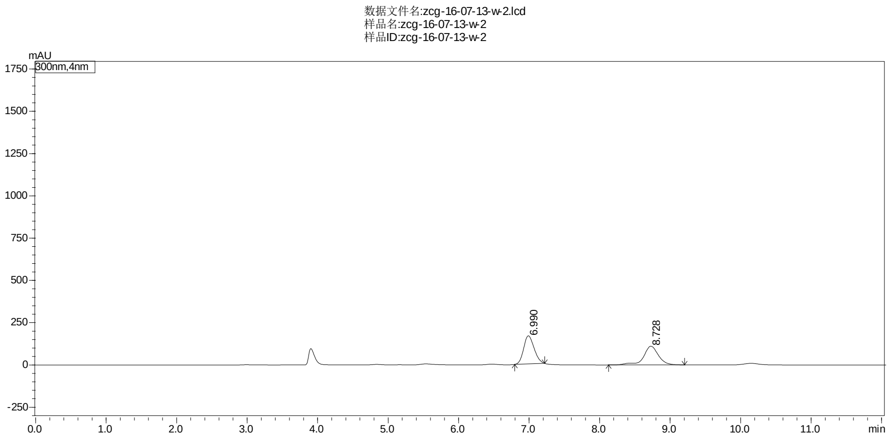

Peak#	Ret. Time	Area	Height	Area%	Height%
1	6.990	1721891	166243	51.421	60.063
2	8.728	1626730	110539	48.579	39.937
Total		3348621	276782	100.000	100.000

Supplementary Figure 55. HPLC Spectrum of 3gg

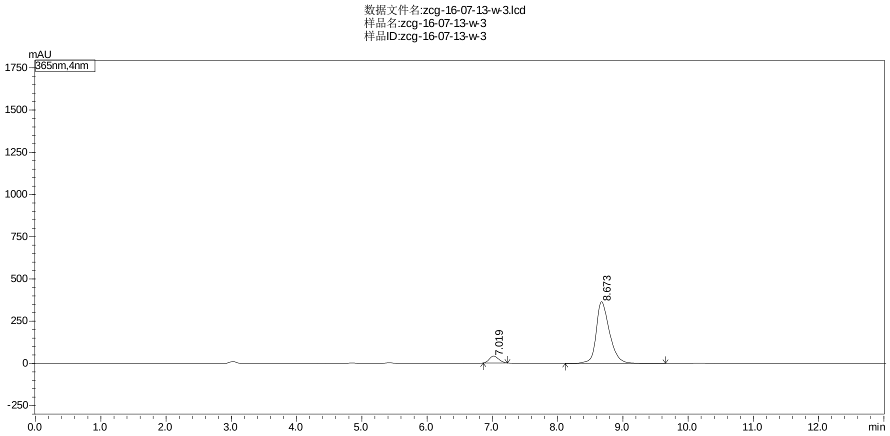

Peak#	Ret. Time	Area	Height	Area%	Height%
1	7.019	404578	40739	7.252	10.017
2	8.673	5174470	365981	92.748	89.983
Total		5579048	406720	100.000	100.000

Supplementary Figure 56. ^{1}H NMR Spectrum of 3gh

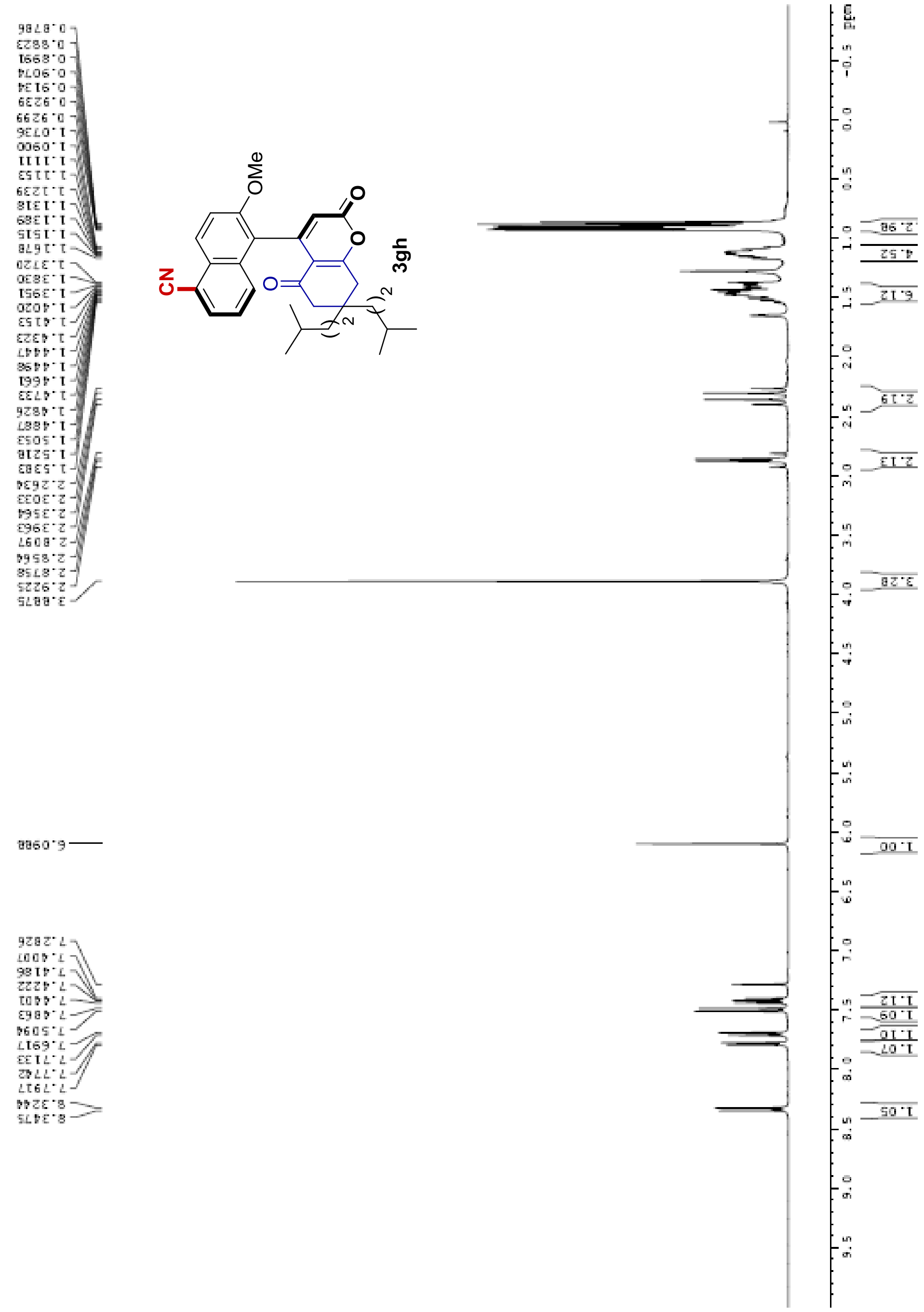

Supplementary Figure 57. ^{13}C NMR Spectrum of 3gh

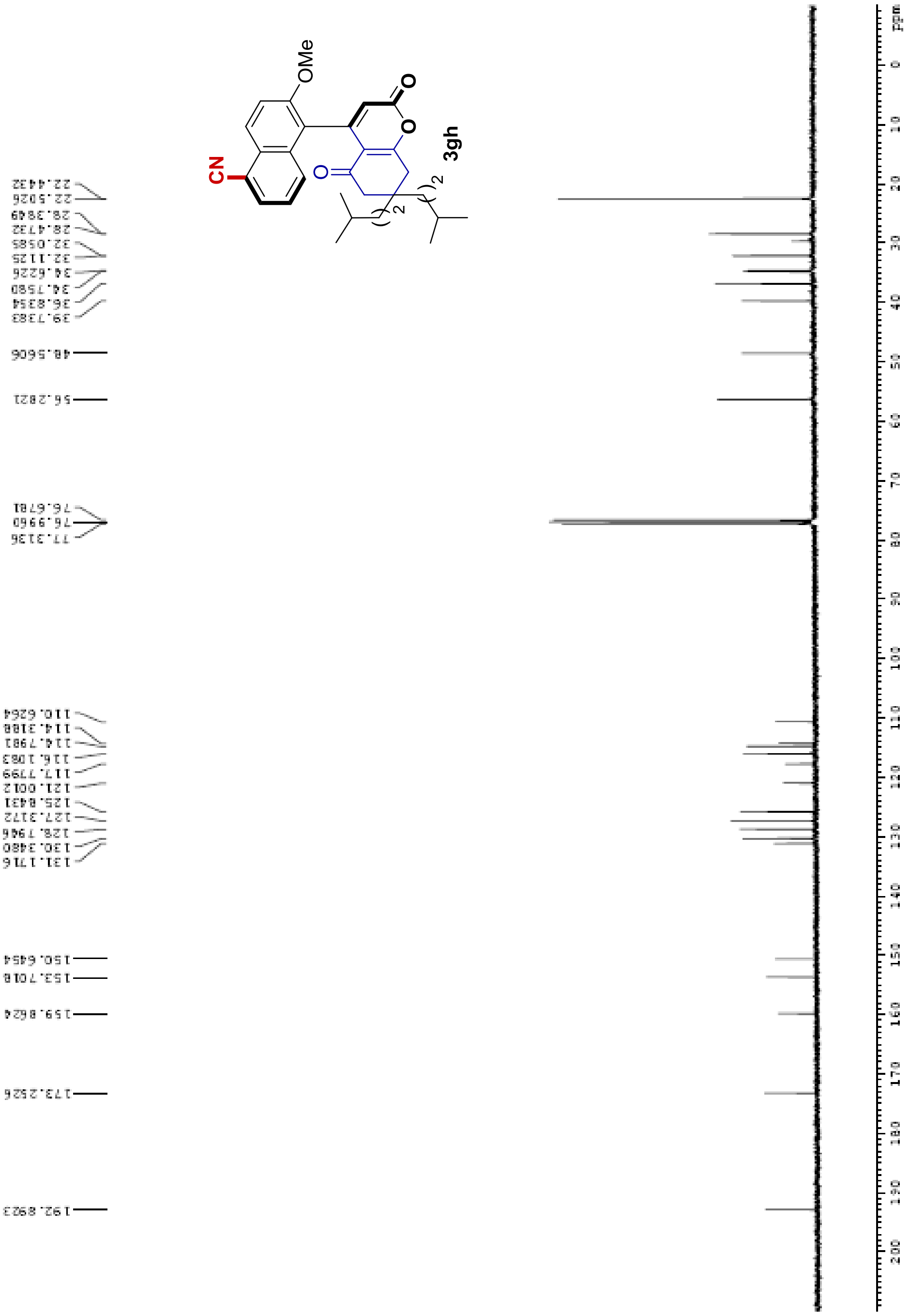

Supplementary Figure 58. ^{13}C NMR Spectrum of 3gh

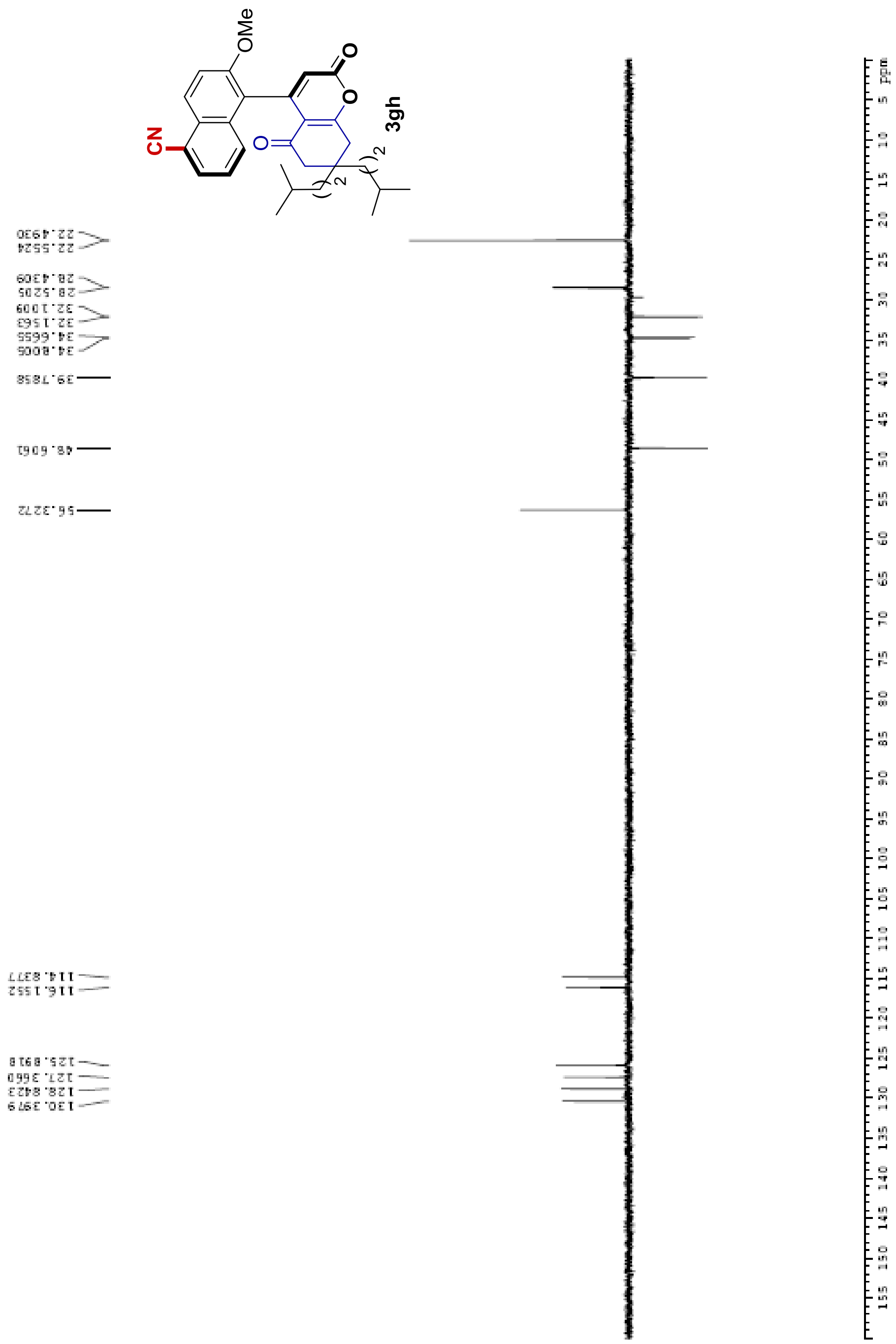

Supplementary Figure 59. HPLC Spectrum of racemic 3gh

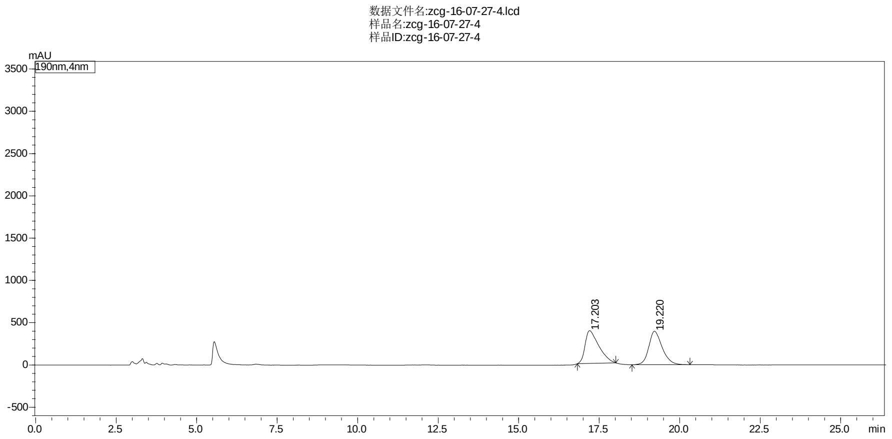

Peak#	Ret. Time	Area	Height	Area%	Height%
1	17.203	11487058	387720	49.937	49.429
2	19.220	11516237	396686	50.063	50.571
Total		23003294	784405	100.000	100.000

Supplementary Figure 60. HPLC Spectrum of 3gh

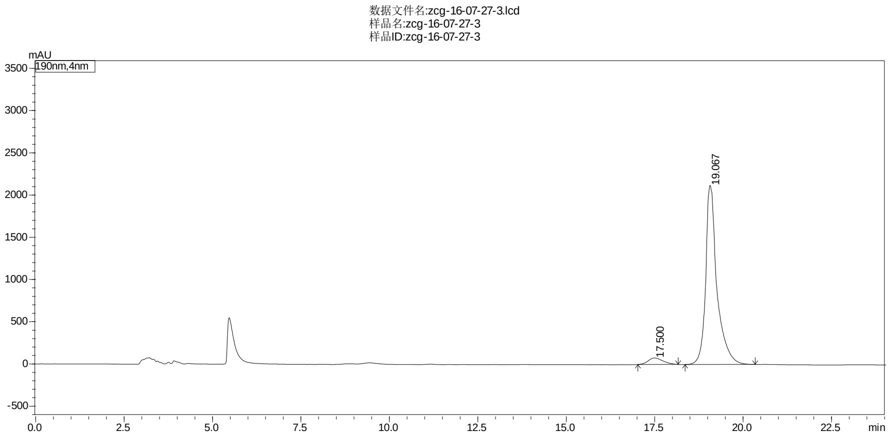

Peak#	Ret. Time	Area	Height	Area%	Height%
1	17.500	2227939	76837	4.531	3.497
2	19.067	46946120	2120381	95.469	96.503
Total		49174059	2197218	100.000	100.000

Supplementary Figure 61. ^{1}H NMR Spectrum of 3gi

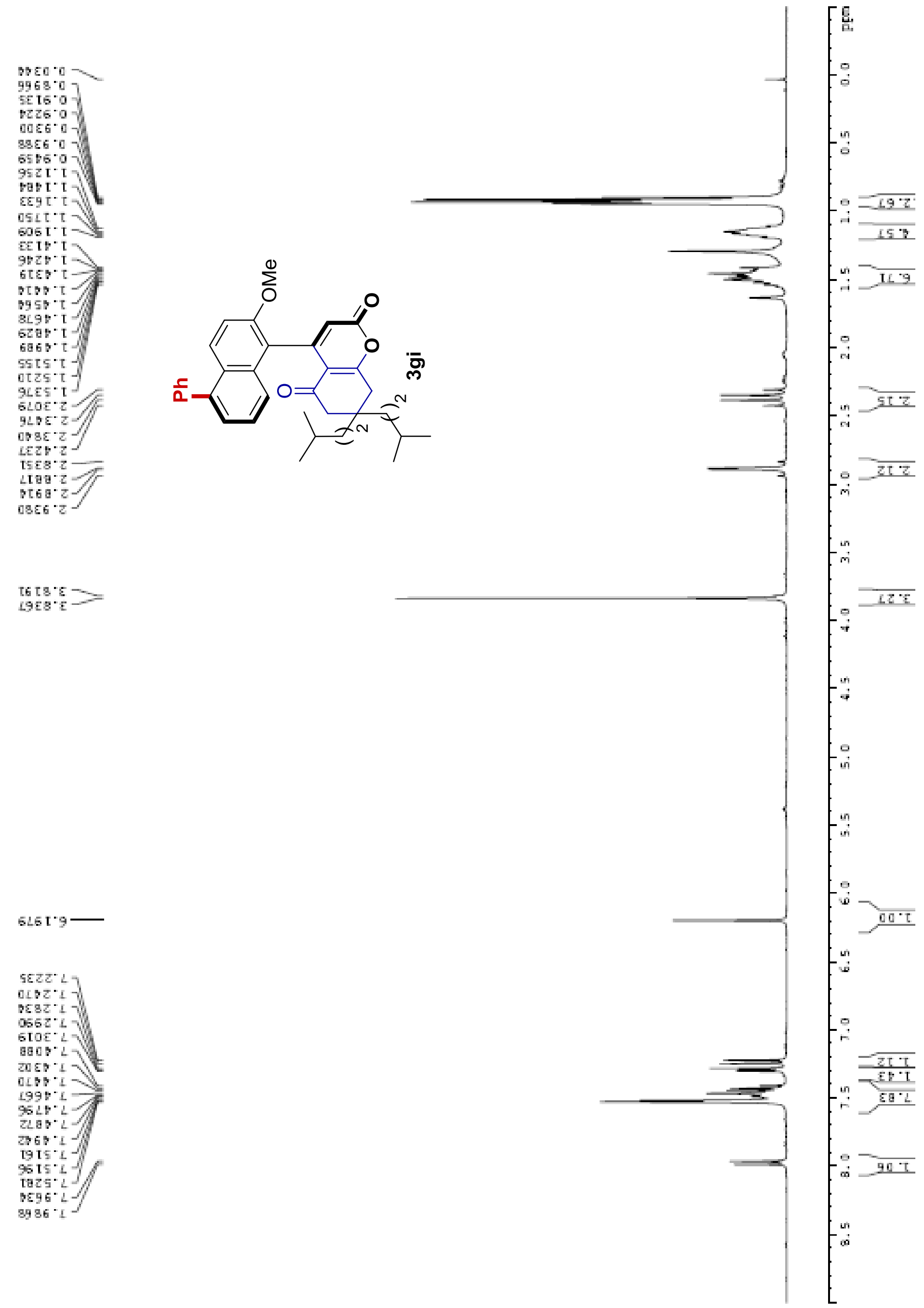

Supplementary Figure 62. ^{13}C NMR Spectrum of 3gi

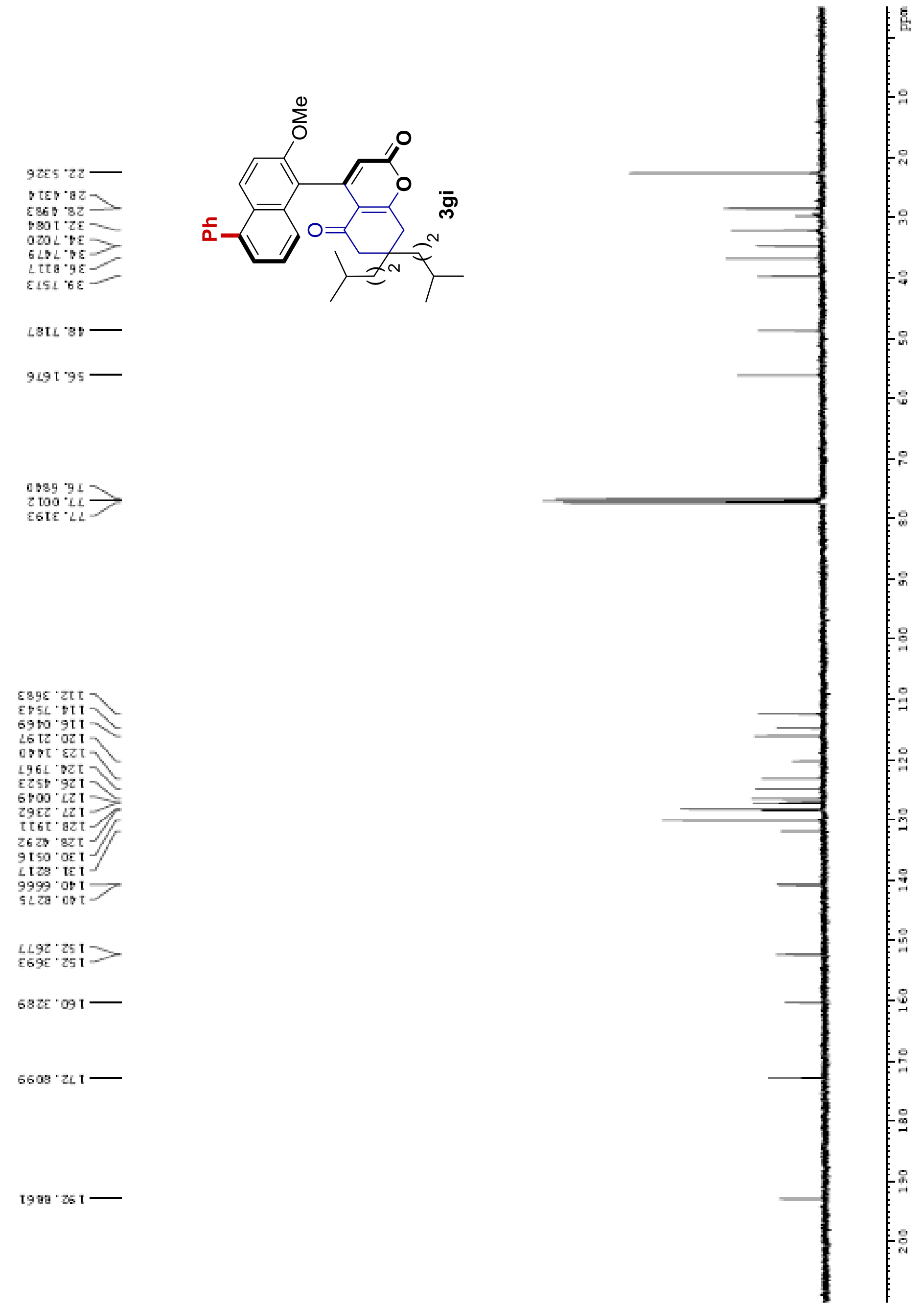

Supplementary Figure 63. HPLC Spectrum of racemic 3gi

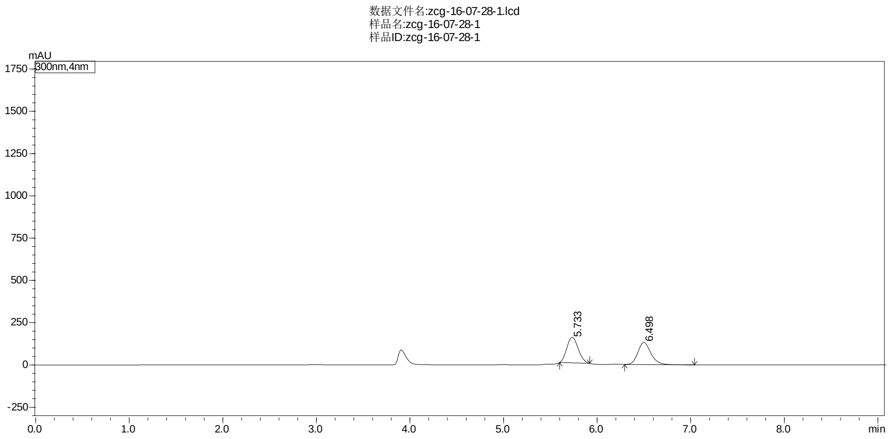

Peak#	Ret. Time	Area	Height	Area%	Height%
1	5.733	1280854	150715	50.291	53.419
2	6.498	1266028	131423	49.709	46.581
Total		2546881	282138	100.000	100.000

Supplementary Figure 64. HPLC Spectrum of 3gi

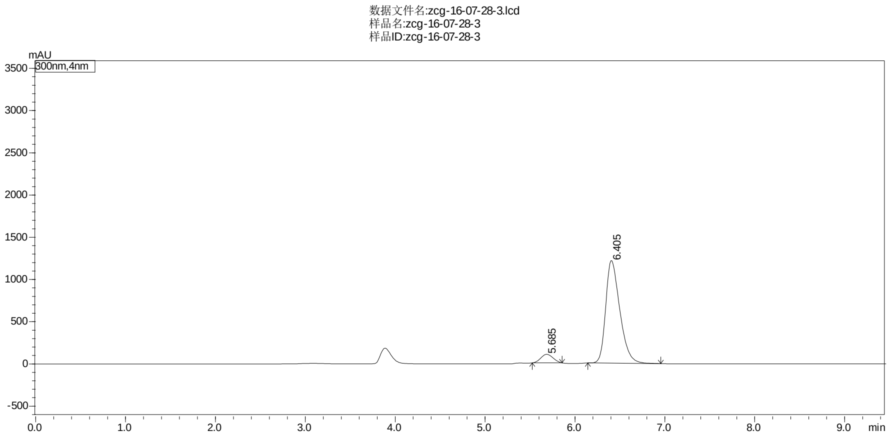

Peak#	Ret. Time	Area	Height	Area%	Height%
1	5.685	952256	101183	6.705	7.695
2	6.405	13248874	1213725	93.295	92.305
Total		14201129	1314908	100.000	100.000

Supplementary Figure 65. ^{1}H NMR Spectrum of 3gj

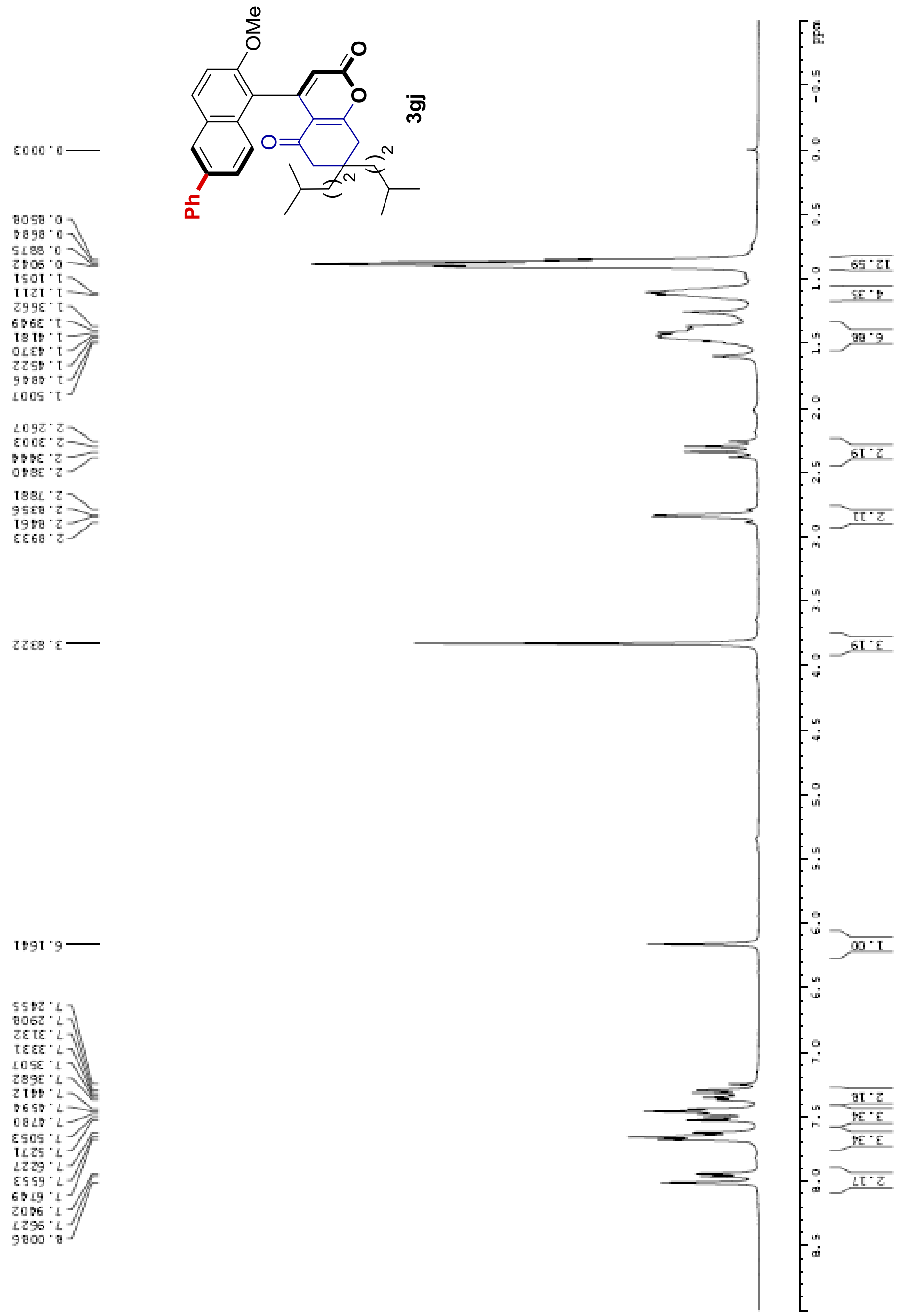

Supplementary Figure 66. ^{13}C NMR Spectrum of 3gj

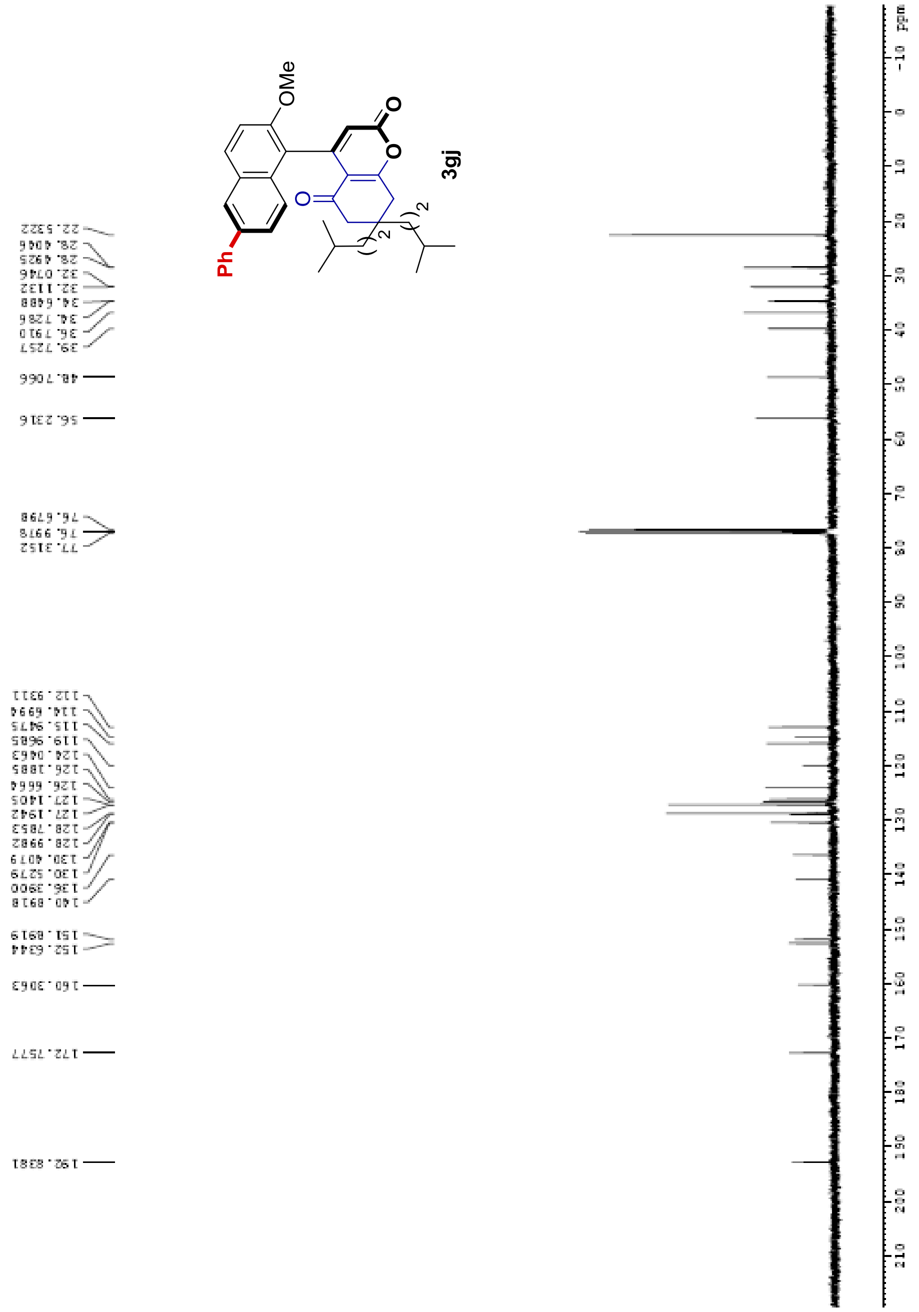

Supplementary Figure 67. HPLC Spectrum of racemic 3gj

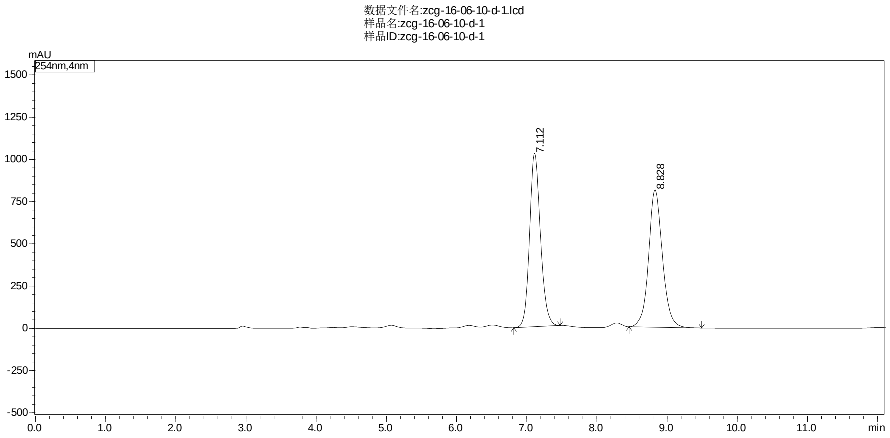

Peak#	Ret. Time	Area	Height	Area%	Height%
1	7.112	11063381	1027867	49.839	55.831
2	8.828	11134856	813166	50.161	44.169
Total		22198236	1841034	100.000	100.000

Supplementary Figure 68. HPLC Spectrum of 3gj

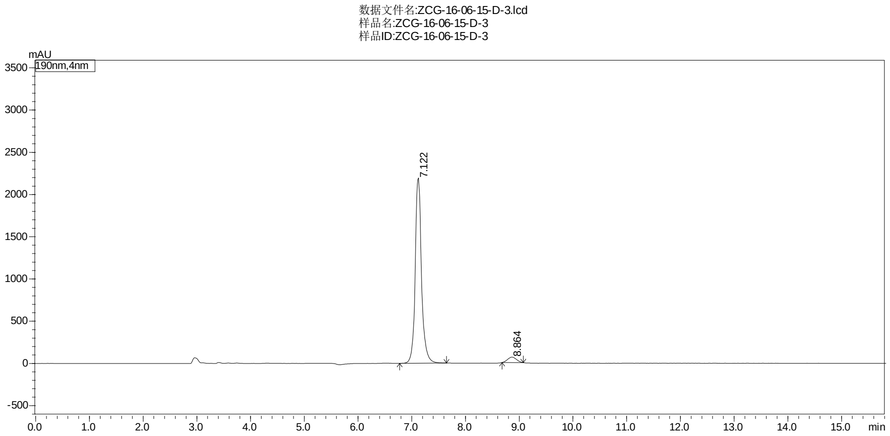

Peak#	Ret. Time	Area	Height	Area%	Height%
1	7.122	17978023	2195255	96.026	97.146
2	8.864	744049	64485	3.974	2.854
Total		18722072	2259740	100.000	100.000

Supplementary Figure 69. ^{1}H NMR Spectrum of 3gk

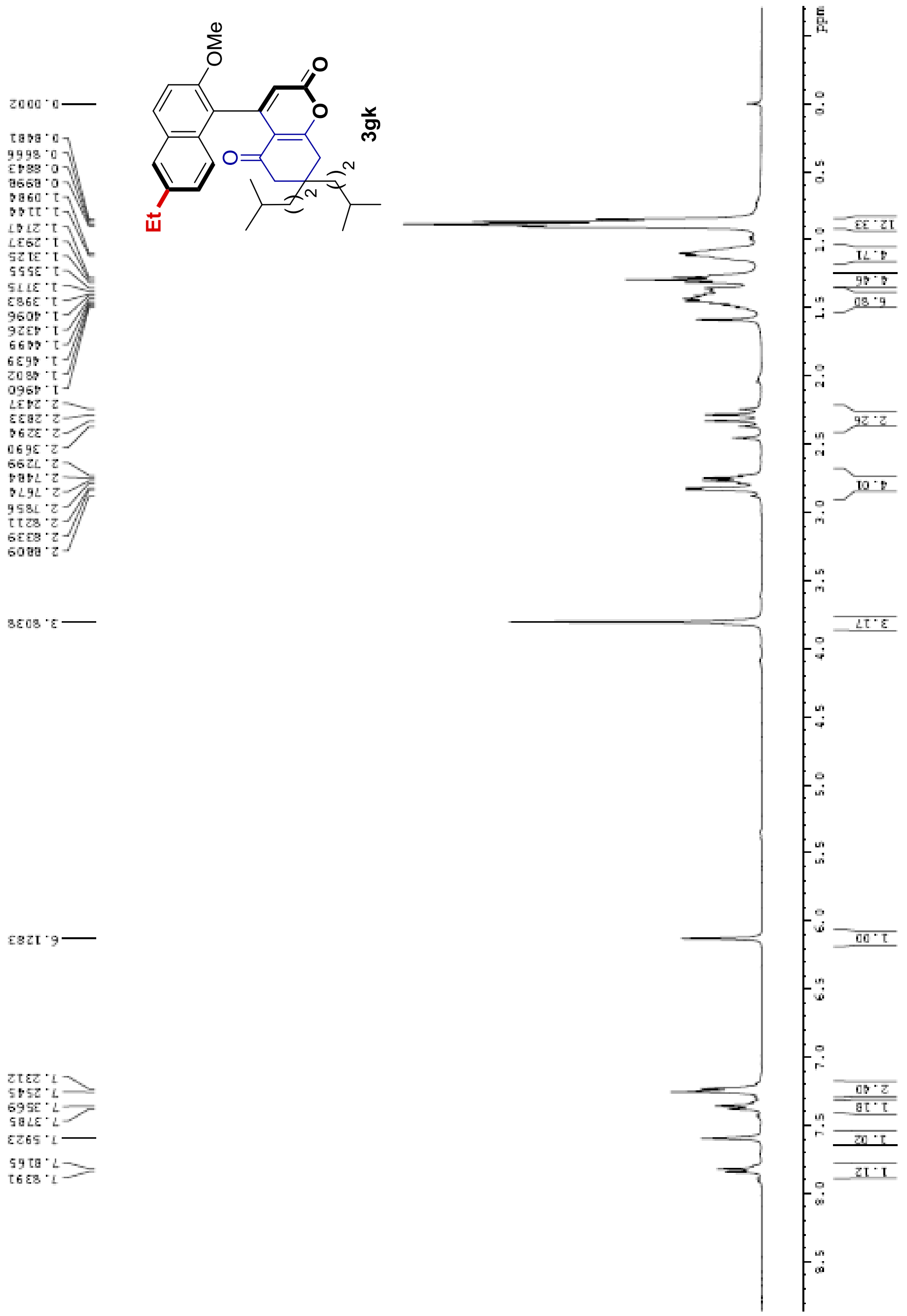

Supplementary Figure 70. ^{13}C NMR Spectrum of 3gk

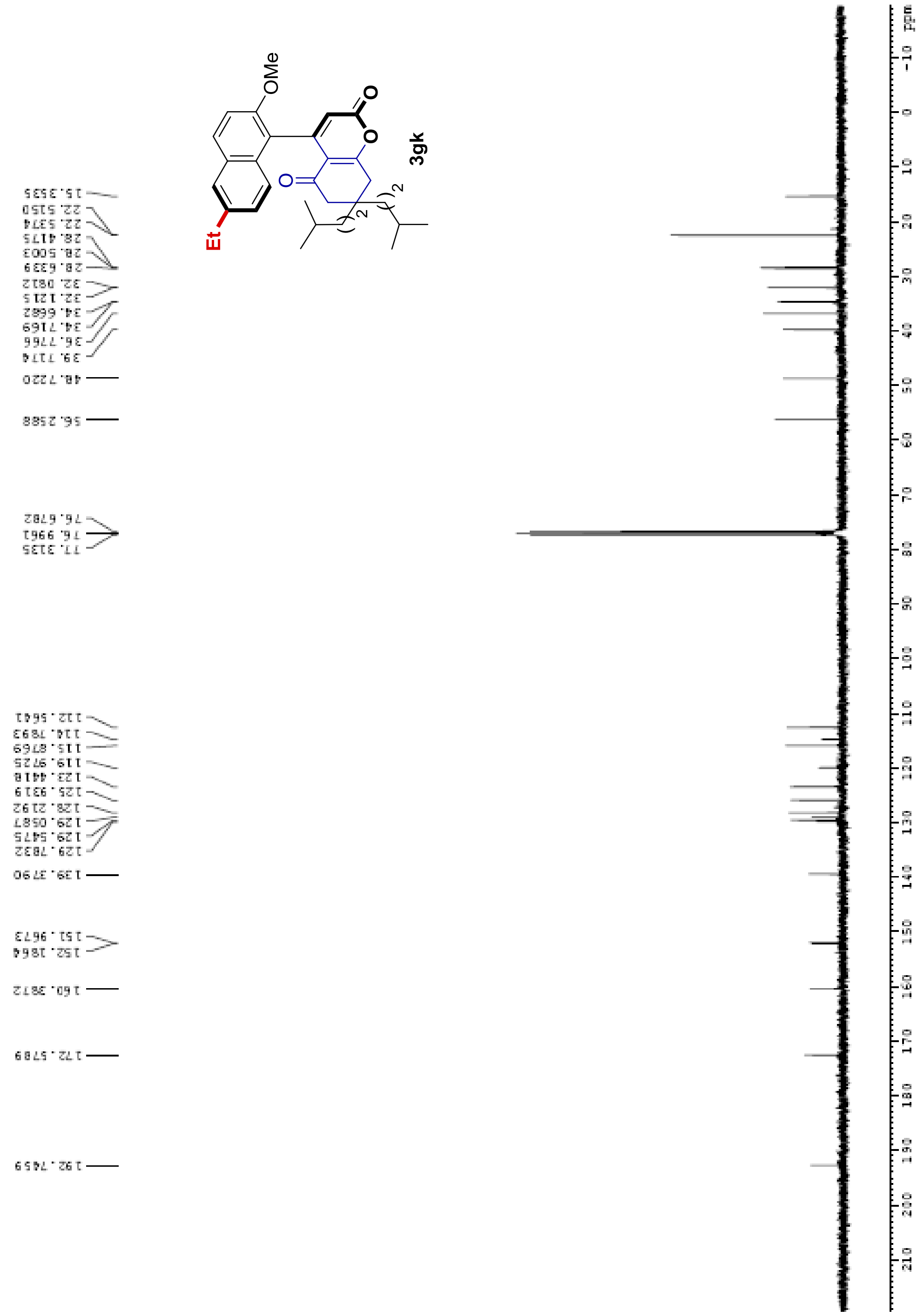

Supplementary Figure 71. HPLC Spectrum of racemic 3gk

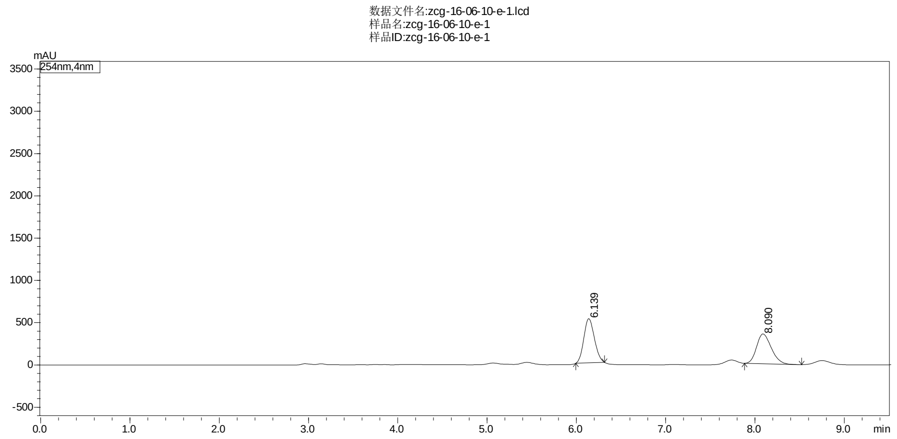

Peak#	Ret. Time	Area	Height	Area%	Height%
1	6.139	4285266	520570	51.561	59.647
2	8.090	4025802	352186	48.439	40.353
Total		8311068	872756	100.000	100.000

Supplementary Figure 72. HPLC Spectrum of 3gk

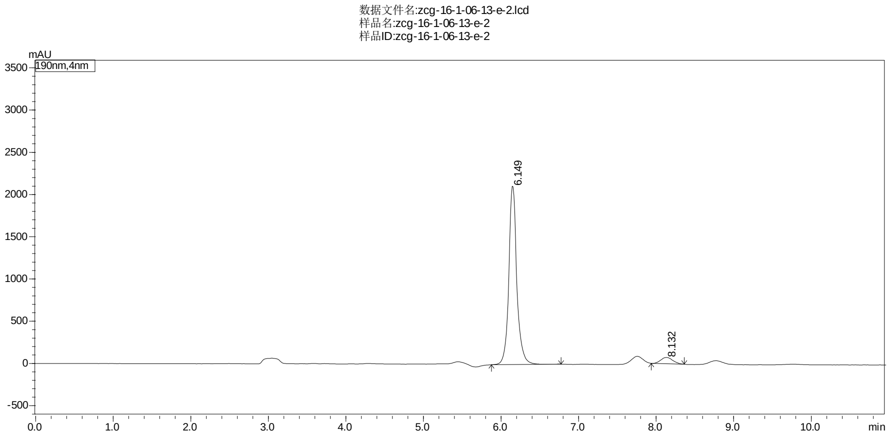

Peak#	Ret. Time	Area	Height	Area%	Height%
1	6.149	15098195	2112835	94.974	96.593
2	8.132	798923	74526	5.026	3.407
Total		15897118	2187361	100.000	100.000

Supplementary Figure 73. ^{1}H NMR Spectrum of 3gl

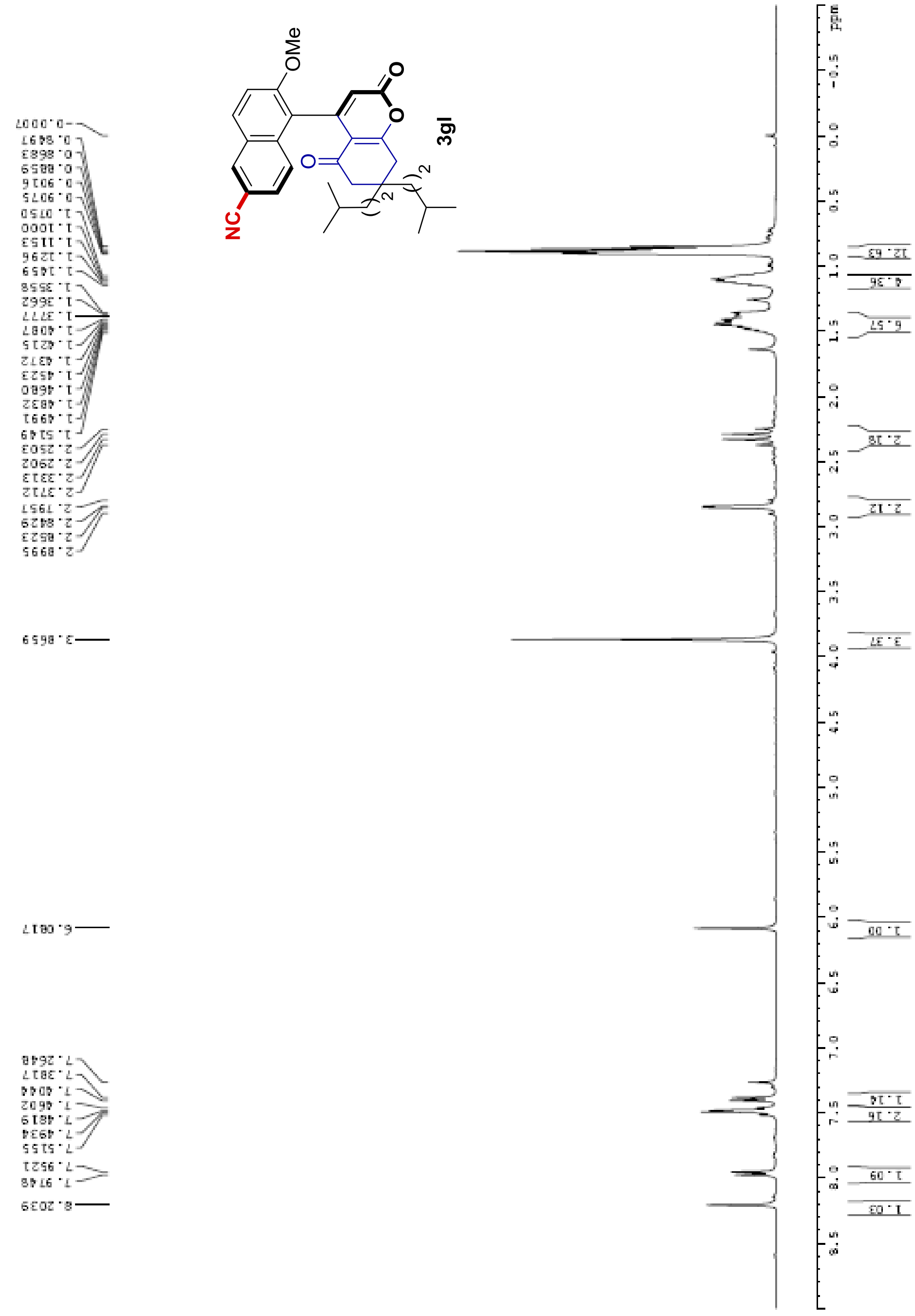

Supplementary Figure 74. ^{13}C NMR Spectrum of 3gl

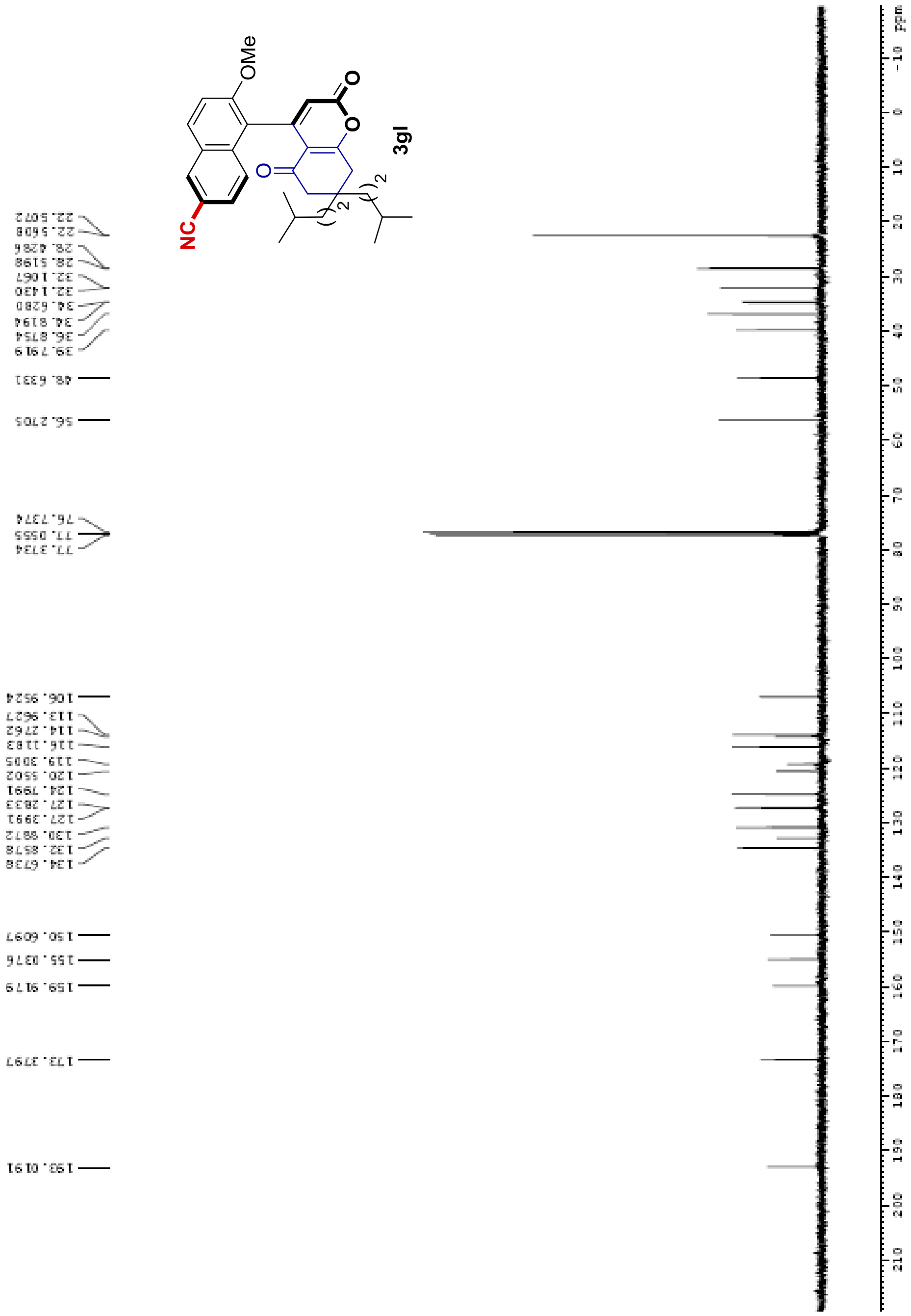

Supplementary Figure 75. HPLC Spectrum of racemic 3gl

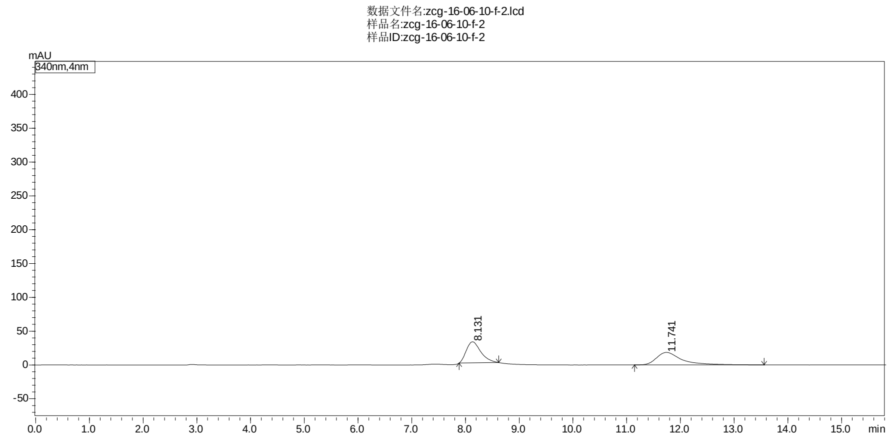

Peak#	Ret. Time	Area	Height	Area%	Height%
1	8.131	587408	31133	49.491	62.851
2	11.741	599478	18402	50.509	37.149
Total		1186886	49535	100.000	100.000

Supplementary Figure 76. HPLC Spectrum of 3gl

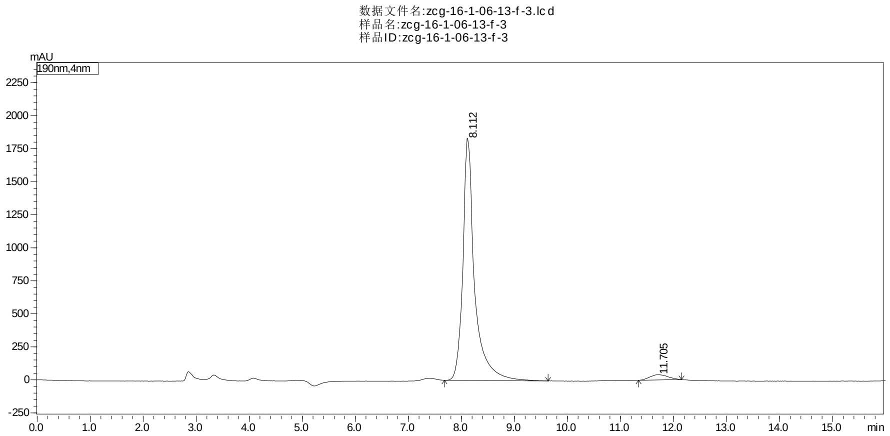

Peak#	Ret. Time	Area	Height	Area%	Height%
1	8.112	28314251	1831659	96.789	97.893
2	11.705	939237	39423	3.211	2.107
Total		29253488	1871082	100.000	100.000

Supplementary Figure 77. ^{1}H NMR Spectrum of 3gm

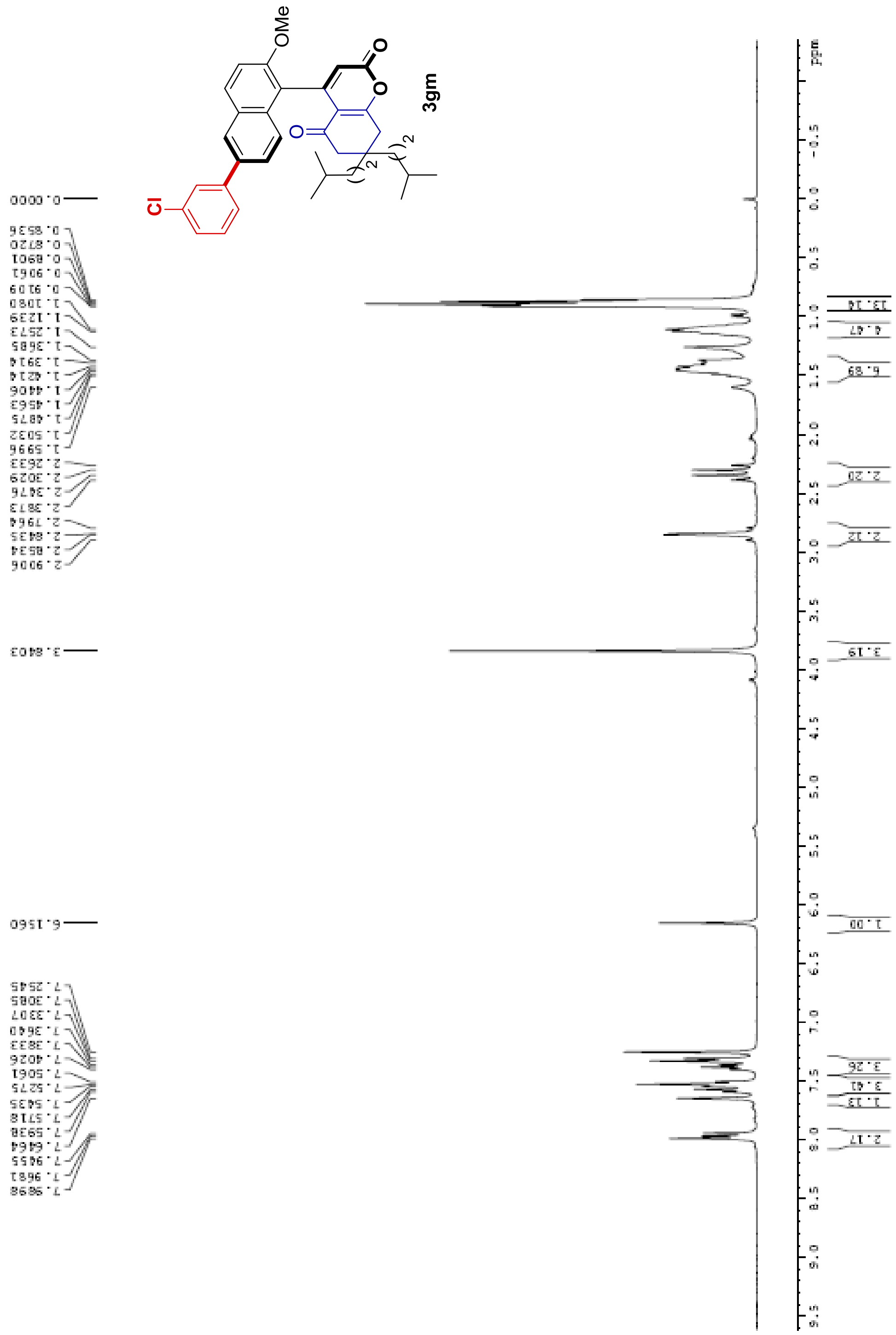

Supplementary Figure 78. ^{13}C NMR Spectrum of 3gm

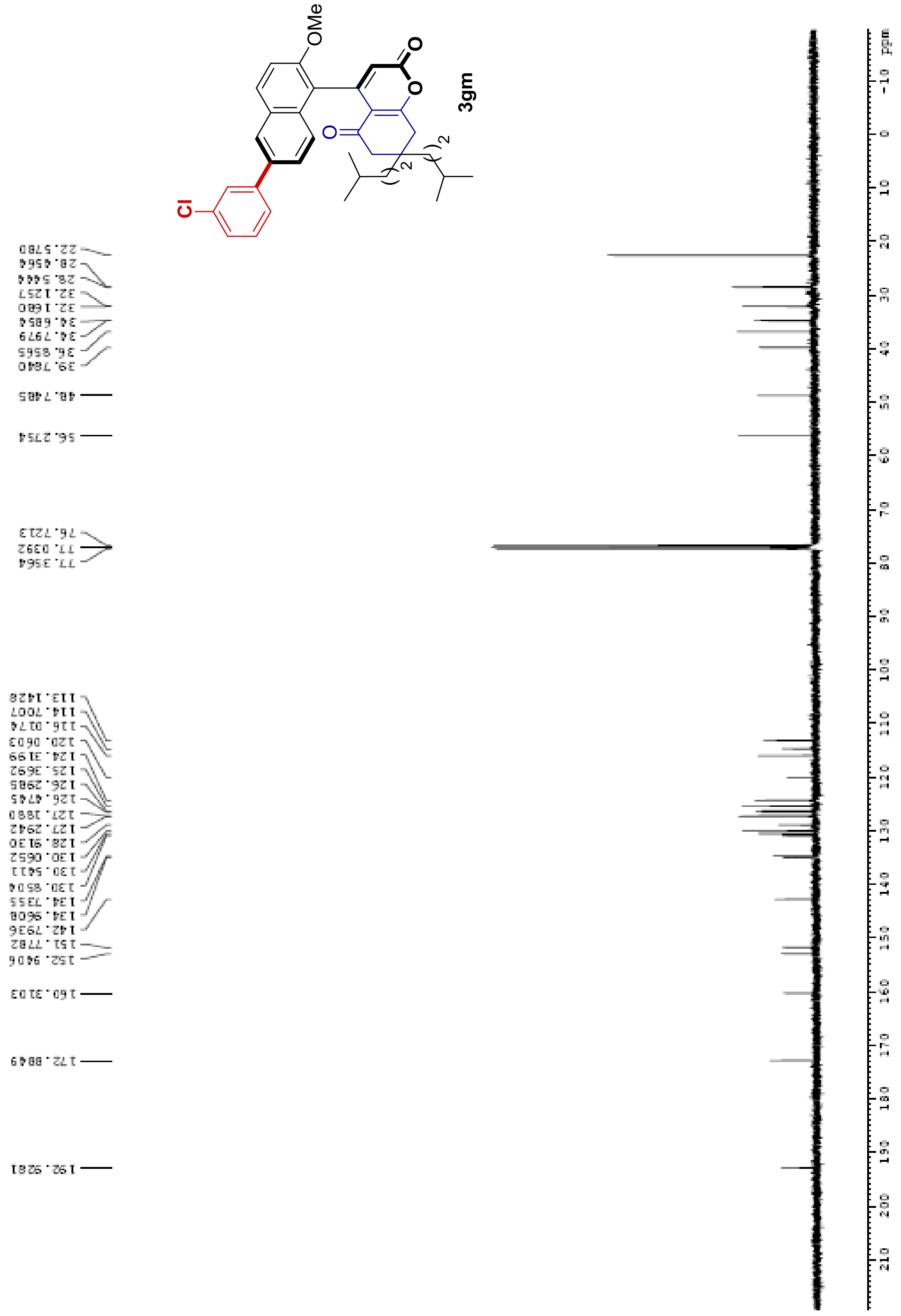

Supplementary Figure 79. HPLC Spectrum of racemic 3gm

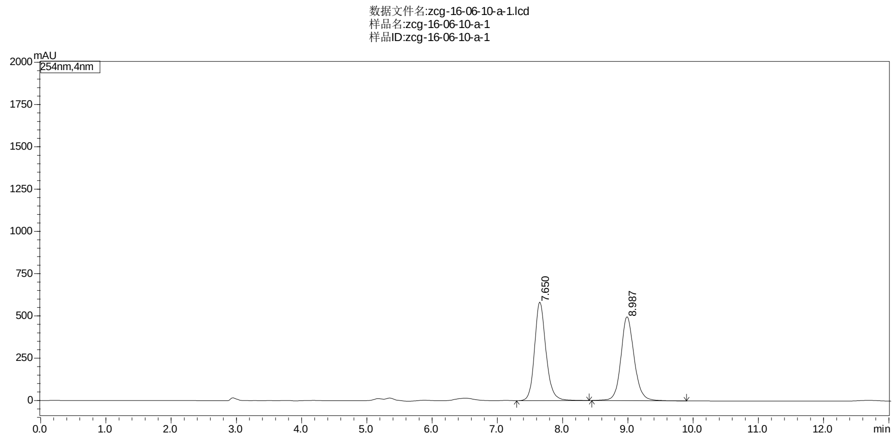

Peak#	Ret. Time	Area	Height	Area%	Height%
1	7.650	7077812	582844	50.279	54.061
2	8.987	6999163	495284	49.721	45.939
Total		14076975	1078128	100.000	100.000

Supplementary Figure 80. HPLC Spectrum of 3gm

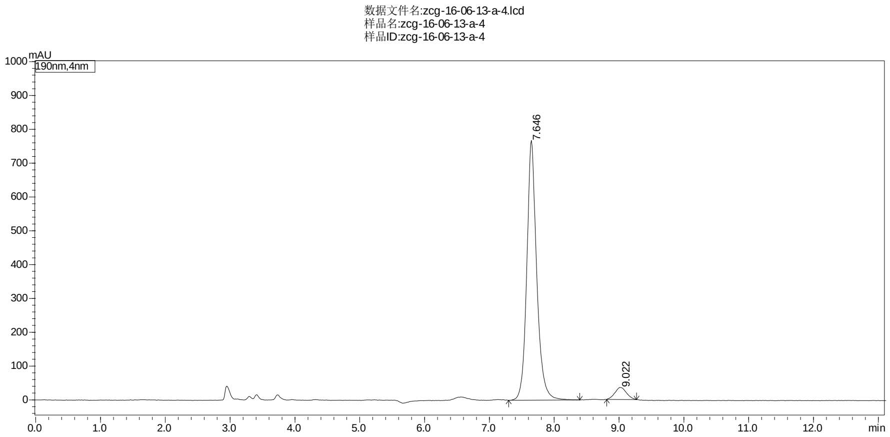

Peak#	Ret. Time	Area	Height	Area%	Height%
1	7.646	8154268	768209	94.861	95.587
2	9.022	441758	35464	5.139	4.413
Total		8596026	803673	100.000	100.000

Supplementary Figure 81. ^{1}H NMR Spectrum of 3gn

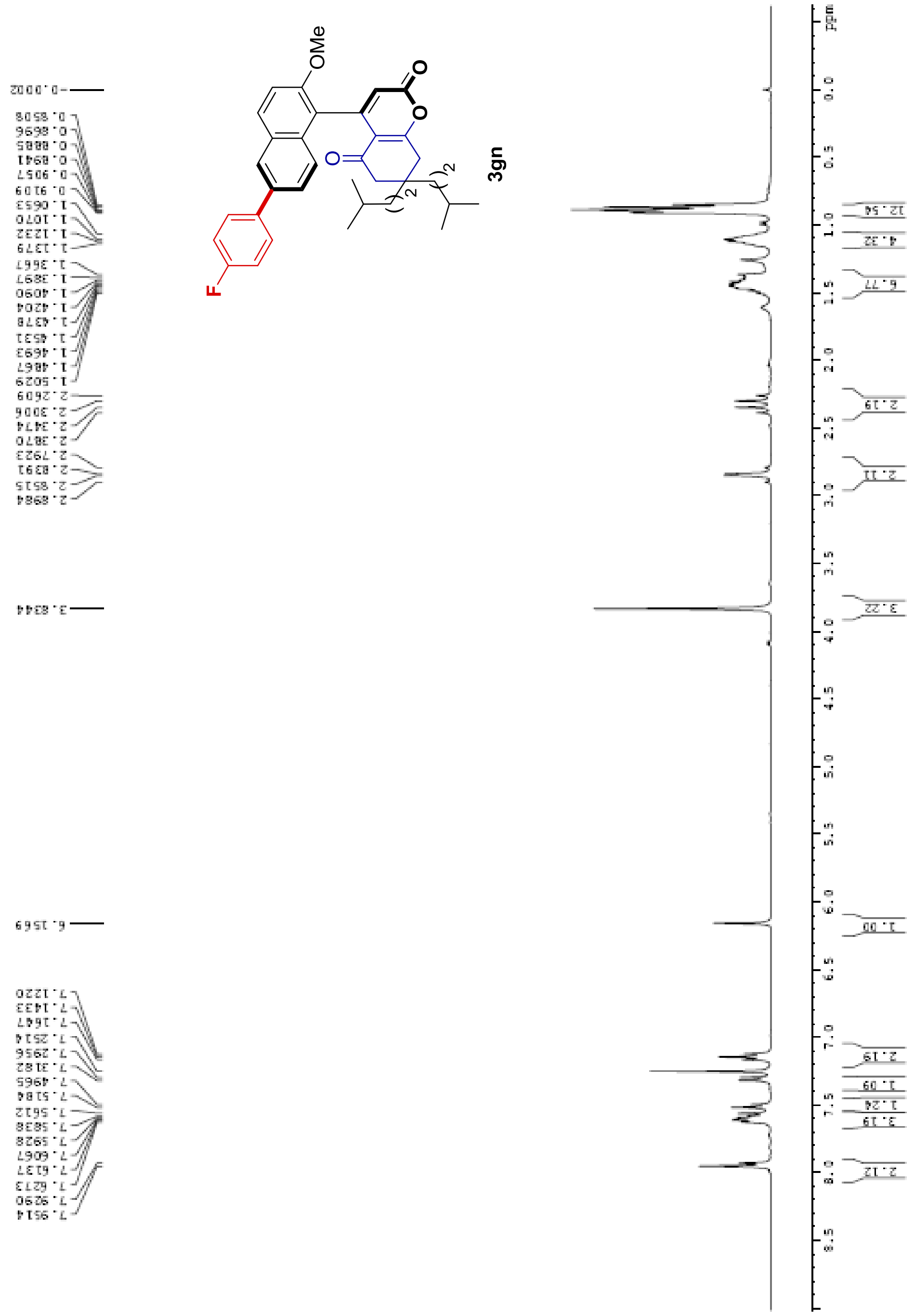

Supplementary Figure 82. ^{13}C NMR Spectrum of 3gn

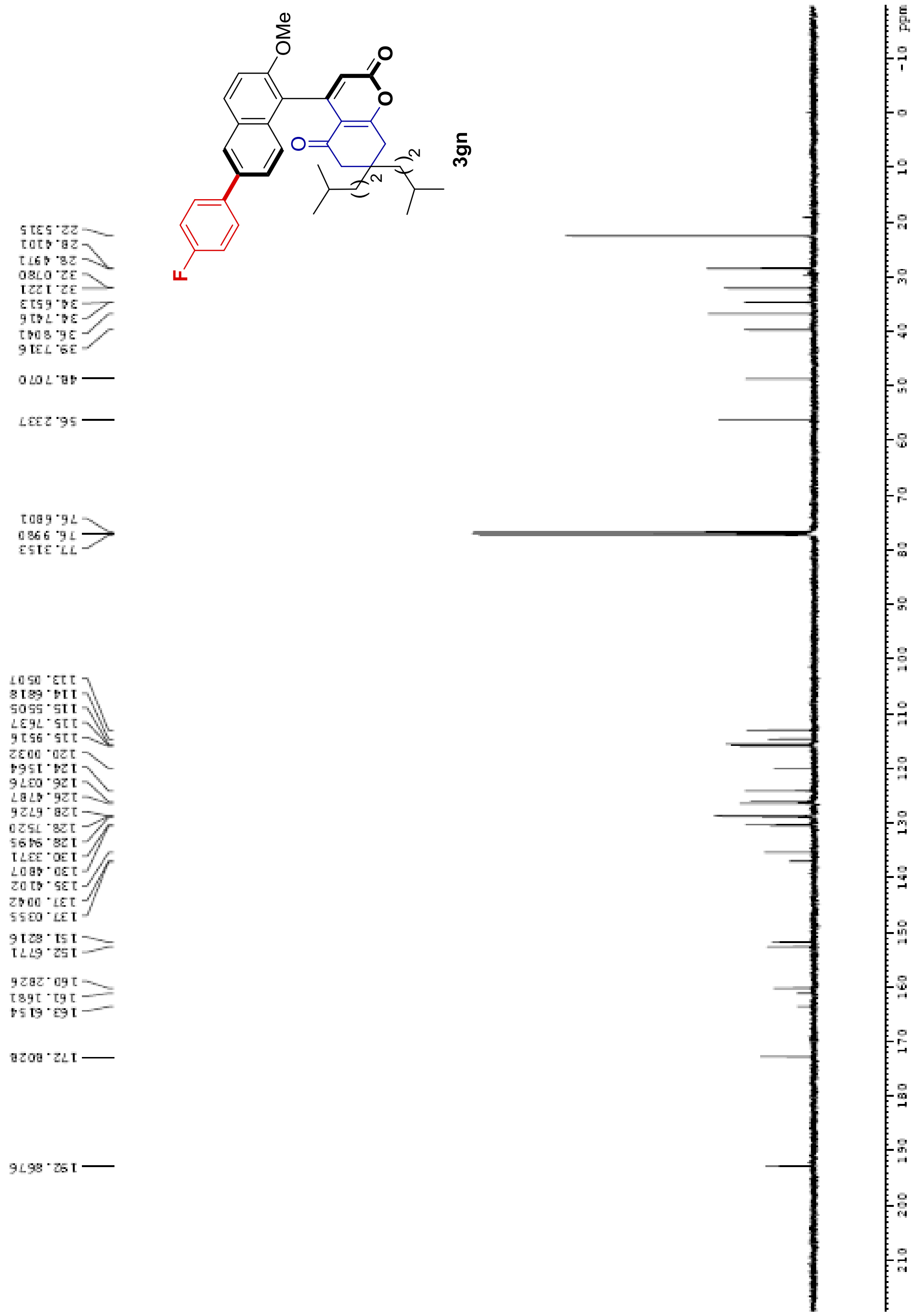

Supplementary Figure 83. HPLC Spectrum of racemic 3gn

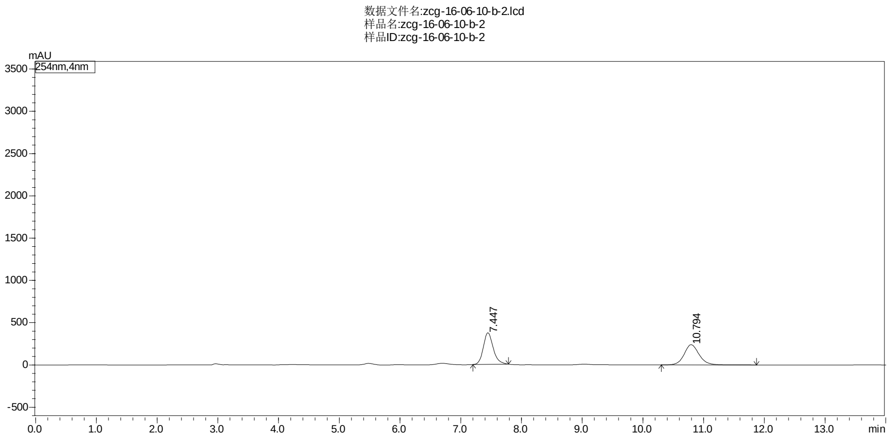

Peak#	Ret. Time	Area	Height	Area%	Height%
1	7.447	4271126	373837	50.981	60.969
2	10.794	4106737	239327	49.019	39.031
Total		8377862	613164	100.000	100.000

Supplementary Figure 84. HPLC Spectrum of 3gn

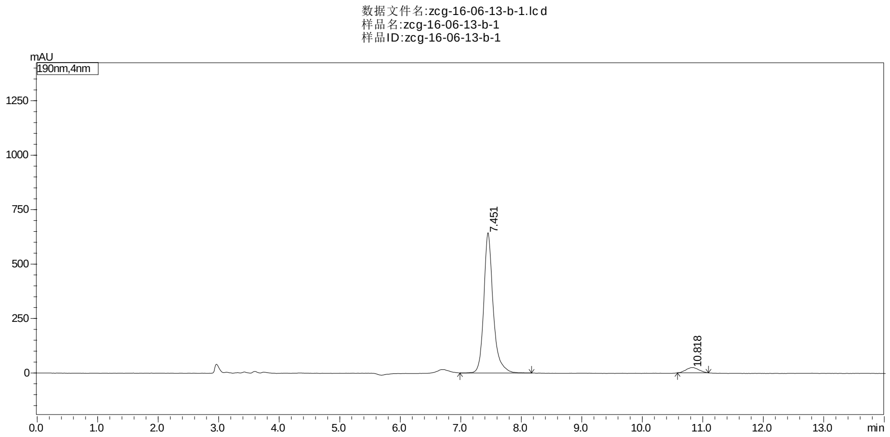

Peak#	Ret. Time	Area	Height	Area%	Height%
1	7.451	6933821	643907	95.157	96.390
2	10.818	352928	24113	4.843	3.610
Total		7286749	668021	100.000	100.000

Supplementary Figure 85. ^{19}F NMR Spectrum of 3gn

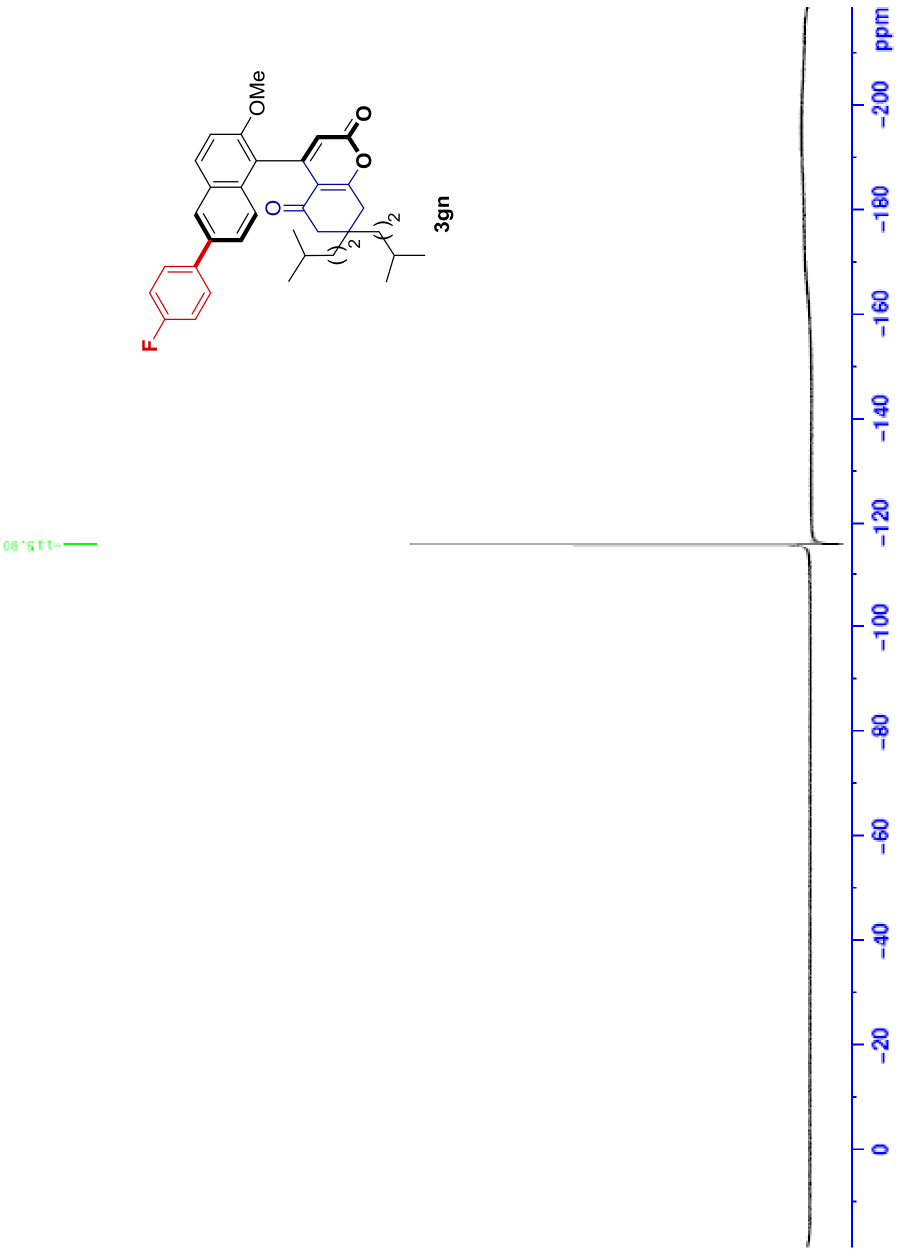

Supplementary Figure 86. ^{1}H NMR Spectrum of 3go

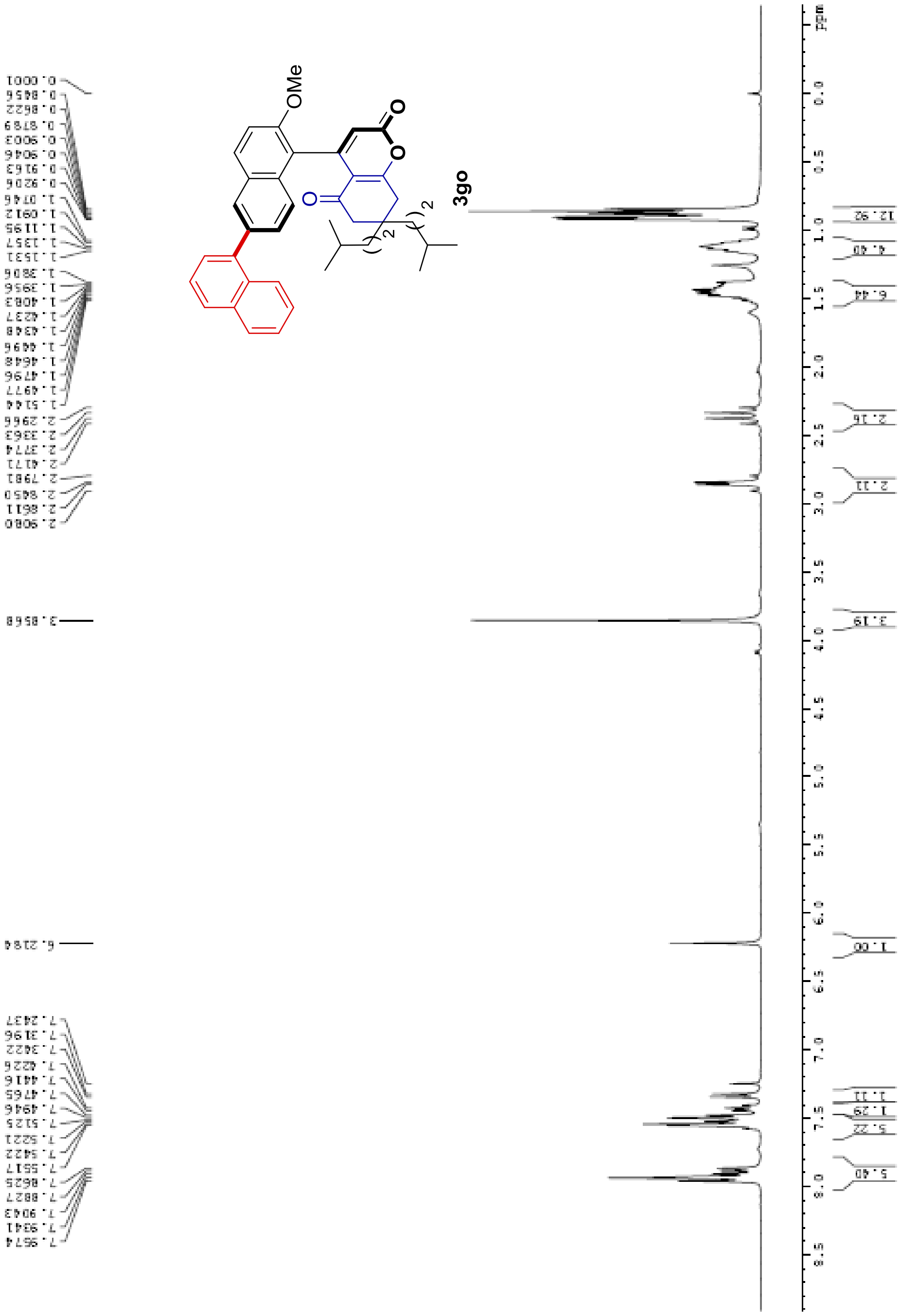

Supplementary Figure 87. ^{13}C NMR Spectrum of 3go

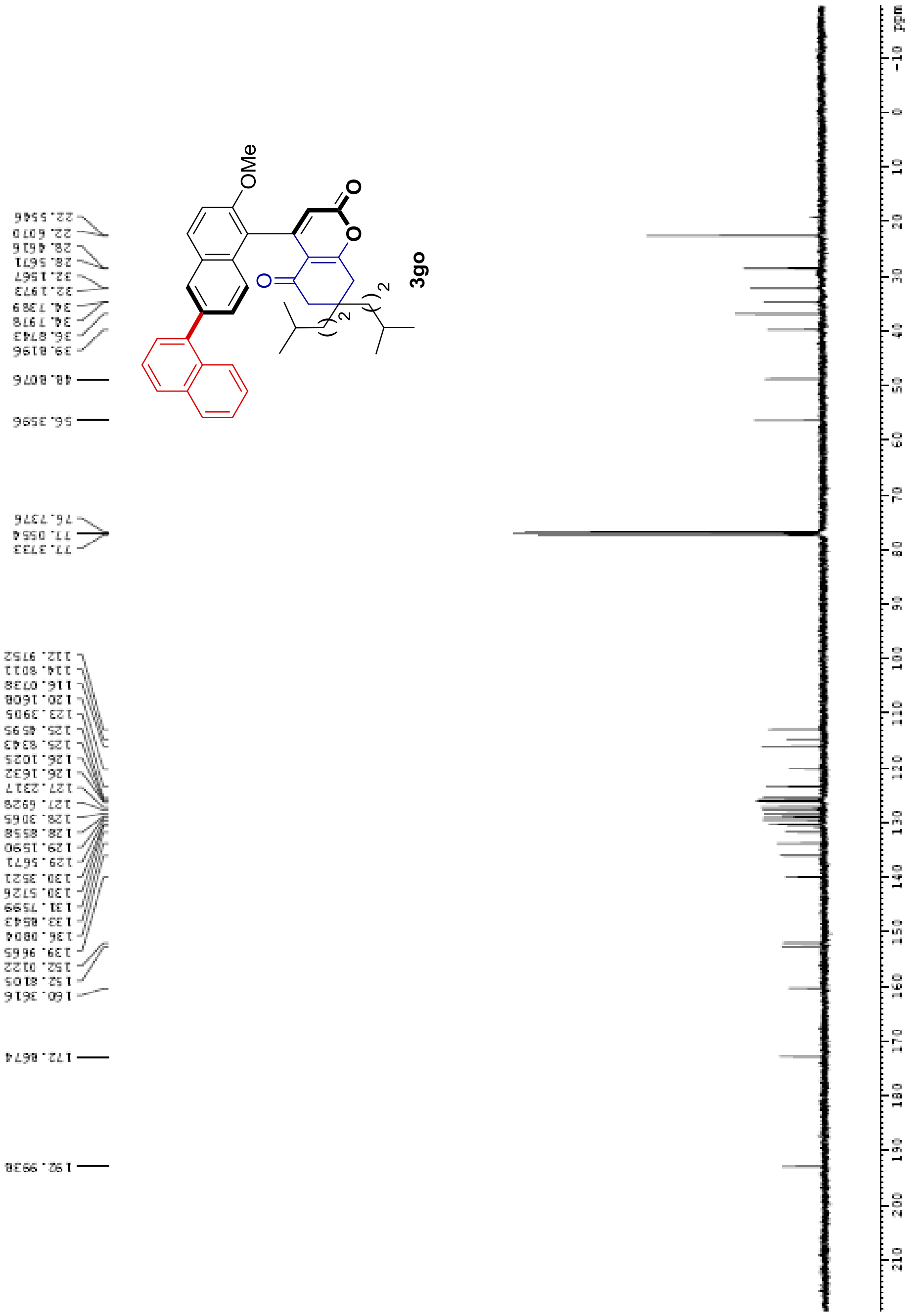

Supplementary Figure 88. HPLC Spectrum of racemic 3go

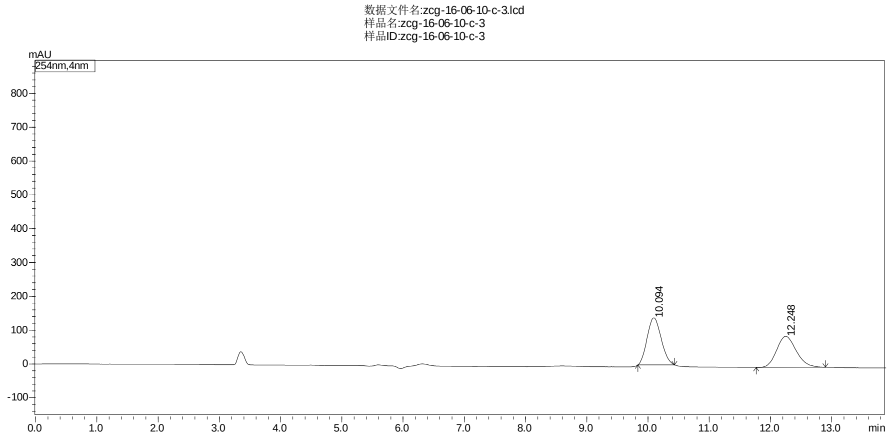

Peak#	Ret. Time	Area	Height	Area%	Height%
1	10.094	2221219	139039	52.106	60.283
2	12.248	2041701	91605	47.894	39.717
Total		4262920	230643	100.000	100.000

Supplementary Figure 89. HPLC Spectrum of 3go

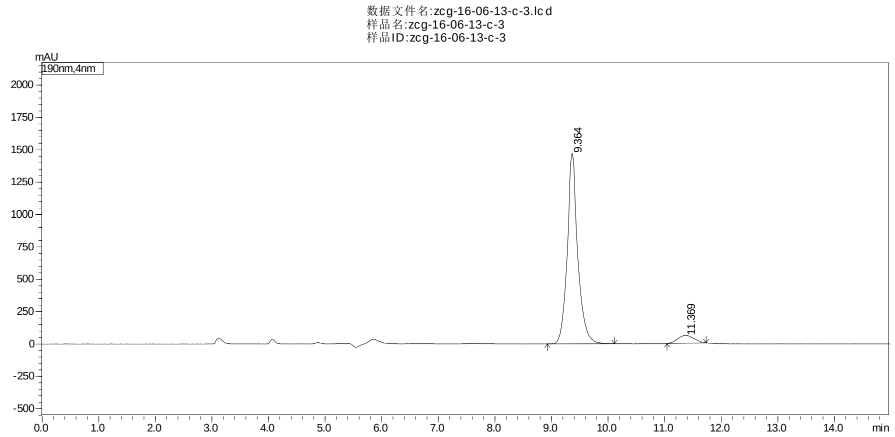

Peak#	Ret. Time	Area	Height	Area%	Height%
1	9.364	18923994	1471967	94.056	96.084
2	11.369	1195912	59997	5.944	3.916
Total		20119906	1531964	100.000	100.000

Supplementary Figure 90. ^{1}H NMR Spectrum of 3gp

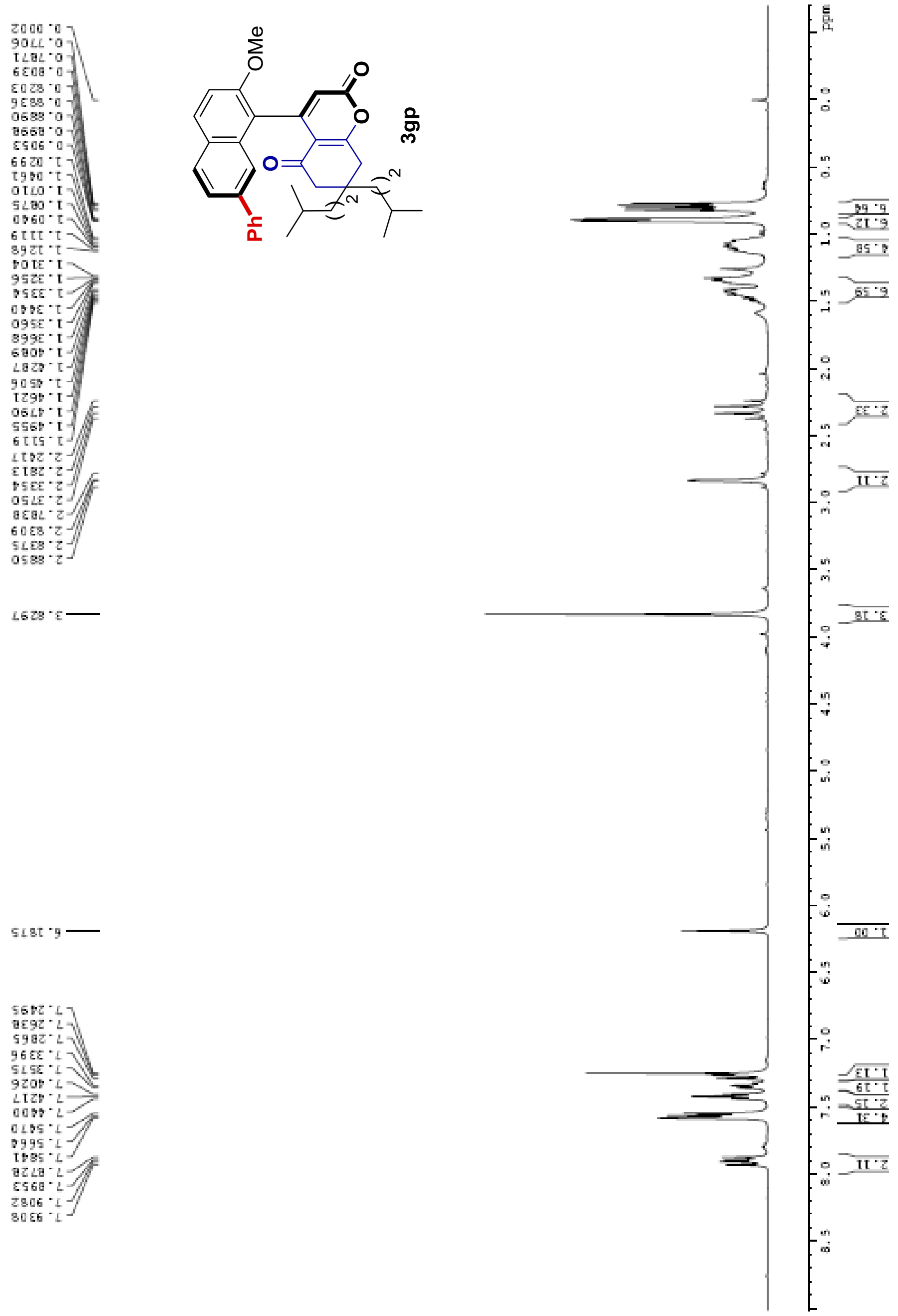

Supplementary Figure 91. ^{13}C NMR Spectrum of 3gp

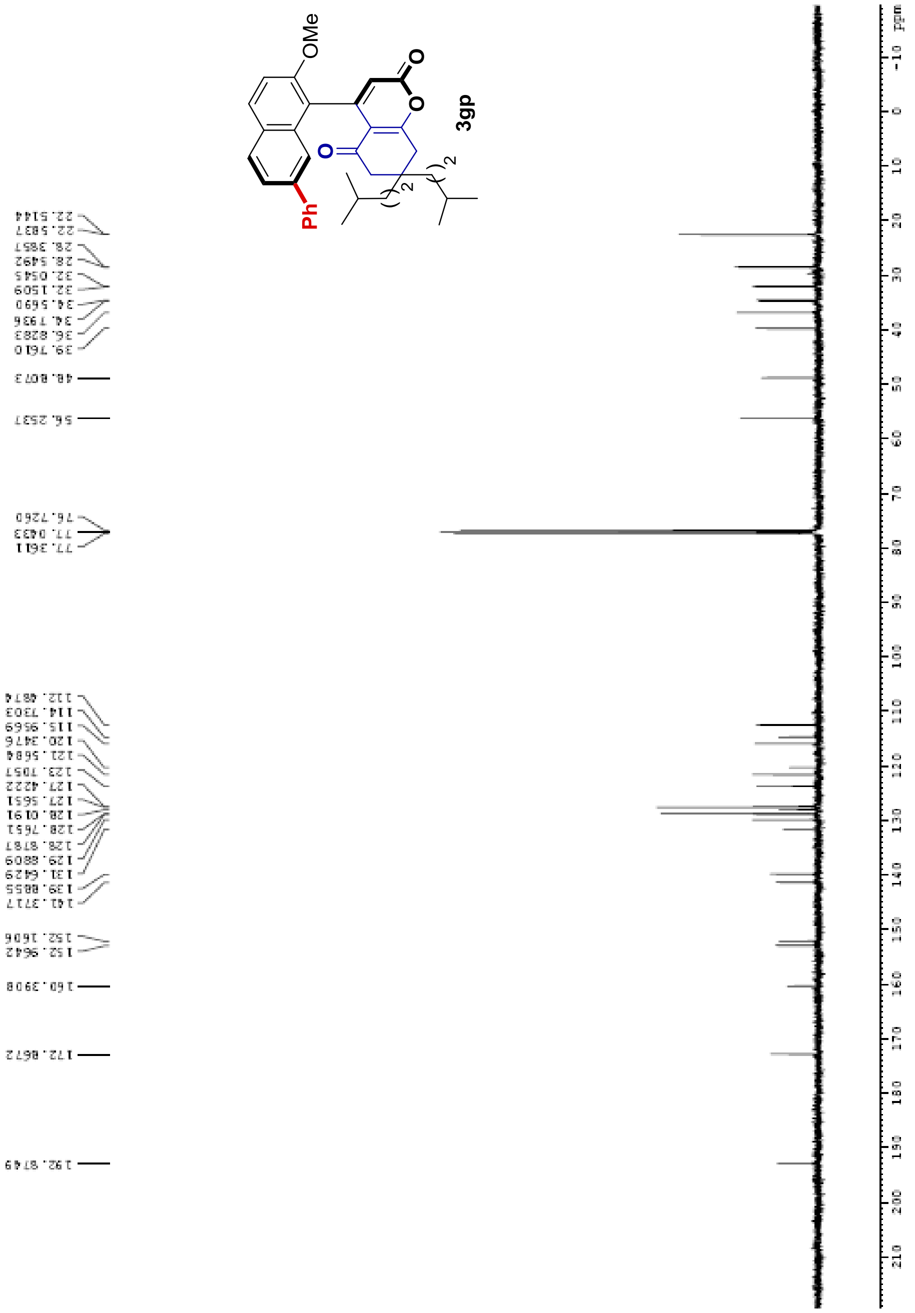

Supplementary Figure 92. HPLC Spectrum of racemic 3gp

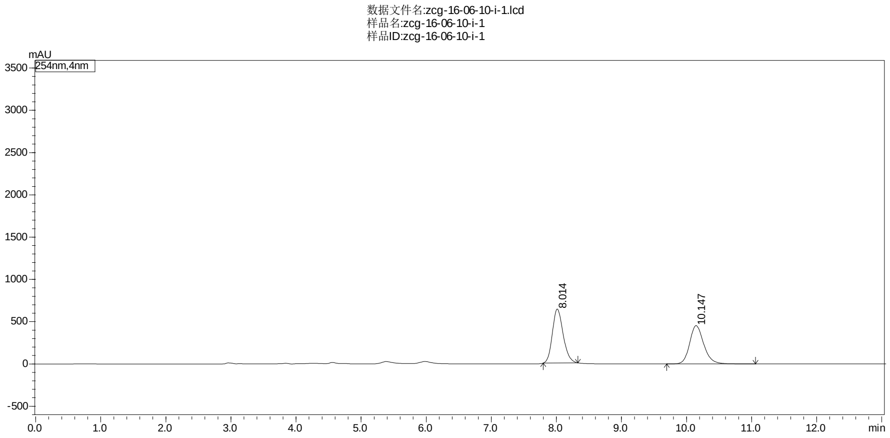

Peak#	Ret. Time	Area	Height	Area%	Height%
1	8.014	7364436	638487	51.877	58.463
2	10.147	6831546	453629	48.123	41.537
Total		14195982	1092117	100.000	100.000

Supplementary Figure 93. HPLC Spectrum of 3gp

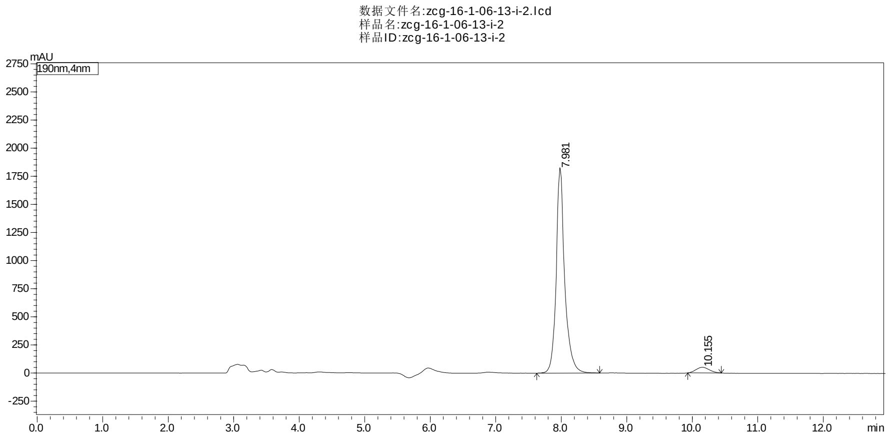

Peak#	Ret. Time	Area	Height	Area%	Height%
1	7.981	16583727	1827685	95.927	97.304
2	10.155	704089	50645	4.073	2.696
Total		17287816	1878330	100.000	100.000

Supplementary Figure 94. ^{1}H NMR Spectrum of 3gq

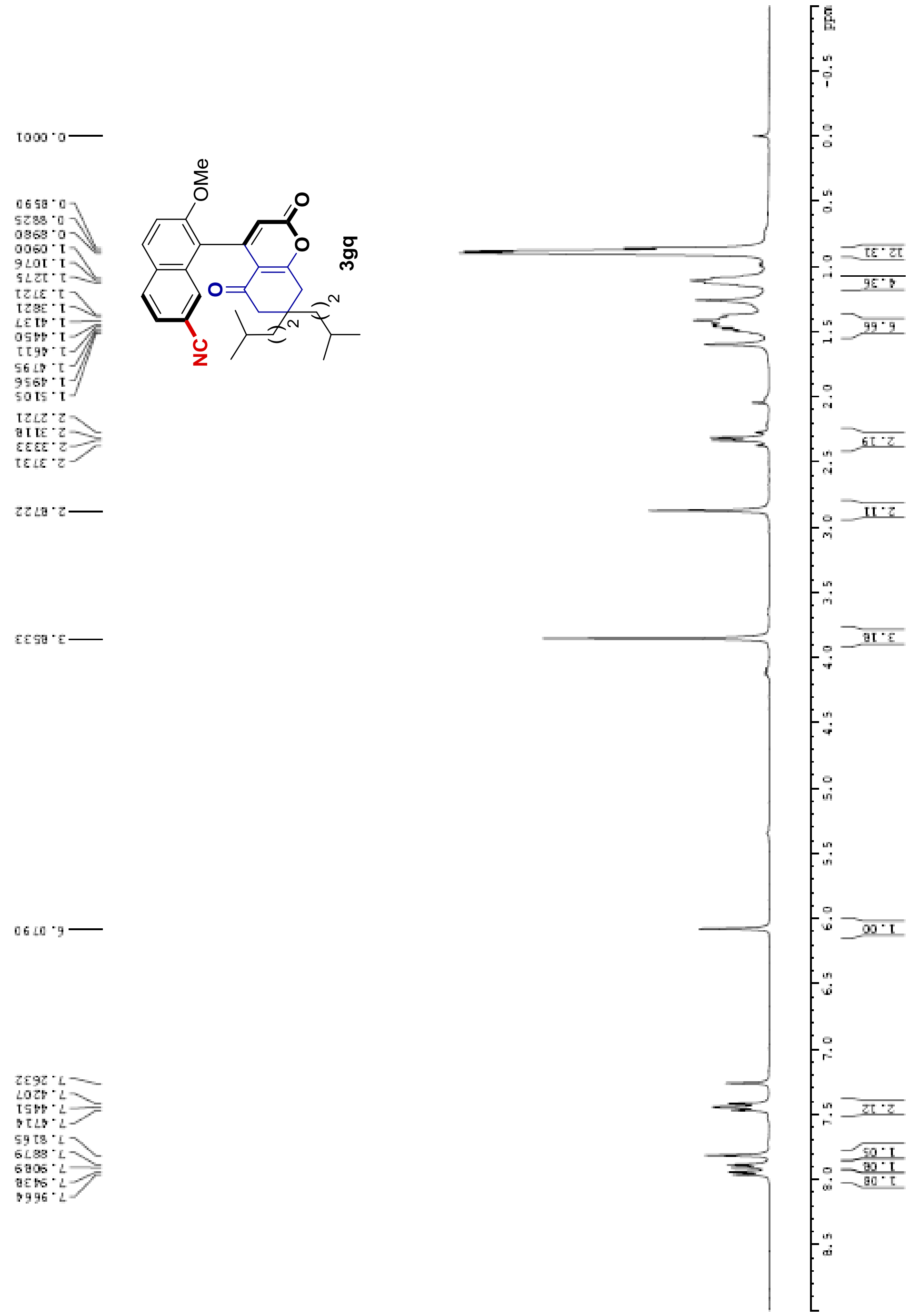

Supplementary Figure 95. ^{13}C NMR Spectrum of 3gq

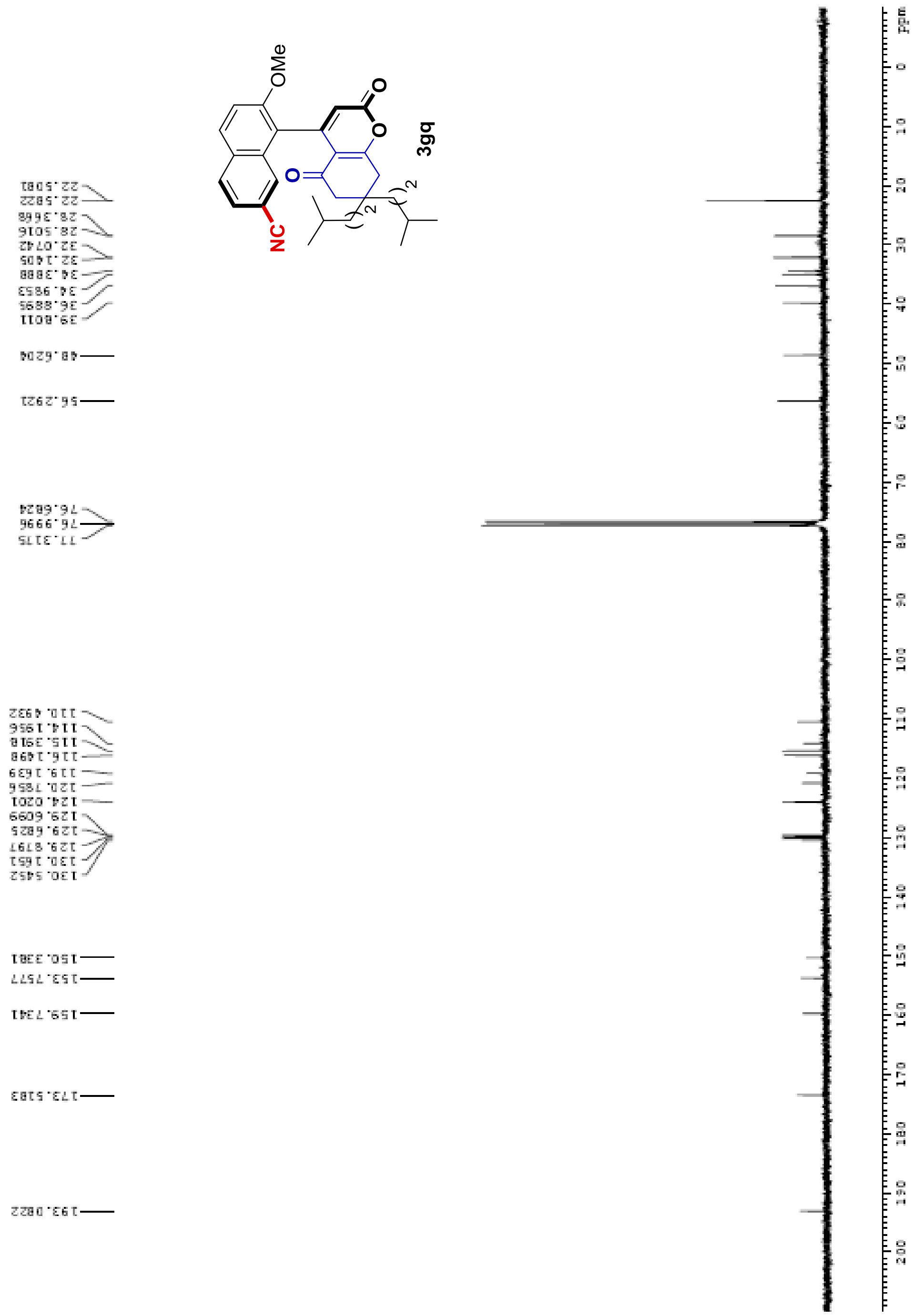

Supplementary Figure 96. HPLC Spectrum of racemic 3gq

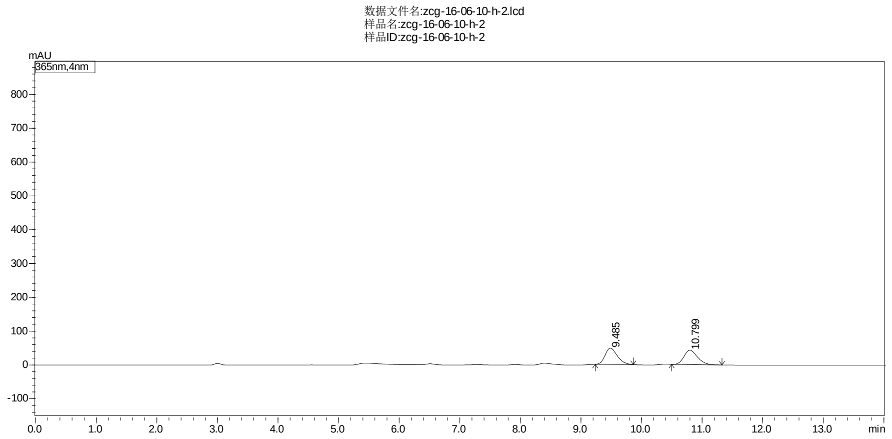

Peak#	Ret. Time	Area	Height	Area%	Height%
1	9.485	676990	48172	50.573	53.134
2	10.799	661654	42489	49.427	46.866
Total		1338644	90661	100.000	100.000

Supplementary Figure 97. HPLC Spectrum of 3gq

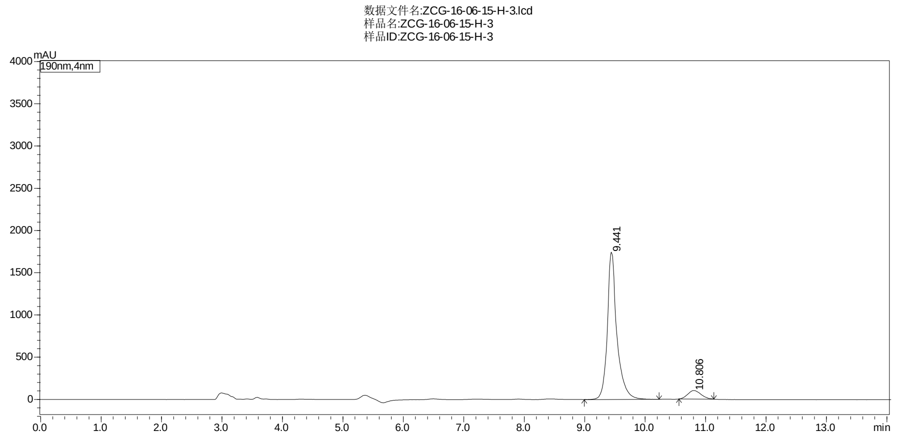

Peak#	Ret. Time	Area	Height	Area%	Height%
1	9.441	19233666	1746260	92.685	94.545
2	10.806	1518071	100753	7.315	5.455
Total		20751737	1847013	100.000	100.000

Supplementary Figure 98. ^{1}H NMR Spectrum of 3gr

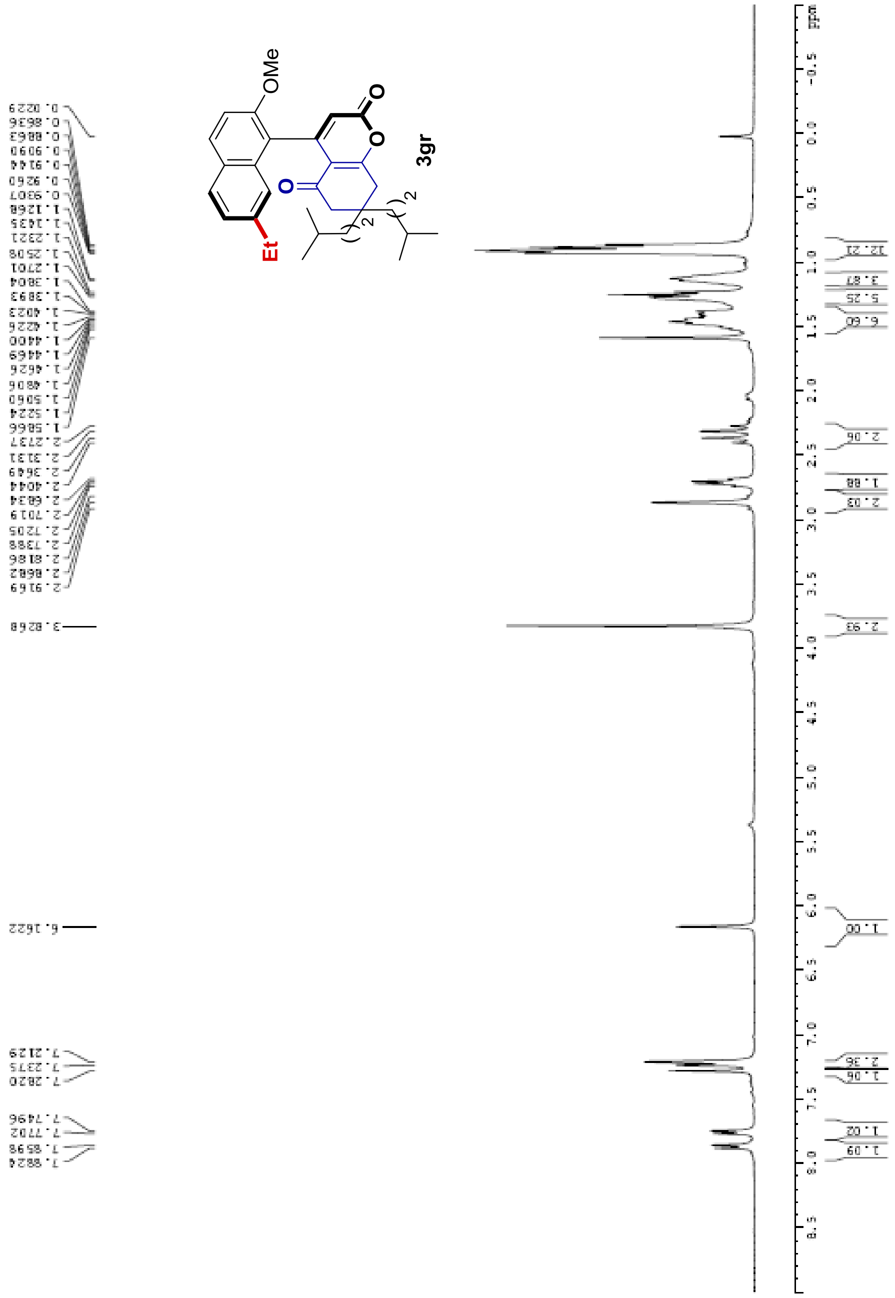

Supplementary Figure 99. ^{13}C NMR Spectrum of 3gr

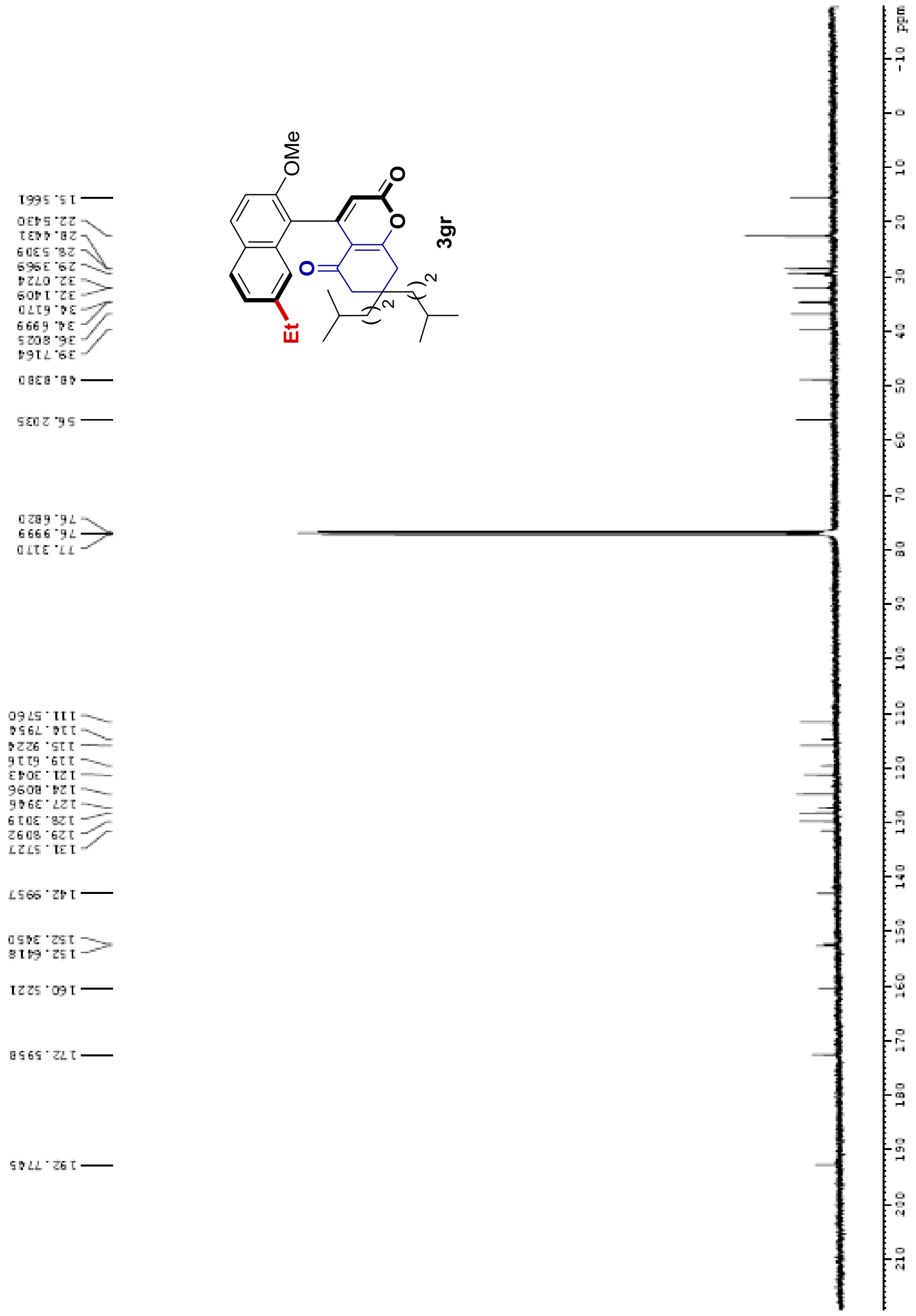

Supplementary Figure 100. HPLC Spectrum of racemic 3gr

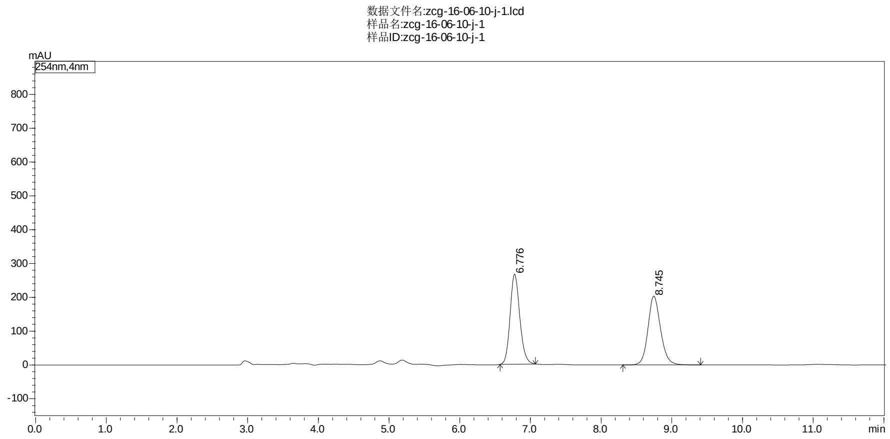

Peak#	Ret. Time	Area	Height	Area%	Height%
1	6.776	2540743	266769	50.166	56.733
2	8.745	2523925	203449	49.834	43.267
Total		5064668	470218	100.000	100.000

Supplementary Figure 101. HPLC Spectrum of 3gr

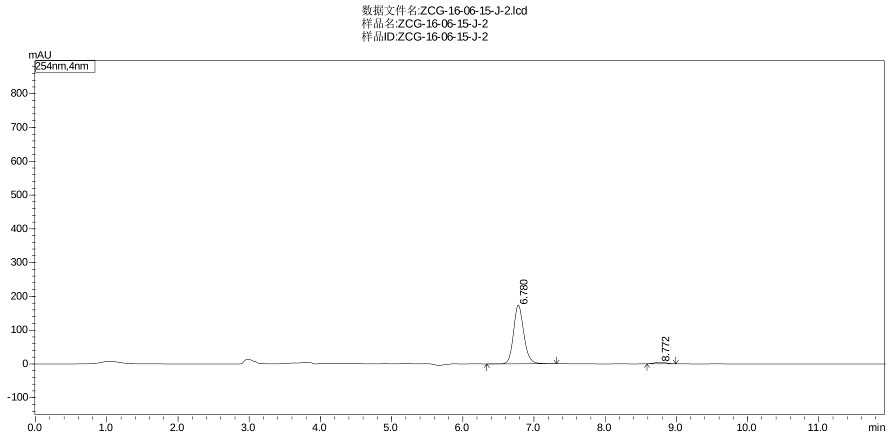

Peak#	Ret. Time	Area	Height	Area%	Height%
1	6.780	1738998	173713	96.799	97.149
2	8.772	57500	5098	3.201	2.851
Total		1796497	178811	100.000	100.000

Supplementary Figure 102. ^{1}H NMR Spectrum of 3gs

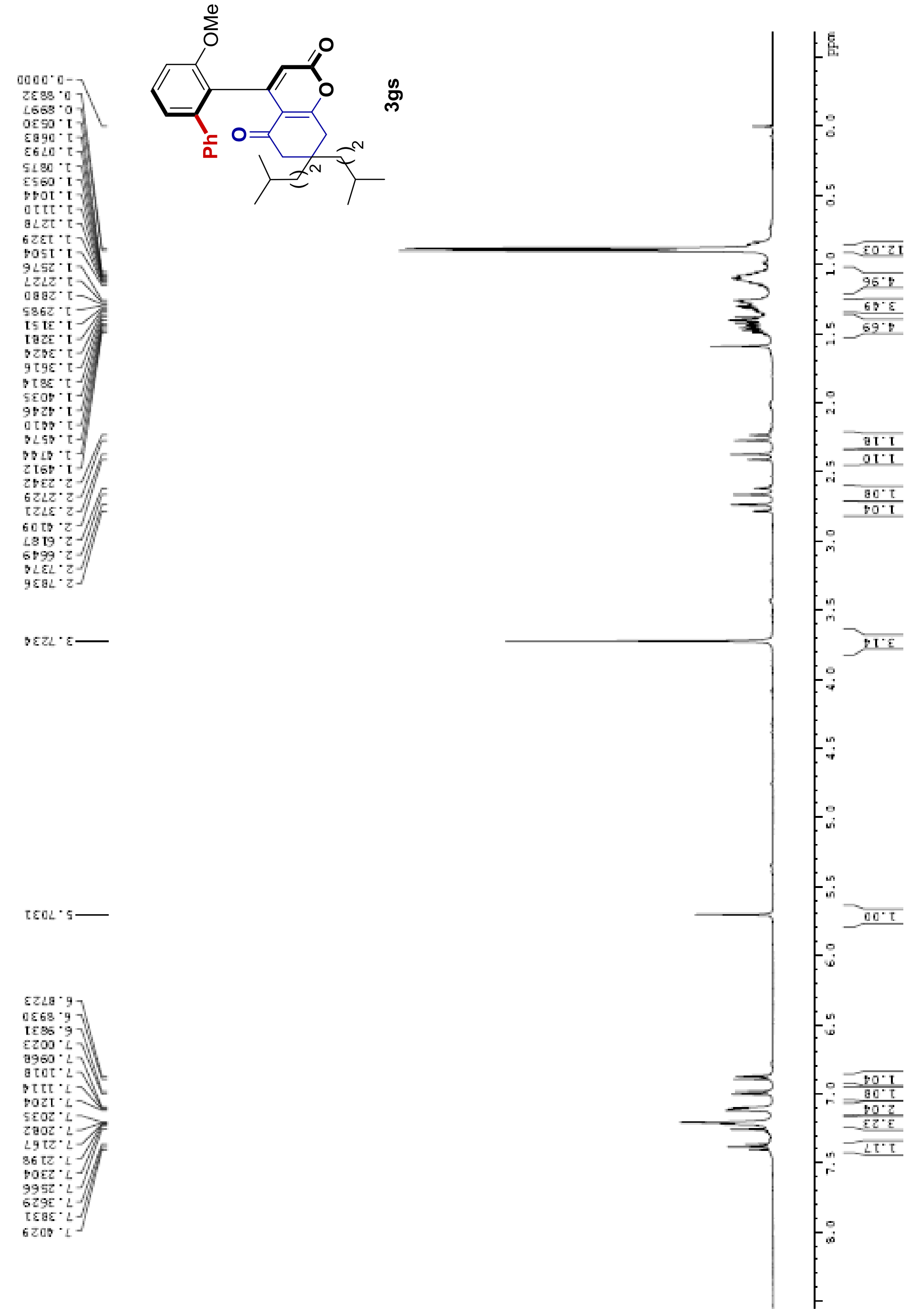

Supplementary Figure 103. ^{13}C NMR Spectrum of 3gs

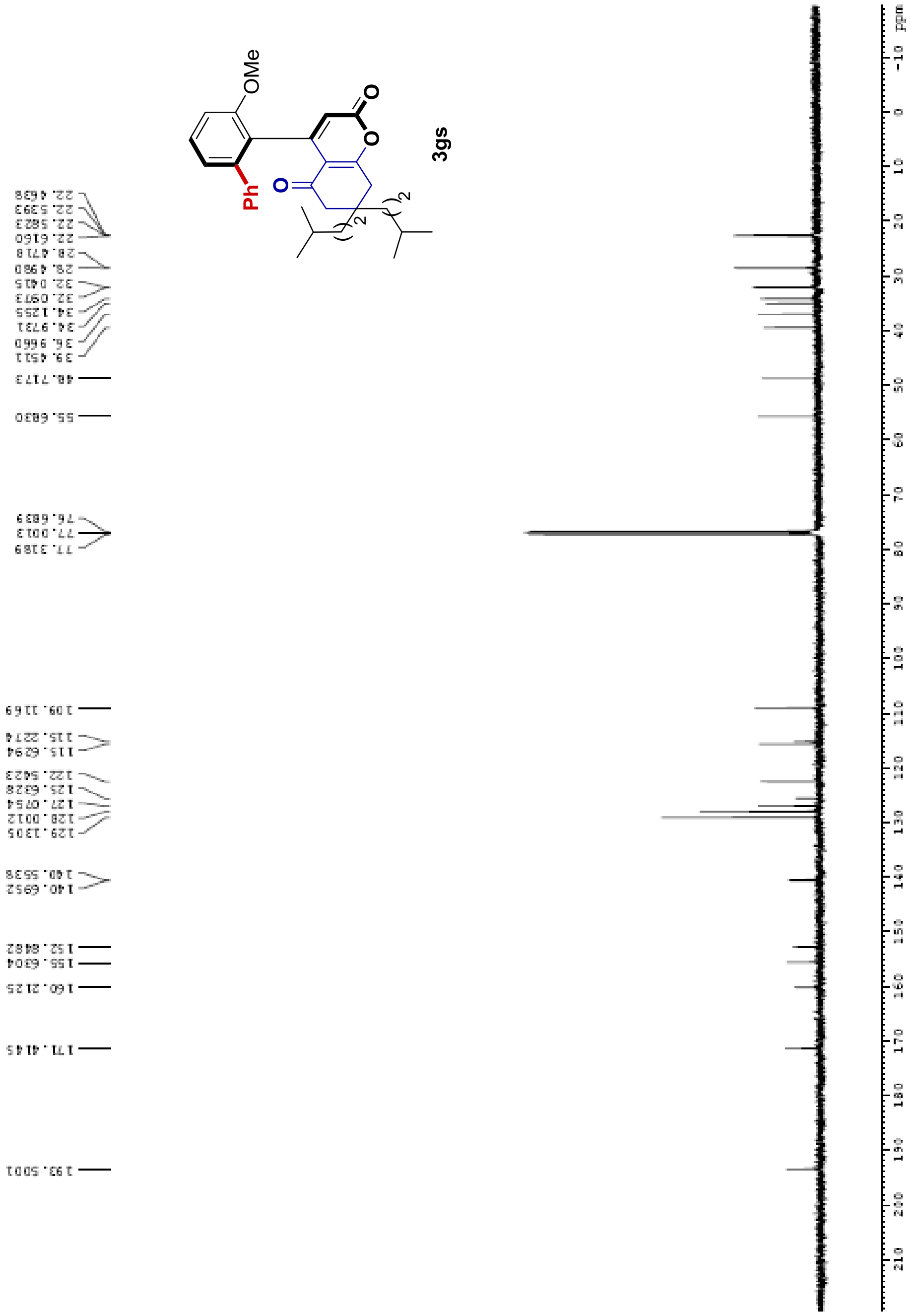

Supplementary Figure 104. HPLC Spectrum of racemic 3gs

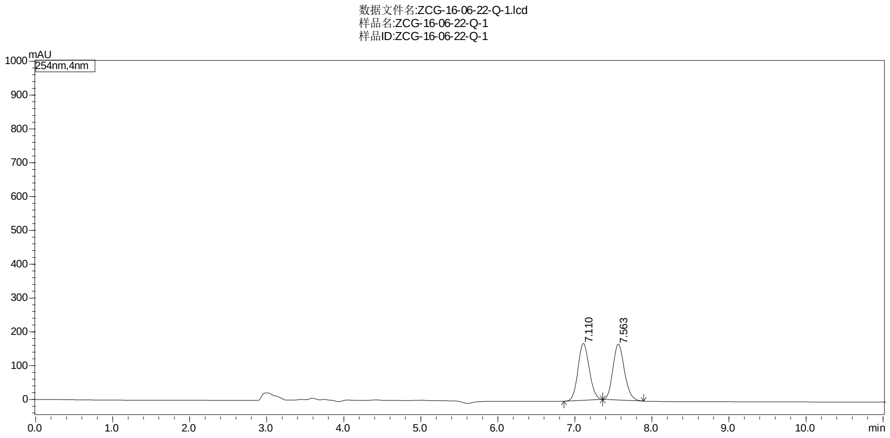

Peak#	Ret. Time	Area	Height	Area%	Height%
1	7.110	1715873	168339	49.969	50.426
2	7.563	1717971	165496	50.031	49.574
Total		3433844	333835	100.000	100.000

Supplementary Figure 105. HPLC Spectrum of 3gs

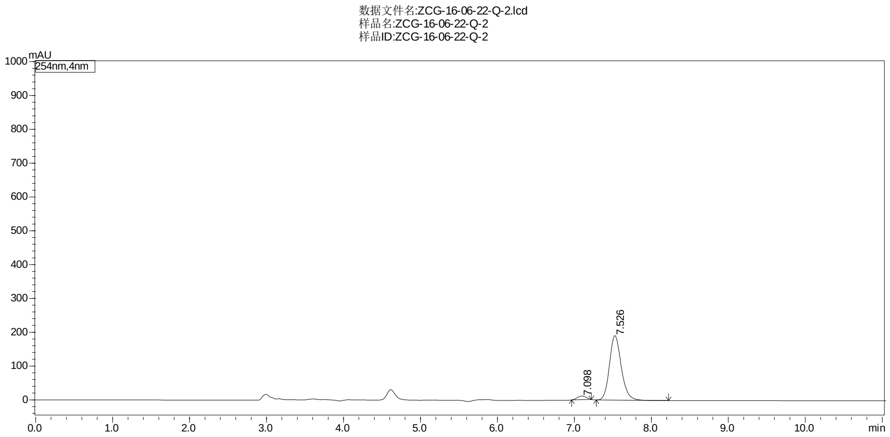

Peak#	Ret. Time	Area	Height	Area%	Height%
1	7.098	87885	10767	4.135	5.341
2	7.526	2037287	190826	95.865	94.659
Total		2125172	201593	100.000	100.000

Supplementary Figure 106. ^{1}H NMR Spectrum of 3gt

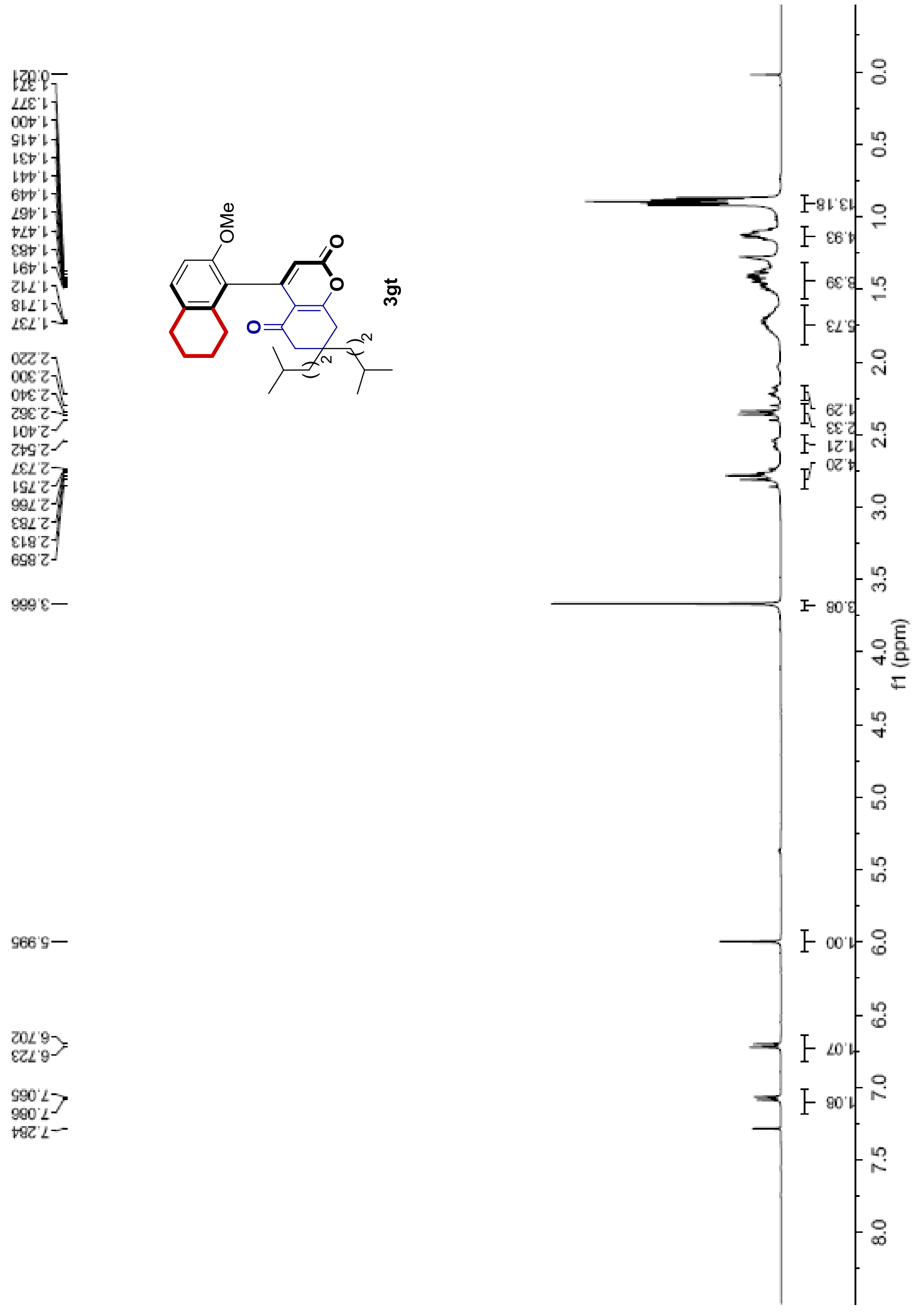

Supplementary Figure 107. ^{13}C NMR Spectrum of 3gt

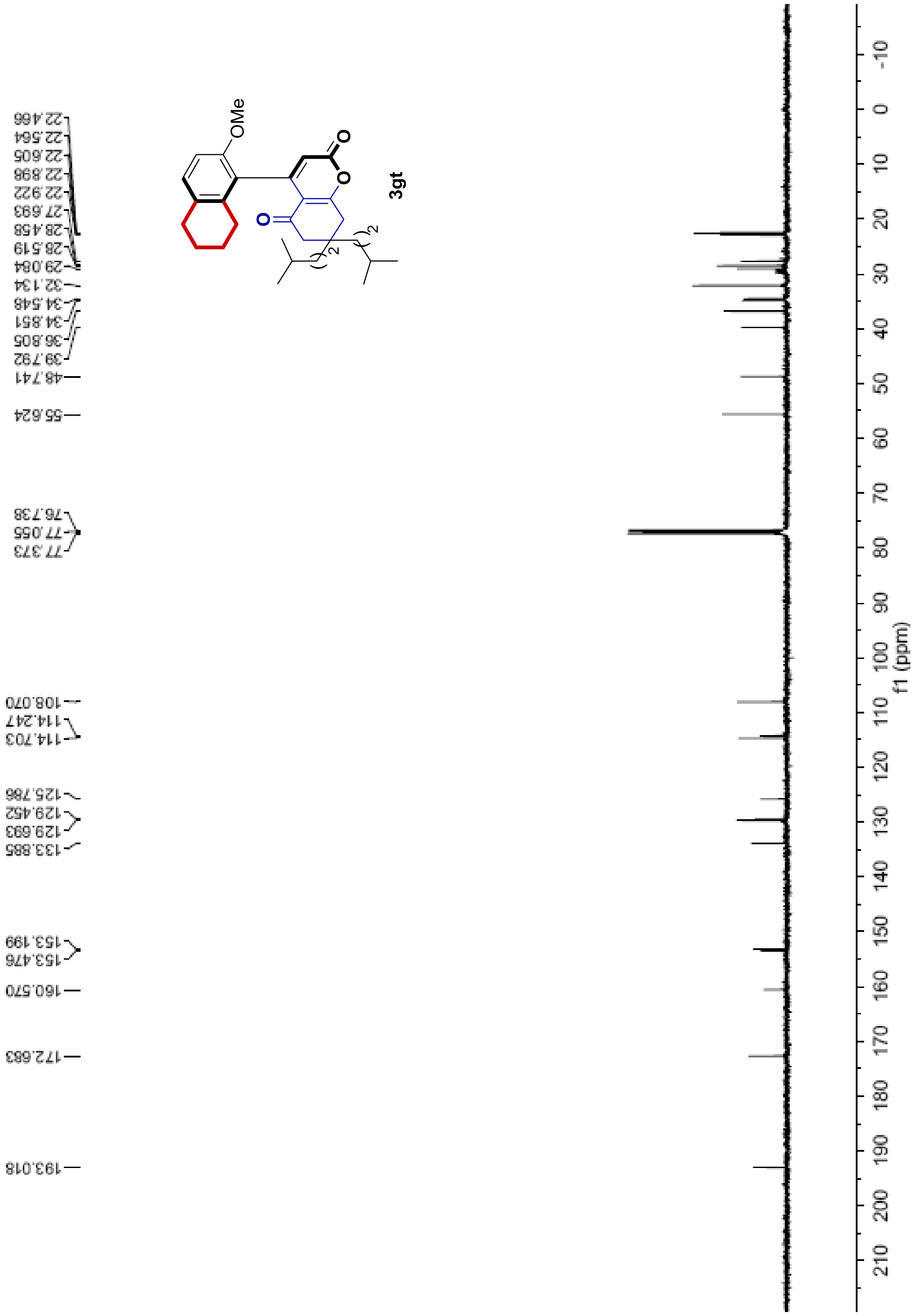

Supplementary Figure 108. ^{13}C NMR Spectrum of 3gt

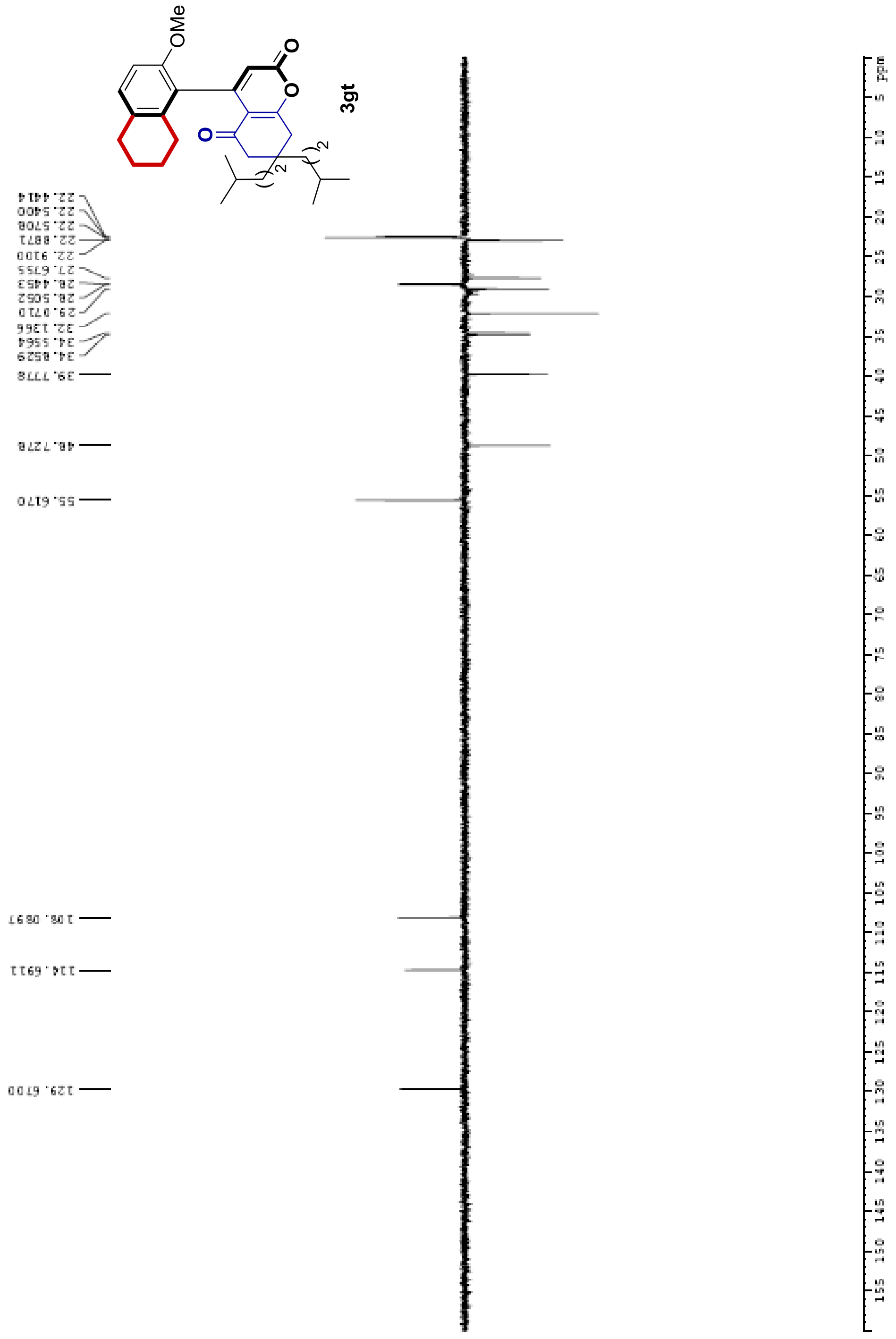

Supplementary Figure 109. HPLC Spectrum of racemic 3gt

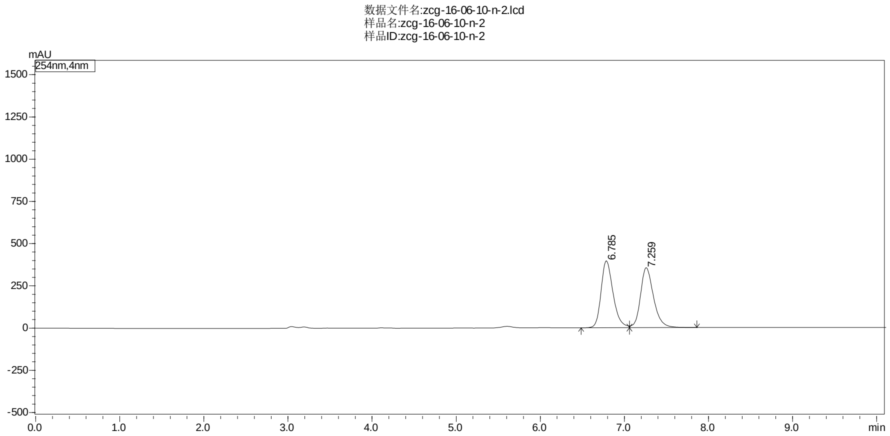

Peak#	Ret. Time	Area	Height	Area%	Height%
1	6.785	3928716	396665	50.613	52.821
2	7.259	3833479	354298	49.387	47.179
Total		7762196	750964	100.000	100.000

Supplementary Figure 110. HPLC Spectrum of 3gt

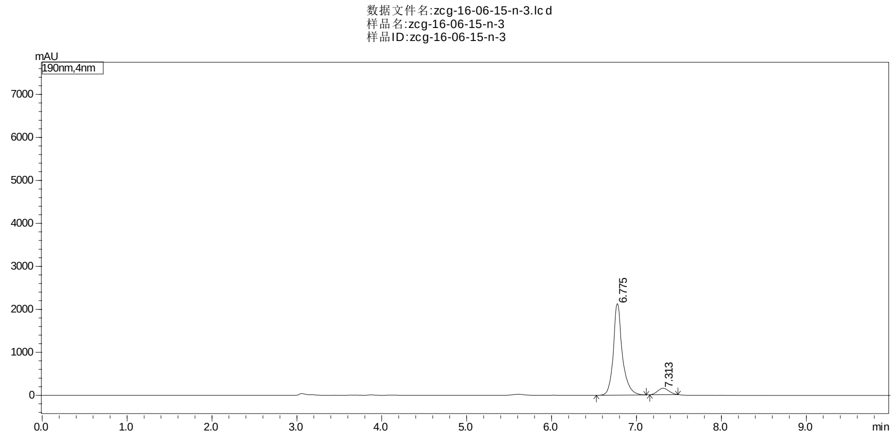

Peak#	Ret. Time	Area	Height	Area%	Height%
1	6.775	15674564	2130564	91.932	93.436
2	7.313	1375667	149665	8.068	6.564
Total		17050230	2280228	100.000	100.000

Supplementary Figure 111. ^{1}H NMR Spectrum of 3au

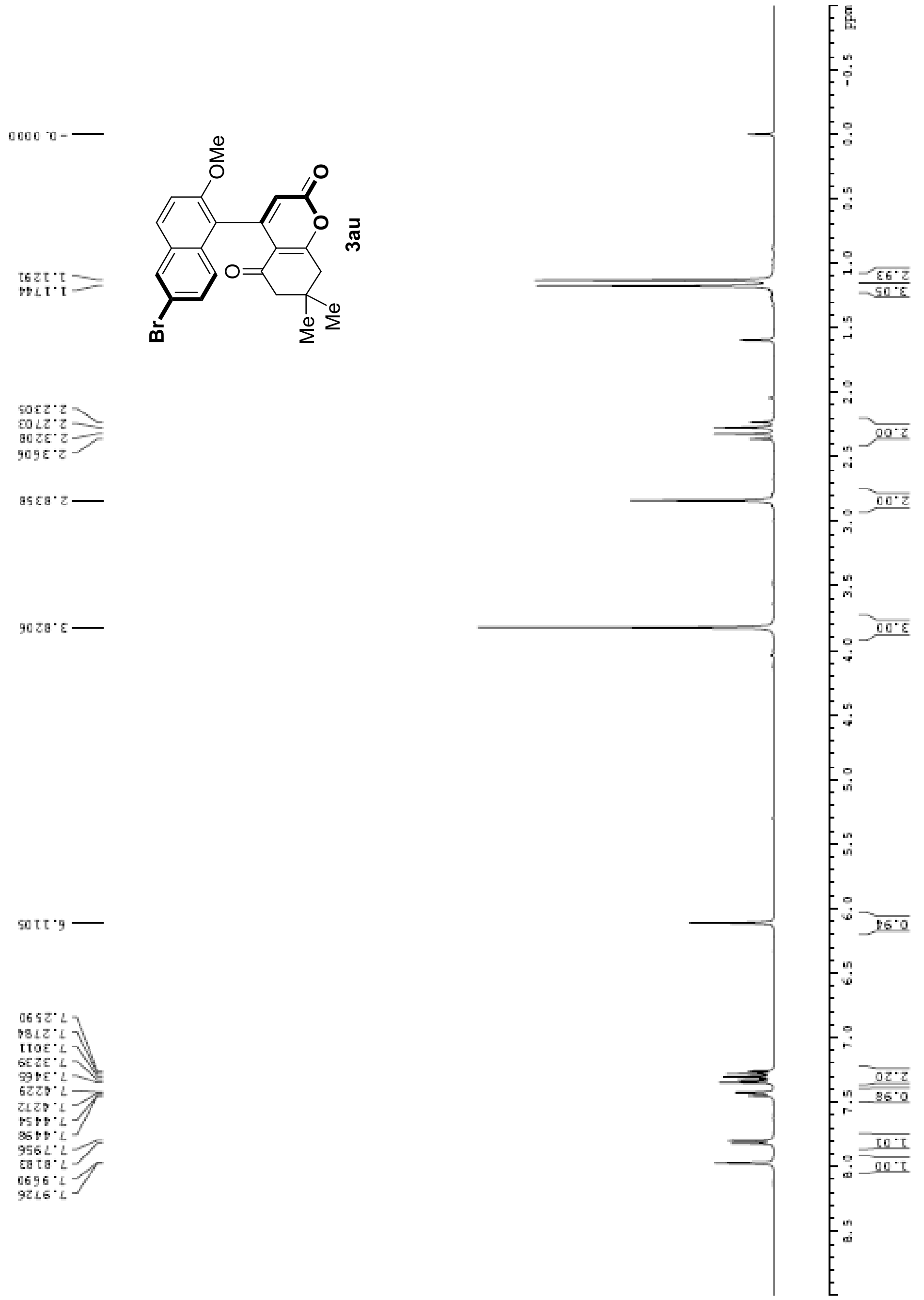

Supplementary Figure 112. ^{13}C NMR Spectrum of 3au

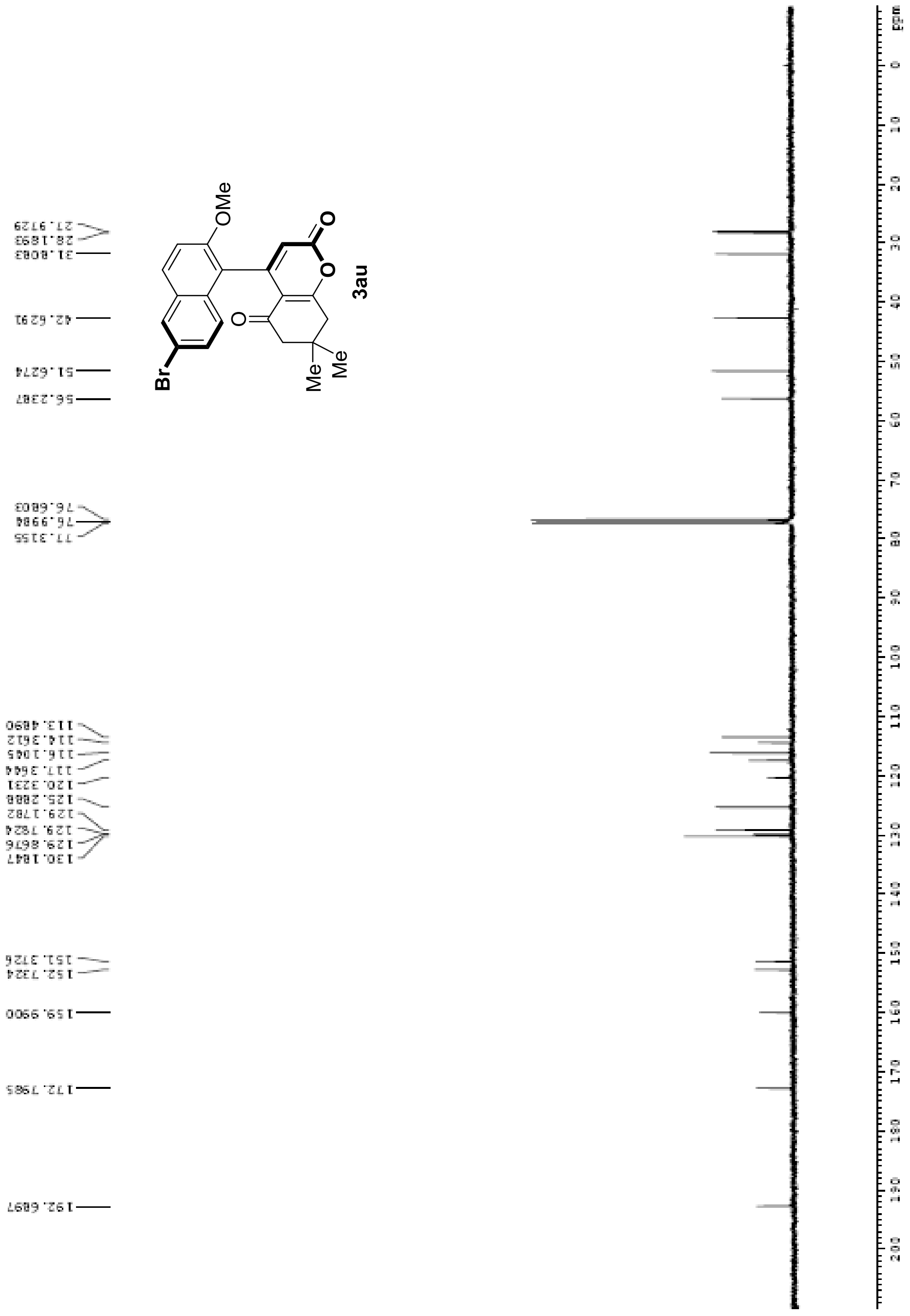

Supplementary Figure 113. HPLC Spectrum of racemic 3au

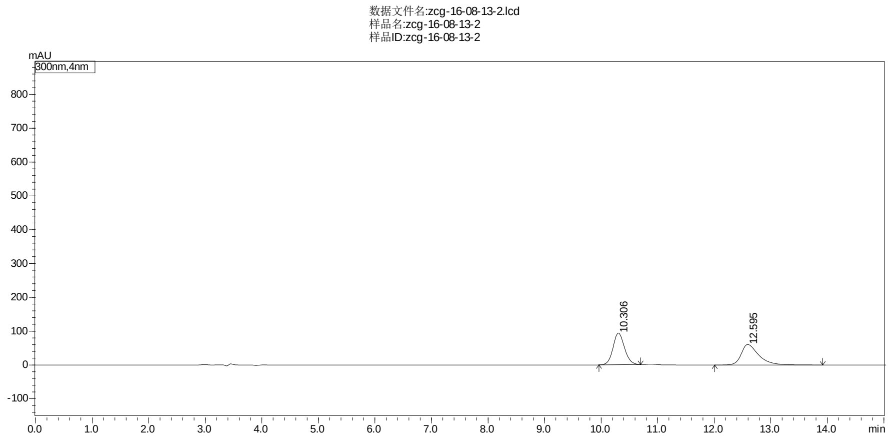

Peak#	Ret. Time	Area	Height	Area%	Height%
1	10.306	1331070	93867	50.295	60.677
2	12.595	1315457	60832	49.705	39.323
Total		2646527	154699	100.000	100.000

Supplementary Figure 114. HPLC Spectrum of 3au

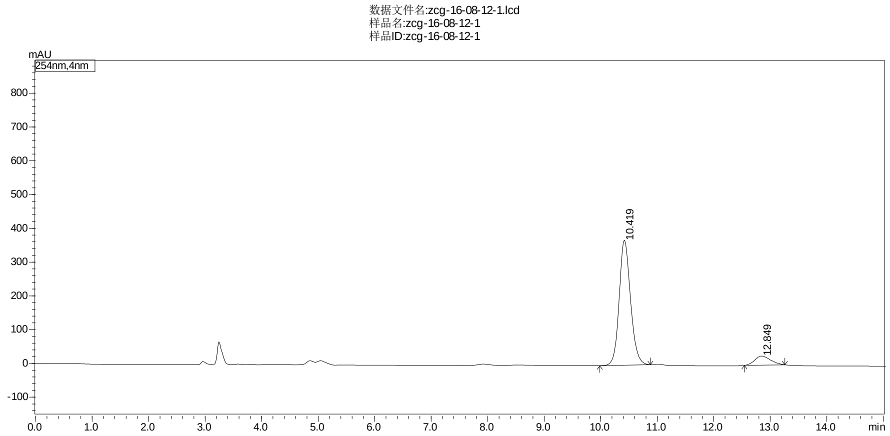

Peak#	Ret. Time	Area	Height	Area%	Height%
1	10.419	5164108	369842	90.855	93.259
2	12.849	519786	26732	9.145	6.741
Total		5683895	396574	100.000	100.000

Supplementary Figure 115. HPLC Spectrum of 3au after first crystallization

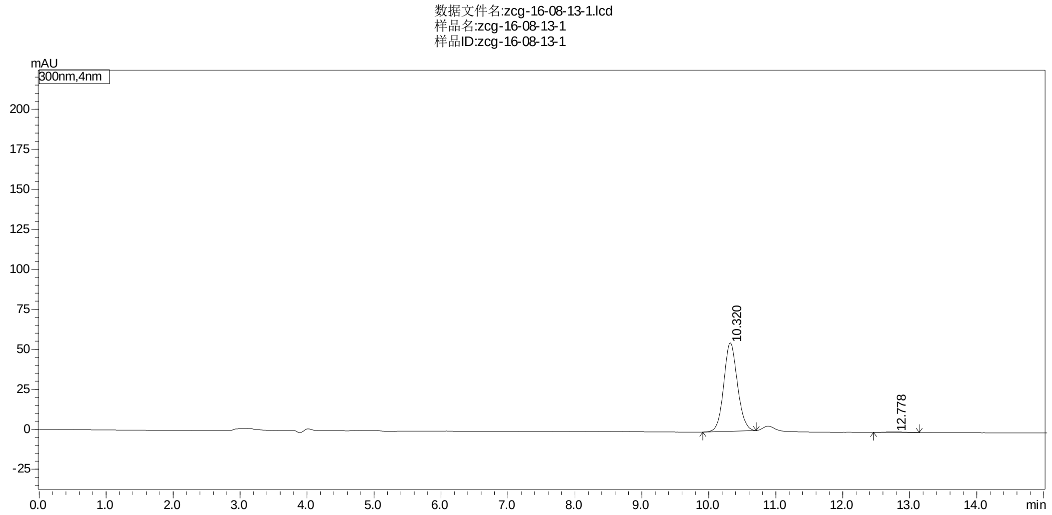

Peak#	Ret. Time	Area	Height	Area%	Height%
1	10.320	785169	55222	99.070	99.317
2	12.778	7372	380	0.930	0.683
Total		792541	55602	100.000	100.000

Supplementary Figure 116. ^{1}H NMR Spectrum of 4aa

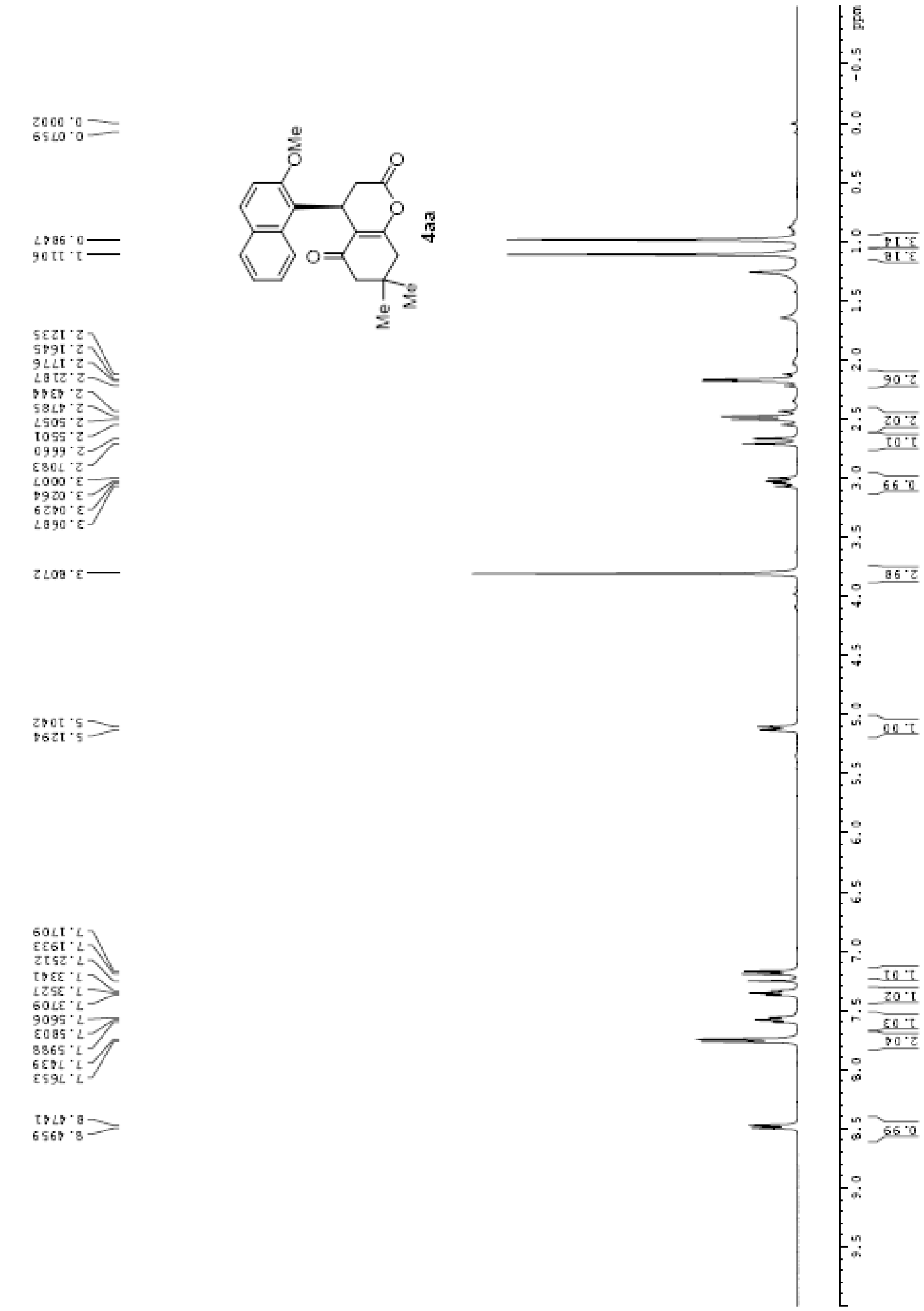

Supplementary Figure 117. ^{13}C NMR Spectrum of 4aa

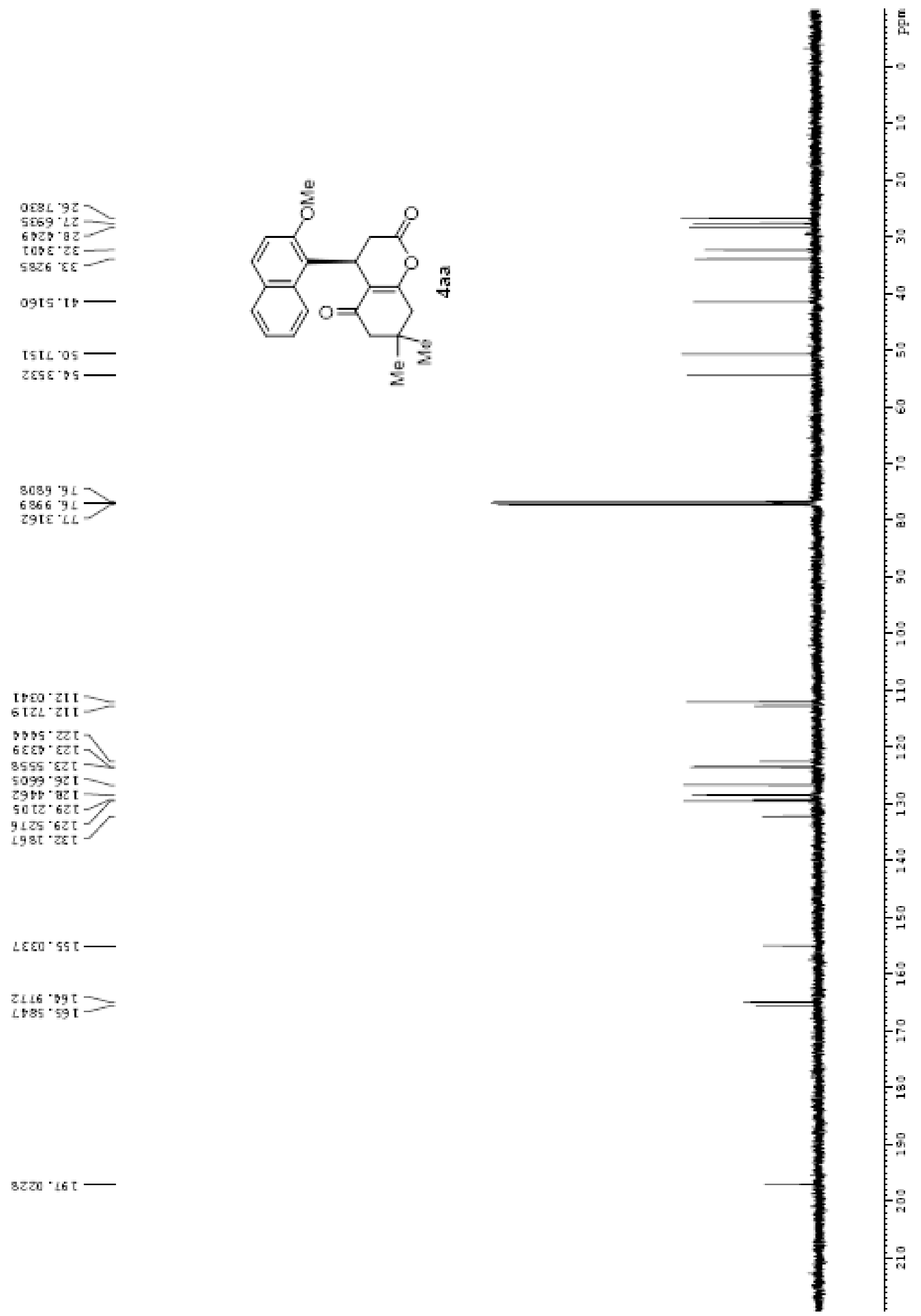

Supplementary Figure 118. ^{13}C NMR Spectrum of 4aa

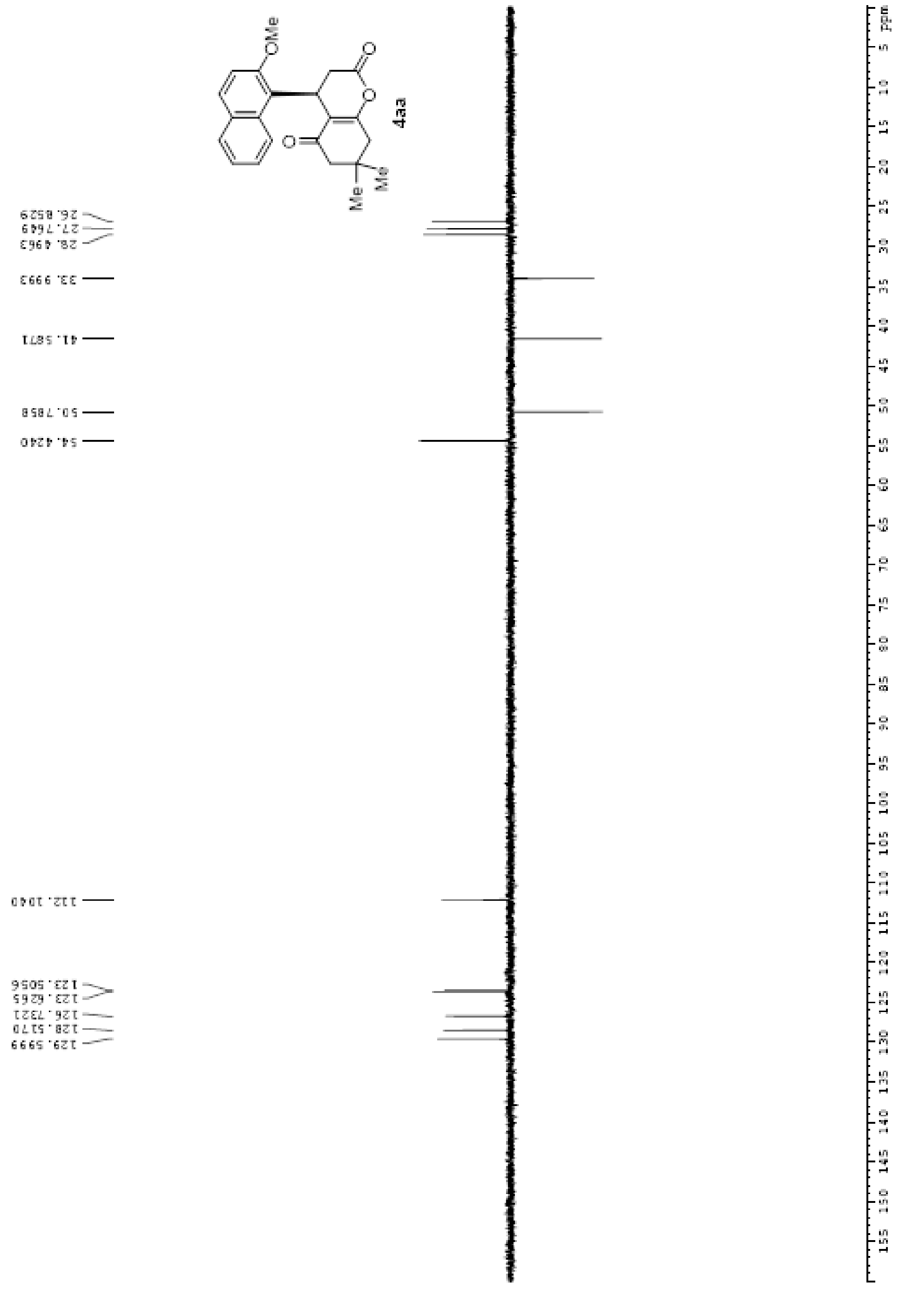

Supplementary Figure 119. HPLC Spectrum of racemic 4aa

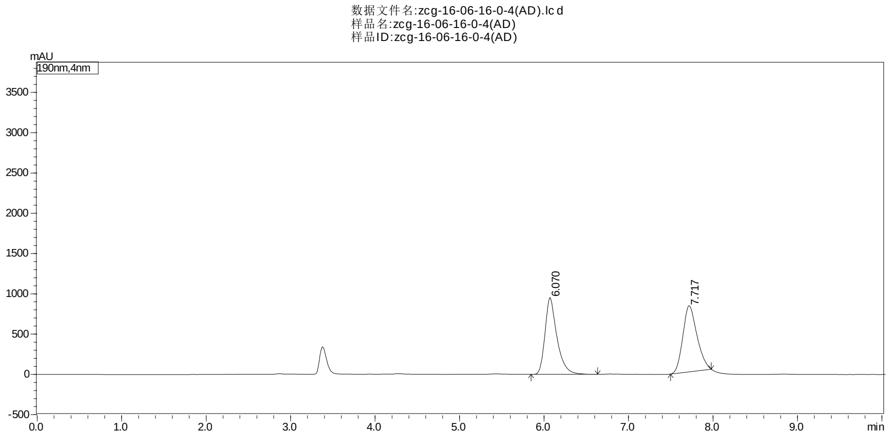

Peak#	Ret. Time	Area	Height	Area%	Height%
1	6.070	9315359	955058	49.373	53.837
2	7.717	9552141	818936	50.627	46.163
Total		18867500	1773995	100.000	100.000

Supplementary Figure 120. HPLC Spectrum of 4aa

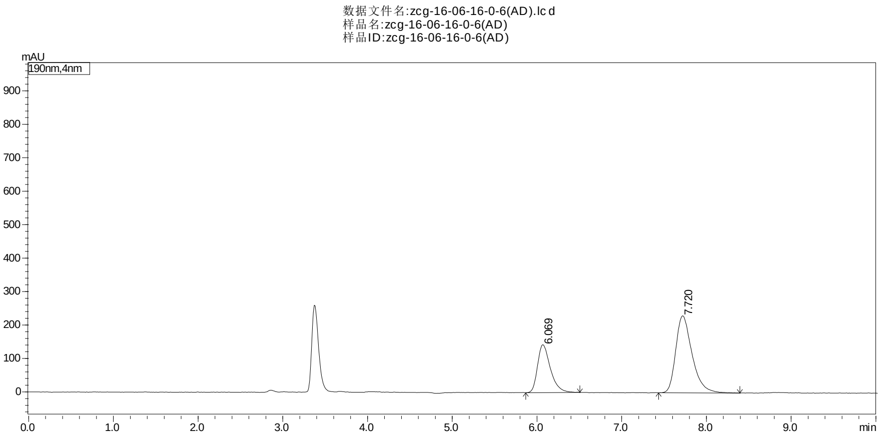

Peak#	Ret. Time	Area	Height	Area%	Height%
1	6.069	1516859	143429	32.858	38.353
2	7.720	3099564	230542	67.142	61.647
Total		4616423	373971	100.000	100.000

Supplementary Figure 121. ^{1}H NMR Spectrum of 11

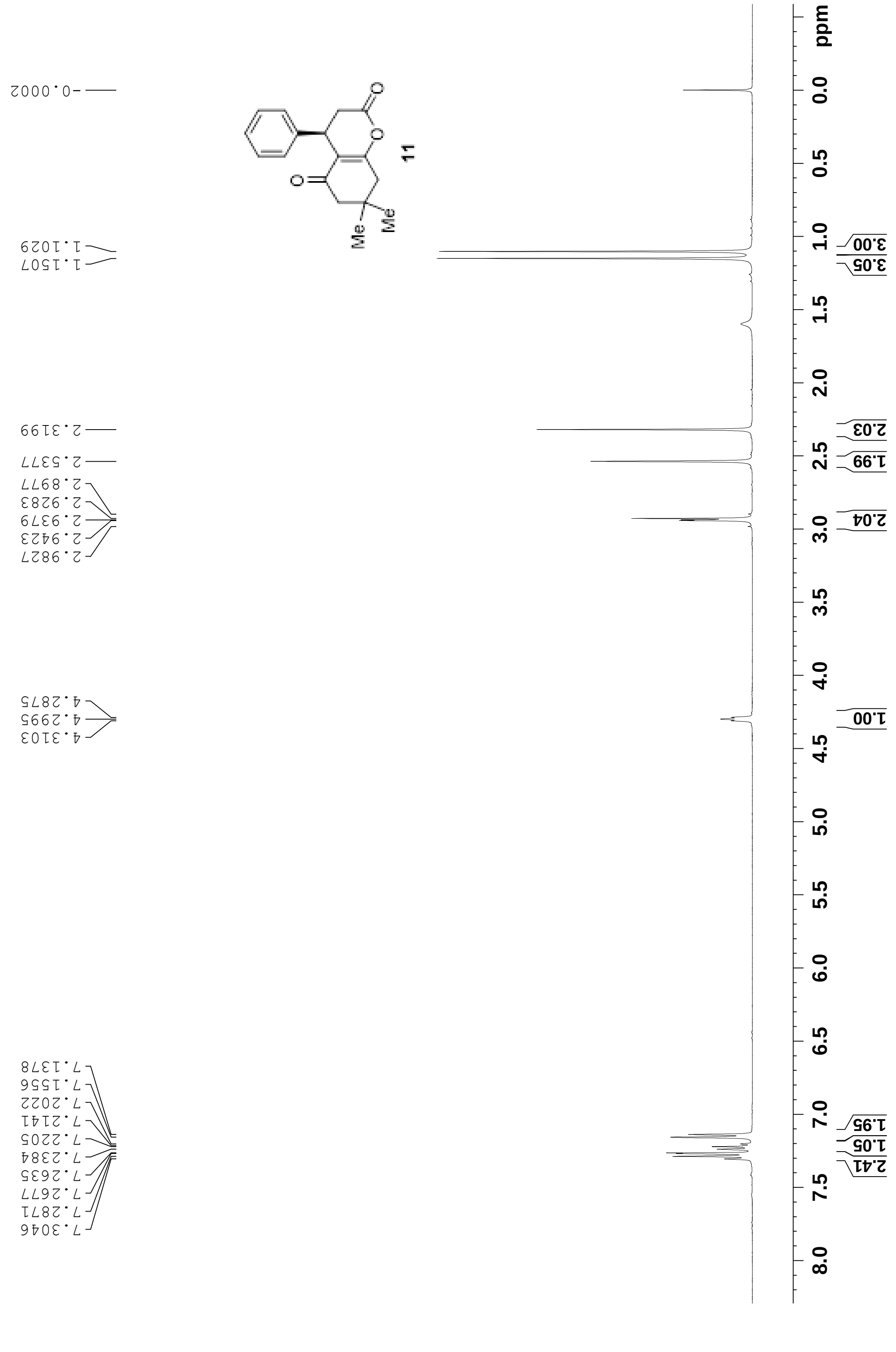

Supplementary Figure 122. ^{13}C NMR Spectrum of 11

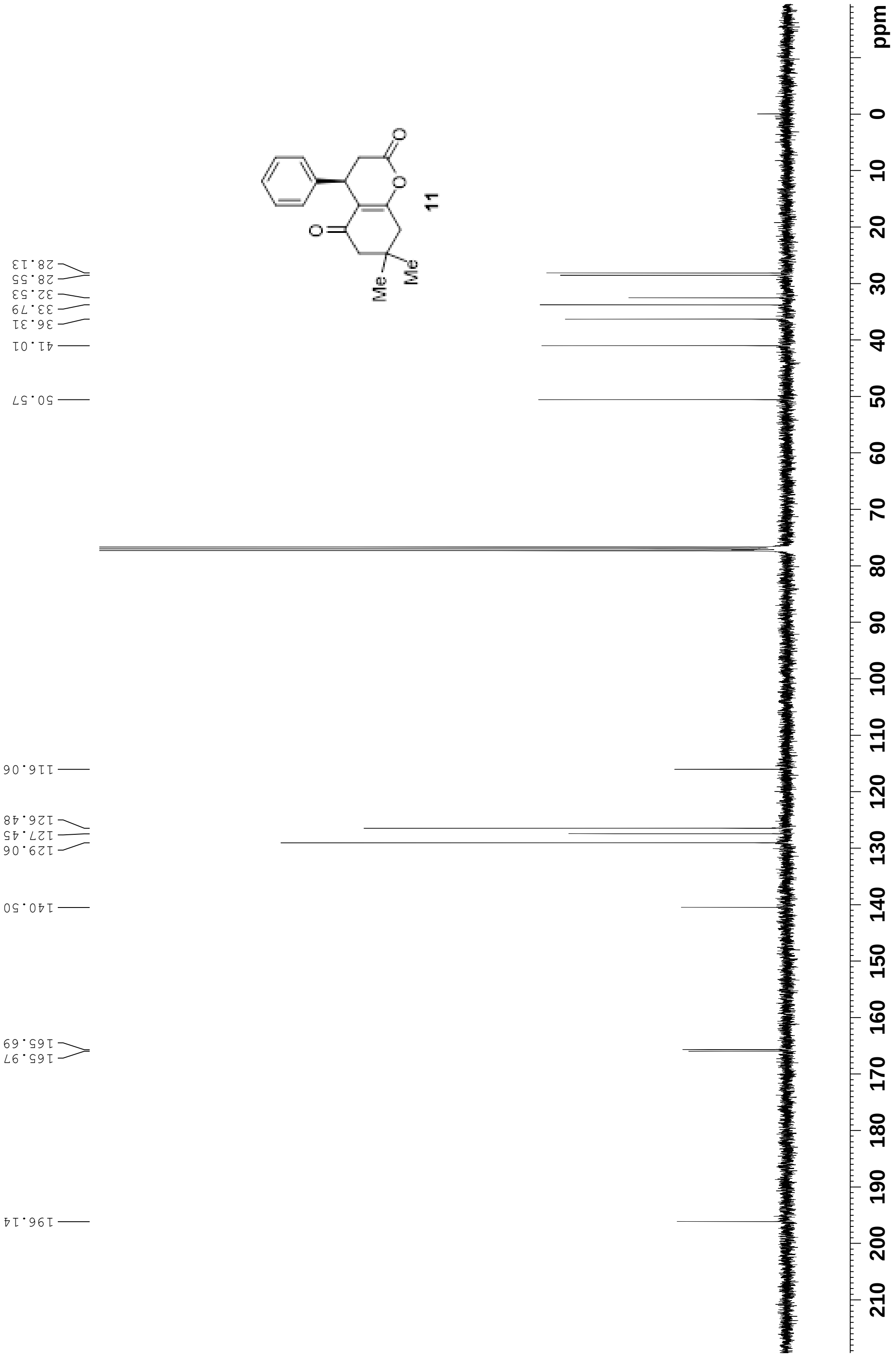

Supplementary Figure 123. HPLC Spectrum of racemic 11

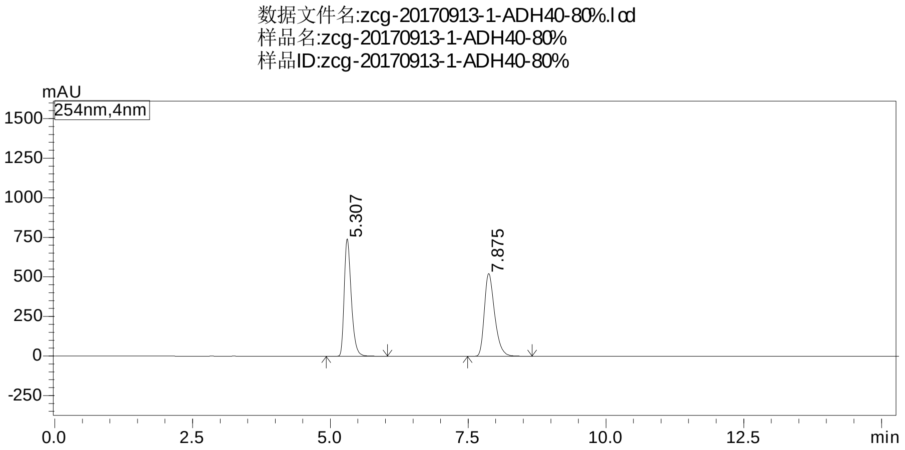

Peak#	Ret. Time	Area	Height	Area%	Height%
1	5.307	6572017	741111	49.280	58.635
2	7.875	6764154	522830	50.720	41.365
Total		13336171	1263942	100.000	100.000

Supplementary Figure 124. HPLC Spectrum of 11

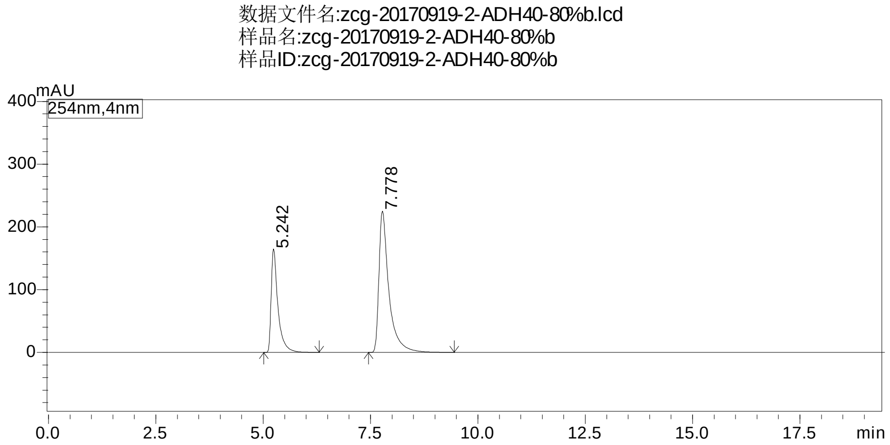

Peak#	Ret. Time	Area	Height	Area%	Height%
1	5.242	1812216	164708	33.294	42.258
2	7.778	3630915	225061	66.706	57.742
Total		5443130	389770	100.000	100.000

Supplementary Figure 125. ^{1}H NMR Spectrum of 5aa

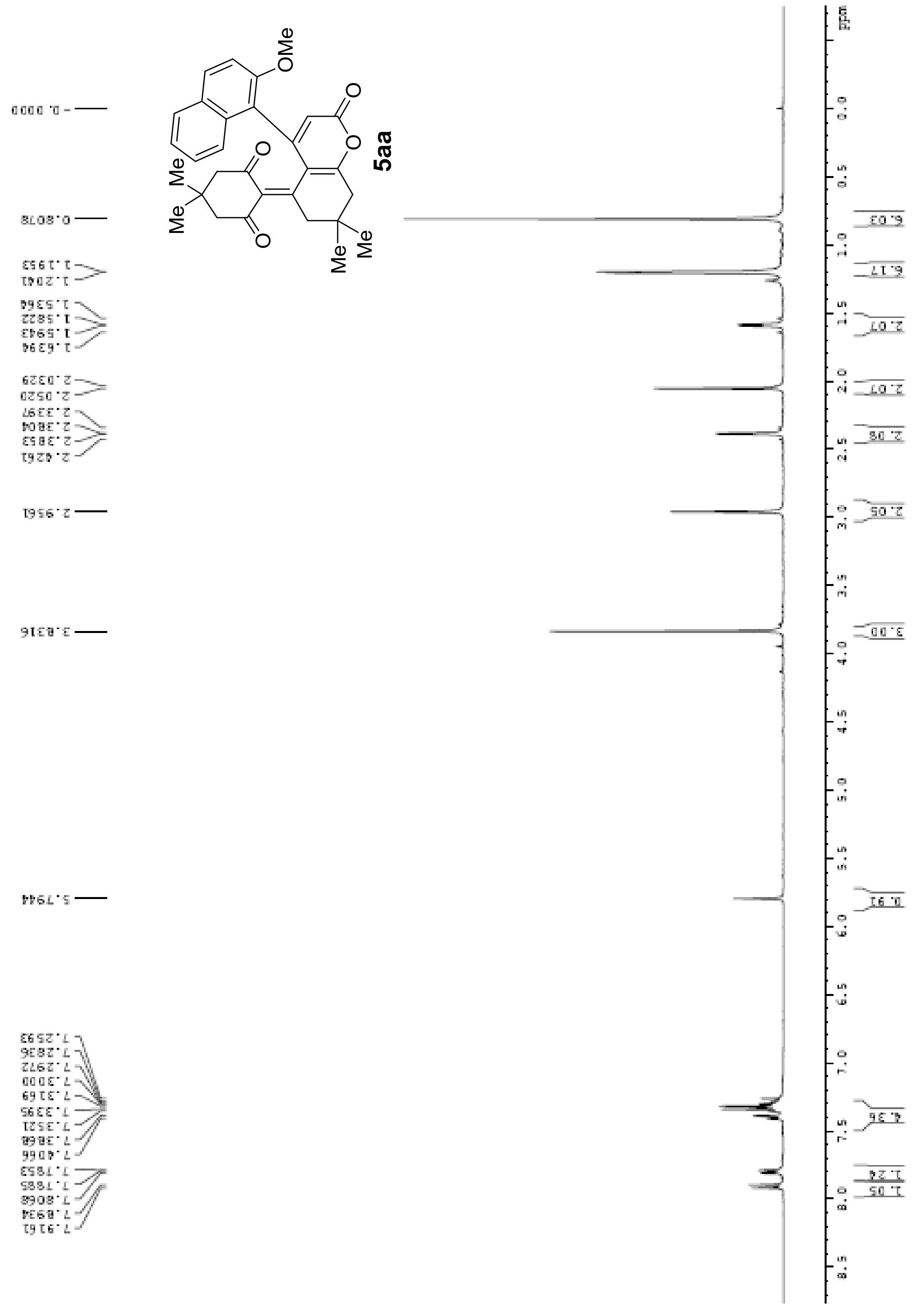

Supplementary Figure 126. ^{13}C NMR Spectrum of 5aa

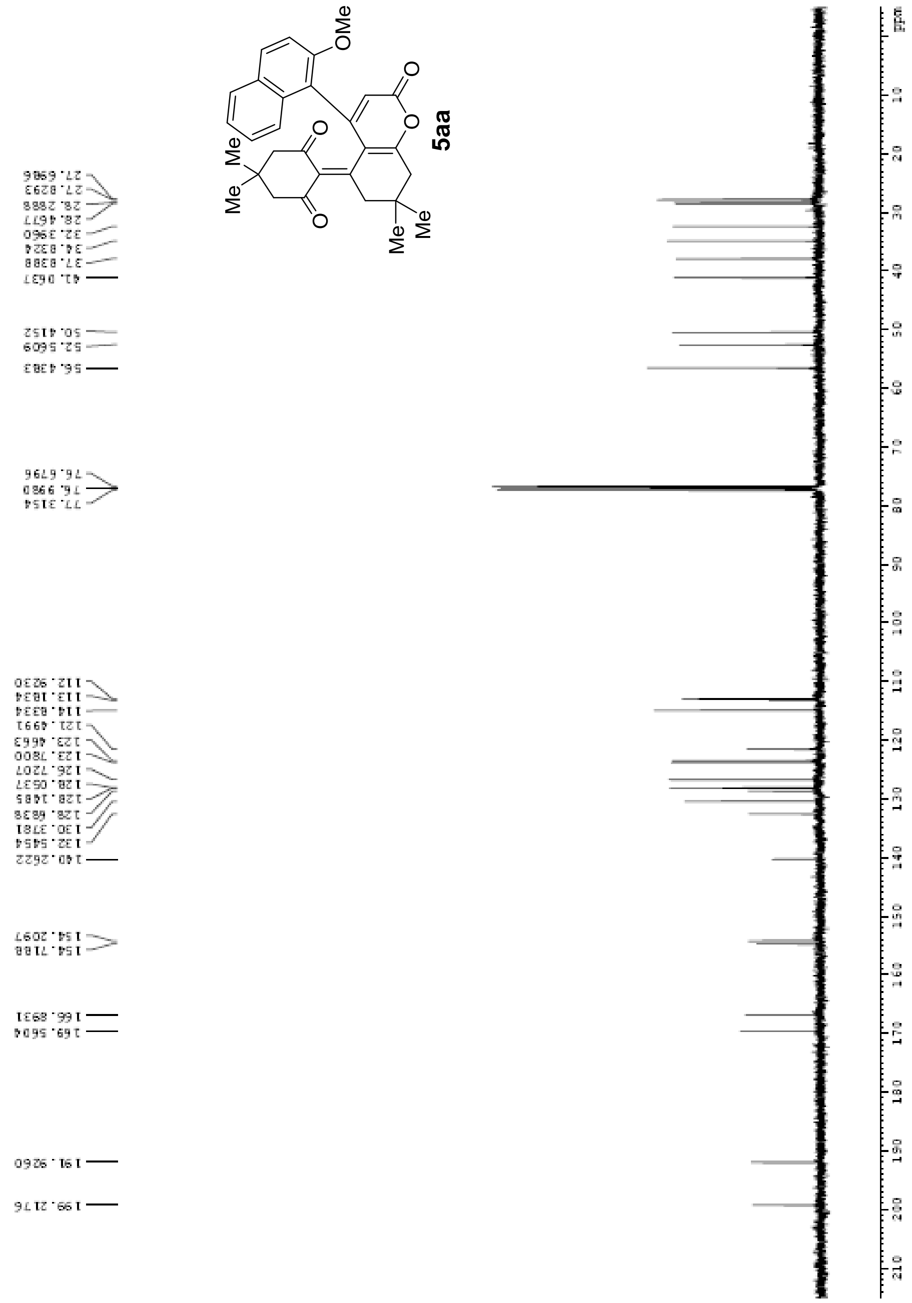

Supplementary Figure 127. ^{13}C NMR Spectrum of 5aa

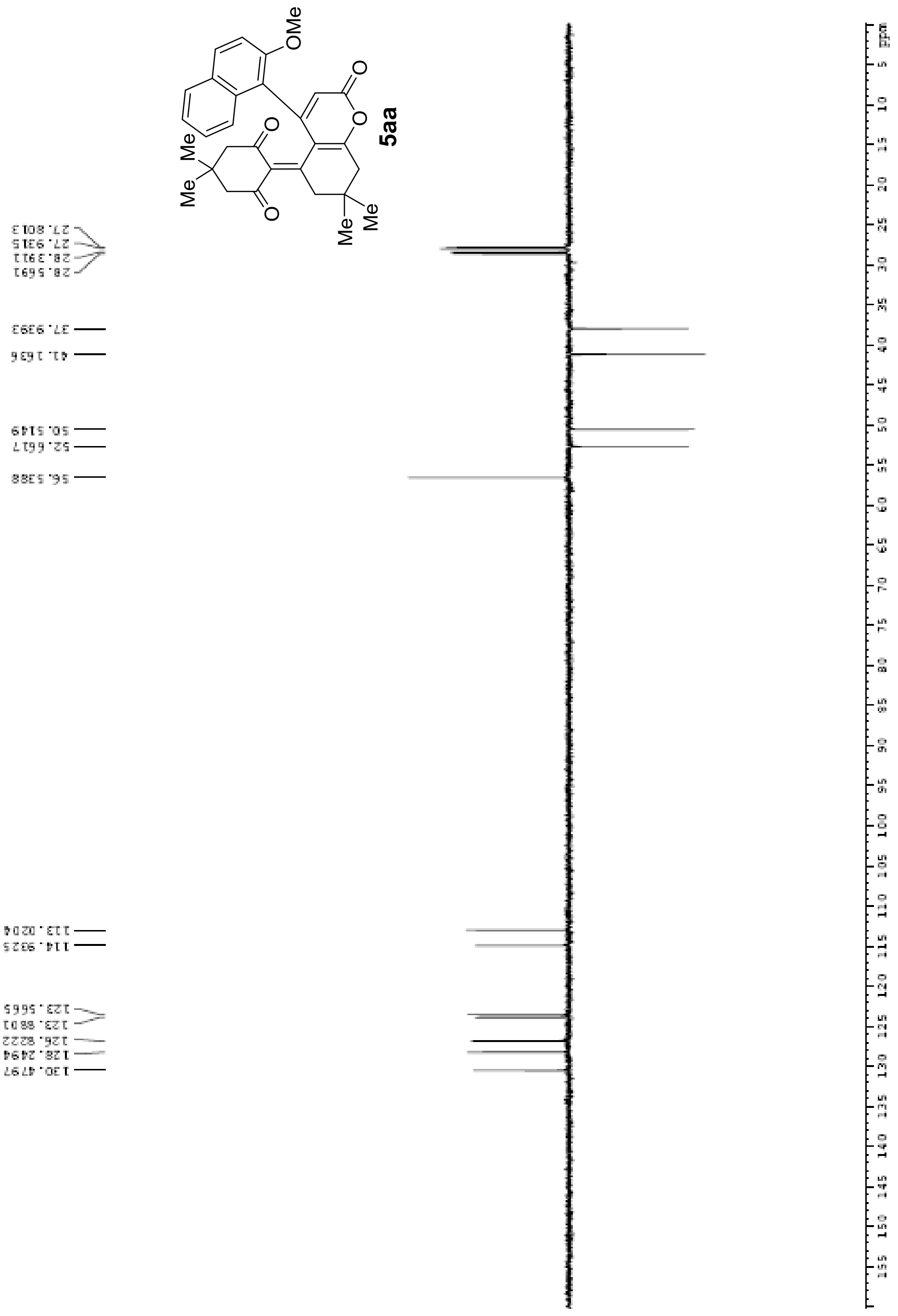

Supplementary Figure 128. ^{1}H NMR Spectrum of 6aa

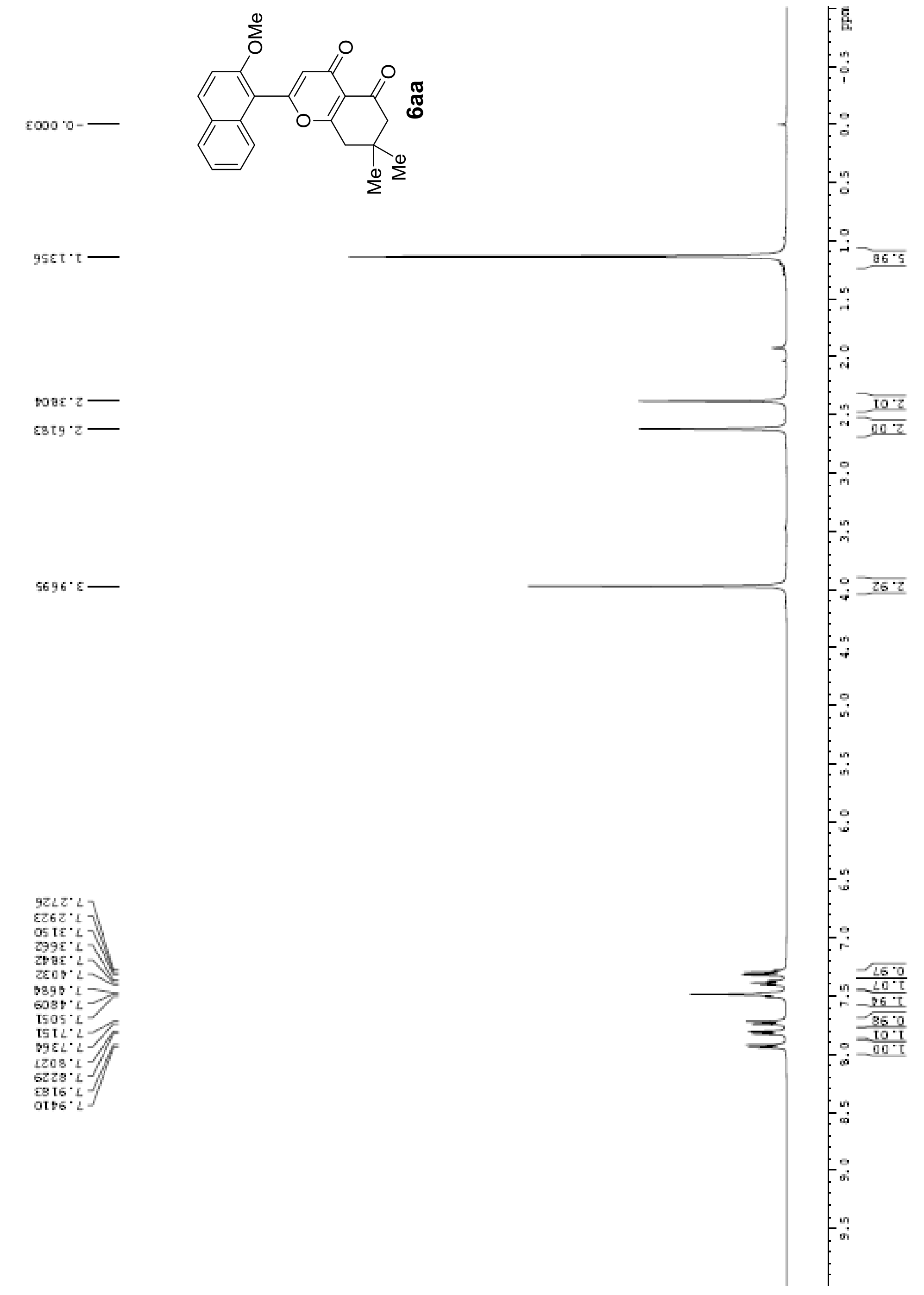

Supplementary Figure 129. ^{13}C NMR Spectrum of 6aa

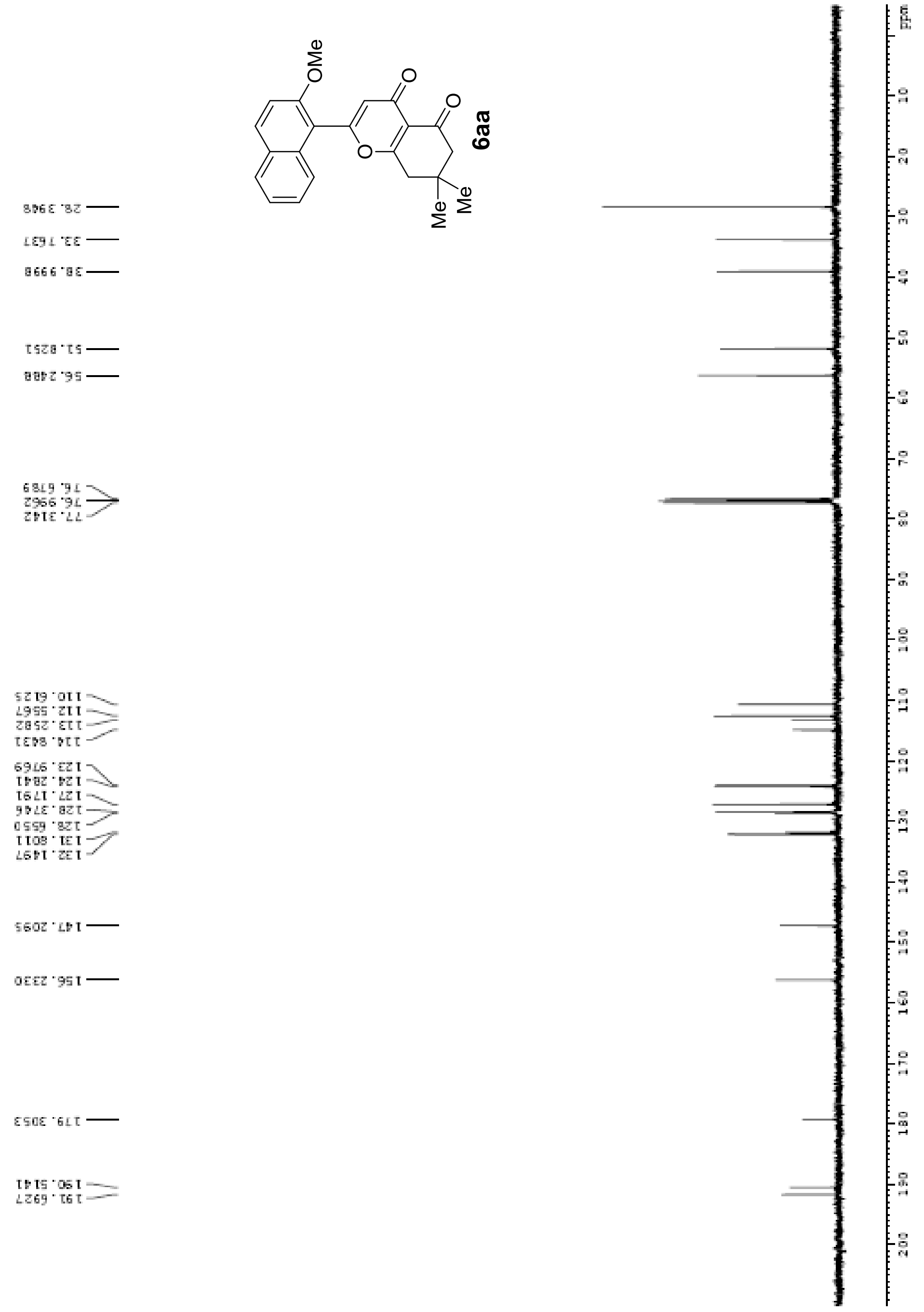

Supplementary Figure 130. ^{13}C NMR Spectrum of 6aa

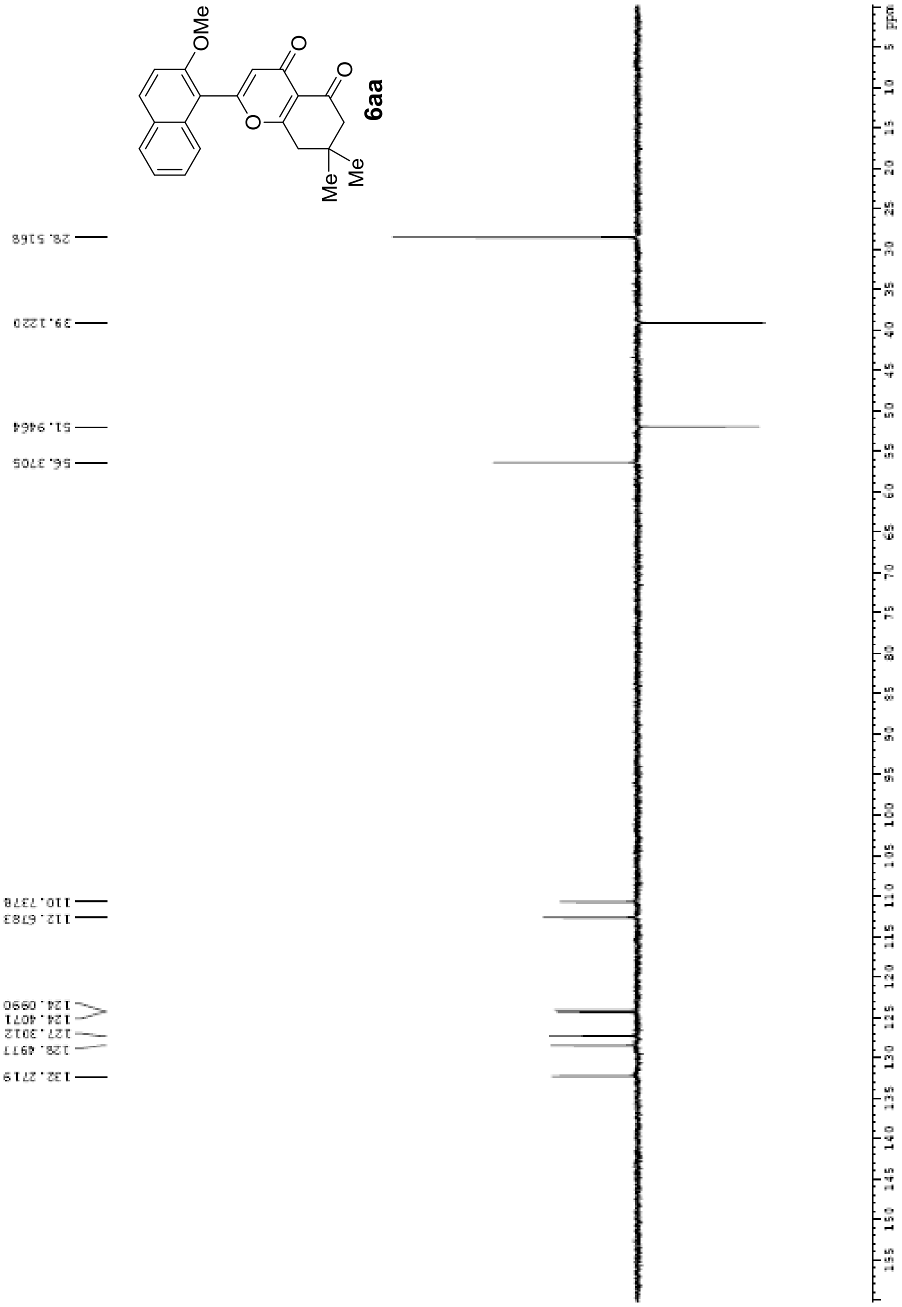

Supplementary Figure 131. ^{1}H NMR Spectrum of 7

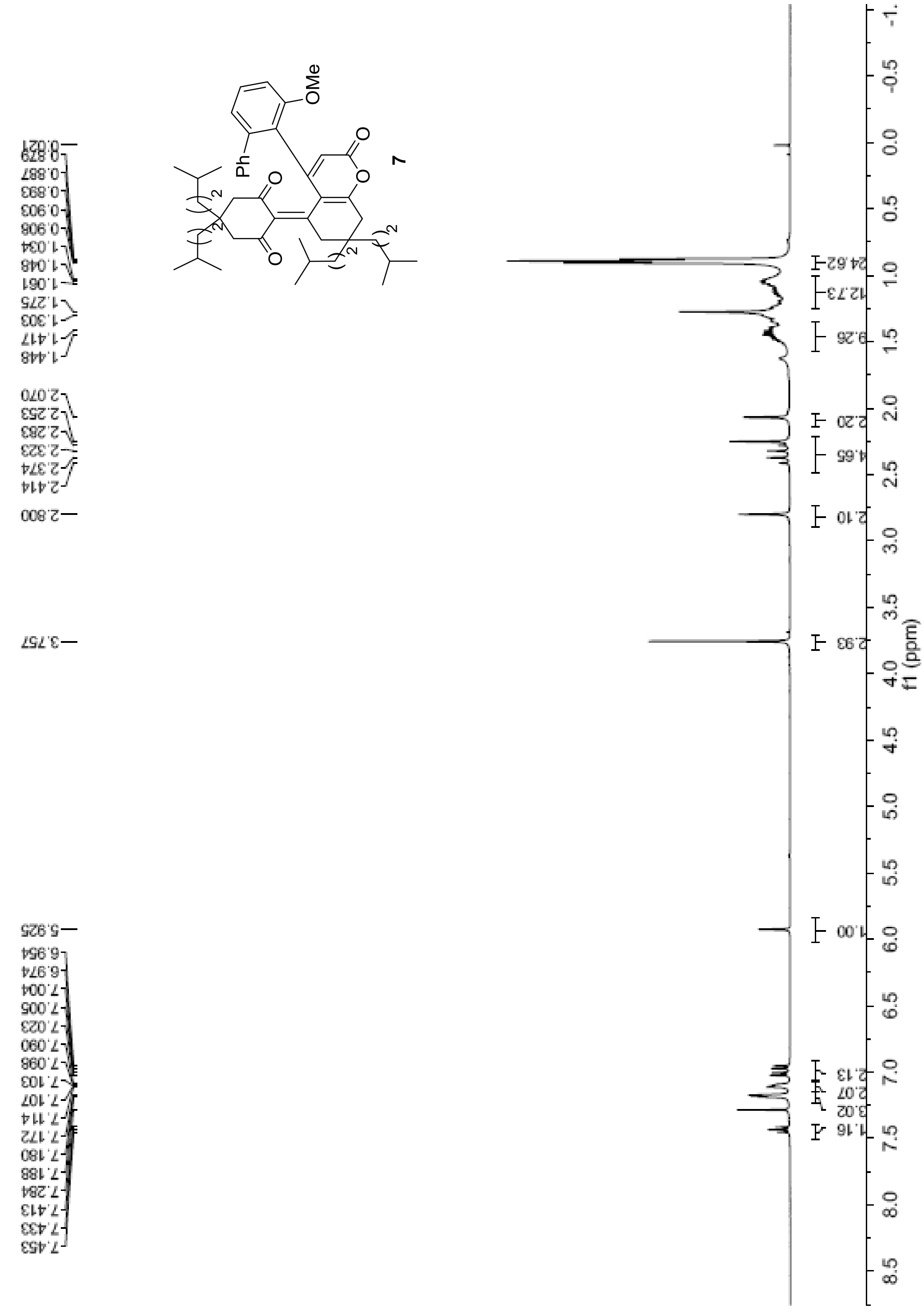

Supplementary Figure 132. ^{13}C NMR Spectrum of 7

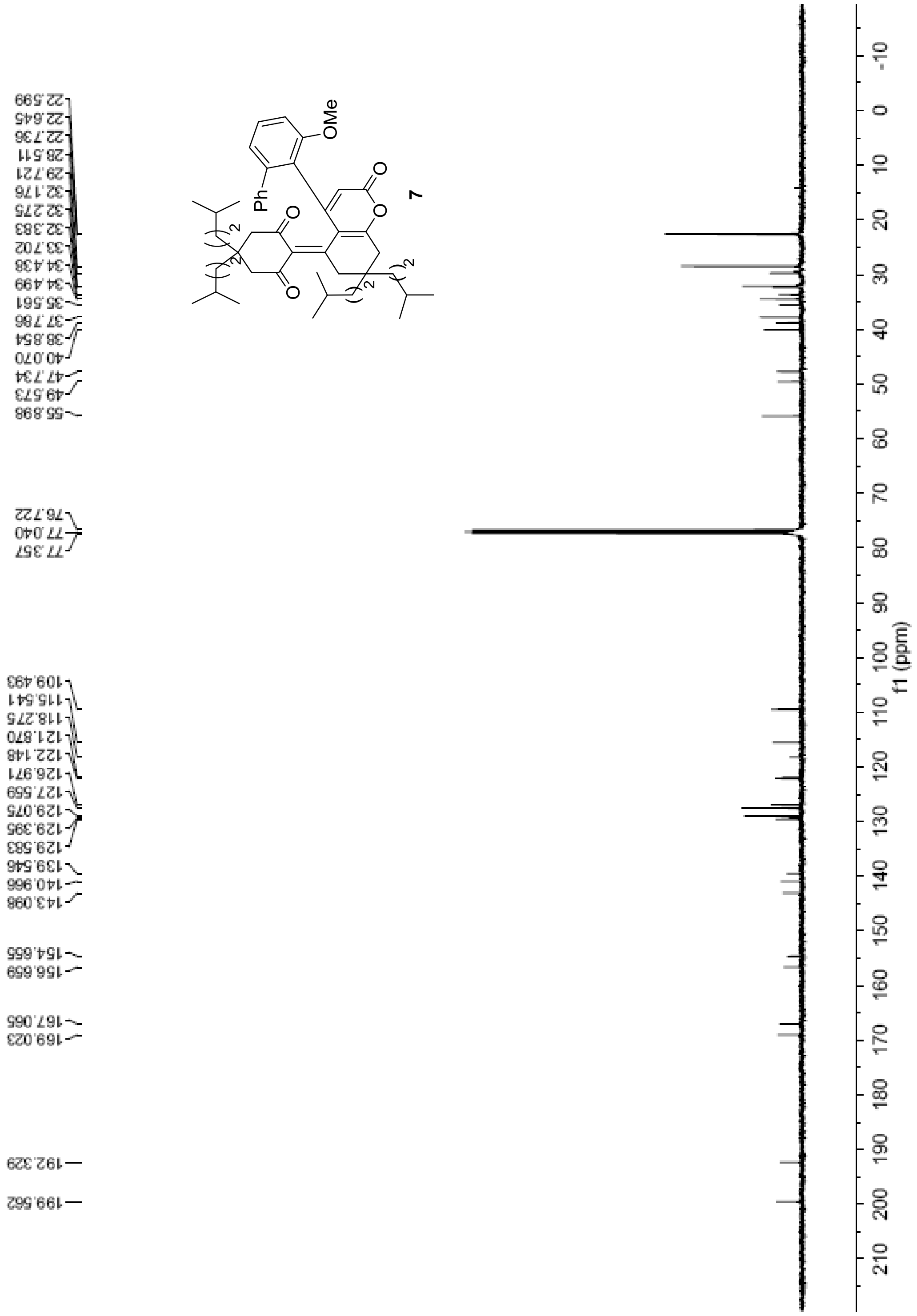

Supplementary Figure 133. ^{13}C NMR Spectrum of 7

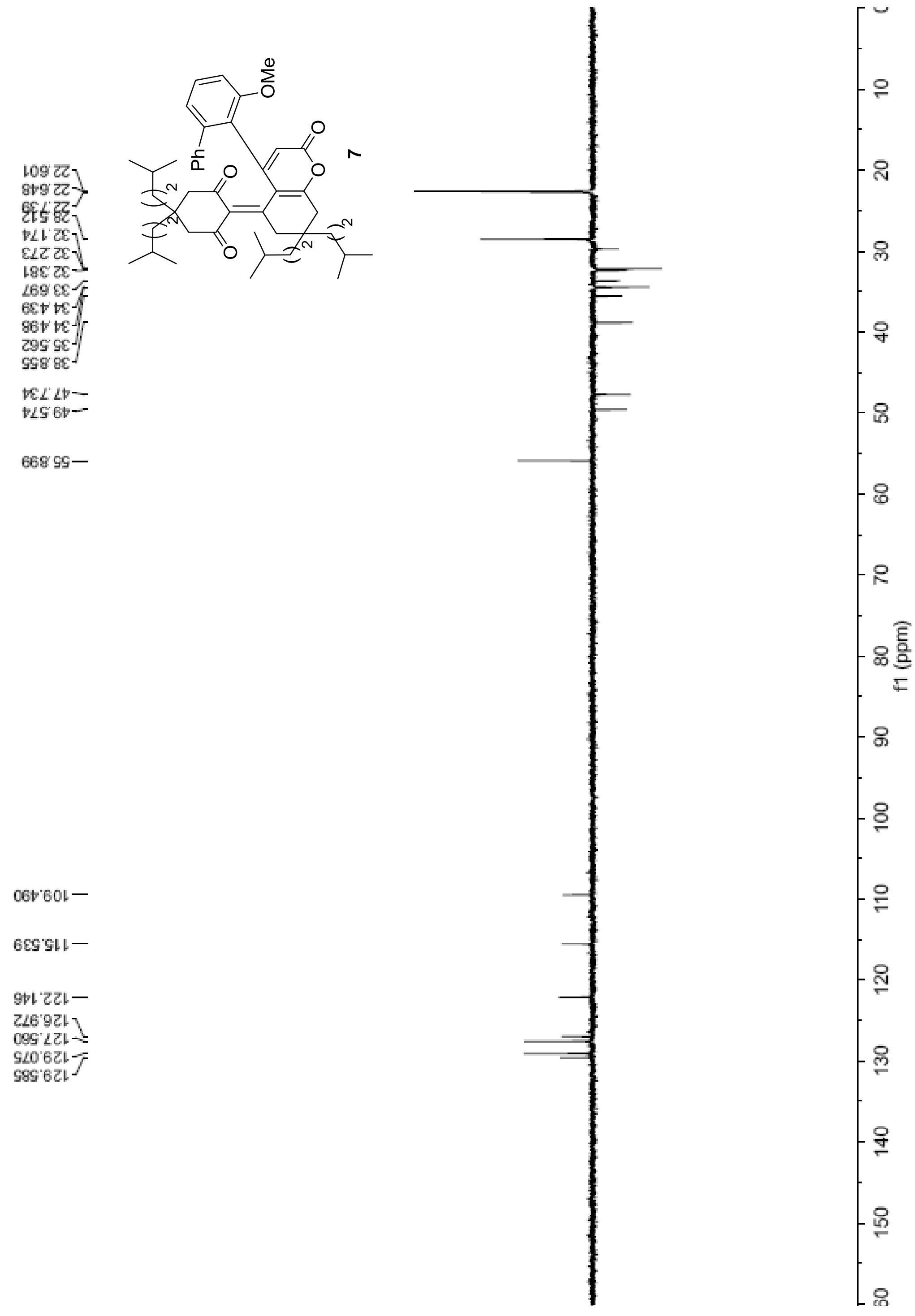

Supplementary Figure 134. HPLC Spectrum of racemic 7

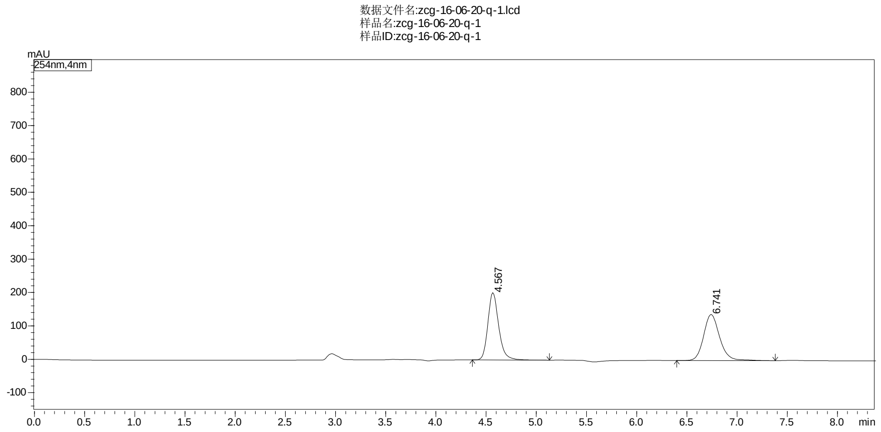

Peak#	Ret. Time	Area	Height	Area%	Height%
1	4.567	1518426	201541	49.658	59.301
2	6.741	1539320	138321	50.342	40.699
Total		3057746	339862	100.000	100.000

Supplementary Figure 135. HPLC Spectrum of 7

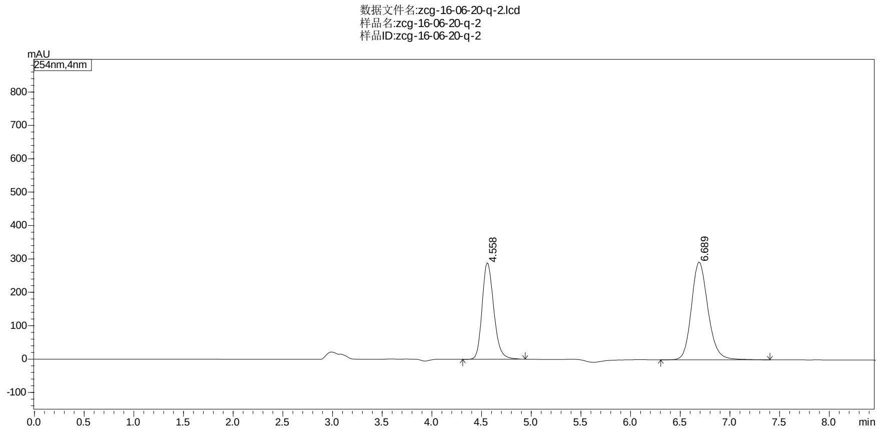

Peak#	Ret. Time	Area	Height	Area%	Height%
1	4.558	2362045	289010	40.993	49.678
2	6.689	3399994	292757	59.007	50.322
Total		5762040	581766	100.000	100.000

Supplementary Figure 136. ^{1}H NMR Spectrum of 8

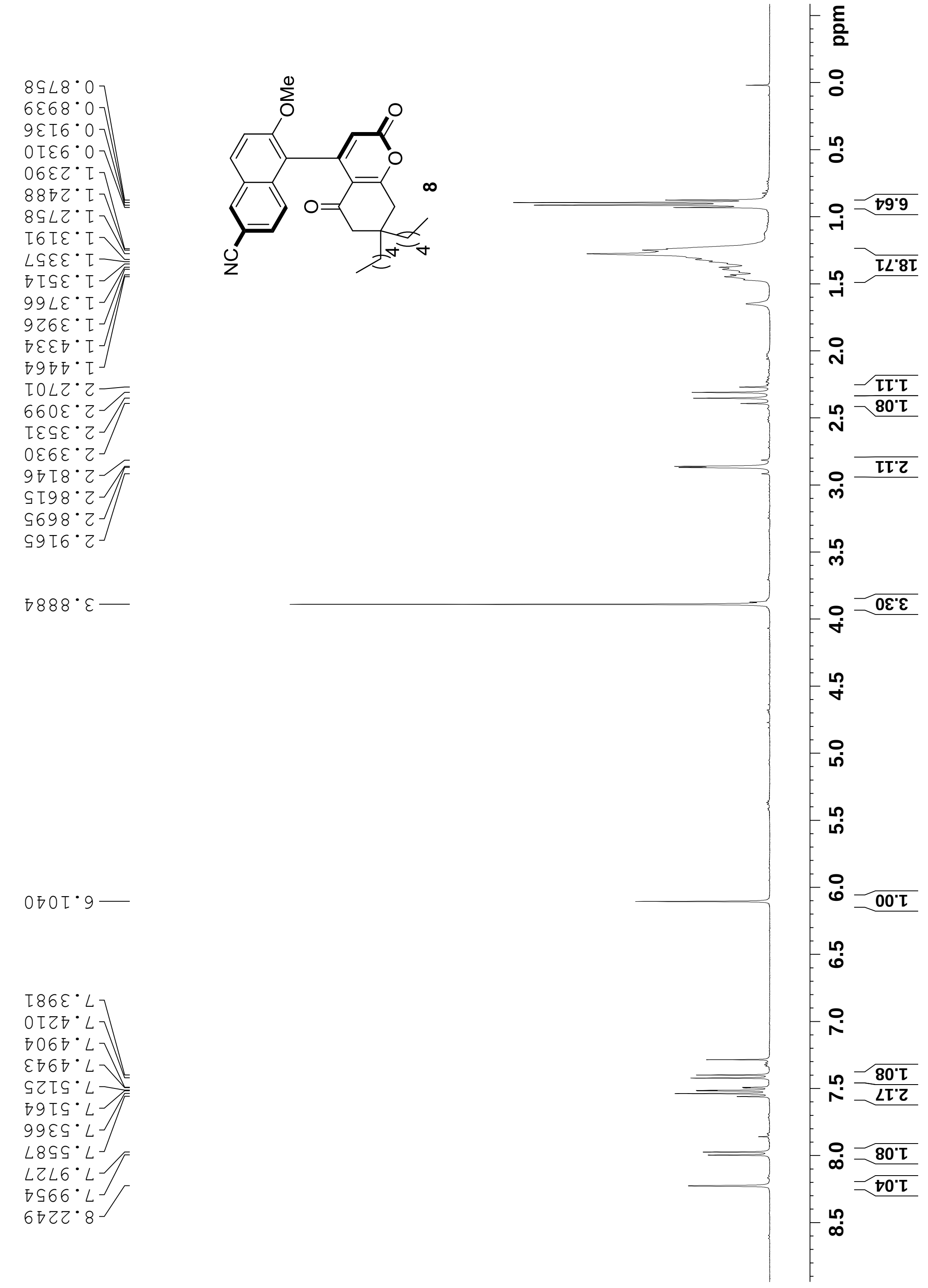

Supplementary Figure 137. ^{13}C NMR Spectrum of 8

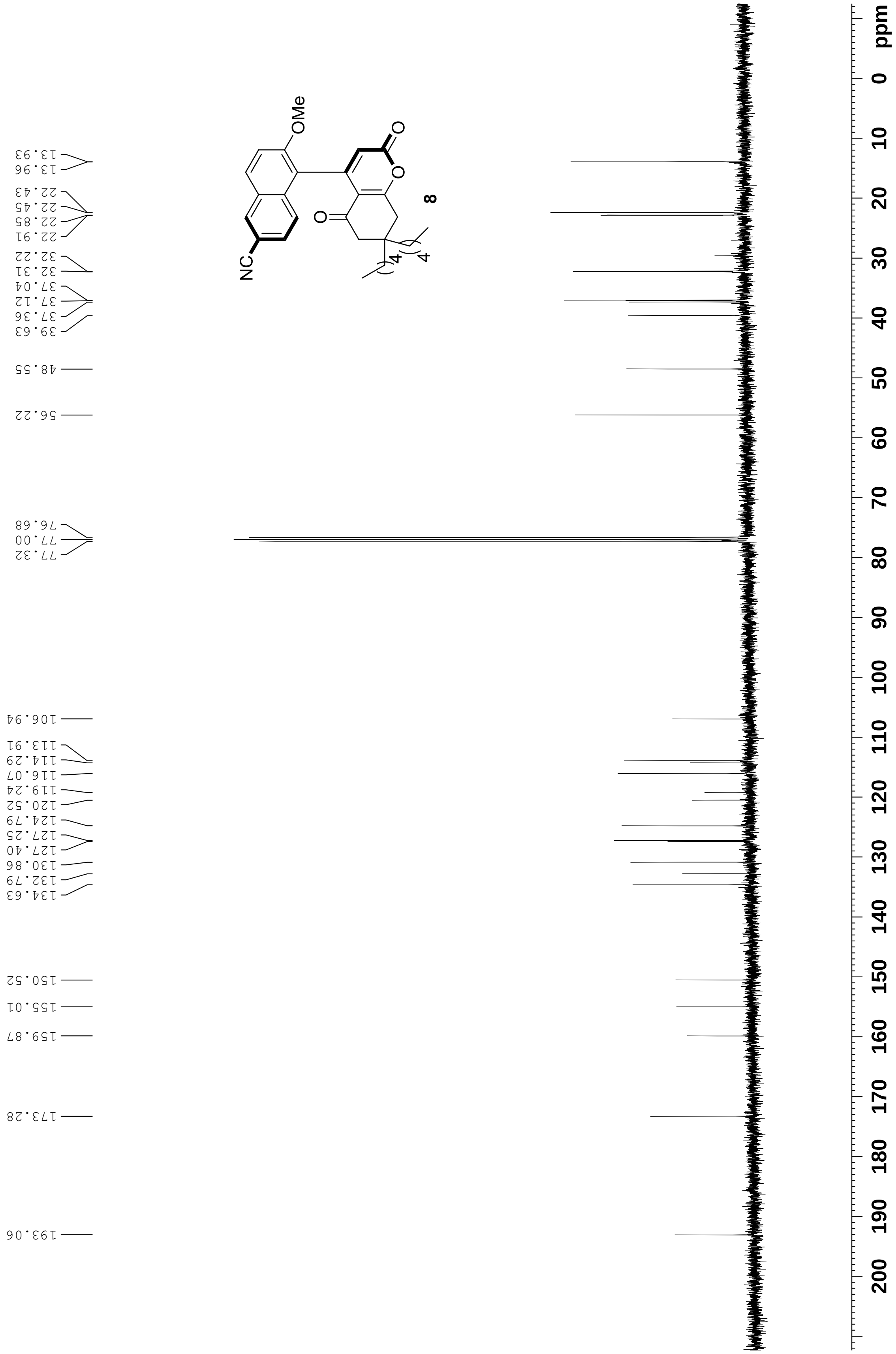

Supplementary Figure 138. HPLC Spectrum of racemic 8

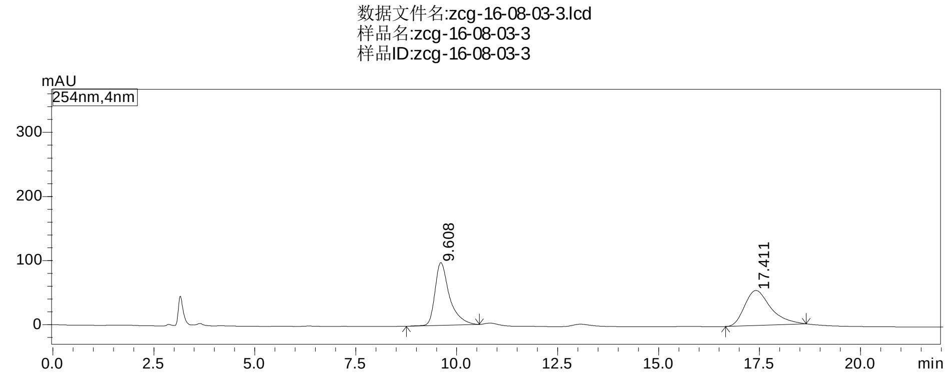

Peak#	Ret. Time	Area	Height	Area%	Height%
1	9.608	2446895	98076	49.825	64.197
2	17.411	2464079	54698	50.175	35.803
Total		4910973	152774	100.000	100.000

Supplementary Figure 139. HPLC Spectrum of 8

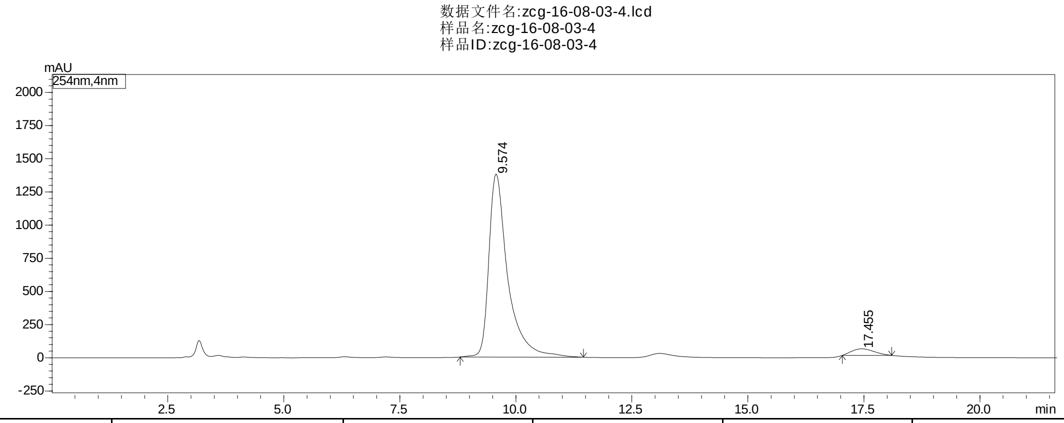

Peak#	Ret. Time	Area	Height	Area%	Height%
1	9.574	39228581	1378247	95.895	96.525
2	17.455	1679209	49617	4.105	3.475
Total		40907791	1427863	100.000	100.000

Supplementary Figure 140. ^{1}H NMR Spectrum of 10a

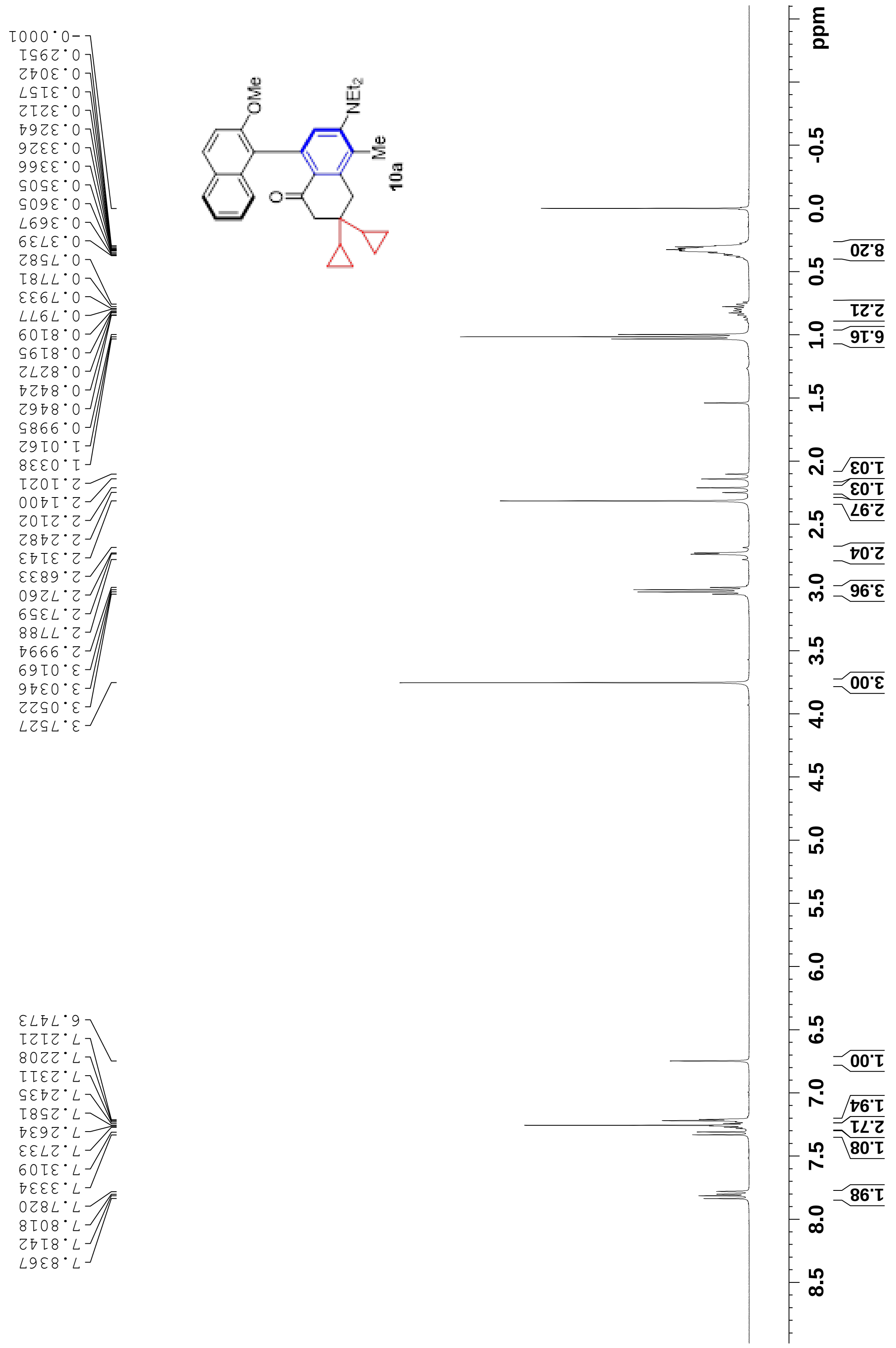

Supplementary Figure 141. ^{13}C NMR Spectrum of 10a

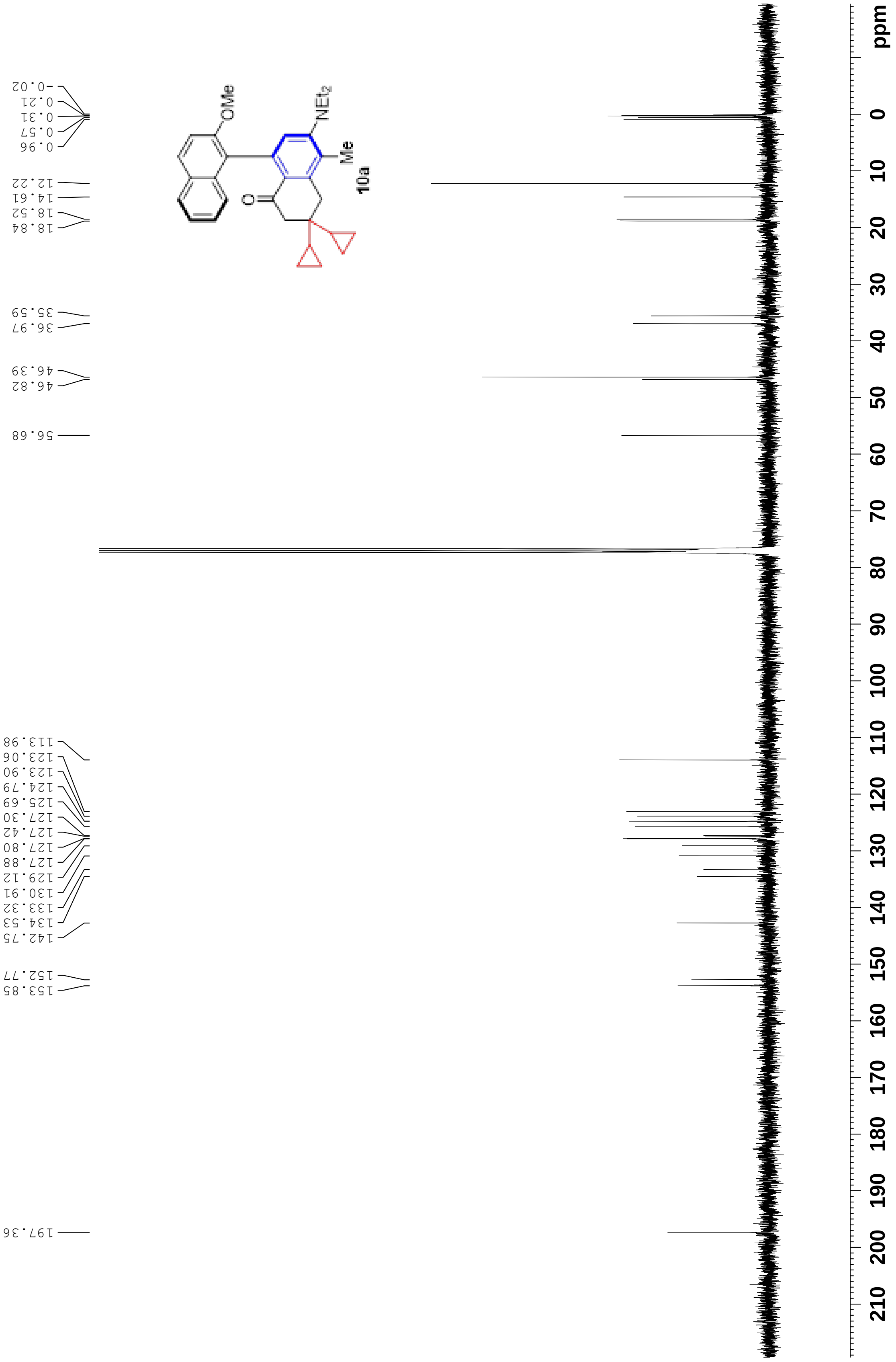

Supplementary Figure 142. HPLC Spectrum of racemic 10a

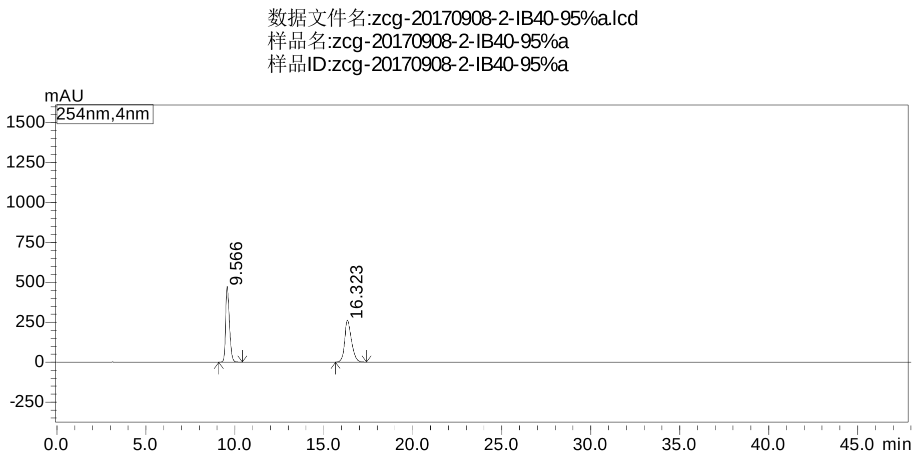

Peak#	Ret. Time	Area	Height	Area%	Height%
1	9.566	7000121	473590	50.084	64.367
2	16.323	6976682	262176	49.916	35.633
Total		13976803	735766	100.000	100.000

Supplementary Figure 143. HPLC Spectrum of 10a

数据文件名:zcg-20170922-1-IB40-95%.lcd
样品名:zcg-20170922-1-IB40-95%
样品ID:zcg-20170922-1-IB40-95%

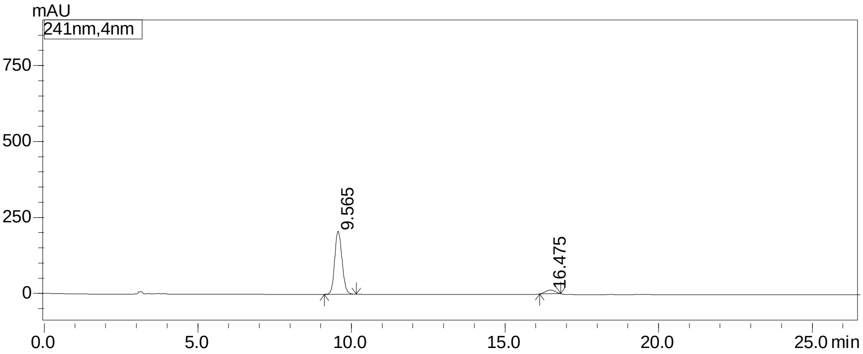

Peak#	Ret. Time	Area	Height	Area%	Height%
1	9.565	3456980	207485	92.694	94.307
2	16.475	272463	12525	7.306	5.693
Total		3729443	220010	100.000	100.000

Supplementary Figure 144. ^{1}H NMR Spectrum of 10b

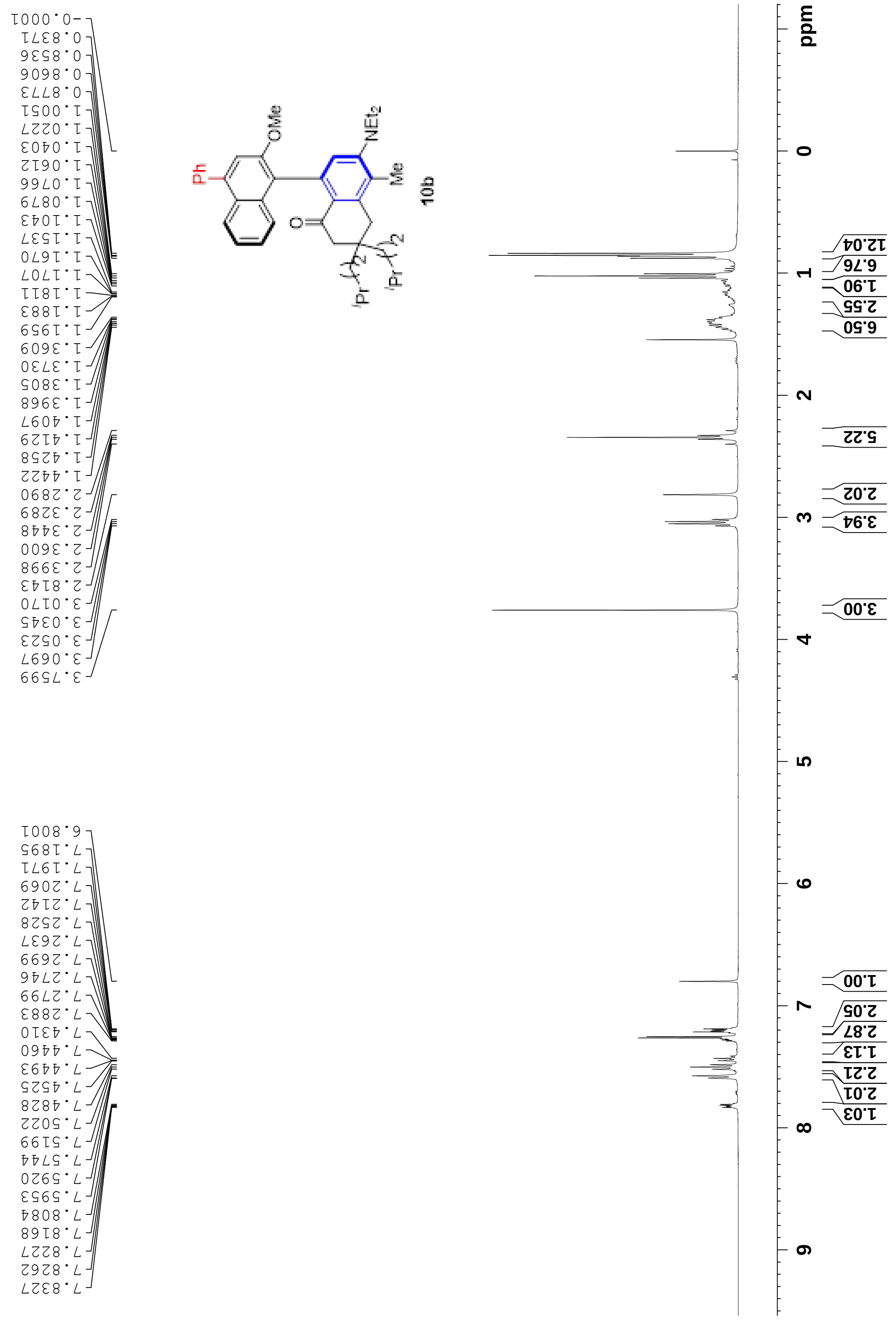

Supplementary Figure 145. ^{13}C NMR Spectrum of 10b

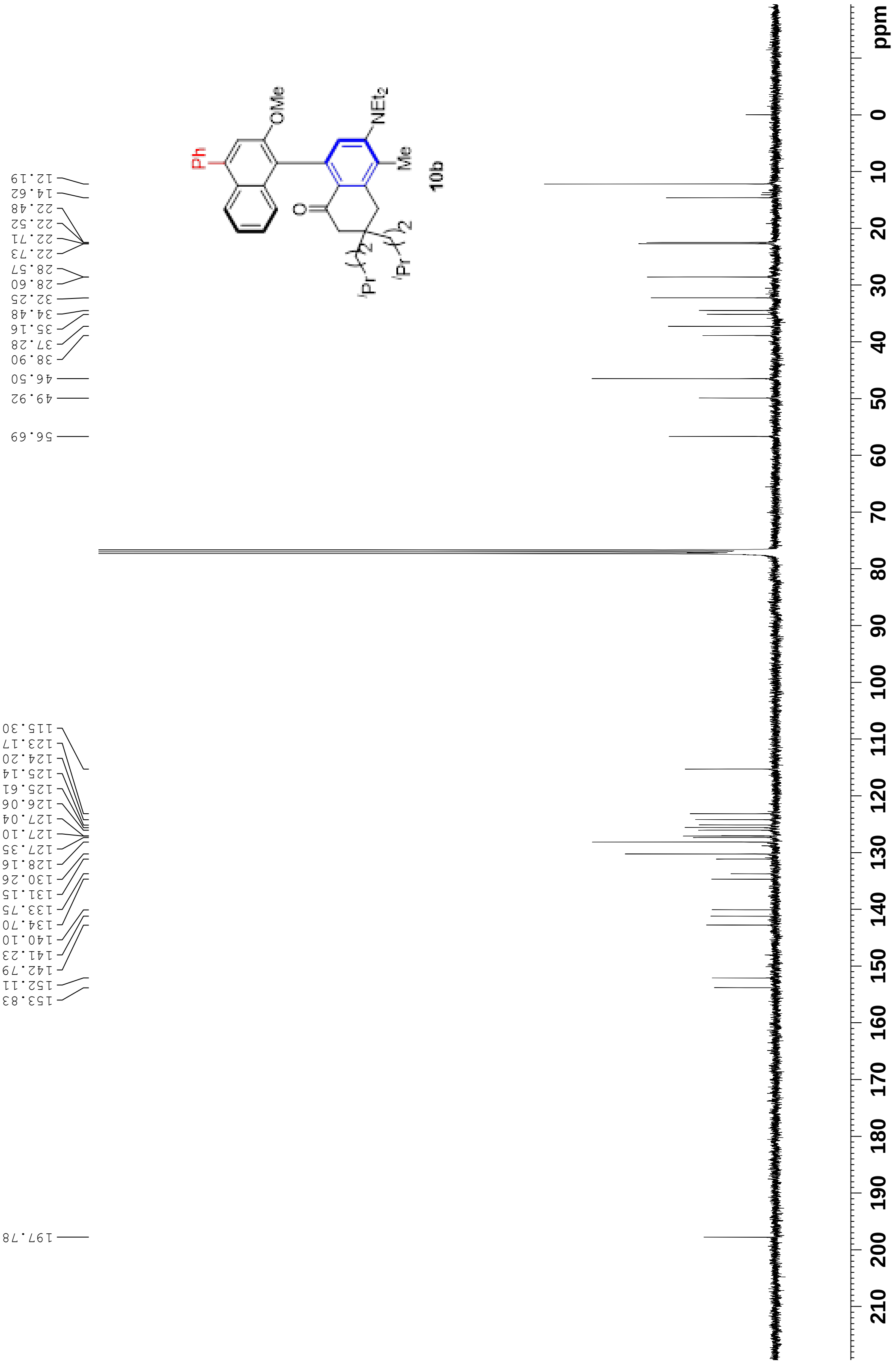

Supplementary Figure 146. ^{13}C NMR Spectrum of 10b

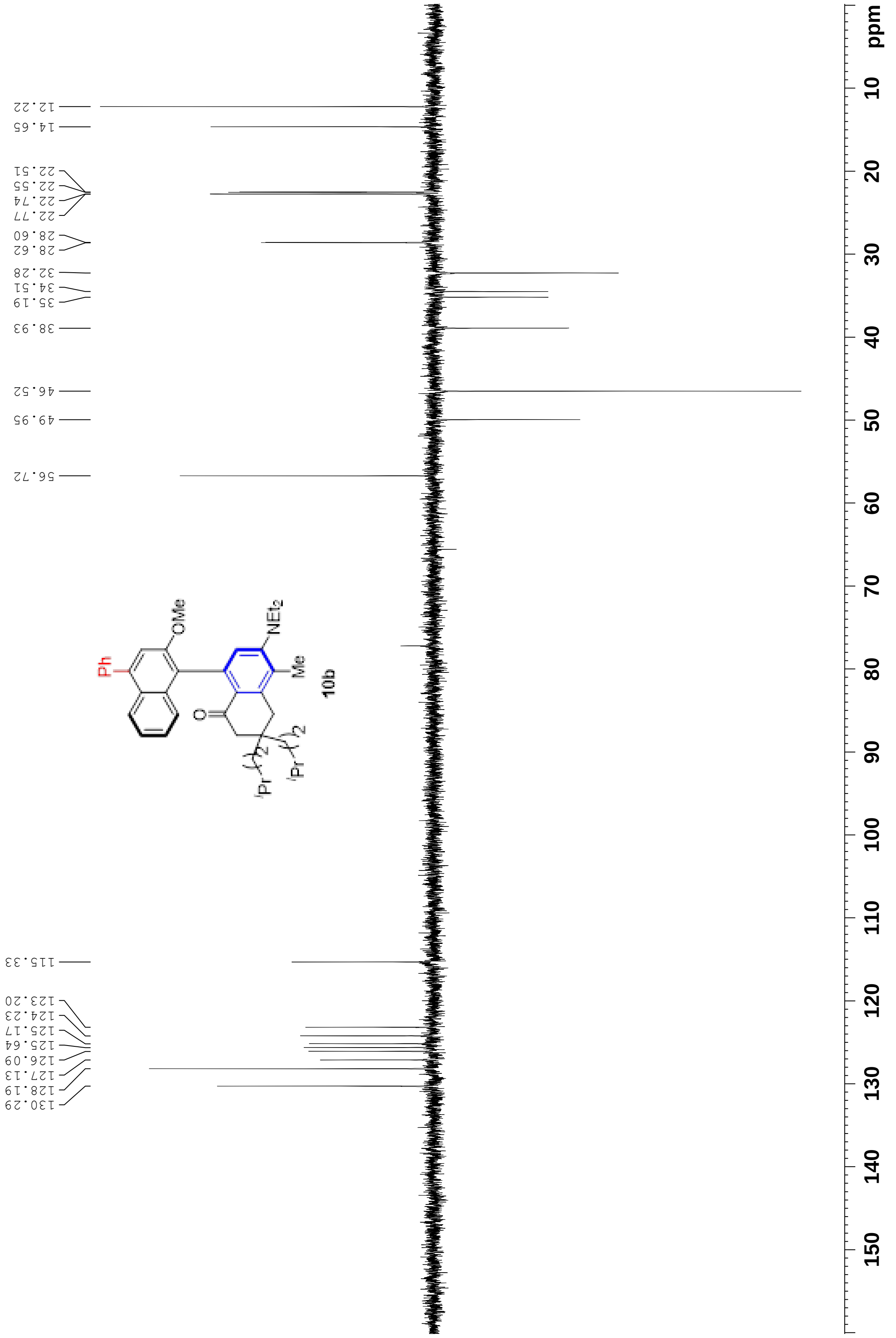

Supplementary Figure 147. HPLC Spectrum of racemic 10b

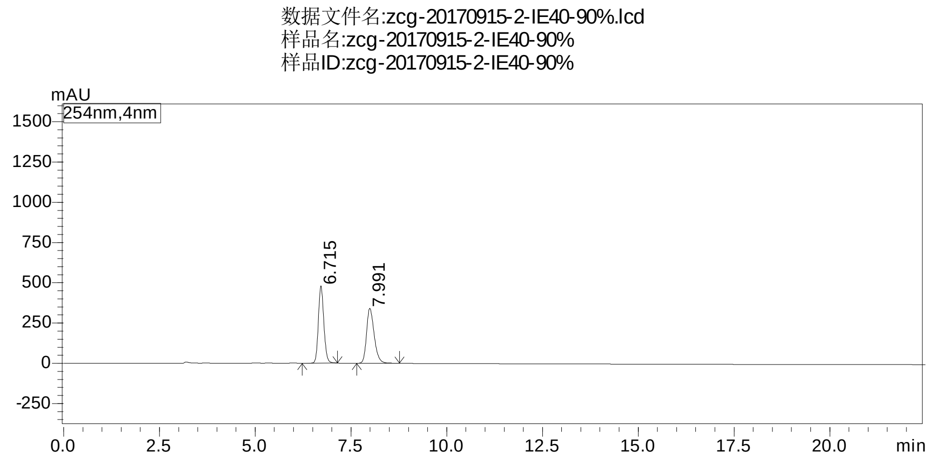

Peak#	Ret. Time	Area	Height	Area%	Height%
1	6.715	4767825	478543	50.117	58.330
2	7.991	4745523	341863	49.883	41.670
Total		9513349	820406	100.000	100.000

Supplementary Figure 148. HPLC Spectrum of 10b

数据文件名:zcg-20170915-2a-IE40-90%.lcd
样品名:zcg-20170915-2a-IE40-90%
样品ID:zcg-20170915-2a-IE40-90%

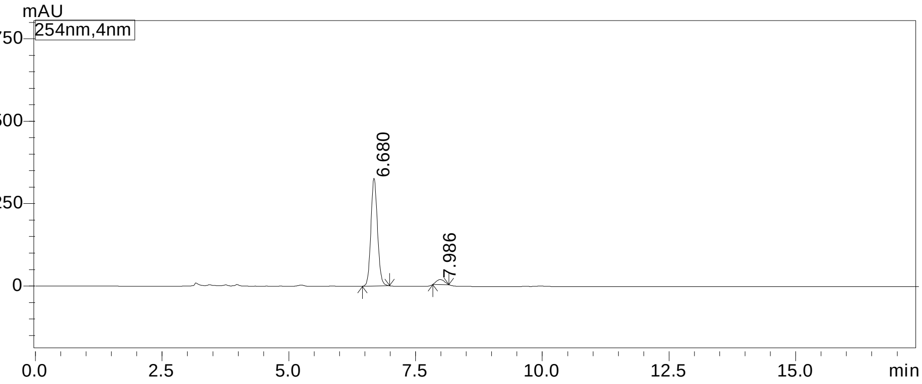

Peak#	Ret. Time	Area	Height	Area%	Height%
1	6.680	2956597	326990	94.708	95.591
2	7.986	165196	15082	5.292	4.409
Total		3121794	342072	100.000	100.000

Supplementary Figure 149. ^{1}H NMR Spectrum of 10c

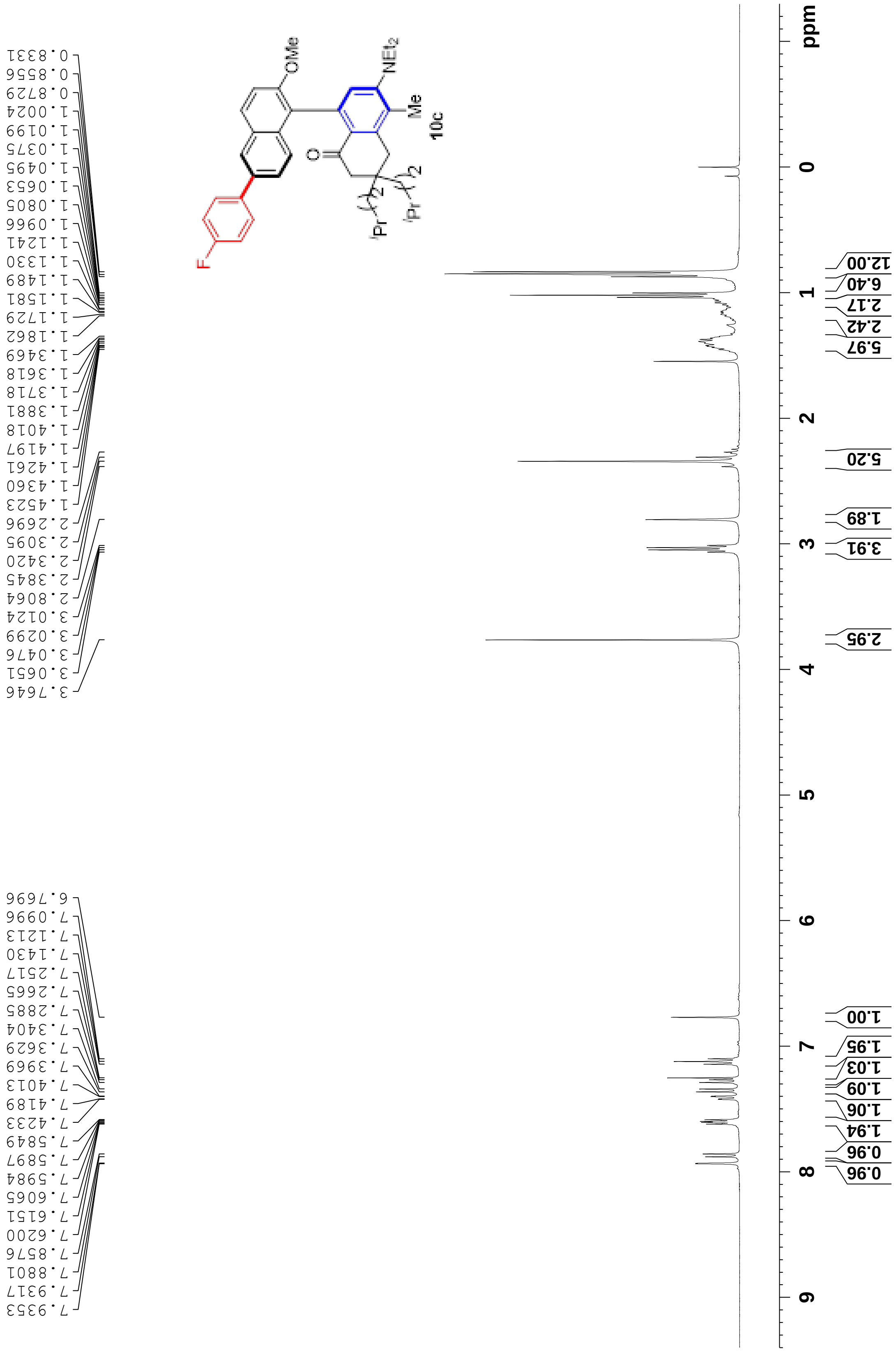

Supplementary Figure 150. ^{19}F NMR Spectrum of 10c

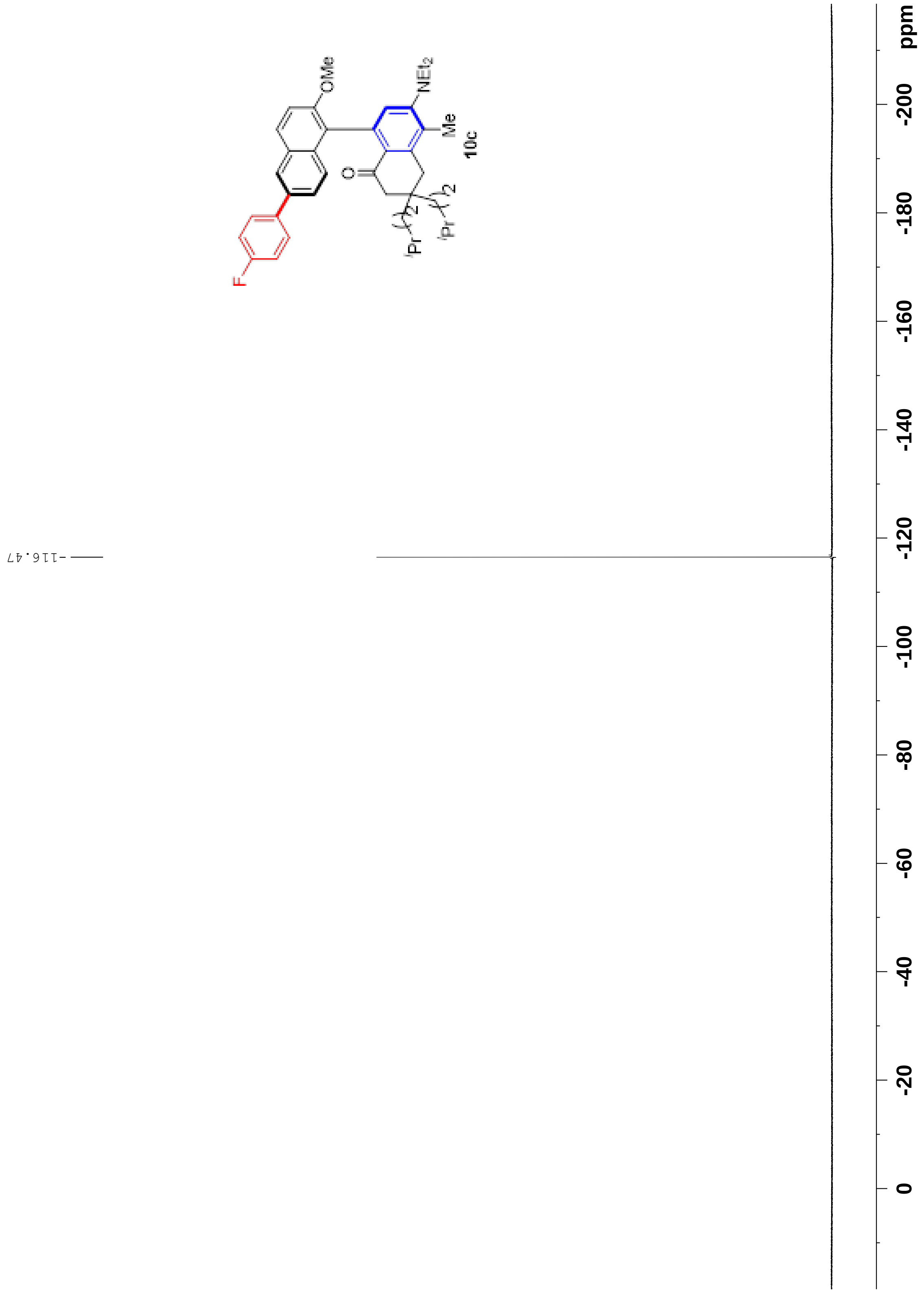

Supplementary Figure 151. ^{13}C NMR Spectrum of 10c

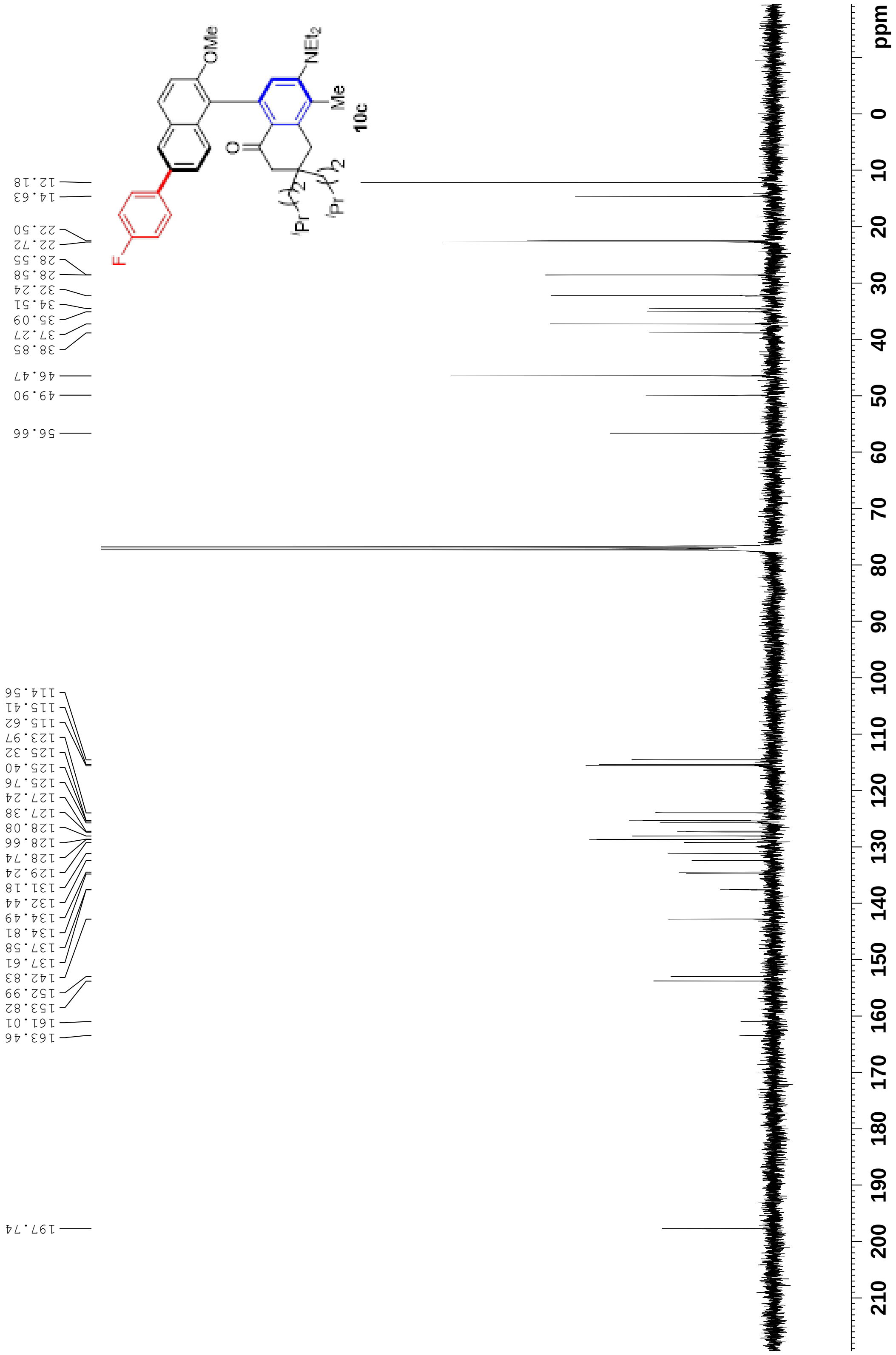

Supplementary Figure 152. ^{13}C NMR Spectrum of 10c

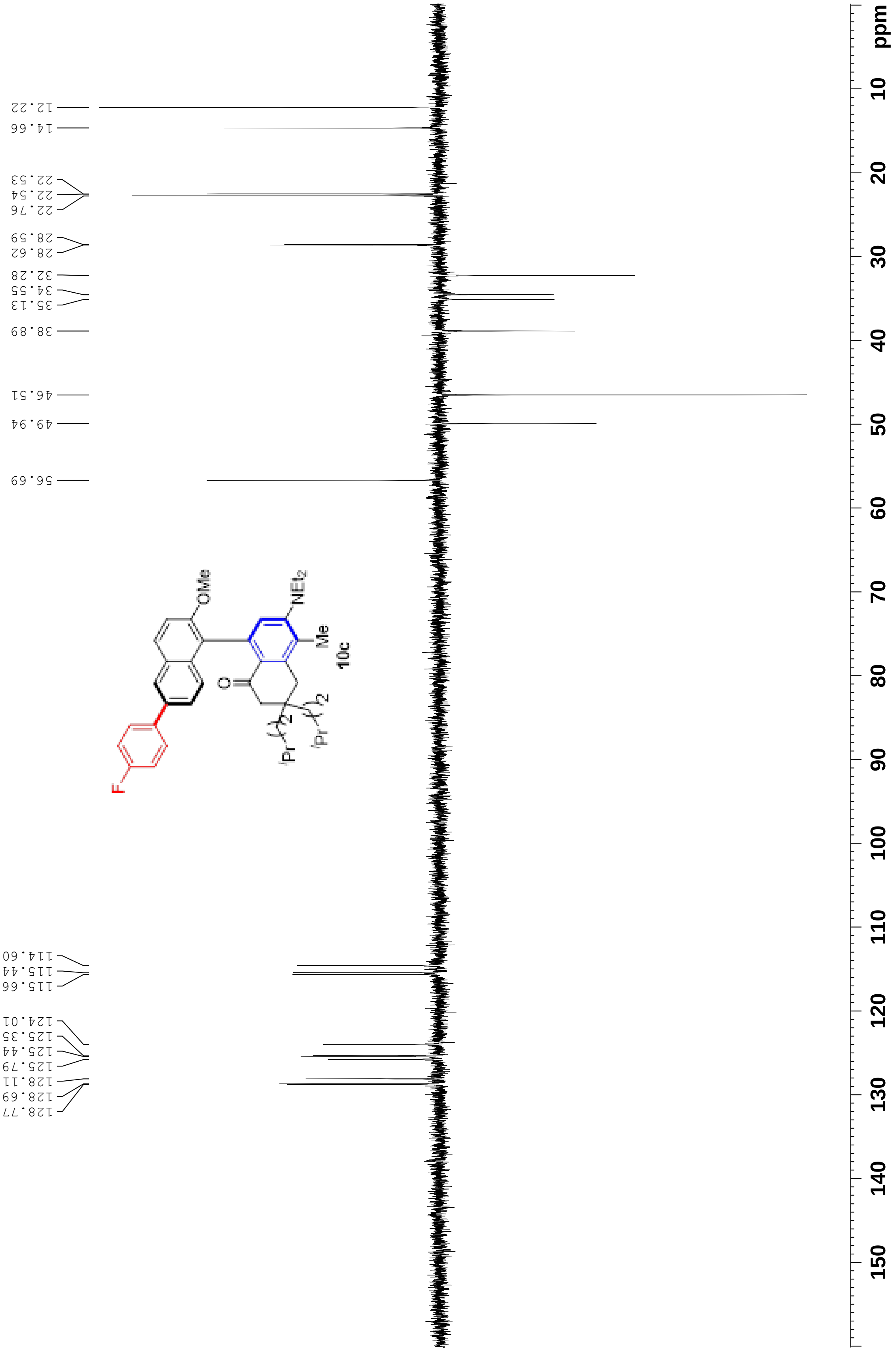

Supplementary Figure 153. HPLC Spectrum of racemic 10c

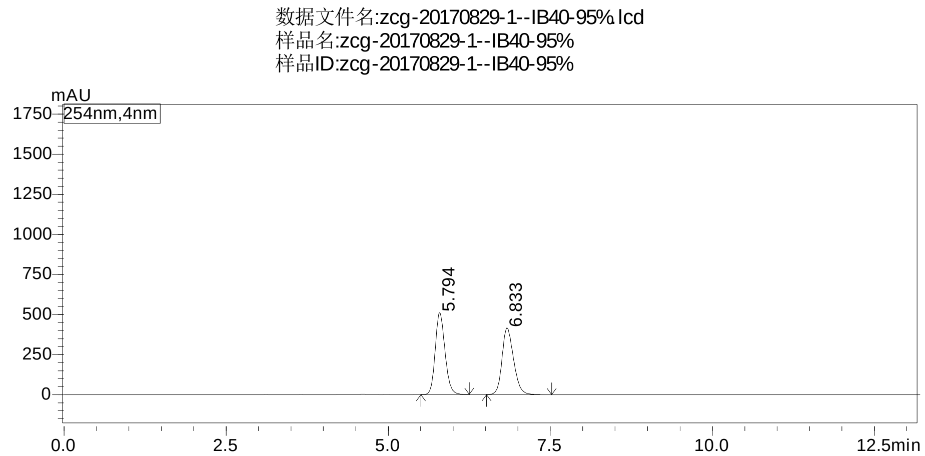

Peak#	Ret. Time	Area	Height	Area%	Height%
1	5.794	5378207	511182	50.999	55.179
2	6.833	5167519	415218	49.001	44.821
Total		10545726	926400	100.000	100.000

Supplementary Figure 154. HPLC Spectrum of 10c

数据文件名:zcg-20170915-1-IB40-95%.lcd
样品名:zcg-20170915-1-IB40-95%
样品ID:zcg-20170915-1-IB40-95%

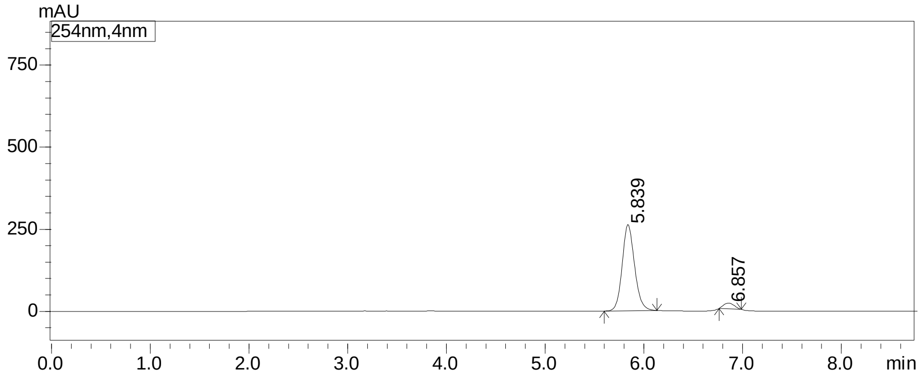

Peak#	Ret. Time	Area	Height	Area%	Height%
1	5.839	2286055	262530	93.048	92.874
2	6.857	170789	20144	6.952	7.126
Total		2456845	282675	100.000	100.000

Supplementary Figure 155. ^{1}H NMR Spectrum of 10d

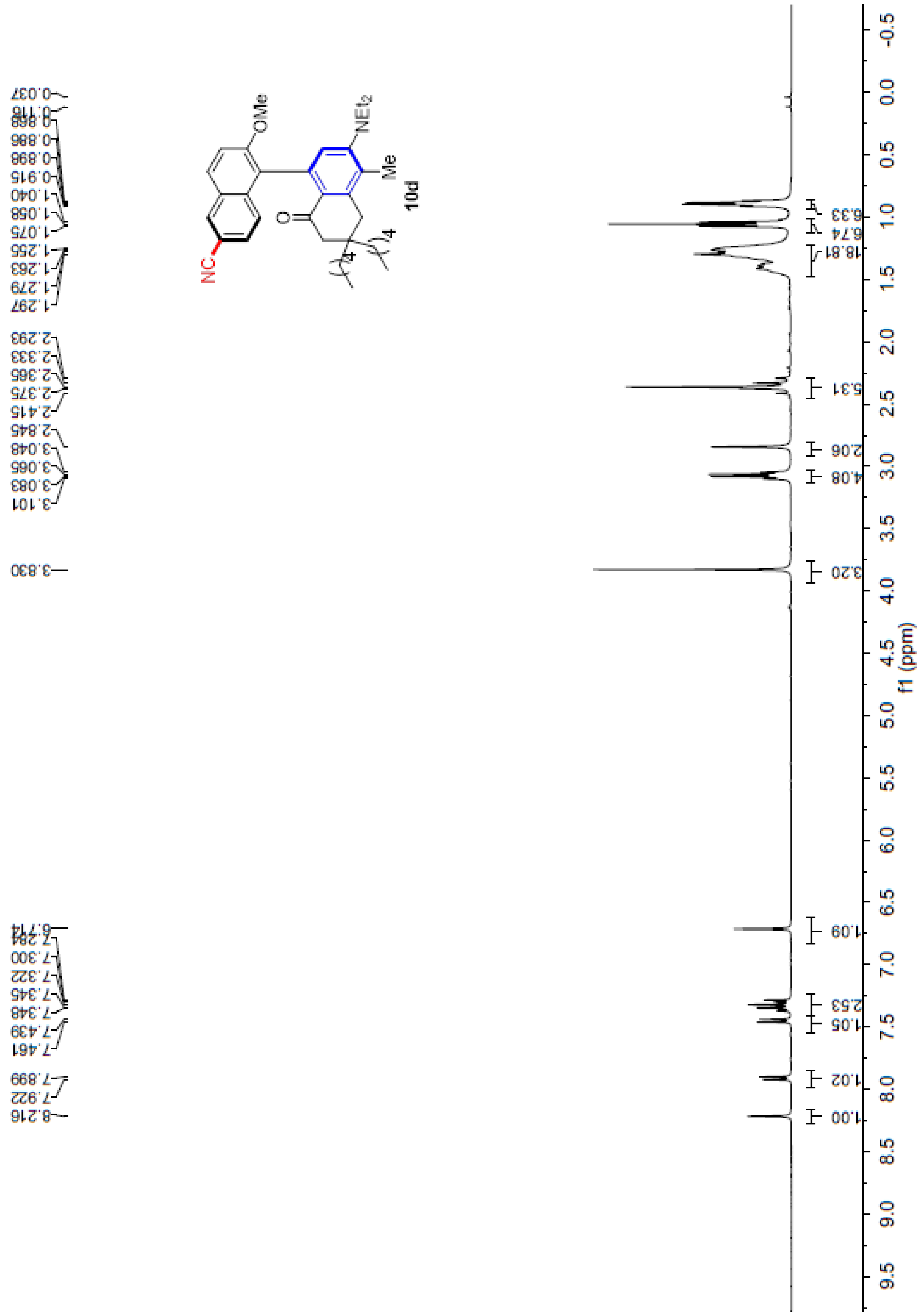

Supplementary Figure 156. ^{13}**C NMR Spectrum of 10d**

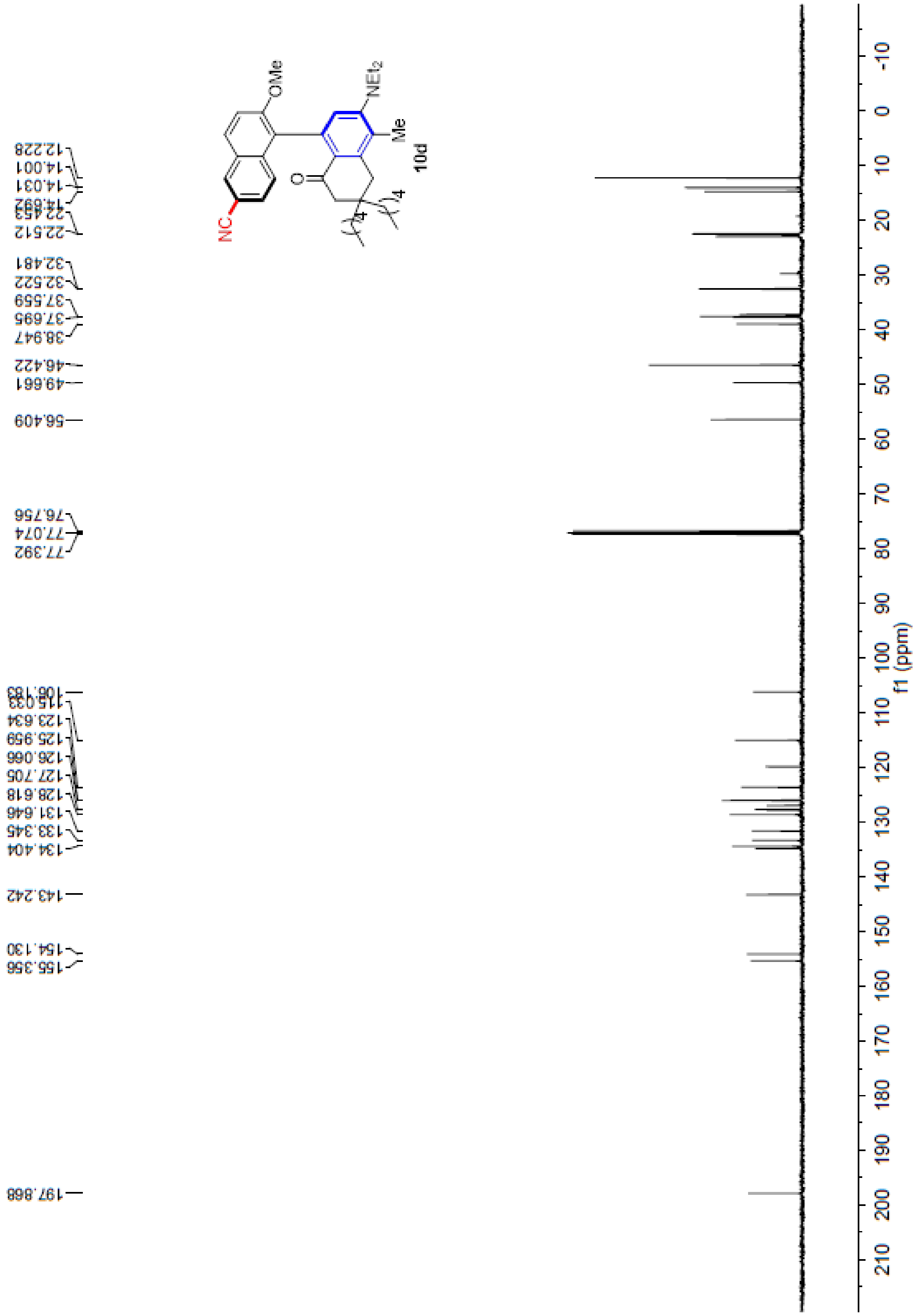

Supplementary Figure 157. HPLC Spectrum of racemic 10d

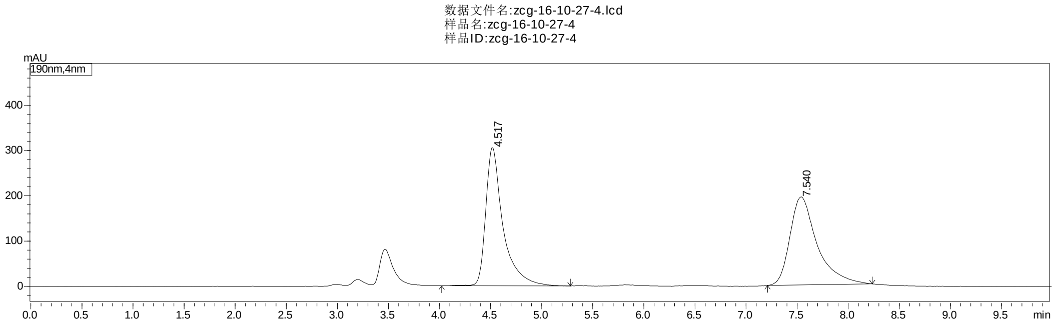

Peak#	Ret. Time	Area	Height	Area%	Height%
1	4.517	3602478	304856	49.961	61.052
2	7.540	3608147	194479	50.039	38.948
Total		7210625	499334	100.000	100.000

Supplementary Figure 158. HPLC Spectrum of 10d

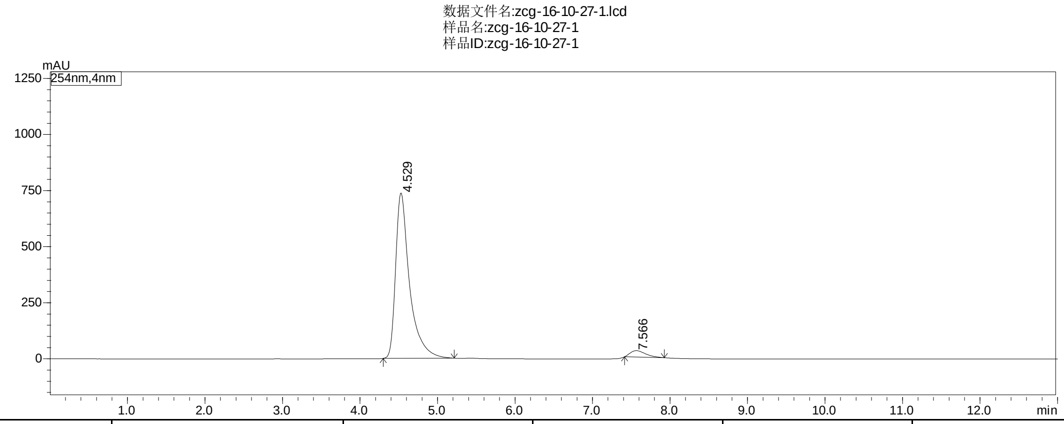

Peak#	Ret. Time	Area	Height	Area%	Height%
1	4.529	9250521	736931	95.883	96.235
2	7.566	397235	28832	4.117	3.765
Total		9647756	765763	100.000	100.000

Supplementary Figure 159. ¹H NMR Spectrum of 10e

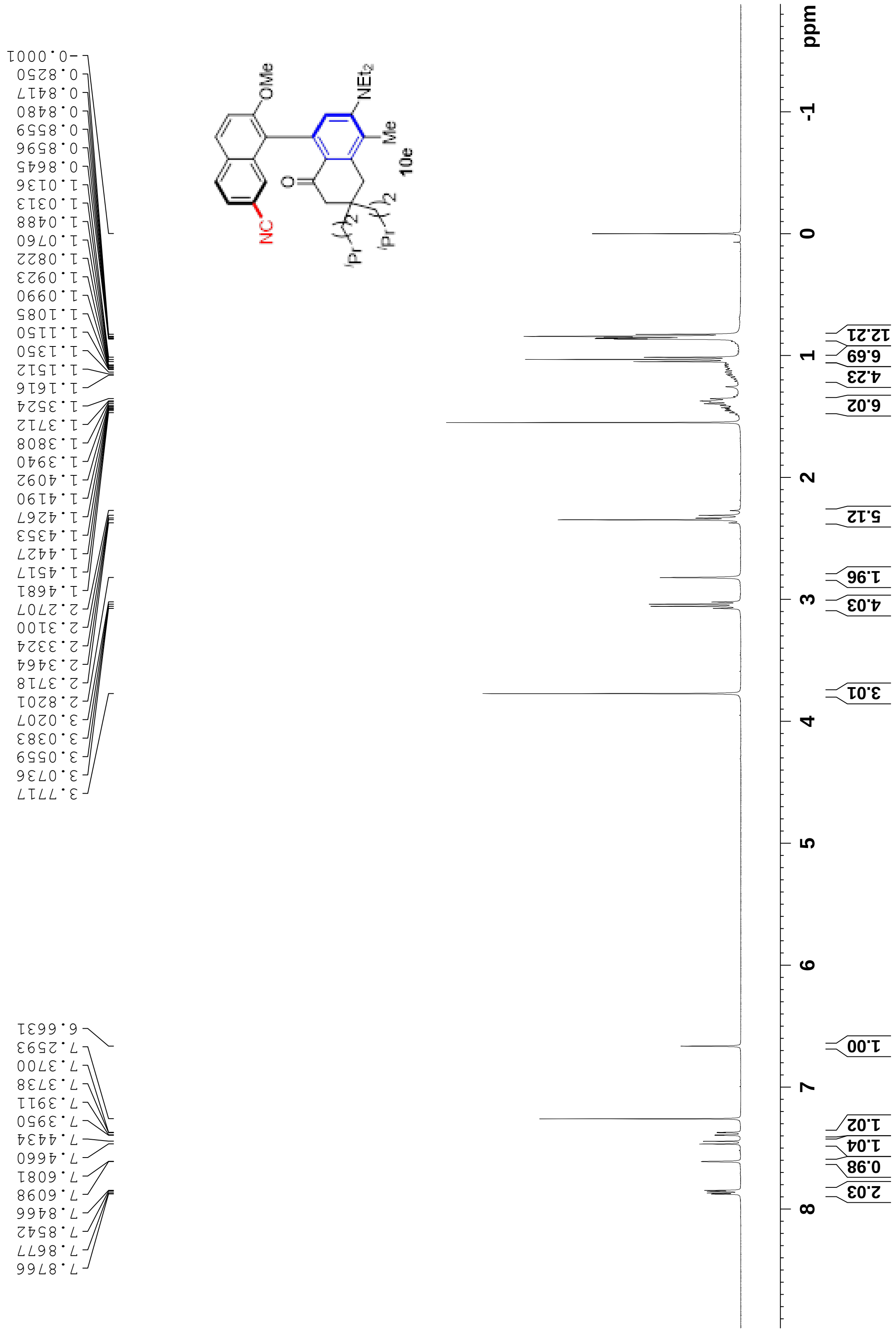

Supplementary Figure 160. ^{13}C NMR Spectrum of 10e

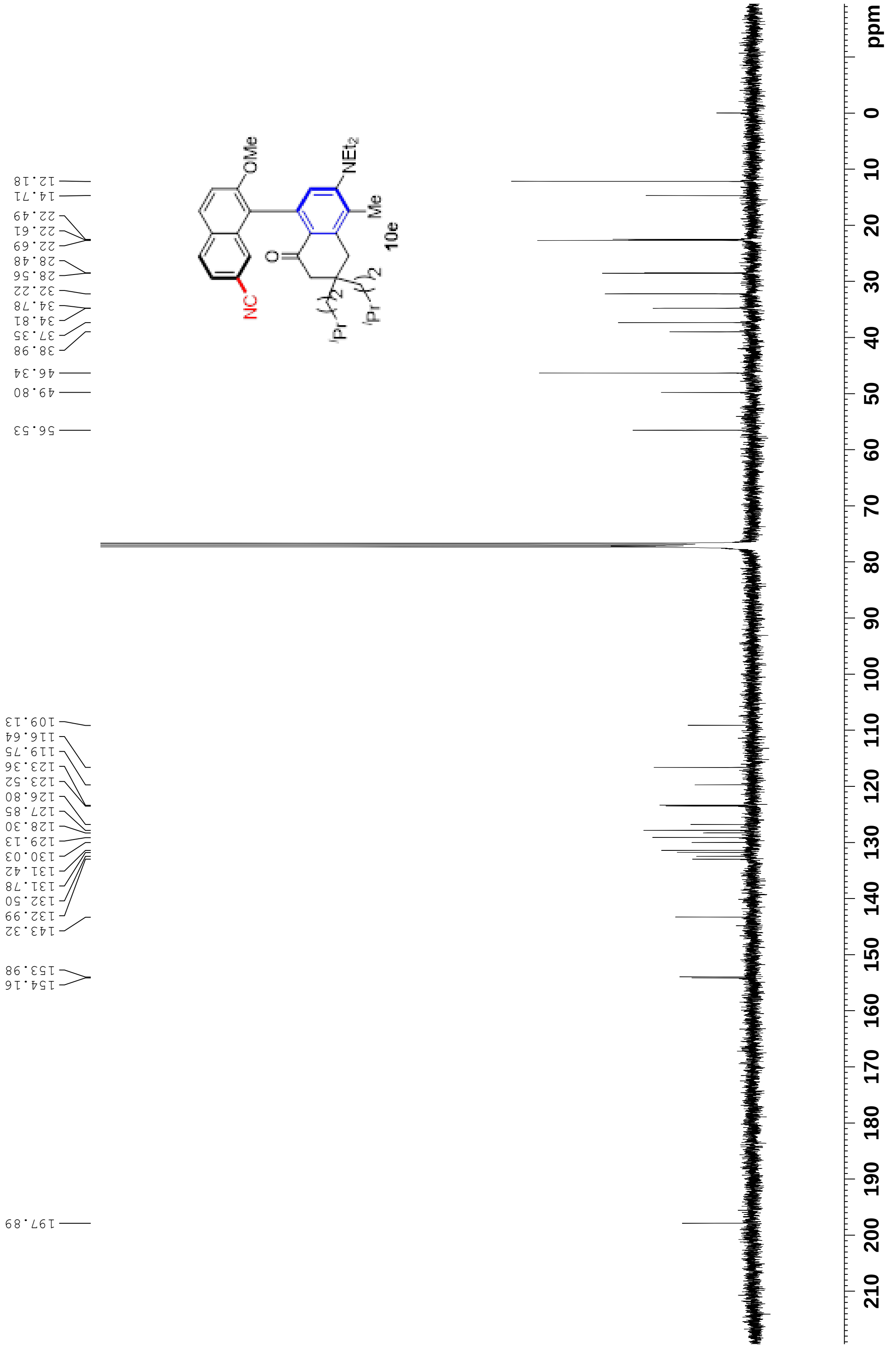

Supplementary Figure 161. ^{13}C NMR Spectrum of 10e

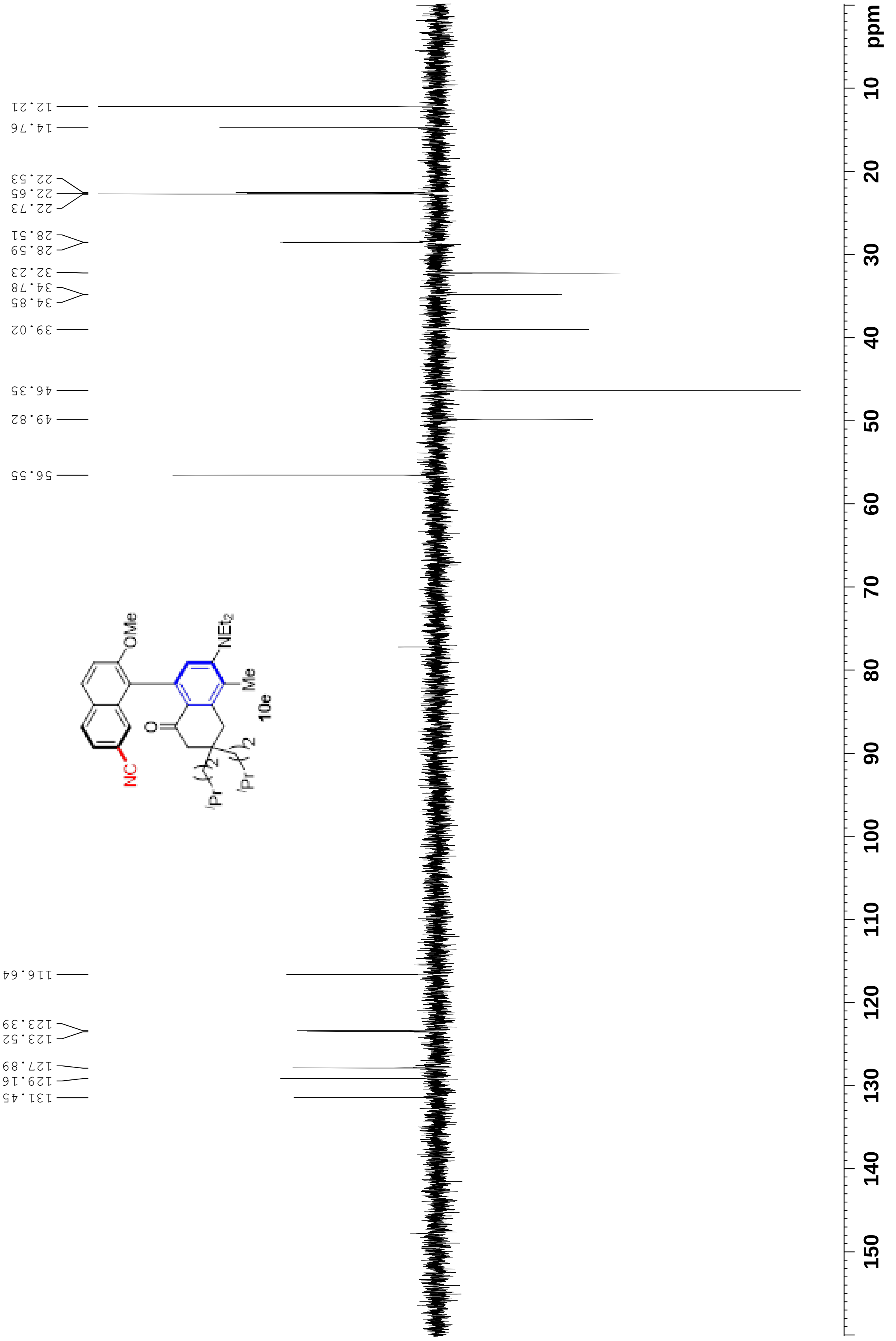

Supplementary Figure 162. HPLC Spectrum of racemic 10e

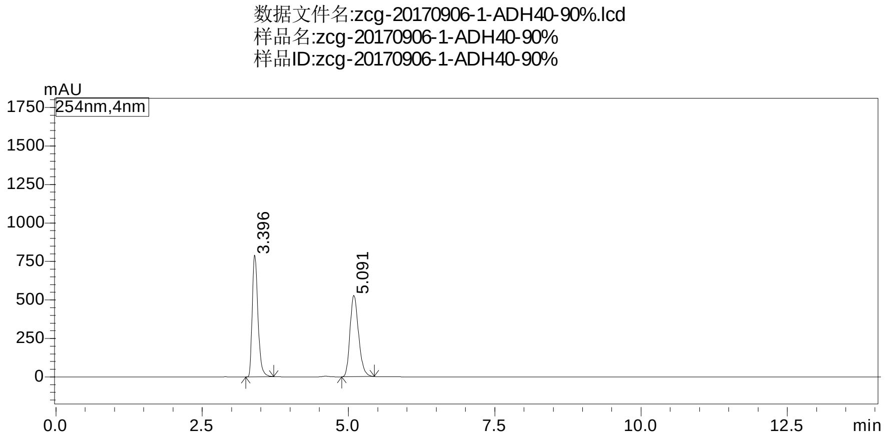

Peak#	Ret. Time	Area	Height	Area%	Height%
1	3.396	5370929	790228	49.928	59.919
2	5.091	5386396	528592	50.072	40.081
Total		10757326	1318820	100.000	100.000

Supplementary Figure 163. HPLC Spectrum of 10e

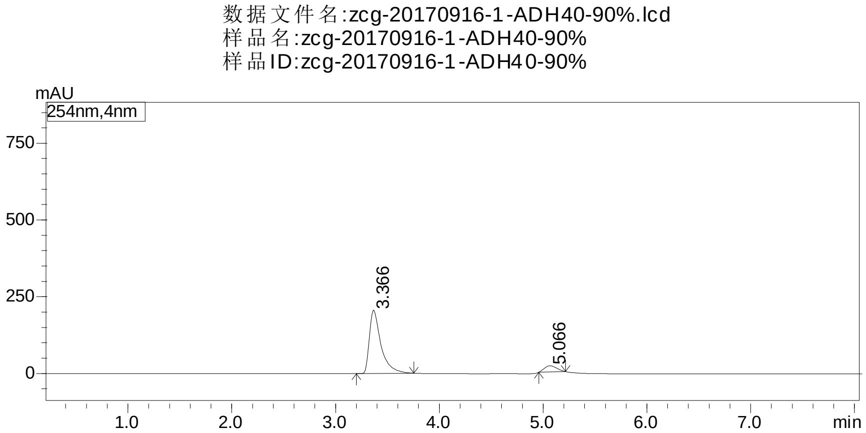

Peak#	Ret. Time	Area	Height	Area%	Height%
1	3.366	1606805	206745	90.580	91.104
2	5.066	167098	20187	9.420	8.896
Total		1773903	226932	100.000	100.000

Supplementary Figure 164. ^{1}H NMR Spectrum of 10f

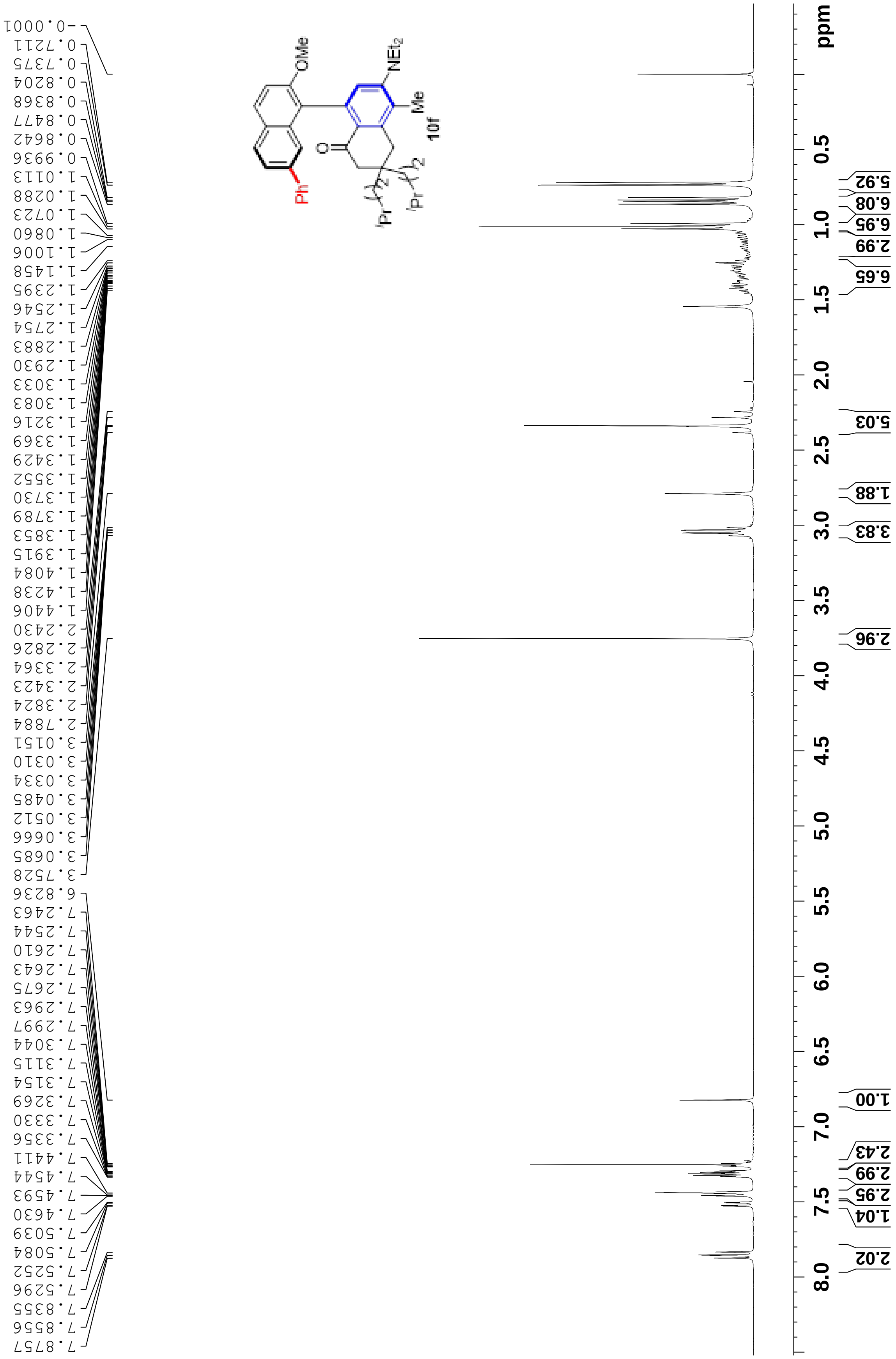

Supplementary Figure 165. ^{13}C NMR Spectrum of 10f

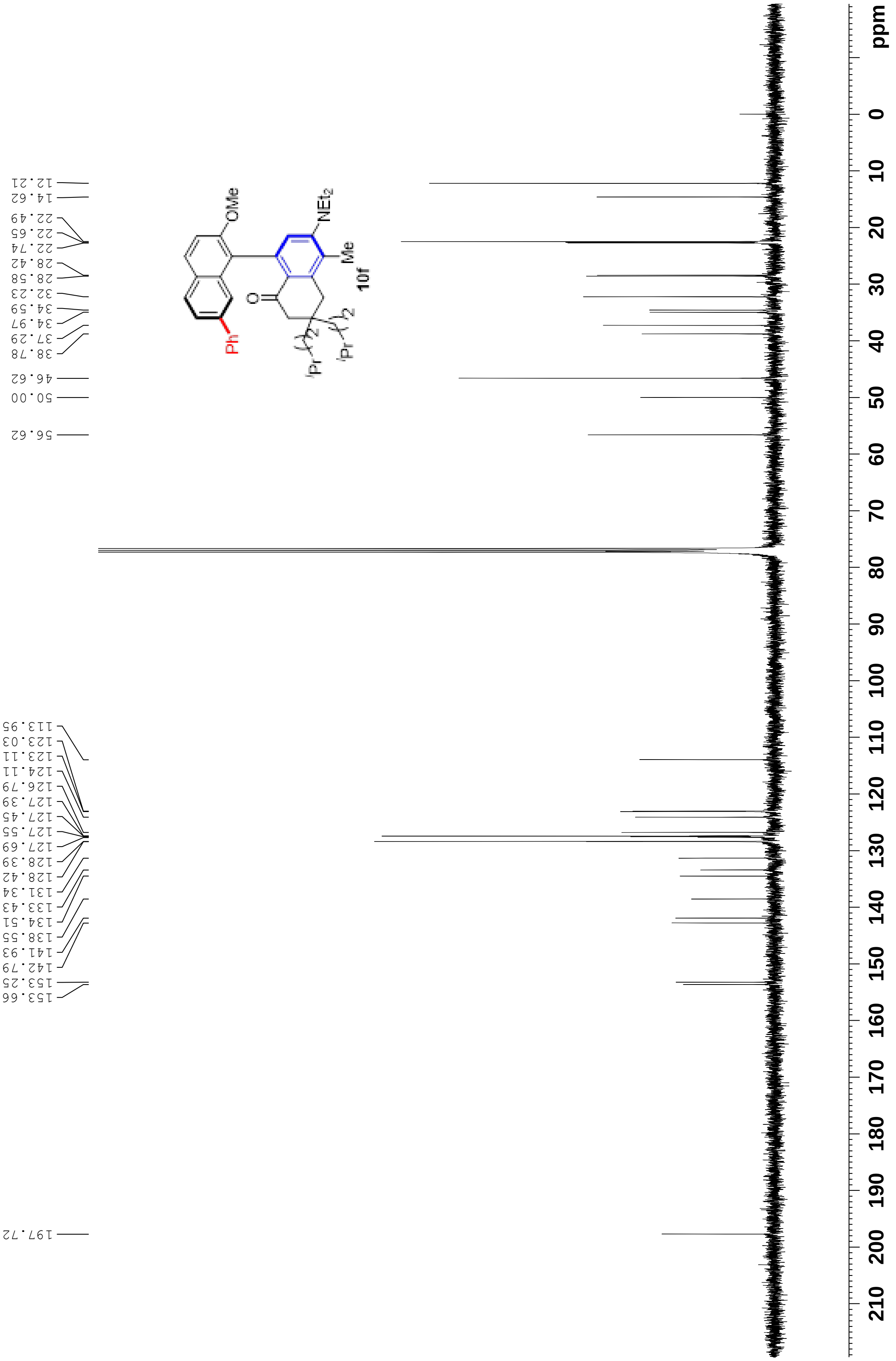

Supplementary Figure 166. ^{13}**C NMR Spectrum of 10f**

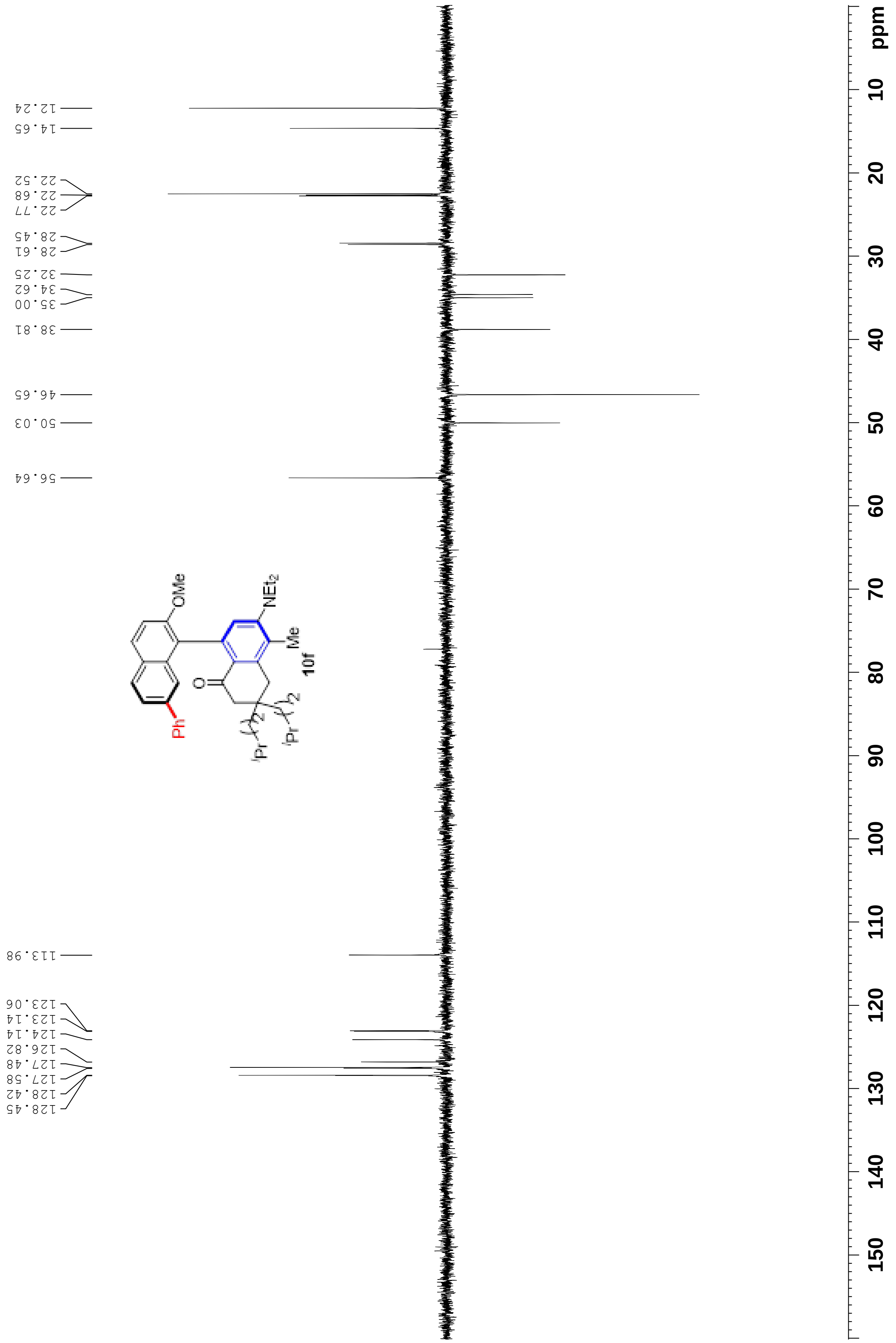

Supplementary Figure 167. HPLC Spectrum of racemic 10f

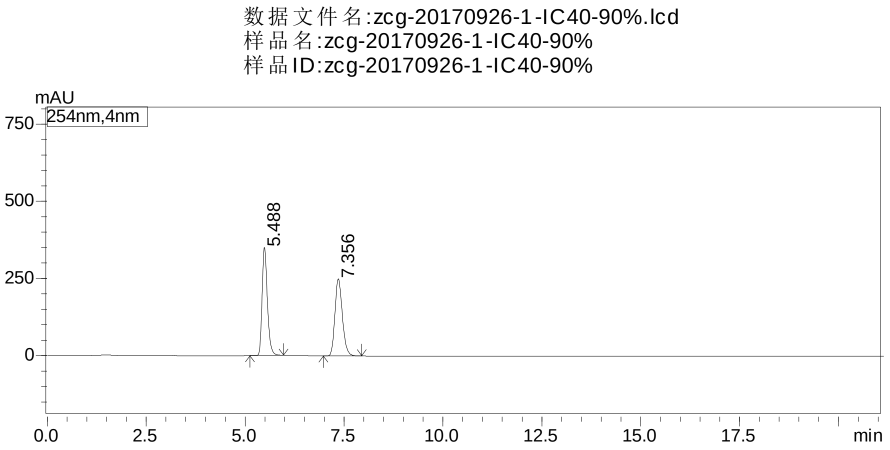

Peak#	Ret. Time	Area	Height	Area%	Height%
1	5.488	3258170	349886	50.058	58.381
2	7.356	3250581	249425	49.942	41.619
Total		6508751	599311	100.000	100.000

Supplementary Figure 168. HPLC Spectrum of 10f

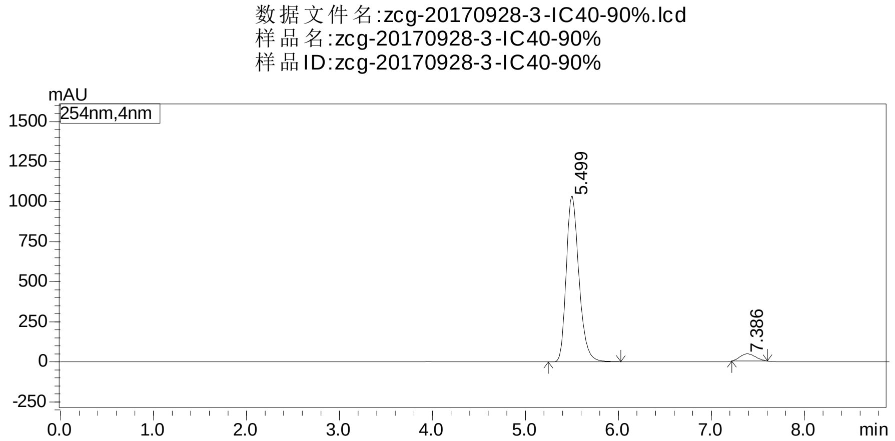

Peak#	Ret. Time	Area	Height	Area%	Height%
1	5.499	9828855	1034129	94.923	95.798
2	7.386	525704	45355	5.077	4.202
Total		10354559	1079485	100.000	100.000

Supplementary Figure 169. X-ray of 3au

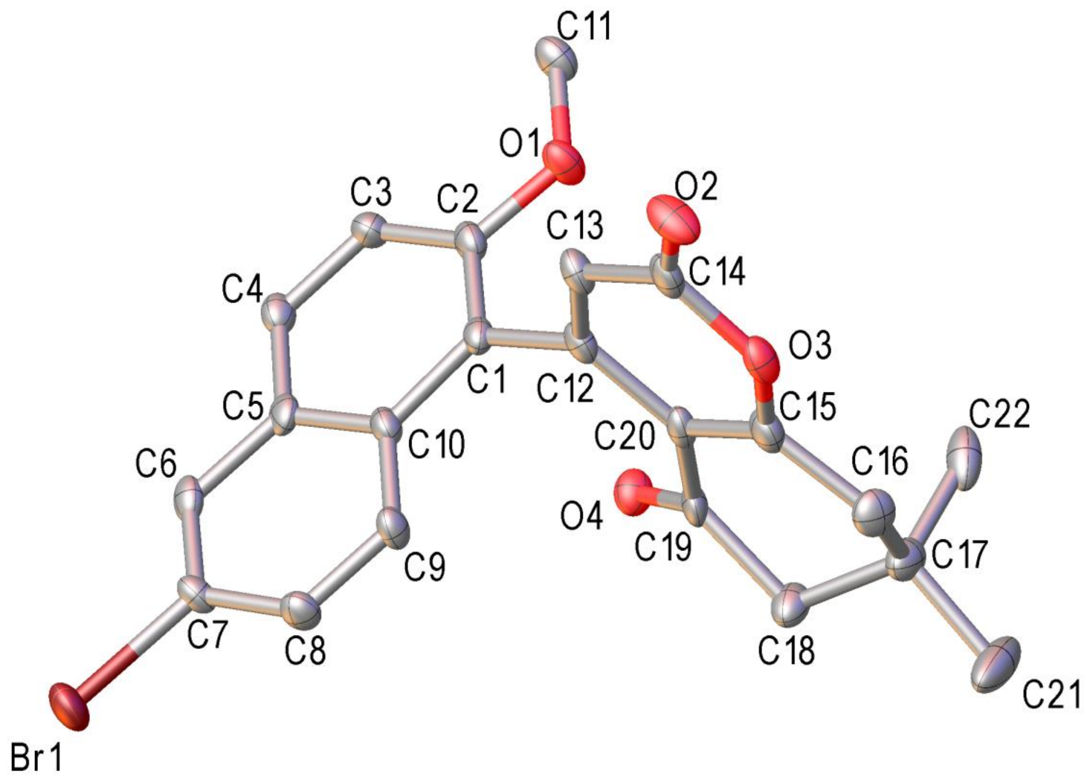

Supplementary Tables

Supplementary Table 1. **Crystal data and structure refinement for 3au.**

Identification code	a	
Empirical formula	C22 H19 Br O4	
Formula weight	427.28	
Temperature	173.1500 K	
Wavelength	0.71073 Å	
Crystal system	Orthorhombic	
Space group	P 21 21 21	
Unit cell dimensions	a = 11.666(2) Å	= 90°.
	b = 12.090(2) Å	= 90°.
	c = 13.715(3) Å	= 90°.
Volume	1934.4(6) $Å^3$	
Z	4	

Density (calculated)	1.467 Mg/m^3
Absorption coefficient	2.149 mm^{-1}
F(000)	872
Crystal size	0.385 x 0.276 x 0.174 mm^3
Theta range for data collection	2.246 to 27.483 °.
Index ranges	-15<=h<=15, -15<=k<=15, -17<=l<=17
Reflections collected	20386
Independent reflections	4432 [R(int) = 0.1690]
Completeness to theta = 25.242 °	100.0 %
Absorption correction	Semi-empirical from equivalents
Max. and min. transmission	1.00000 and 0.58344
Refinement method	Full-matrix least-squares on F^2
Data / restraints / parameters	4432 / 0 / 248
Goodness-of-fit on F^2	1.147
Final R indices [I>2sigma(I)]	R1 = 0.0852, wR2 = 0.2416
R indices (all data)	R1 = 0.1375, wR2 = 0.3162
Absolute structure parameter	0.06(3)
Extinction coefficient	n/a
Largest diff. peak and hole	1.047 and -1.211 e.$Å^{-3}$

Supplementary Table 2. Atomic coordinates (x 10^4) and equivalent isotropic displacement parameters (Å^{2}x 10^3) for 3au.

U(eq) is defined as one third of the trace of the orthogonalized U^{ij} tensor.

	x	y	z	U(eq)
Br1	3427(1)	1560(1)	-1572(1)	50(1)
O1	3650(6)	5700(7)	3181(6)	42(2)
O2	-799(7)	5520(7)	3099(6)	51(2)
O3	-83(6)	4028(6)	3798(5)	38(2)
O4	3660(6)	2594(6)	3363(5)	39(2)
C1	2988(8)	4337(8)	2137(7)	31(2)
C2	3813(9)	5110(9)	2353(7)	35(2)
C3	4780(9)	5260(8)	1743(7)	36(2)
C4	4881(9)	4632(9)	912(7)	36(2)
C5	4038(9)	3807(9)	664(7)	35(2)
C6	4124(9)	3177(9)	-201(7)	36(2)
C7	3327(8)	2393(8)	-411(7)	32(2)
C8	2425(9)	2185(9)	236(7)	37(2)
C9	2313(8)	2803(9)	1071(7)	34(2)
C10	3110(9)	3647(8)	1299(7)	32(2)
C11	4457(9)	6561(10)	3406(7)	41(2)
C12	1939(8)	4234(8)	2757(7)	33(2)
C13	1096(9)	4982(10)	2639(7)	37(2)
C14	-4(10)	4929(10)	3144(7)	41(2)
C15	766(10)	3278(10)	3940(7)	41(2)
C16	449(9)	2402(11)	4652(8)	43(2)
C17	1490(11)	1810(9)	5071(9)	48(3)
C18	2298(11)	1482(9)	4240(8)	45(3)
C19	2659(10)	2498(9)	3646(6)	36(2)
C20	1781(8)	3328(8)	3447(6)	31(2)
C21	1120(17)	753(13)	5611(13)	86(7)
C22	2111(12)	2561(13)	5799(8)	59(4)

Supplementary Table 3. Bond lengths [Å] and angles [°] for 3au.

Br1-C7	1.888(9)
O1-C2	1.354(12)
O1-C11	1.437(13)
O2-C14	1.172(14)
O3-C14	1.415(14)
O3-C15	1.356(14)
O4-C19	1.236(13)
C1-C2	1.373(14)
C1-C10	1.428(12)
C1-C12	1.495(13)
C2-C3	1.416(14)
C3-H3	0.9300
C3-C4	1.375(14)
C4-H4	0.9300
C4-C5	1.442(15)
C5-C6	1.413(14)
C5-C10	1.403(14)
C6-H6	0.9300
C6-C7	1.358(14)
C7-C8	1.399(13)
C8-H8	0.9300
C8-C9	1.374(13)
C9-H9	0.9300
C9-C10	1.415(14)
C11-H11A	0.9600
C11-H11B	0.9600
C11-H11C	0.9600
C12-C13	1.346(14)
C12-C20	1.460(14)
C13-H13	0.9300
C13-C14	1.460(14)
C15-C16	1.488(16)
C15-C20	1.366(13)
C16-H16A	0.9700
C16-H16B	0.9700
C16-C17	1.523(17)

C17-C18	1.531(14)
C17-C21	1.538(17)
C17-C22	1.53(2)
C18-H18A	0.9700
C18-H18B	0.9700
C18-C19	1.533(15)
C19-C20	1.459(14)
C21-H21A	0.9600
C21-H21B	0.9600
C21-H21C	0.9600
C22-H22A	0.9600
C22-H22B	0.9600
C22-H22C	0.9600

C2-O1-C11	118.1(8)
C15-O3-C14	123.9(8)
C2-C1-C10	120.1(9)
C2-C1-C12	120.5(8)
C10-C1-C12	119.4(9)
O1-C2-C1	116.2(9)
O1-C2-C3	122.6(10)
C1-C2-C3	121.3(9)
C2-C3-H3	120.4
C4-C3-C2	119.2(9)
C4-C3-H3	120.4
C3-C4-H4	119.4
C3-C4-C5	121.3(9)
C5-C4-H4	119.4
C6-C5-C4	121.5(9)
C10-C5-C4	118.4(9)
C10-C5-C6	120.1(10)
C5-C6-H6	119.8
C7-C6-C5	120.4(10)
C7-C6-H6	119.8
C6-C7-Br1	120.6(7)
C6-C7-C8	120.4(8)
C8-C7-Br1	119.0(7)
C7-C8-H8	119.9

C9-C8-C7	120.2(9)
C9-C8-H8	119.9
C8-C9-H9	119.6
C8-C9-C10	120.9(9)
C10-C9-H9	119.6
C5-C10-C1	119.8(9)
C5-C10-C9	118.0(8)
C9-C10-C1	122.2(9)
O1-C11-H11A	109.5
O1-C11-H11B	109.5
O1-C11-H11C	109.5
H11A-C11-H11B	109.5
H11A-C11-H11C	109.5
H11B-C11-H11C	109.5
C13-C12-C1	118.3(9)
C13-C12-C20	119.4(9)
C20-C12-C1	122.3(8)
C12-C13-H13	118.1
C12-C13-C14	123.8(11)
C14-C13-H13	118.1
O2-C14-O3	116.8(10)
O2-C14-C13	130.1(12)
O3-C14-C13	113.1(10)
O3-C15-C16	112.8(9)
O3-C15-C20	122.3(10)
C20-C15-C16	124.9(11)
C15-C16-H16A	109.1
C15-C16-H16B	109.1
C15-C16-C17	112.6(9)
H16A-C16-H16B	107.8
C17-C16-H16A	109.1
C17-C16-H16B	109.1
C16-C17-C18	109.4(10)
C16-C17-C21	110.4(12)
C16-C17-C22	110.2(10)
C18-C17-C21	108.5(10)
C18-C17-C22	110.3(11)
C22-C17-C21	108.1(13)

C17-C18-H18A	109.5
C17-C18-H18B	109.5
C17-C18-C19	110.9(9)
H18A-C18-H18B	108.0
C19-C18-H18A	109.5
C19-C18-H18B	109.5
O4-C19-C18	120.1(10)
O4-C19-C20	122.7(9)
C20-C19-C18	117.2(9)
C15-C20-C12	117.6(10)
C15-C20-C19	119.1(9)
C19-C20-C12	123.3(8)
C17-C21-H21A	109.5
C17-C21-H21B	109.5
C17-C21-H21C	109.5
H21A-C21-H21B	109.5
H21A-C21-H21C	109.5
H21B-C21-H21C	109.5
C17-C22-H22A	109.5
C17-C22-H22B	109.5
C17-C22-H22C	109.5
H22A-C22-H22B	109.5
H22A-C22-H22C	109.5
H22B-C22-H22C	109.5

Symmetry transformations used to generate equivalent atoms:

Supplementary Table 4. **Anisotropic displacement parameters ($Å^2$x 10^3) for 3au.**

The anisotropic displacement factor exponent takes the form: -2 ^{2}[h^2 $a^{*2}U^{11}$ + ...

+ 2 h k a* b* U^{12}]

	U^{11}	U^{22}	U^{33}	U^{23}	U^{13}	U^{12}
Br1	62(1)	57(1)	32(1)	-17(1)	6(1)	-1(1)
O1	36(4)	52(4)	37(4)	-16(3)	2(3)	-9(3)
O2	44(4)	58(5)	50(5)	-16(4)	-14(4)	25(4)
O3	28(3)	56(4)	30(3)	-12(3)	6(3)	-3(3)
O4	34(3)	47(4)	36(4)	4(3)	5(3)	7(3)
C1	41(5)	30(4)	22(4)	4(4)	5(3)	8(4)
C2	47(5)	37(5)	21(4)	-1(4)	-1(4)	6(4)
C3	46(5)	30(4)	32(5)	-4(4)	3(4)	-3(4)
C4	42(5)	37(5)	29(5)	1(4)	8(4)	10(4)
C5	46(5)	36(5)	21(4)	9(4)	1(4)	6(4)
C6	40(5)	47(5)	20(4)	8(4)	2(4)	12(4)
C7	30(4)	35(4)	30(4)	-11(4)	-1(4)	3(4)
C8	37(5)	38(5)	35(5)	-8(4)	-2(4)	-7(4)
C9	31(4)	44(5)	26(4)	-7(4)	6(4)	-3(4)
C10	41(5)	34(5)	22(4)	-3(3)	4(3)	6(4)
C11	40(5)	50(5)	34(5)	-11(5)	-5(4)	1(4)
C12	34(4)	43(5)	22(4)	-10(4)	0(3)	1(4)
C13	36(5)	51(6)	25(4)	-9(4)	3(4)	3(4)
C14	47(6)	51(6)	23(4)	-13(4)	2(4)	-7(5)
C15	48(6)	49(6)	25(4)	-10(4)	2(4)	-4(5)
C16	30(4)	51(6)	47(6)	2(5)	8(4)	-5(4)
C17	56(7)	39(6)	48(6)	5(5)	22(6)	4(5)
C18	53(6)	39(5)	42(6)	10(5)	15(5)	15(5)
C19	46(5)	44(5)	17(4)	-3(4)	7(4)	-2(4)
C20	31(4)	43(5)	19(4)	-6(4)	3(3)	-9(4)
C21	114(15)	53(8)	92(12)	31(8)	68(12)	30(9)
C22	62(8)	83(10)	33(6)	18(6)	12(5)	34(8)

Supplementary Table 5. Hydrogen coordinates (x 10^4) and isotropic displacement parameters ($Å^2$x 10^3) for 3au.

	x	y	z	U(eq)
H3	5339	5778	1903	44
H4	5506	4742	502	43
H6	4730	3299	-628	43
H8	1901	1626	101	44
H9	1705	2665	1493	40
H11A	4492	7075	2874	62
H11B	5201	6242	3509	62
H11C	4217	6940	3986	62
H13	1221	5566	2211	45
H16A	-42	1865	4332	51
H16B	16	2732	5181	51
H18A	1916	956	3816	54
H18B	2974	1127	4507	54
H21A	571	939	6106	130
H21B	1778	416	5908	130
H21C	781	246	5157	130
H22A	2180	3291	5529	88
H22B	2860	2269	5930	88
H22C	1681	2595	6395	88

Supplementary Table 6. Torsion angles [°] for 3au.

Br1-C7-C8-C9	-178.0(8)
O1-C2-C3-C4	-179.7(9)
O3-C15-C16-C17	-160.4(9)
O3-C15-C20-C12	0.7(14)
O3-C15-C20-C19	179.3(8)
O4-C19-C20-C12	11.4(14)
O4-C19-C20-C15	-167.1(10)
C1-C2-C3-C4	1.1(15)
C1-C12-C13-C14	175.3(9)
C1-C12-C20-C15	-176.6(9)
C1-C12-C20-C19	4.9(14)
C2-C1-C10-C5	-3.9(14)
C2-C1-C10-C9	176.9(9)
C2-C1-C12-C13	77.9(12)
C2-C1-C12-C20	-105.0(11)
C2-C3-C4-C5	-1.6(15)
C3-C4-C5-C6	178.5(9)
C3-C4-C5-C10	-0.7(14)
C4-C5-C6-C7	179.1(9)
C4-C5-C10-C1	3.4(14)
C4-C5-C10-C9	-177.4(9)
C5-C6-C7-Br1	179.3(7)
C5-C6-C7-C8	-1.4(15)
C6-C5-C10-C1	-175.8(8)
C6-C5-C10-C9	3.4(14)
C6-C7-C8-C9	2.7(16)
C7-C8-C9-C10	-0.9(16)
C8-C9-C10-C1	177.1(9)
C8-C9-C10-C5	-2.2(15)
C10-C1-C2-O1	-177.6(9)
C10-C1-C2-C3	1.6(14)
C10-C1-C12-C13	-100.2(11)
C10-C1-C12-C20	76.9(12)
C10-C5-C6-C7	-1.7(14)
C11-O1-C2-C1	-176.5(9)
C11-O1-C2-C3	4.3(14)
C12-C1-C2-O1	4.3(14)
C12-C1-C2-C3	-176.4(9)
C12-C1-C10-C5	174.2(9)
C12-C1-C10-C9	-5.0(14)
C12-C13-C14-O2	-179.6(11)
C12-C13-C14-O3	1.9(14)

C13-C12-C20-C15	0.5(13)
C13-C12-C20-C19	-178.0(9)
C14-O3-C15-C16	-178.7(9)
C14-O3-C15-C20	-0.6(14)
C15-O3-C14-O2	-179.3(9)
C15-O3-C14-C13	-0.7(13)
C15-C16-C17-C18	-47.8(14)
C15-C16-C17-C21	-167.1(11)
C15-C16-C17-C22	73.6(12)
C16-C15-C20-C12	178.6(9)
C16-C15-C20-C19	-2.8(15)
C16-C17-C18-C19	57.3(14)
C17-C18-C19-O4	139.2(11)
C17-C18-C19-C20	-40.3(14)
C18-C19-C20-C12	-169.1(9)
C18-C19-C20-C15	12.4(13)
C20-C12-C13-C14	-1.9(15)
C20-C15-C16-C17	21.5(16)
C21-C17-C18-C19	177.7(13)
C22-C17-C18-C19	-64.1(13)

Symmetry transformations used to generate equivalent atoms:

Supplementary Table 7. Hydrogen bonds for 3au [Å and °].

D-H...A	d(D-H)	d(H...A)	d(D...A)	<(DHA)

Supplementary Discussion

Computational Details

All structures were optimized at the B3LYP/6-31G(d,p) level of theory. Minima were confirmed by frequency calculations (1 imaginary frequency for transition state, 0 imaginary frequency for intermediates).

Conformers

As intermediate **VI**, acyl azolium, has been ruled out by equation (2), only path A starting from **II** was investigated. **II** has four conformers denoted A, B, C, and D (Supplementary Figure 170). **II_A** and **II_C** with the carbonyl group pointing towards the indane ring is favored over **II_B** and **II_D** where the carbonyl group is towards the tribromophenyl group. Attention was focused on **II_A** as the methoxy group is in the same direction as the carbonyl group for an important favorable coordination to magnesium during the reaction.

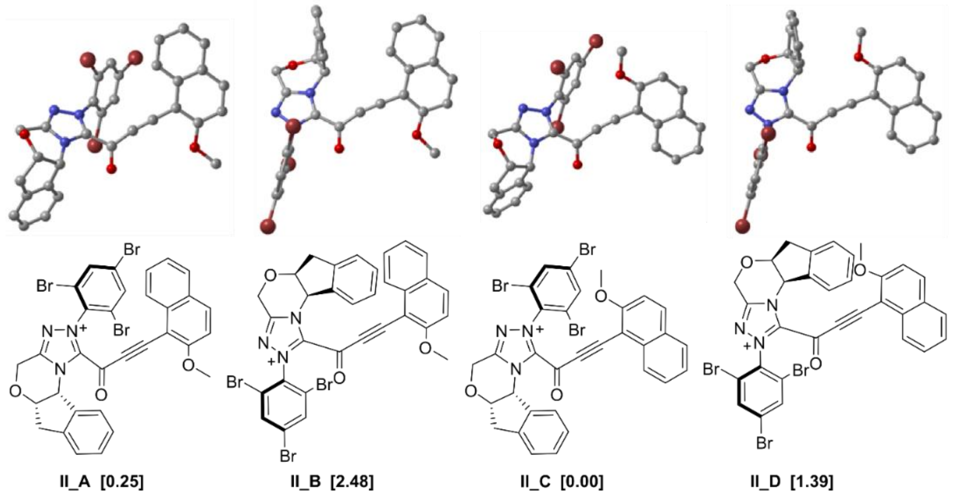

Supplementary Figure 170. Optimized conformers of II and relative Gibbs Free Energies given in kcal/mol.

The alkynyl azolium creates a twisted plane in which the ketoenolate can attack from two different sides (Supplementary Figure 171);

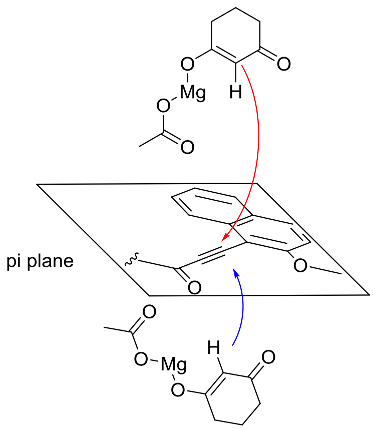

Supplementary Figure 171. The attack by the ketoenolate can happen from either sides.

Furthermore, the Mg-coordinated ketoenolate has two enantio-faces for the nucleophilic attack (Supplementary Figure 172). The attack via **TS1(i)** will lead to an allenolate ready for the next proton transfer, while the attack via **TS1(ii)** results in an allenolate with the proton pointing away from the allene moiety and creates a higher energy barrier (2.78 kcal/mole higher, Supplementary Table 8).

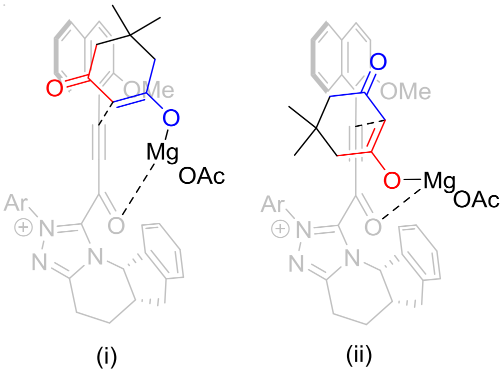

Supplementary Figure 172. The ketoenolate can attack in two different ways, (i) and (ii).

Supplementary Table 8. Comparison between TS1(i) and TS1(ii).

<table>
<tr><td rowspan="2">i</td><td colspan="2">TS1(i) (0 kcal/mol)</td></tr>
<tr><td>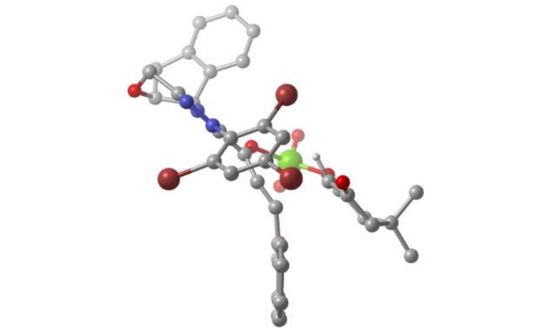</td><td>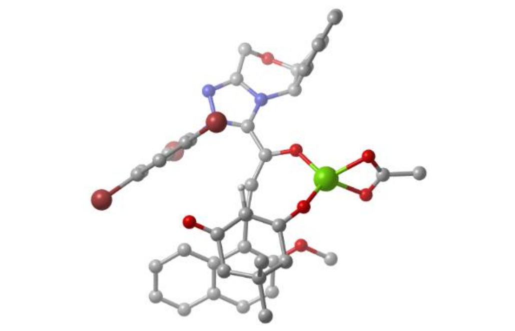</td></tr>
<tr><td>ii</td><td colspan="2">TS1(ii) (+2.78 kcal/mol)</td></tr>
</table>

	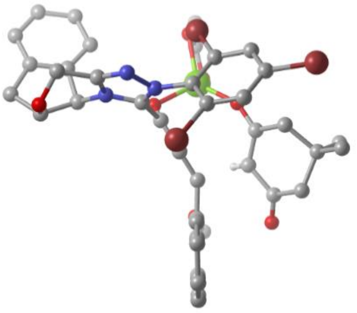	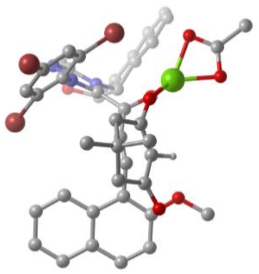

We hypothesize that enantioselectivity is determined by the attack on the pi plane by the ketoenolate (Figure 172 and 173).

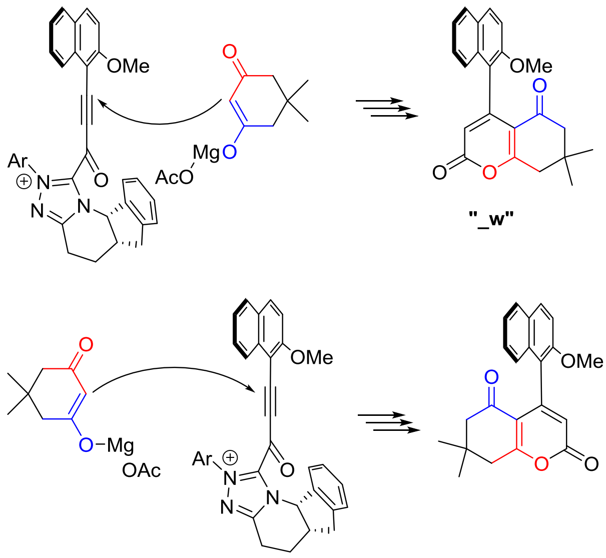

Supplementary Figure 173. The attack by the ketoenolate on the alkyne bond .

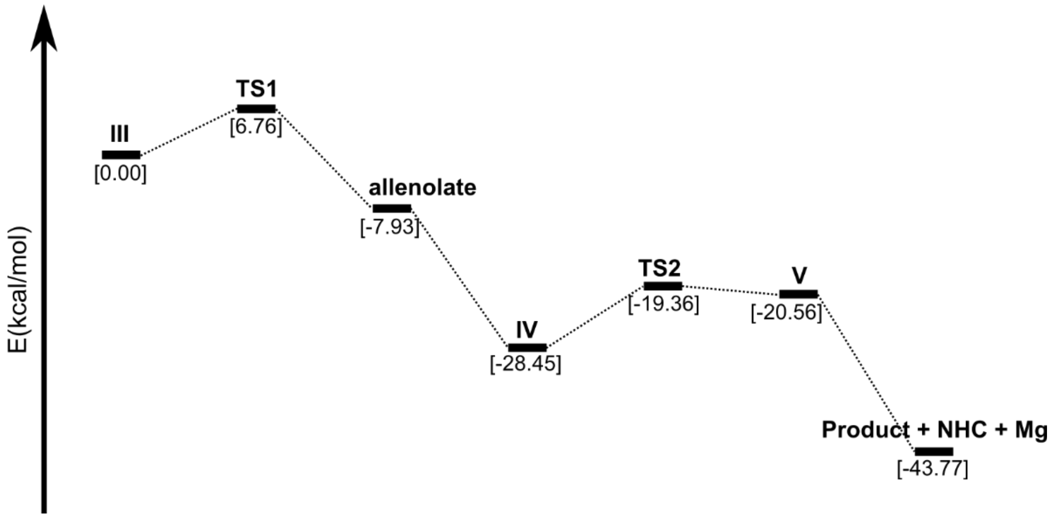

Supplementary Figure 174. Energy profile of Path A from Figure 6 for **A**.

Geometries and Energies

II_A

E(RB3LYP/6-31G(d,p)) = -9336.82746821 A.U.

Temp. 298.150 K 1.00000 Atm.

E + zero-point Energies = -9336.367804
E + thermal Energies = -9336.333140
E + thermal Enthalpies = -9336.332196
E + thermal Free Energies = -9336.439358

Geometry at E(RB3LYP/6-31G(d,p))
Charge: 1 Multiplicity: 1
C -2.767866000000 2.595911000000 -0.119090000000
C -3.162194000000 3.638569000000 -0.993607000000
C -4.482027000000 4.144260000000 -0.940775000000
C -5.381047000000 3.627133000000 -0.035805000000
H -4.789158000000 4.936122000000 -1.611440000000
H -6.392080000000 4.024168000000 -0.008346000000
C -1.460270000000 2.109576000000 -0.210746000000
C -0.311127000000 1.689094000000 -0.308282000000
C 1.067187000000 1.444050000000 -0.450926000000
O 1.859225000000 2.132159000000 -1.095722000000
C 3.087946000000 -0.999698000000 1.363656000000
C 1.660039000000 0.262641000000 0.284806000000
N 2.050760000000 -1.769876000000 1.172964000000
N 2.894019000000 0.248563000000 0.822785000000
C 3.975300000000 1.262586000000 0.847739000000
H 3.499679000000 2.235544000000 0.717467000000
C 4.328322000000 -1.211627000000 2.171164000000
H 5.190968000000 -1.417918000000 1.523743000000
H 4.193260000000 -2.046543000000 2.859860000000
C 4.756160000000 1.171803000000 2.219352000000
H 4.423490000000 1.959852000000 2.896670000000
O 4.492139000000 -0.031668000000 2.947504000000
C 6.248806000000 1.326372000000 1.836126000000
H 6.585243000000 2.348950000000 2.046554000000
H 6.863768000000 0.661041000000 2.450376000000
C 5.030766000000 0.994451000000 -0.208854000000
C 6.309034000000 1.030877000000 0.356809000000
C 4.844120000000 0.770231000000 -1.573618000000
C 7.431979000000 0.834978000000 -0.447810000000
C 5.970414000000 0.565871000000 -2.371165000000

H 3.847827000000 0.755510000000 -2.002434000000
C 7.253466000000 0.598930000000 -1.811944000000
H 8.430162000000 0.862187000000 -0.020941000000
H 5.851602000000 0.386306000000 -3.434913000000
H 8.119541000000 0.440100000000 -2.446831000000
N 1.163035000000 -0.967571000000 0.495341000000
C -0.053759000000 -1.535060000000 0.008117000000
C -1.065925000000 -1.901908000000 0.904321000000
C -0.217919000000 -1.801862000000 -1.359182000000
C -2.229258000000 -2.513083000000 0.450435000000
C -1.380773000000 -2.404161000000 -1.827943000000
C -2.375576000000 -2.755471000000 -0.915476000000
H -3.008260000000 -2.790587000000 1.147944000000
H -1.504353000000 -2.604209000000 -2.884247000000
Br -0.876060000000 -1.546654000000 2.755232000000
Br 1.146030000000 -1.353869000000 -2.600439000000
Br -3.957725000000 -3.582183000000 -1.543719000000
C -3.707268000000 2.066336000000 0.842026000000
C -5.034233000000 2.592131000000 0.872427000000
C -5.967706000000 2.079292000000 1.809352000000
C -5.604691000000 1.084831000000 2.691393000000
C -4.288143000000 0.574970000000 2.666123000000
H -3.994158000000 -0.201905000000 3.365798000000
C -3.359951000000 1.055227000000 1.764010000000
H -6.322702000000 0.698161000000 3.407014000000
H -6.974004000000 2.488003000000 1.821548000000
H -2.350385000000 0.663413000000 1.767958000000
O -2.228160000000 4.081942000000 -1.847166000000
C -2.539699000000 5.142388000000 -2.758070000000
H -1.622683000000 5.318900000000 -3.318459000000
H -3.339174000000 4.849198000000 -3.446005000000
H -2.822140000000 6.053325000000 -2.220873000000

II_B

E(RB3LYP/6-31G(d,p)) = -9336.82149543 A.U.

Temp. 298.150 K 1.00000 Atm.

E + zero-point Energies = -9336.362132
E + thermal Energies = -9336.327292
E + thermal Enthalpies = -9336.326348
E + thermal Free Energies = -9336.435796

Geometry at E(RB3LYP/6-31G(d,p))

Charge: 1 Multiplicity: 1
C 3.610653000000 2.463564000000 0.215033000000
C 3.603099000000 3.853385000000 0.492609000000
C 4.815302000000 4.582366000000 0.491429000000
C 6.001505000000 3.940964000000 0.219090000000
H 4.813537000000 5.642550000000 0.708588000000
H 6.926157000000 4.511432000000 0.226083000000
C 2.389572000000 1.781541000000 0.276614000000
C 1.302583000000 1.217612000000 0.363792000000
C -0.056980000000 0.848560000000 0.491396000000
O -0.986440000000 1.644065000000 0.584772000000
C -0.507687000000 -2.791781000000 0.835853000000
C -0.416740000000 -0.618810000000 0.548094000000
N -1.746176000000 -2.433468000000 0.627341000000
N 0.339719000000 -1.710615000000 0.787834000000
C 1.796707000000 -1.914459000000 0.891731000000
H 2.237913000000 -0.957607000000 1.178039000000
C 0.074742000000 -4.111495000000 1.228880000000
H 0.543488000000 -4.607615000000 0.368485000000
H -0.704879000000 -4.761094000000 1.628996000000
C 2.118555000000 -3.024569000000 1.964599000000
H 2.384128000000 -2.566948000000 2.919065000000
O 0.995482000000 -3.849479000000 2.281443000000
C 3.300909000000 -3.818379000000 1.354028000000
H 4.246735000000 -3.474065000000 1.790534000000
H 3.206860000000 -4.880296000000 1.600535000000
C 2.381289000000 -2.459441000000 -0.399144000000
C 3.245596000000 -3.525125000000 -0.126861000000
C 2.158808000000 -2.015672000000 -1.703609000000
C 3.920281000000 -4.156127000000 -1.172630000000
C 2.831010000000 -2.655693000000 -2.746495000000
H 1.462585000000 -1.208529000000 -1.915324000000
C 3.708247000000 -3.713651000000 -2.480636000000
H 4.590978000000 -4.987443000000 -0.976575000000
H 2.670639000000 -2.332610000000 -3.769934000000
H 4.221359000000 -4.203195000000 -3.302432000000
N -1.675267000000 -1.078879000000 0.445364000000
C -2.863426000000 -0.367232000000 0.087251000000
C -3.794682000000 -0.011966000000 1.066395000000
C -3.119007000000 -0.070181000000 -1.254813000000
C -4.977623000000 0.627509000000 0.711937000000
C -4.293822000000 0.572587000000 -1.626050000000
C -5.214438000000 0.910999000000 -0.633192000000
H -5.697911000000 0.904282000000 1.470609000000

H -4.489765000000 0.804038000000 -2.664753000000
Br -3.427468000000 -0.372888000000 2.886580000000
Br -1.841175000000 -0.525480000000 -2.586283000000
Br -6.821301000000 1.784276000000 -1.125434000000
C 6.134814000000 -0.175204000000 -0.703286000000
H 6.162286000000 -1.229698000000 -0.960698000000
C 7.333834000000 0.569352000000 -0.673646000000
C 7.294721000000 1.912617000000 -0.369744000000
C 4.922331000000 0.422523000000 -0.419840000000
C 4.853358000000 1.795399000000 -0.092461000000
C 6.063781000000 2.553689000000 -0.078512000000
O 2.407546000000 4.397435000000 0.758784000000
C 2.307192000000 5.795029000000 1.055854000000
H 2.871841000000 6.047314000000 1.959114000000
H 2.653645000000 6.402875000000 0.214076000000
H 1.246929000000 5.977078000000 1.225317000000
H 8.206969000000 2.501998000000 -0.353221000000
H 8.278627000000 0.085498000000 -0.898010000000
H 4.008383000000 -0.157666000000 -0.474452000000

II_C

E(RB3LYP/6-31G(d,p)) = -9336.82755746 A.U.

Temp. 298.150 K 1.00000 Atm.
E + zero-point Energies = -9336.367816
E + thermal Energies = -9336.333179
E + thermal Enthalpies = -9336.332235
E + thermal Free Energies = -9336.439756

Geometry at E(RB3LYP/6-31G(d,p))
Charge: 1 Multiplicity: 1
C -2.988892000000 2.116043000000 0.583153000000
C -3.826011000000 1.336597000000 1.414409000000
C -5.169034000000 1.720089000000 1.624000000000
C -5.662243000000 2.854683000000 1.017910000000
H -5.815677000000 1.127273000000 2.257385000000
H -6.698171000000 3.135787000000 1.186526000000
C -1.655402000000 1.748889000000 0.384789000000
C -0.475991000000 1.496882000000 0.158169000000
C 0.907132000000 1.441151000000 -0.091444000000
O 1.577307000000 2.329954000000 -0.620445000000
C 3.252079000000 -1.105290000000 1.070348000000
C 1.655007000000 0.210296000000 0.357719000000

N 2.252659000000 -1.909266000000 0.822019000000
N 2.930609000000 0.200329000000 0.786530000000
C 3.938835000000 1.279129000000 0.916662000000
H 3.387936000000 2.215388000000 1.014539000000
C 4.571321000000 -1.359488000000 1.726557000000
H 5.383439000000 -1.379078000000 0.987587000000
H 4.551853000000 -2.311584000000 2.258309000000
C 4.853397000000 1.002410000000 2.176445000000
H 4.542464000000 1.630475000000 3.012643000000
O 4.729828000000 -0.327104000000 2.691244000000
C 6.291210000000 1.334241000000 1.704945000000
H 6.590364000000 2.320119000000 2.081159000000
H 6.998780000000 0.611407000000 2.123412000000
C 4.901563000000 1.299890000000 -0.255813000000
C 6.224212000000 1.328403000000 0.196778000000
C 4.596183000000 1.324522000000 -1.617465000000
C 7.272814000000 1.376154000000 -0.722659000000
C 5.649224000000 1.363722000000 -2.531650000000
H 3.565546000000 1.313281000000 -1.956378000000
C 6.976372000000 1.389565000000 -2.086617000000
H 8.304606000000 1.399790000000 -0.384712000000
H 5.438244000000 1.380794000000 -3.596122000000
H 7.784345000000 1.421813000000 -2.810867000000
N 1.259071000000 -1.072045000000 0.374449000000
C 0.040745000000 -1.631719000000 -0.117520000000
C -0.890921000000 -2.177627000000 0.773212000000
C -0.199036000000 -1.704501000000 -1.497149000000
C -2.044176000000 -2.793251000000 0.299745000000
C -1.356551000000 -2.302436000000 -1.983328000000
C -2.266251000000 -2.845632000000 -1.076113000000
H -2.757223000000 -3.224334000000 0.989646000000
H -1.540511000000 -2.352527000000 -3.048489000000
Br -0.592271000000 -2.069777000000 2.638384000000
Br 1.051701000000 -0.985085000000 -2.729669000000
Br -3.839594000000 -3.673385000000 -1.727855000000
C -3.502636000000 3.314762000000 -0.046856000000
C -4.865813000000 3.676704000000 0.180305000000
C -5.392224000000 4.844601000000 -0.429441000000
C -4.599671000000 5.632099000000 -1.234224000000
C -3.251467000000 5.274128000000 -1.454640000000
H -2.628747000000 5.898795000000 -2.087519000000
C -2.711760000000 4.141197000000 -0.877516000000
H -5.005112000000 6.525182000000 -1.698005000000
H -6.430802000000 5.106039000000 -0.248401000000

H -1.674000000000 3.881689000000 -1.056444000000
O -3.266981000000 0.247859000000 1.976645000000
C -4.025797000000 -0.517139000000 2.920632000000
H -3.339340000000 -1.281213000000 3.281412000000
H -4.355128000000 0.105110000000 3.758656000000
H -4.892066000000 -0.988818000000 2.444969000000

II_D
E(RB3LYP/6-31G(d,p)) = -9336.82439084 A. U.

Temp. 298.150 K 1.00000 Atm.
E + zero-point Energies = -9336.364738
E + thermal Energies = -9336.330013
E + thermal Enthalpies = -9336.329069
E + thermal Free Energies = -9336.437547

Geometry at E(RB3LYP/6-31G(d,p))
Charge: 1 Multiplicity: 1
C 3.896300000000 1.967374000000 0.176735000000
C 5.014206000000 1.110364000000 0.074608000000
C 6.315977000000 1.650455000000 -0.000483000000
C 6.493566000000 3.017017000000 0.025902000000
H 7.177396000000 0.999797000000 -0.074491000000
H 7.501182000000 3.419619000000 -0.030207000000
C 2.608226000000 1.427245000000 0.246494000000
C 1.460776000000 1.000823000000 0.320025000000
C 0.074153000000 0.741720000000 0.390898000000
O -0.794031000000 1.609330000000 0.386104000000
C -0.612099000000 -2.849759000000 0.818164000000
C -0.385203000000 -0.692247000000 0.502896000000
N -1.830580000000 -2.409404000000 0.652761000000
N 0.306100000000 -1.830998000000 0.726445000000
C 1.752264000000 -2.135469000000 0.774926000000
H 2.275332000000 -1.214777000000 1.037059000000
C -0.113943000000 -4.202822000000 1.211093000000
H 0.287862000000 -4.745202000000 0.344684000000
H -0.924122000000 -4.787543000000 1.648762000000
C 2.031185000000 -3.270601000000 1.837167000000
H 2.390960000000 -2.835050000000 2.770688000000
O 0.859522000000 -3.988912000000 2.225891000000
C 3.097592000000 -4.176299000000 1.170887000000
H 4.090572000000 -3.942731000000 1.574706000000
H 2.900378000000 -5.225552000000 1.411400000000

C 2.250186000000 -2.714058000000 -0.536963000000
C 3.017773000000 -3.860131000000 -0.304190000000
C 2.042245000000 -2.227296000000 -1.827569000000
C 3.599547000000 -4.536988000000 -1.377700000000
C 2.620059000000 -2.911894000000 -2.897924000000
H 1.429262000000 -1.348549000000 -2.007805000000
C 3.394927000000 -4.055814000000 -2.672900000000
H 4.192172000000 -5.432015000000 -1.212268000000
H 2.466534000000 -2.556027000000 -3.911575000000
H 3.834749000000 -4.579785000000 -3.515714000000
N -1.675197000000 -1.065726000000 0.451554000000
C -2.828006000000 -0.279912000000 0.134257000000
C -3.679965000000 0.163342000000 1.148461000000
C -3.131293000000 0.000799000000 -1.201273000000
C -4.832625000000 0.876269000000 0.835977000000
C -4.276526000000 0.715000000000 -1.531025000000
C -5.118695000000 1.142237000000 -0.503114000000
H -5.491947000000 1.221376000000 1.621623000000
H -4.509626000000 0.934037000000 -2.564723000000
Br -3.246118000000 -0.182666000000 2.957667000000
Br -1.956567000000 -0.577676000000 -2.577701000000
Br -6.685134000000 2.113100000000 -0.939736000000
C 4.078978000000 3.402251000000 0.202690000000
C 5.407333000000 3.923004000000 0.125185000000
C 5.611558000000 5.326953000000 0.149781000000
C 4.541557000000 6.187971000000 0.245980000000
C 3.229371000000 5.670313000000 0.321010000000
H 2.388666000000 6.352948000000 0.396040000000
C 2.998531000000 4.308854000000 0.300855000000
H 4.701221000000 7.260906000000 0.264468000000
H 6.626718000000 5.708663000000 0.091331000000
H 1.984475000000 3.928023000000 0.359198000000
O 4.753385000000 -0.214828000000 0.054106000000
C 5.831542000000 -1.130660000000 -0.171174000000
H 5.367522000000 -2.112612000000 -0.253810000000
H 6.354985000000 -0.900137000000 -1.104137000000
H 6.539090000000 -1.121997000000 0.664503000000

III_ A_i

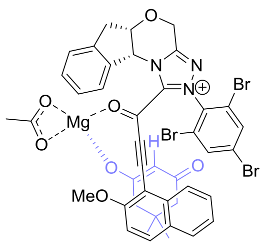

III

E(RB3LYP/6-31G(d,p)) = -10227.5228534 A. U.

Temp. 298.150 K 1.00000 Atm.

E + zero-point Energies = -10226.832762

E + thermal Energies = -10226.779821

E + thermal Enthalpies = -10226.778877

E + thermal Free Energies = -10226.928725

Geometry at E(RB3LYP/6-31G(d,p))

Charge: 1 Multiplicity: 1

C -3.351654000000 -2.706544000000 -0.503470000000
C -1.857142000000 -1.107130000000 -0.576885000000
N -2.208452000000 -3.313707000000 -0.324133000000
N -3.185156000000 -1.352732000000 -0.654227000000
C -4.371008000000 -0.469352000000 -0.771236000000
H -4.034493000000 0.457883000000 -1.232436000000
C -4.726614000000 -3.263384000000 -0.689122000000
H -5.322194000000 -3.149244000000 0.226525000000
H -4.671079000000 -4.321998000000 -0.946163000000
C -5.486994000000 -1.170249000000 -1.639578000000
H -5.466519000000 -0.788512000000 -2.661676000000
O -5.287356000000 -2.577277000000 -1.800173000000
C -6.814515000000 -0.825158000000 -0.919076000000
H -7.315834000000 0.002220000000 -1.436268000000
H -7.496032000000 -1.680675000000 -0.959248000000
C -5.029529000000 -0.233487000000 0.575354000000
C -6.411160000000 -0.425082000000 0.480137000000
C -4.427919000000 0.150526000000 1.774160000000
C -7.219425000000 -0.221787000000 1.599003000000
C -5.240296000000 0.345116000000 2.891919000000
H -3.351628000000 0.271066000000 1.849863000000
C -6.625422000000 0.164038000000 2.802041000000
H -8.294034000000 -0.366409000000 1.539021000000
H -4.795472000000 0.640476000000 3.836609000000
H -7.244194000000 0.320751000000 3.679970000000
N -1.282800000000 -2.306774000000 -0.371935000000

C 0.068472000000 -2.606141000000 -0.019674000000
C 0.900822000000 -3.269943000000 -0.928341000000
C 0.521701000000 -2.334429000000 1.278727000000
C 2.173387000000 -3.673765000000 -0.541670000000
C 1.808388000000 -2.694221000000 1.667340000000
C 2.606796000000 -3.380598000000 0.753129000000
H 2.815484000000 -4.200129000000 -1.235622000000
H 2.177050000000 -2.421952000000 2.646344000000
Br 0.297355000000 -3.608777000000 -2.690682000000
Br -0.630204000000 -1.499804000000 2.525025000000
Br 4.344338000000 -3.934380000000 1.276347000000
C 2.325611000000 1.512598000000 -1.883331000000
C 2.188892000000 2.794211000000 -2.499538000000
C 3.320351000000 3.426706000000 -3.060440000000
C 4.553396000000 2.817030000000 -2.992440000000
H 3.220701000000 4.386668000000 -3.548847000000
H 5.412382000000 3.316121000000 -3.432752000000
C 1.169308000000 0.894854000000 -1.434546000000
C 0.094571000000 0.364966000000 -1.125045000000
C -1.224731000000 0.224910000000 -0.730125000000
C 3.633037000000 0.898475000000 -1.773768000000
C 4.754090000000 1.564633000000 -2.353589000000
C 6.041854000000 0.982694000000 -2.255896000000
C 6.221981000000 -0.210538000000 -1.587434000000
C 5.117742000000 -0.851104000000 -0.990379000000
H 5.260145000000 -1.770923000000 -0.433339000000
C 3.847089000000 -0.309363000000 -1.081836000000
H 7.210881000000 -0.649142000000 -1.506493000000
H 6.885543000000 1.498550000000 -2.704936000000
H 3.012817000000 -0.797293000000 -0.592043000000
O 0.957850000000 3.308703000000 -2.544585000000
C 0.729877000000 4.578186000000 -3.193586000000
H -0.335042000000 4.765066000000 -3.073114000000
H 0.996045000000 4.514237000000 -4.252677000000
H 1.308145000000 5.365935000000 -2.702285000000
O -1.994351000000 1.219825000000 -0.546482000000
Mg -1.591220000000 3.197730000000 -0.248576000000
O -0.089660000000 3.549959000000 0.832915000000
C 0.931935000000 3.076481000000 1.486976000000
C 1.060500000000 1.752576000000 1.843581000000
C 2.023217000000 4.071586000000 1.823786000000
C 2.226981000000 1.220636000000 2.507378000000
H 0.252084000000 1.060216000000 1.645138000000
C 2.925588000000 3.657152000000 3.009562000000

H 1.548760000000 5.041495000000 2.011353000000
H 2.638317000000 4.205362000000 0.920004000000
C 3.368097000000 2.192184000000 2.801530000000
H 4.060258000000 2.137742000000 1.947299000000
H 3.917370000000 1.812063000000 3.668737000000
C 2.153518000000 3.795210000000 4.338017000000
H 1.849025000000 4.834244000000 4.504124000000
H 2.783095000000 3.493555000000 5.181747000000
H 1.252861000000 3.174617000000 4.353676000000
C 4.161089000000 4.572100000000 3.054946000000
H 3.870336000000 5.619847000000 3.189928000000
H 4.745498000000 4.501400000000 2.130368000000
H 4.818241000000 4.300093000000 3.887710000000
O 2.343150000000 0.019980000000 2.791246000000
O -3.417999000000 4.079691000000 -0.074675000000
C -3.223258000000 4.593717000000 -1.224072000000
C -4.225905000000 5.544757000000 -1.819901000000
H -5.215026000000 5.378040000000 -1.392750000000
H -4.246955000000 5.447796000000 -2.906661000000
H -3.917607000000 6.568508000000 -1.580838000000
O -2.143861000000 4.313779000000 -1.860128000000

TS1(i)

Imaginary Frequency = -254.0239 cm^{-1}

E(RB3LYP/6-31G(d,p)) = -10227.5152368

Temp. 298.150 K 1.00000 Atm.

E + zero-point Energies = -10226.825403

E + thermal Energies = -10226.773537

E + thermal Enthalpies = -10226.772593

E + thermal Free Energies = -10226.917958

Geometry at E(RB3LYP/6-31G(d,p))

Charge: 1 Multiplicity: 1

C 3.390782000000 -2.671890000000 0.610528000000
C 1.904781000000 -1.061861000000 0.474319000000
N 2.275427000000 -3.282974000000 0.321012000000
N 3.213798000000 -1.312733000000 0.708085000000
C 4.377204000000 -0.430920000000 0.958353000000
H 3.984816000000 0.503381000000 1.356581000000
C 4.733633000000 -3.224202000000 0.964963000000
H 5.434886000000 -3.121920000000 0.125881000000
H 4.647915000000 -4.279199000000 1.228358000000
C 5.371983000000 -1.116348000000 1.973133000000

H 5.217979000000 -0.721656000000 2.978881000000
O 5.160838000000 -2.522969000000 2.125522000000
C 6.781654000000 -0.774565000000 1.427238000000
H 7.207828000000 0.064127000000 1.991613000000
H 7.455126000000 -1.625341000000 1.570372000000
C 5.204738000000 -0.213209000000 -0.294531000000
C 6.562780000000 -0.399241000000 -0.019404000000
C 4.762987000000 0.149966000000 -1.566962000000
C 7.508411000000 -0.213600000000 -1.028305000000
C 5.712301000000 0.327383000000 -2.574332000000
H 3.704958000000 0.269846000000 -1.778760000000
C 7.074296000000 0.150161000000 -2.304445000000
H 8.566411000000 -0.355045000000 -0.828144000000
H 5.392930000000 0.606722000000 -3.573163000000
H 7.800905000000 0.292832000000 -3.098172000000
N 1.354407000000 -2.267859000000 0.233702000000
C 0.030536000000 -2.576087000000 -0.192841000000
C -0.837996000000 -3.269248000000 0.659222000000
C -0.385960000000 -2.257465000000 -1.492818000000
C -2.103012000000 -3.642961000000 0.218155000000
C -1.656814000000 -2.596328000000 -1.938659000000
C -2.498962000000 -3.294933000000 -1.072778000000
H -2.772879000000 -4.179462000000 0.876978000000
H -1.993766000000 -2.301970000000 -2.921928000000
Br -0.312649000000 -3.679186000000 2.430848000000
Br 0.781213000000 -1.319891000000 -2.664074000000
Br -4.244210000000 -3.749895000000 -1.647919000000
C -2.256498000000 1.449075000000 1.601396000000
C -2.034227000000 2.642847000000 2.323525000000
C -3.000634000000 3.095786000000 3.253198000000
C -4.166996000000 2.389214000000 3.437338000000
H -2.824839000000 3.997516000000 3.824572000000
H -4.899071000000 2.750808000000 4.154025000000
C -1.205542000000 0.968595000000 0.769525000000
C -0.062447000000 0.438455000000 0.697497000000
C 1.273181000000 0.273801000000 0.483409000000
C -3.491023000000 0.726014000000 1.767082000000
C -4.453128000000 1.205313000000 2.710572000000
C -5.669624000000 0.497676000000 2.889330000000
C -5.938770000000 -0.634756000000 2.152846000000
C -4.996034000000 -1.097531000000 1.208582000000
H -5.215505000000 -1.971451000000 0.603479000000
C -3.797570000000 -0.437768000000 1.023187000000
H -6.874975000000 -1.166383000000 2.287882000000

H -6.388422000000 0.870604000000 3.613302000000
H -3.097633000000 -0.790992000000 0.276195000000
O -0.871081000000 3.293273000000 2.094209000000
C -0.569789000000 4.483543000000 2.846118000000
H 0.409828000000 4.804874000000 2.497114000000
H -0.534962000000 4.264294000000 3.917782000000
H -1.312626000000 5.263041000000 2.648563000000
O 2.098015000000 1.266241000000 0.352027000000
Mg 1.648856000000 3.128984000000 -0.146835000000
O 0.006435000000 3.266282000000 -1.145663000000
C -1.200555000000 2.896615000000 -1.292837000000
C -1.561489000000 1.531192000000 -1.455133000000
C -2.281749000000 3.948221000000 -1.240872000000
C -2.921730000000 1.150944000000 -1.871346000000
H -0.785996000000 0.834679000000 -1.746374000000
C -3.521692000000 3.638006000000 -2.112656000000
H -1.834065000000 4.911839000000 -1.502977000000
H -2.589972000000 4.022063000000 -0.185420000000
C -4.007915000000 2.210599000000 -1.770825000000
H -4.382421000000 2.193220000000 -0.736490000000
H -4.836845000000 1.901958000000 -2.414051000000
C -3.154639000000 3.733894000000 -3.607006000000
H -2.807515000000 4.741319000000 -3.858904000000
H -4.024960000000 3.513609000000 -4.233069000000
H -2.362433000000 3.029698000000 -3.883238000000
C -4.631274000000 4.653585000000 -1.794796000000
H -4.304334000000 5.675220000000 -2.016286000000
H -4.921061000000 4.613225000000 -0.738881000000
H -5.524688000000 4.453521000000 -2.394934000000
O -3.164554000000 0.014115000000 -2.272394000000
O 3.249139000000 4.215377000000 -0.756476000000
C 3.130510000000 4.893463000000 0.317114000000
C 4.038249000000 6.061131000000 0.591795000000
H 4.221767000000 6.157546000000 1.663113000000
H 3.539860000000 6.976328000000 0.253644000000
H 4.973991000000 5.957627000000 0.042164000000
O 2.205447000000 4.585096000000 1.152212000000

TS1(ii)

Imaginary Frequency = -283.2260 cm^{-1}

E(RB3LYP/6-31G(d,p)) = -10227.5117001

Temp. 298.150 K 1.00000 Atm.

E + zero-point Energies = -10226.821985

E + thermal Energies = -10226.770310
E + thermal Enthalpies = -10226.769366
E + thermal Free Energies = -10226.913528

Geometry at E(RB3LYP/6-31G(d,p))
Charge: 1 Multiplicity: 1

C	-3.143721000000	-0.332946000000	-2.499936000000
C	-1.692980000000	0.138279000000	-0.922132000000
N	-2.200466000000	-1.219376000000	-2.654783000000
N	-2.880950000000	0.505002000000	-1.441823000000
C	-3.834880000000	1.565587000000	-1.053422000000
H	-3.273596000000	2.298077000000	-0.469567000000
C	-4.319443000000	0.009948000000	-3.357374000000
H	-5.257663000000	-0.326934000000	-2.897619000000
H	-4.218499000000	-0.449216000000	-4.341402000000
C	-4.452922000000	2.216402000000	-2.355307000000
H	-3.941572000000	3.150751000000	-2.592675000000
O	-4.268691000000	1.421642000000	-3.532137000000
C	-5.945915000000	2.438658000000	-2.014894000000
H	-6.115280000000	3.486878000000	-1.738763000000
H	-6.564484000000	2.241376000000	-2.895943000000
C	-5.036363000000	1.026093000000	-0.295555000000
C	-6.222271000000	1.521848000000	-0.847807000000
C	-5.050751000000	0.188926000000	0.821503000000
C	-7.451850000000	1.173082000000	-0.288451000000
C	-6.284231000000	-0.164626000000	1.369748000000
H	-4.129197000000	-0.178036000000	1.258033000000
C	-7.474802000000	0.323526000000	0.818993000000
H	-8.378288000000	1.552855000000	-0.709106000000
H	-6.316027000000	-0.820361000000	2.233960000000
H	-8.426211000000	0.040035000000	1.258339000000
N	-1.300221000000	-0.920990000000	-1.650202000000
C	-0.216553000000	-1.817952000000	-1.413812000000
C	0.857338000000	-1.877525000000	-2.311990000000
C	-0.263690000000	-2.722873000000	-0.342568000000
C	1.879485000000	-2.806354000000	-2.138441000000
C	0.746199000000	-3.661037000000	-0.161134000000
C	1.812688000000	-3.688186000000	-1.059981000000
H	2.709069000000	-2.840945000000	-2.832588000000
H	0.705634000000	-4.355366000000	0.667868000000
Br	0.929528000000	-0.688208000000	-3.783130000000
Br	-1.698699000000	-2.681439000000	0.902338000000
Br	3.215839000000	-4.929185000000	-0.787173000000
C	1.997580000000	3.147810000000	0.133462000000

C	2.123039000000	4.188512000000	1.077710000000
C	2.939315000000	5.311022000000	0.802389000000
C	3.614494000000	5.389299000000	-0.390558000000
H	3.034159000000	6.105459000000	1.530838000000
H	4.240318000000	6.253994000000	-0.592703000000
C	1.216516000000	2.007328000000	0.463906000000
C	0.158908000000	1.360127000000	0.181289000000
C	-1.069541000000	0.801709000000	0.256387000000
C	2.704087000000	3.228400000000	-1.124438000000
C	3.522881000000	4.372188000000	-1.379128000000
C	4.226869000000	4.470309000000	-2.605768000000
C	4.135171000000	3.475424000000	-3.554819000000
C	3.324654000000	2.348039000000	-3.303957000000
H	3.243619000000	1.562857000000	-4.049851000000
C	2.625224000000	2.226493000000	-2.119835000000
H	4.679080000000	3.557181000000	-4.490122000000
H	4.843641000000	5.346782000000	-2.782988000000
H	1.995462000000	1.360262000000	-1.959029000000
O	1.392949000000	4.059662000000	2.199005000000
C	1.690247000000	4.895704000000	3.327242000000
H	1.054361000000	4.530088000000	4.132945000000
H	1.442044000000	5.941194000000	3.119827000000
H	2.742171000000	4.791669000000	3.604619000000
O	-1.876527000000	0.902523000000	1.278328000000
Mg	-1.241521000000	-0.176525000000	2.810851000000
O	0.607034000000	-0.651574000000	2.353587000000
C	1.796614000000	-0.282201000000	2.107032000000
C	2.195298000000	1.077672000000	2.197980000000
C	2.774687000000	-1.324508000000	1.617983000000
C	3.622249000000	1.439169000000	2.121157000000
H	1.582731000000	1.738946000000	2.801717000000
C	4.253896000000	-1.044318000000	1.971334000000
H	2.669836000000	-1.365649000000	0.523618000000
H	2.452027000000	-2.298102000000	1.998964000000
C	4.582240000000	0.405972000000	1.551423000000
H	5.595330000000	0.692075000000	1.847266000000
H	4.533470000000	0.488653000000	0.454950000000
C	5.150170000000	-2.026055000000	1.198512000000
H	4.901507000000	-3.064293000000	1.442158000000
H	6.204470000000	-1.866018000000	1.445003000000
H	5.036409000000	-1.903776000000	0.115518000000
C	4.479803000000	-1.226351000000	3.485496000000
H	4.255910000000	-2.253608000000	3.791826000000
H	3.850914000000	-0.556010000000	4.081067000000

H	5.521991000000	-1.019623000000	3.747875000000
O	4.006682000000	2.553806000000	2.461967000000
C	-3.375463000000	-0.945270000000	5.925430000000
H	-4.381614000000	-1.256314000000	5.639555000000
H	-2.906037000000	-1.782922000000	6.452889000000
H	-3.407541000000	-0.087557000000	6.597260000000
O	-2.712250000000	-1.316356000000	3.628620000000
O	-1.682018000000	0.297594000000	4.727823000000
C	-2.557488000000	-0.631958000000	4.703150000000

TS1'(i)

Imaginary Frequency = -256.6398 cm^{-1}

E(RB3LYP/6-31G(d,p)) = -10227.5112208 A.U.

Temp. 298.150 K 1.00000 Atm.

E + zero-point Energies = -10226.821359

E + thermal Energies = -10226.769557

E + thermal Enthalpies = -10226.768613

E + thermal Free Energies = -10226.914152

Geometry at E(RB3LYP/6-31G(d,p))

Charge: 1 Multiplicity: 1

C -2.540998000000 1.185443000000 -1.046911000000
C -2.438741000000 1.985313000000 -2.208238000000
C -3.567318000000 2.167142000000 -3.043441000000
C -4.771459000000 1.589492000000 -2.715195000000
H -3.486770000000 2.762622000000 -3.942702000000
H -5.629034000000 1.742722000000 -3.364278000000
C -1.354039000000 0.962312000000 -0.291041000000
C -0.207682000000 0.433143000000 -0.233881000000
C 1.144993000000 0.340272000000 -0.094529000000
C 3.210464000000 -2.067965000000 1.660971000000
C 1.778126000000 -0.814334000000 0.568822000000
N 2.173557000000 -2.838988000000 1.478202000000
N 3.016399000000 -0.821268000000 1.118788000000
C 4.107934000000 0.184673000000 1.151827000000
H 3.638941000000 1.161627000000 1.052040000000
C 4.453555000000 -2.298440000000 2.457313000000
H 5.307829000000 -2.514853000000 1.801389000000
H 4.311052000000 -3.136305000000 3.140982000000
C 4.915654000000 0.069357000000 2.506239000000
H 4.613047000000 0.863694000000 3.190243000000
O 4.645309000000 -1.127555000000 3.238720000000

C 6.404288000000 0.199455000000 2.093139000000
H 6.768216000000 1.208879000000 2.319806000000
H 7.016961000000 -0.493900000000 2.678199000000
C 5.139258000000 -0.066095000000 0.068112000000
C 6.429305000000 -0.061154000000 0.606549000000
C 4.921553000000 -0.251149000000 -1.297771000000
C 7.531882000000 -0.251975000000 -0.227321000000
C 6.027055000000 -0.449148000000 -2.125824000000
H 3.917099000000 -0.240932000000 -1.708627000000
C 7.321714000000 -0.449988000000 -1.592989000000
H 8.539235000000 -0.250176000000 0.178425000000
H 5.882812000000 -0.599039000000 -3.191095000000
H 8.171975000000 -0.605114000000 -2.249852000000
N 1.288725000000 -2.049568000000 0.788636000000
C 0.087208000000 -2.656566000000 0.313884000000
C -1.073473000000 -2.639205000000 1.093833000000
C 0.109918000000 -3.379498000000 -0.885405000000
C -2.197721000000 -3.355418000000 0.692895000000
C -1.009492000000 -4.089340000000 -1.304416000000
C -2.150056000000 -4.076148000000 -0.500036000000
H -3.096060000000 -3.343699000000 1.295238000000
H -0.991371000000 -4.646074000000 -2.232086000000
Br -1.147100000000 -1.571531000000 2.651386000000
Br 1.678897000000 -3.383479000000 -1.951302000000
Br -3.675796000000 -5.056420000000 -1.048859000000
C -3.807468000000 0.597543000000 -0.689552000000
C -4.934553000000 0.803895000000 -1.546011000000
C -6.186851000000 0.228471000000 -1.209528000000
C -6.331365000000 -0.518516000000 -0.061817000000
C -5.220440000000 -0.716025000000 0.787334000000
H -5.340131000000 -1.291445000000 1.700395000000
C -3.986229000000 -0.176429000000 0.482665000000
H -7.293136000000 -0.950456000000 0.194409000000
H -7.031182000000 0.392890000000 -1.872828000000
H -3.151500000000 -0.332423000000 1.154771000000
O -1.234517000000 2.538548000000 -2.478894000000
C -1.066642000000 3.291075000000 -3.696204000000
H -0.028163000000 3.615836000000 -3.694919000000
H -1.266628000000 2.660861000000 -4.568145000000
H -1.727546000000 4.163621000000 -3.705560000000
O 1.967537000000 1.254627000000 -0.509380000000
Mg 1.535364000000 3.162275000000 -0.811058000000
O 0.110652000000 3.754713000000 0.348853000000
C -1.008563000000 3.493304000000 0.890782000000

C -1.252262000000 2.282455000000 1.598442000000
C -2.130329000000 4.487873000000 0.724104000000
C -2.444826000000 2.136495000000 2.455087000000
H -0.398241000000 1.701758000000 1.920275000000
C -3.105498000000 4.561475000000 1.924155000000
H -1.689598000000 5.464016000000 0.499678000000
H -2.688210000000 4.180383000000 -0.175773000000
C -3.581325000000 3.126247000000 2.247720000000
H -4.193032000000 2.751506000000 1.414127000000
H -4.210913000000 3.102680000000 3.141582000000
C -2.394297000000 5.177107000000 3.145974000000
H -2.049858000000 6.192905000000 2.925862000000
H -3.075662000000 5.232638000000 4.000693000000
H -1.523171000000 4.590150000000 3.456892000000
C -4.312022000000 5.434529000000 1.543253000000
H -3.996953000000 6.453730000000 1.295253000000
H -4.844528000000 5.025113000000 0.677757000000
H -5.021730000000 5.498824000000 2.374104000000
O -2.506266000000 1.244828000000 3.297381000000
O 3.233850000000 4.273261000000 -0.896037000000
C 2.956337000000 4.498907000000 -2.119962000000
C 3.836222000000 5.387313000000 -2.955615000000
H 3.467415000000 6.415911000000 -2.875692000000
H 4.861944000000 5.365487000000 -2.586272000000
H 3.791570000000 5.092962000000 -4.004979000000
O 1.895836000000 3.980297000000 -2.626065000000

Allenolate_ A_i

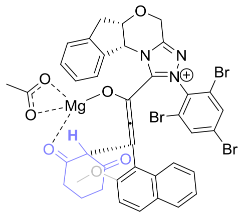

Allenolate

E(RB3LYP/6-31G(d,p)) = -10227.5410639 A.U.

Temp. 298.150 K 1.00000 Atm.
E + zero-point Energies = -10226.848947
E + thermal Energies = -10226.797217
E + thermal Enthalpies = -10226.796272
E + thermal Free Energies= -10226.941362

Geometry at E(RB3LYP/6-31G(d,p))
Charge: 1 Multiplicity: 1
C 3.148233000000 -2.885654000000 0.416093000000
C 1.770356000000 -1.171134000000 0.371262000000
N 2.011163000000 -3.398324000000 0.043283000000
N 3.051336000000 -1.529307000000 0.624702000000
C 4.254588000000 -0.753939000000 1.004631000000
H 3.898657000000 0.166014000000 1.464886000000
C 4.437190000000 -3.552742000000 0.772140000000
H 5.184625000000 -3.420215000000 -0.021476000000
H 4.275379000000 -4.619551000000 0.931653000000
C 5.151078000000 -1.590627000000 1.997241000000
H 4.970692000000 -1.276804000000 3.026860000000
O 4.848701000000 -2.989025000000 2.010854000000
C 6.606162000000 -1.298398000000 1.550472000000
H 7.054375000000 -0.541598000000 2.206306000000
H 7.216248000000 -2.201810000000 1.648219000000
C 5.159454000000 -0.482254000000 -0.182233000000
C 6.486884000000 -0.785666000000 0.134708000000
C 4.809105000000 0.025050000000 -1.433161000000
C 7.492646000000 -0.579515000000 -0.810038000000
C 5.817351000000 0.222728000000 -2.377748000000
H 3.774272000000 0.248509000000 -1.672410000000
C 7.148834000000 -0.075947000000 -2.066194000000
H 8.527501000000 -0.812403000000 -0.576713000000
H 5.567962000000 0.613391000000 -3.359077000000
H 7.922538000000 0.083027000000 -2.810901000000
N 1.158297000000 -2.317205000000 0.007368000000
C -0.189908000000 -2.536242000000 -0.393478000000
C -1.057295000000 -3.244170000000 0.449576000000
C -0.642023000000 -2.129819000000 -1.655898000000
C -2.348569000000 -3.552821000000 0.034940000000
C -1.937614000000 -2.406598000000 -2.075542000000
C -2.774354000000 -3.125342000000 -1.221264000000
H -3.014063000000 -4.100338000000 0.688969000000
H -2.292045000000 -2.057239000000 -3.034947000000
Br -0.494445000000 -3.768098000000 2.178556000000
Br 0.508834000000 -1.153750000000 -2.813483000000
Br -4.554209000000 -3.493754000000 -1.756652000000
C -2.052107000000 1.493421000000 1.558412000000
C -1.650039000000 2.538868000000 2.391366000000
C -2.422720000000 2.891499000000 3.527617000000
C -3.578246000000 2.206061000000 3.817207000000
H -2.108458000000 3.700142000000 4.173982000000

H -4.162722000000 2.486772000000 4.688717000000
C -1.199044000000 1.131445000000 0.379718000000
C -0.074647000000 0.461741000000 0.495636000000
C 1.234845000000 0.198325000000 0.438809000000
C -3.248861000000 0.767089000000 1.859565000000
C -4.029360000000 1.138648000000 3.004002000000
C -5.225966000000 0.431184000000 3.296462000000
C -5.650040000000 -0.602488000000 2.494261000000
C -4.879629000000 -0.977532000000 1.368751000000
H -5.211879000000 -1.787973000000 0.727103000000
C -3.707643000000 -0.320034000000 1.066180000000
H -6.569541000000 -1.132047000000 2.721782000000
H -5.802823000000 0.725738000000 4.168708000000
H -3.129158000000 -0.633649000000 0.206856000000
O -0.505938000000 3.217319000000 2.048496000000
C -0.060193000000 4.282480000000 2.905163000000
H 0.854890000000 4.663902000000 2.455526000000
H 0.151735000000 3.909314000000 3.912175000000
H -0.807639000000 5.081365000000 2.958713000000
O 2.165993000000 1.146322000000 0.469598000000
Mg 1.890049000000 2.986450000000 -0.035456000000
O 0.166020000000 3.400523000000 -0.995205000000
C -1.045041000000 3.152503000000 -1.034151000000
C -1.514967000000 1.704548000000 -1.045130000000
C -2.049088000000 4.264423000000 -1.006755000000
C -2.954746000000 1.508100000000 -1.560172000000
H -0.870841000000 1.155458000000 -1.737995000000
C -3.346976000000 4.006938000000 -1.809803000000
H -1.551878000000 5.191946000000 -1.305135000000
H -2.303331000000 4.368060000000 0.061375000000
C -3.933934000000 2.642377000000 -1.365468000000
H -4.188269000000 2.685539000000 -0.296845000000
H -4.845785000000 2.400860000000 -1.917342000000
C -3.036453000000 3.992768000000 -3.319925000000
H -2.622288000000 4.953741000000 -3.640805000000
H -3.945715000000 3.808695000000 -3.899771000000
H -2.313924000000 3.213767000000 -3.592174000000
C -4.355163000000 5.125112000000 -1.499182000000
H -3.953547000000 6.103405000000 -1.782773000000
H -4.603947000000 5.156131000000 -0.433134000000
H -5.283867000000 4.972430000000 -2.057200000000
O -3.233121000000 0.475108000000 -2.135983000000
O 3.387344000000 3.993876000000 -0.966278000000
C 3.404365000000 4.778785000000 0.038118000000

C 4.345297000000 5.950860000000 0.089782000000
H 5.094543000000 5.882042000000 -0.698076000000
H 4.820830000000 6.001683000000 1.071959000000
H 3.768883000000 6.872803000000 -0.039669000000
O 2.590447000000 4.568029000000 1.009780000000

IV_A_i

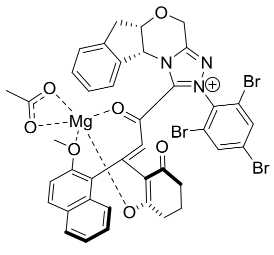

IV

E(RB3LYP) = -10227.5751754

Temperature 298.150 Kelvin. Pressure 1.00000 Atm.
E + zero-point Energies = -10226.882619
E + thermal Energies = -10226.831288
E + thermal Enthalpies = -10226.830343
E + thermal Free Energies = -10226.974064

Charge: 1 Multiplicity: 1
C 2.555198000000 -2.802958000000 1.330150000000
C 1.157446000000 -1.239804000000 0.690157000000
N 1.418189000000 -3.426366000000 1.180050000000
N 2.442723000000 -1.465866000000 1.033042000000
C 3.633984000000 -0.587099000000 1.057722000000
H 3.286353000000 0.441803000000 1.152218000000
C 3.858002000000 -3.276255000000 1.889063000000
H 4.590548000000 -3.449209000000 1.089275000000
H 3.715728000000 -4.203580000000 2.445397000000
C 4.574985000000 -0.992831000000 2.258456000000
H 4.430226000000 -0.312010000000 3.098974000000
O 4.279961000000 -2.279139000000 2.811735000000
C 6.011096000000 -0.900108000000 1.684130000000
H 6.481199000000 0.037492000000 2.005220000000
H 6.626995000000 -1.713668000000 2.080245000000
C 4.498580000000 -0.763114000000 -0.177658000000
C 5.838807000000 -0.932611000000 0.184255000000
C 4.103481000000 -0.729507000000 -1.516040000000
C 6.814017000000 -1.075180000000 -0.803662000000

C 5.082838000000 -0.881704000000 -2.499164000000
H 3.061250000000 -0.592069000000 -1.784747000000
C 6.426641000000 -1.051812000000 -2.145106000000
H 7.858137000000 -1.208204000000 -0.535849000000
H 4.800639000000 -0.862706000000 -3.547050000000
H 7.175844000000 -1.168313000000 -2.922099000000
N 0.552944000000 -2.439039000000 0.772877000000
C -0.740436000000 -2.809716000000 0.292974000000
C -1.808356000000 -2.991464000000 1.176366000000
C -0.924367000000 -3.052039000000 -1.074614000000
C -3.053865000000 -3.398420000000 0.711176000000
C -2.165498000000 -3.453211000000 -1.557500000000
C -3.215820000000 -3.624677000000 -0.656165000000
H -3.878588000000 -3.535024000000 1.398162000000
H -2.307703000000 -3.632623000000 -2.614954000000
Br -1.565834000000 -2.640994000000 3.023271000000
Br 0.519591000000 -2.836182000000 -2.275121000000
Br -4.908726000000 -4.179126000000 -1.304091000000
C -1.686002000000 2.604428000000 1.369570000000
C -0.777043000000 3.221402000000 2.213514000000
C -1.193968000000 4.092082000000 3.244026000000
C -2.536466000000 4.324800000000 3.430666000000
H -0.468909000000 4.588143000000 3.876050000000
H -2.859691000000 4.994834000000 4.221761000000
C -1.190519000000 1.694720000000 0.288304000000
C 0.580377000000 0.067714000000 0.276737000000
C -3.087625000000 2.846276000000 1.548051000000
C -3.513851000000 3.717840000000 2.604272000000
C -4.901484000000 3.953681000000 2.798063000000
C -5.838449000000 3.351269000000 1.992336000000
C -5.421882000000 2.479014000000 0.958685000000
H -6.166902000000 1.997880000000 0.332289000000
C -4.085095000000 2.230589000000 0.741259000000
H -6.896093000000 3.537597000000 2.147962000000
H -5.208336000000 4.619867000000 3.599318000000
H -3.786588000000 1.550618000000 -0.048889000000
O 0.582500000000 3.001829000000 1.962936000000
C 1.485323000000 3.131324000000 3.090871000000
H 1.603632000000 4.181042000000 3.368614000000
H 2.444189000000 2.749607000000 2.751004000000
H 1.100349000000 2.554848000000 3.935321000000
O 1.422059000000 0.839587000000 -0.315813000000
Mg 1.460793000000 2.865995000000 -0.054132000000
O -0.037565000000 4.144308000000 -0.751212000000

C -0.969980000000 3.552423000000 -1.347685000000
C -1.184067000000 2.141824000000 -1.031063000000
C -1.709645000000 4.186779000000 -2.481834000000
C -1.355324000000 1.226437000000 -2.206826000000
C -1.591817000000 3.297138000000 -3.759321000000
H -1.316452000000 5.190326000000 -2.660213000000
H -2.769135000000 4.266689000000 -2.203014000000
C -2.052820000000 1.862123000000 -3.410319000000
H -3.129465000000 1.874491000000 -3.183931000000
H -1.911059000000 1.187660000000 -4.259117000000
C -0.135245000000 3.281637000000 -4.264152000000
H 0.205220000000 4.294181000000 -4.501247000000
H -0.056285000000 2.679361000000 -5.174578000000
H 0.565685000000 2.864538000000 -3.532899000000
C -2.507418000000 3.878864000000 -4.849433000000
H -2.202126000000 4.896899000000 -5.111211000000
H -3.551916000000 3.911339000000 -4.521821000000
H -2.457252000000 3.271797000000 -5.758932000000
O -1.047225000000 0.047205000000 -2.195317000000
C -0.710664000000 0.396321000000 0.663635000000
H -1.208970000000 -0.180134000000 1.435202000000
C 5.235210000000 3.455516000000 -0.774874000000
H 5.660417000000 2.466991000000 -0.981700000000
H 5.753385000000 3.874209000000 0.089431000000
H 5.382618000000 4.081776000000 -1.655112000000
C 3.766761000000 3.292405000000 -0.484870000000
O 3.373976000000 3.006187000000 0.706048000000
O 2.906318000000 3.402087000000 -1.411914000000

IV-V_ A_i

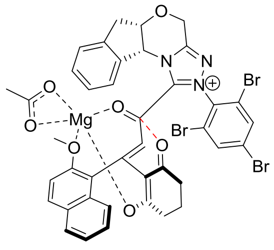

IV-V

E(RB3LYP/6-31G(d,p)) = -10227.5649095 A.U.

E + zero-point Energies = -10226.871614
E + thermal Energies = -10226.821654
E + thermal Enthalpies = -10226.820710

E + thermal Free Energies = -10226.959572

Charge: 1 Multiplicity: 1

C -2.188613000000 -2.927999000000 -1.398694000000
C -1.029644000000 -1.251799000000 -0.594710000000
N -0.986520000000 -3.408431000000 -1.238438000000
N -2.259017000000 -1.608141000000 -1.018610000000
C -3.544950000000 -0.866472000000 -1.111646000000
H -3.307240000000 0.196337000000 -1.174526000000
C -3.387880000000 -3.528266000000 -2.056603000000
H -4.147571000000 -3.812359000000 -1.316227000000
H -3.100240000000 -4.411319000000 -2.628437000000
C -4.346766000000 -1.338360000000 -2.390124000000
H -4.237042000000 -0.602485000000 -3.188017000000
O -3.855352000000 -2.548338000000 -2.973845000000
C -5.817053000000 -1.459435000000 -1.914698000000
H -6.391371000000 -0.588182000000 -2.252224000000
H -6.286094000000 -2.338790000000 -2.367691000000
C -4.467035000000 -1.174868000000 0.052593000000
C -5.744866000000 -1.508891000000 -0.407445000000
C -4.177625000000 -1.118433000000 1.416588000000
C -6.759082000000 -1.802643000000 0.505019000000
C -5.193593000000 -1.421086000000 2.324302000000
H -3.187176000000 -0.839284000000 1.760481000000
C -6.473521000000 -1.761615000000 1.870962000000
H -7.755092000000 -2.064416000000 0.160018000000
H -4.991572000000 -1.387600000000 3.390191000000
H -7.253207000000 -1.994532000000 2.589547000000
N -0.271001000000 -2.351872000000 -0.731456000000
C 1.122592000000 -2.546206000000 -0.478309000000
C 2.056558000000 -2.279265000000 -1.485314000000
C 1.555828000000 -3.089910000000 0.737093000000
C 3.414186000000 -2.497812000000 -1.273883000000
C 2.909089000000 -3.319035000000 0.962389000000
C 3.824958000000 -3.010736000000 -0.043970000000
H 4.132237000000 -2.283757000000 -2.054505000000
H 3.241928000000 -3.734946000000 1.904305000000
Br 1.472312000000 -1.645865000000 -3.176502000000
Br 0.304011000000 -3.527389000000 2.084209000000
Br 5.671071000000 -3.310218000000 0.262495000000
C 1.575956000000 3.110577000000 -0.795017000000
C 0.826581000000 4.080743000000 -1.445854000000
C 1.431906000000 5.224786000000 -2.013115000000
C 2.794977000000 5.383348000000 -1.932134000000

H 0.830352000000 5.984062000000 -2.495508000000
H 3.259149000000 6.264658000000 -2.364775000000
C 0.877459000000 1.959569000000 -0.152270000000
C -0.672331000000 0.139886000000 -0.116296000000
C 2.995943000000 3.268579000000 -0.703608000000
C 3.612773000000 4.422402000000 -1.288112000000
C 5.022893000000 4.576071000000 -1.209769000000
C 5.801291000000 3.629278000000 -0.586785000000
C 5.196353000000 2.481809000000 -0.020578000000
H 5.817432000000 1.732920000000 0.461749000000
C 3.832336000000 2.303406000000 -0.077926000000
H 6.877726000000 3.755337000000 -0.531055000000
H 5.475589000000 5.457613000000 -1.654742000000
H 3.385158000000 1.414011000000 0.351801000000
O -0.560919000000 3.913367000000 -1.487246000000
C -1.289462000000 4.608981000000 -2.529896000000
H -1.367891000000 5.673731000000 -2.295915000000
H -2.276551000000 4.151224000000 -2.546294000000
H -0.786624000000 4.466868000000 -3.489195000000
O -1.668463000000 0.797720000000 0.341569000000
Mg -1.693798000000 2.901617000000 0.079501000000
O -0.488304000000 3.822359000000 1.522441000000
C 0.303509000000 3.014403000000 2.082103000000
C 0.801052000000 1.908266000000 1.316283000000
C 0.585996000000 3.067867000000 3.559525000000
C 0.710068000000 0.668998000000 1.962108000000
C 0.270372000000 1.700128000000 4.242937000000
H -0.005426000000 3.868368000000 4.010761000000
H 1.649429000000 3.303182000000 3.698255000000
C 0.969092000000 0.558995000000 3.444961000000
H 2.053252000000 0.614634000000 3.609724000000
H 0.631413000000 -0.421398000000 3.792041000000
C -1.252483000000 1.462820000000 4.260930000000
H -1.763414000000 2.268581000000 4.796529000000
H -1.485542000000 0.521937000000 4.770254000000
H -1.680109000000 1.409490000000 3.254335000000
C 0.803791000000 1.717495000000 5.683293000000
H 0.322013000000 2.509924000000 6.264964000000
H 1.884926000000 1.889391000000 5.709914000000
H 0.600033000000 0.765996000000 6.185459000000
O 0.334390000000 -0.386208000000 1.317472000000
C 0.341421000000 0.941258000000 -0.864327000000
H 0.504394000000 0.788338000000 -1.925094000000
C -5.557928000000 3.161155000000 -0.479547000000

H -5.832443000000 3.281829000000 -1.528797000000
H -5.980713000000 3.963518000000 0.125947000000
H -5.975055000000 2.212496000000 -0.122944000000
C -4.059730000000 3.114928000000 -0.322577000000
O -3.324542000000 2.629170000000 -1.253094000000
O -3.514140000000 3.527969000000 0.750308000000

V_ A_i

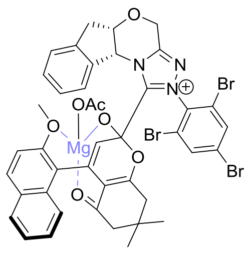

V

E(RB3LYP/6-31G(d,p)) = -10227.5666918 A.U.

Temp. 298.150 K 1.00000 Atm.
E + zero-point Energies = -10226.872474
E + thermal Energies = -10226.822091
E + thermal Enthalpies = -10226.821147
E + thermal Free Energies = -10226.961491

Geometry at E(RB3LYP/6-31G(d,p))
Charge: 1 Multiplicity: 1
C -2.401111000000 -2.855674000000 -1.412416000000
C -1.199159000000 -1.241112000000 -0.552990000000
N -1.231419000000 -3.396603000000 -1.210502000000
N -2.422838000000 -1.532768000000 -1.027278000000
C -3.667094000000 -0.722593000000 -1.148248000000
H -3.352499000000 0.318194000000 -1.221810000000
C -3.604577000000 -3.396173000000 -2.112394000000
H -4.396213000000 -3.659152000000 -1.397706000000
H -3.338209000000 -4.282739000000 -2.689137000000
C -4.483768000000 -1.177157000000 -2.425887000000
H -4.369098000000 -0.435033000000 -3.217040000000
O -4.006344000000 -2.384339000000 -3.025372000000
C -5.955567000000 -1.287433000000 -1.948511000000
H -6.534437000000 -0.432952000000 -2.318743000000
H -6.420179000000 -2.184397000000 -2.371355000000
C -4.609452000000 -0.957850000000 0.014946000000
C -5.890212000000 -1.283770000000 -0.440723000000

C -4.329381000000 -0.844913000000 1.377427000000
C -6.916134000000 -1.520254000000 0.475528000000
C -5.356961000000 -1.088990000000 2.289048000000
H -3.337334000000 -0.563003000000 1.714694000000
C -6.639443000000 -1.427158000000 1.840404000000
H -7.915232000000 -1.774875000000 0.134112000000
H -5.163287000000 -1.010617000000 3.354198000000
H -7.428610000000 -1.614344000000 2.562054000000
N -0.489881000000 -2.377521000000 -0.664589000000
C 0.899979000000 -2.581223000000 -0.399114000000
C 1.837369000000 -2.349419000000 -1.414143000000
C 1.340705000000 -3.026108000000 0.853467000000
C 3.201000000000 -2.468819000000 -1.167401000000
C 2.701961000000 -3.154783000000 1.115358000000
C 3.617204000000 -2.848872000000 0.108055000000
H 3.920003000000 -2.265231000000 -1.949901000000
H 3.040738000000 -3.483449000000 2.089178000000
Br 1.250936000000 -1.868066000000 -3.152536000000
Br 0.094142000000 -3.455403000000 2.208606000000
Br 5.474128000000 -2.942703000000 0.481227000000
C 1.926846000000 2.722439000000 -0.793084000000
C 1.388942000000 3.803054000000 -1.476309000000
C 2.224406000000 4.781047000000 -2.065651000000
C 3.591502000000 4.647168000000 -1.987883000000
H 1.799981000000 5.639062000000 -2.569973000000
H 4.228478000000 5.401226000000 -2.440821000000
C 1.020761000000 1.743028000000 -0.131452000000
C -0.734483000000 0.117272000000 -0.026595000000
C 3.345853000000 2.563139000000 -0.723040000000
C 4.192429000000 3.541069000000 -1.337373000000
C 5.602177000000 3.367452000000 -1.291973000000
C 6.158448000000 2.263958000000 -0.687696000000
C 5.322757000000 1.285466000000 -0.096661000000
H 5.759124000000 0.396769000000 0.350393000000
C 3.954362000000 1.435047000000 -0.106115000000
H 7.236008000000 2.136506000000 -0.667781000000
H 6.234367000000 4.119481000000 -1.755606000000
H 3.325902000000 0.674658000000 0.344001000000
O -0.001145000000 3.893283000000 -1.549821000000
C -0.564033000000 4.812934000000 -2.517471000000
H -1.624797000000 4.574008000000 -2.558090000000
H -0.103643000000 4.651572000000 -3.494946000000
H -0.415214000000 5.845810000000 -2.191742000000
O -1.720442000000 0.949347000000 0.198852000000

Mg -1.418439000000 2.984100000000 -0.091375000000
O -0.132422000000 3.884415000000 1.390056000000
C 0.509801000000 3.017726000000 2.026222000000
C 0.848288000000 1.791823000000 1.331321000000
C 0.732235000000 3.087298000000 3.510454000000
C 0.452596000000 0.657299000000 2.009352000000
C 0.065621000000 1.858359000000 4.215093000000
H 0.316949000000 4.020583000000 3.897939000000
H 1.811922000000 3.073271000000 3.705986000000
C 0.530387000000 0.548144000000 3.506763000000
H 1.574105000000 0.341741000000 3.779560000000
H -0.067503000000 -0.303742000000 3.841444000000
C -1.468404000000 1.975104000000 4.129263000000
H -1.812205000000 2.907338000000 4.587037000000
H -1.942347000000 1.145386000000 4.663769000000
H -1.833810000000 1.957582000000 3.097832000000
C 0.499474000000 1.824981000000 5.687929000000
H 0.171013000000 2.730007000000 6.208407000000
H 1.587845000000 1.753997000000 5.786412000000
H 0.055914000000 0.966582000000 6.203197000000
O -0.078855000000 -0.353671000000 1.339685000000
C 0.385079000000 0.764065000000 -0.805673000000
H 0.546755000000 0.548679000000 -1.855373000000
C -5.107707000000 4.068545000000 -0.761904000000
H -5.744956000000 3.408631000000 -0.164422000000
H -5.383169000000 3.987757000000 -1.813352000000
H -5.276052000000 5.086348000000 -0.401566000000
C -3.665890000000 3.686430000000 -0.544134000000
O -3.122613000000 3.880971000000 0.591118000000
O -2.979817000000 3.154508000000 -1.485508000000

Product

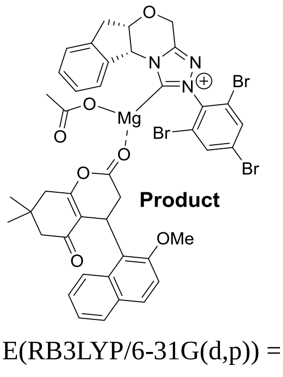

E(RB3LYP/6-31G(d,p)) = -10227.5949318

Temp. 298.150 K 1.00000 Atm.

E + zero-point Energies = -10226.902489
E + thermal Energies = -10226.850673
E + thermal Enthalpies = -10226.849729
E + thermal Free Energies = -10226.998484

Geometry at E(RB3LYP/6-31G(d,p))
Charge: 1 Multiplicity: 1
C 4.356953000000 -1.345292000000 -1.151864000000
C 2.860317000000 -0.524410000000 0.288695000000
N 3.425706000000 -2.254198000000 -1.123751000000
N 4.050465000000 -0.292383000000 -0.317129000000
C 4.928001000000 0.883254000000 -0.236664000000
H 4.712564000000 1.377617000000 0.715024000000
C 5.704061000000 -1.339657000000 -1.798578000000
H 5.711220000000 -0.705846000000 -2.696146000000
H 5.997697000000 -2.351457000000 -2.080906000000
C 6.442634000000 0.445528000000 -0.327997000000
H 6.891650000000 0.423405000000 0.666546000000
O 6.624647000000 -0.889340000000 -0.809353000000
C 7.114272000000 1.507229000000 -1.236971000000
H 7.669167000000 2.228968000000 -0.624828000000
H 7.843800000000 1.028022000000 -1.897421000000
C 4.742898000000 1.831866000000 -1.407168000000
C 5.975866000000 2.180400000000 -1.965416000000
C 3.550301000000 2.353994000000 -1.905934000000
C 6.028380000000 3.065842000000 -3.042818000000
C 3.605164000000 3.234825000000 -2.986903000000
H 2.595299000000 2.076779000000 -1.467809000000
C 4.837028000000 3.587384000000 -3.550387000000
H 6.980541000000 3.342736000000 -3.485931000000
H 2.688526000000 3.648919000000 -3.395376000000
H 4.866013000000 4.272142000000 -4.392336000000
N 2.502526000000 -1.716397000000 -0.239912000000
C 1.330712000000 -2.462067000000 0.068085000000
C 1.075104000000 -2.920827000000 1.368401000000
C 0.389891000000 -2.760473000000 -0.929347000000
C -0.036720000000 -3.700877000000 1.664266000000
C -0.724538000000 -3.550264000000 -0.660071000000
C -0.918618000000 -4.026503000000 0.635118000000
H -0.214753000000 -4.044034000000 2.674939000000
H -1.443052000000 -3.765493000000 -1.439694000000
Br 2.202149000000 -2.400706000000 2.820707000000
Br 0.572969000000 -2.017981000000 -2.666064000000
Br -2.434326000000 -5.097831000000 1.017919000000

C -4.408705000000 0.501311000000 -0.309287000000
C -5.601307000000 0.837095000000 0.334192000000
C -6.807701000000 0.173938000000 0.013682000000
C -6.812547000000 -0.810289000000 -0.945561000000
H -7.726992000000 0.435735000000 0.521459000000
H -7.741998000000 -1.316248000000 -1.190461000000
C -3.161801000000 1.178647000000 0.117351000000
C -0.959665000000 0.979442000000 1.145928000000
C -4.396686000000 -0.529920000000 -1.305266000000
C -5.631194000000 -1.187222000000 -1.629381000000
C -5.654026000000 -2.191960000000 -2.632847000000
C -4.511128000000 -2.543103000000 -3.311217000000
C -3.294781000000 -1.889052000000 -3.007302000000
H -2.389494000000 -2.152181000000 -3.545885000000
C -3.235923000000 -0.912152000000 -2.036652000000
H -4.539887000000 -3.307709000000 -4.080741000000
H -6.600926000000 -2.673901000000 -2.859634000000
H -2.285586000000 -0.427472000000 -1.841962000000
O -5.511754000000 1.790921000000 1.299156000000
C -6.709182000000 2.282933000000 1.901018000000
H -6.393576000000 3.072512000000 2.583301000000
H -7.220231000000 1.500508000000 2.472655000000
H -7.388999000000 2.699413000000 1.150480000000
O -0.000182000000 0.366102000000 1.661085000000
Mg 1.934473000000 0.468925000000 2.024313000000
O -4.897086000000 3.171135000000 -1.241208000000
C -3.862758000000 3.549048000000 -0.727273000000
C -2.934445000000 2.605233000000 -0.007407000000
C -3.408537000000 4.997406000000 -0.857416000000
C -1.771723000000 3.126904000000 0.497013000000
C -2.569698000000 5.525538000000 0.322729000000
H -4.305791000000 5.600836000000 -1.019781000000
H -2.817808000000 5.060806000000 -1.783881000000
C -1.369557000000 4.567541000000 0.496903000000
H -0.648345000000 4.717630000000 -0.320489000000
H -0.821783000000 4.772611000000 1.423745000000
C -3.417909000000 5.570139000000 1.609797000000
H -4.276069000000 6.235637000000 1.474581000000
H -2.829718000000 5.952510000000 2.450273000000
H -3.802882000000 4.584149000000 1.889520000000
C -2.048319000000 6.936555000000 0.006654000000
H -2.880884000000 7.634231000000 -0.125434000000
H -1.451385000000 6.948322000000 -0.911697000000
H -1.422943000000 7.316085000000 0.821694000000

O -0.821990000000 2.336888000000 1.058359000000
C -2.159269000000 0.408650000000 0.676810000000
H -2.275152000000 -0.660297000000 0.798628000000
C 3.827044000000 2.815536000000 4.452991000000
H 4.427458000000 3.551026000000 3.917808000000
H 4.445189000000 2.228186000000 5.134887000000
H 3.082400000000 3.341577000000 5.060024000000
C 3.107841000000 1.906835000000 3.493898000000
O 2.964260000000 2.232922000000 2.263455000000
O 2.611619000000 0.802888000000 3.905528000000

Supplementary Table 9. Data of enantiomerisation barrier determation[a]

Time (min)	% second eluted enantiomer (%t)	ln ((%t-50)/(%0-50))
0	95.50	0
20	95.01	-0.01083
40	93.72	-0.03991
60	92.53	-0.0675
80	91.12	-0.10122
120	88.33	-0.17148
240	81.56	-0.36582
360	76.23	-0.55081
600	68.58	-0.89563
840	62.94	-1.25739
1320	55.78	-2.06331

[a] Conditions: About 3 mg of enantio-enriched (**3gh**) were refluxed in 15 mL of toluene at 85 ℃. Samples of 7 μL of this solution were injected on Chiralpak IC (heptane / *i*PrOH = 80/20, 1 mL/min, UV detection at 254 nm) to monitor the percentage decrease of the second eluted enantiomer over time.

Supplementary Table 10. Statistical results.

k racemization =	2.59688E-05 s^{-1}
k enantiomerisation =	1.29844E-05 s^{-1}
$\Delta G^{\neq}$ enantiomerisation =	119.71 KJ.mol^{-1}
	28.6 kcal.mol^{-1}
half-life time[a] $t_{1/2}$ =	26692 s
	444.87 min
	7.41 h
	0.309 d

[a] T = 85 °C, $t_{1/2}$ = 7.41 h, T = 25 °C, $t_{1/2}$ = 1.67 year.

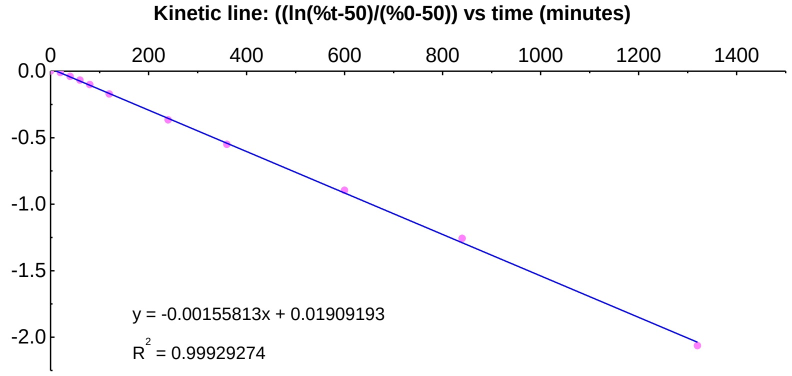

Supplementary Figure 175. Kinetic line based on the data in Supplementary Table 9.

Supplementary Methods

General Information

Chemicals and solvents were purchased from commercial suppliers and used as received. ^{1}H and ^{13}C NMR spectra were recorded on a Bruker ACF400 (400 MHz) spectrometer. Chemical shifts were reported in parts per million (ppm), and the residual solvent peak was used as an internal reference: proton (chloroform δ 7.26), carbon (chloroform δ 77.0) or tetramethylsilane (TMS δ 0.00) was used as a reference. Multiplicity was indicated as follows: s (singlet), d (doublet), t (triplet), q (quartet), m (multiplet), dd (doublet of doublet), bs (broad singlet). Coupling constants were reported in Hertz (Hz). All high resolution mass spectra were obtained on a Finnigan/MAT 95XL-T spectrometer. For thin layer chromatography (TLC), Merck pre-coated TLC plates (Merck 60 F254) were used, and compounds were visualized with a UV light at 254 nm. Flash chromatography separations were performed on Merck 60 (0.040-0.063 mm) mesh silica gel. The enantiomeric excesses of products were determined by chiral phase HPLC analysis. Optical rotations were recorded on Jasco DIP-1000 polarimeter.

General procedure for the synthesis of 1e-g.

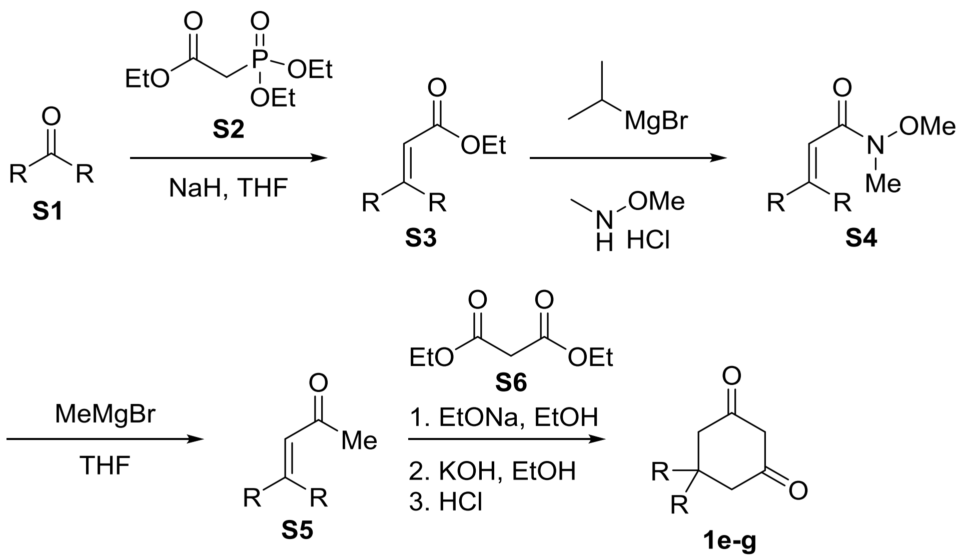

To a solution of **S2** (40.0 mmol) in THF (60 mL) was added NaH (1.6 g, 40 mmol, 60%) at 0 ^oC. The reaction mixture was stirred for 30 min. **S1** (20.0 mmol) in THF (20 mL) was added. The reaction mixture was warmed to 70 ^oC and stirred for 48 h. The reaction was then quenched by addition of NH_4Cl. The reaction mixture was extracted with Et_2O (3 x 80 mL) and washed with brine, dried over sodium sulfate. The solvent was evaporated, and the residue was purified by column chromatography to afford the **S3**.

To the above product **S3** (20.0 mmol), N-methoxymethanamine (40.0 mmol) in THF (60 mL) was added ^iPrMgBr (40.0 mL, 2.0 M in THF) at -20 ^oC under N_2, the mixture was warmed to RT and stirred for 2 h. The reaction was then quenched by addition of NH_4Cl. The reaction mixture was extracted with EtOAc (3 x 80 mL) and washed with brine, dried over sodium sulfate. The solvent was removed under reduced pressure, and the residue was used directly for the next step.

To the above product **S4** (20.0 mmol) in THF (40 mL) was added MeMgBr (20.0 mL, 2.0 M in THF) at -20 ^oC under N_2, the mixture was warmed to 0 ^oC and stirred for 2 h. The reaction was then quenched by addition of NH_4Cl, the reaction mixture was extracted with EtOAc (3 x 80 mL) and washed with brine, dried over sodium sulfate. The solvent was evaporated, and the residue was purified by column chromatography to afford the **S5**.

Sodium metal (460 mg, 20.0 mmol) was added to ethanol (30 mL) at rt under argon. After the sodium dissolved completely, diethyl malonate **S6** (3.20 g, 20.0 mmol) was added to the sodium ethoxide solution followed by addition of **S5** (10.0 mmol) at rt. The solution was warmed to reflux. After 24 h reflux, the solution was cooled to rt and a solution of potassium hydroxide (5.6 g, 100 mmol) in H_2O (20 mL) was added. The resulting solution was warmed to reflux for 48 h. The solution was cooled to 0 ℃ and acidified with 6 N HCl to pH 3. The EtOH was evaporated, and the residue was extracted with EtOAc (3 x 100 mL) and washed with brine, dried over sodium sulfate. The solvent was evaporated, and the residue was purified by column chromatography to afford the **1e-g**.

1b-d were prepared according to references [1] and [2].

General procedure for the synthesis of 2.

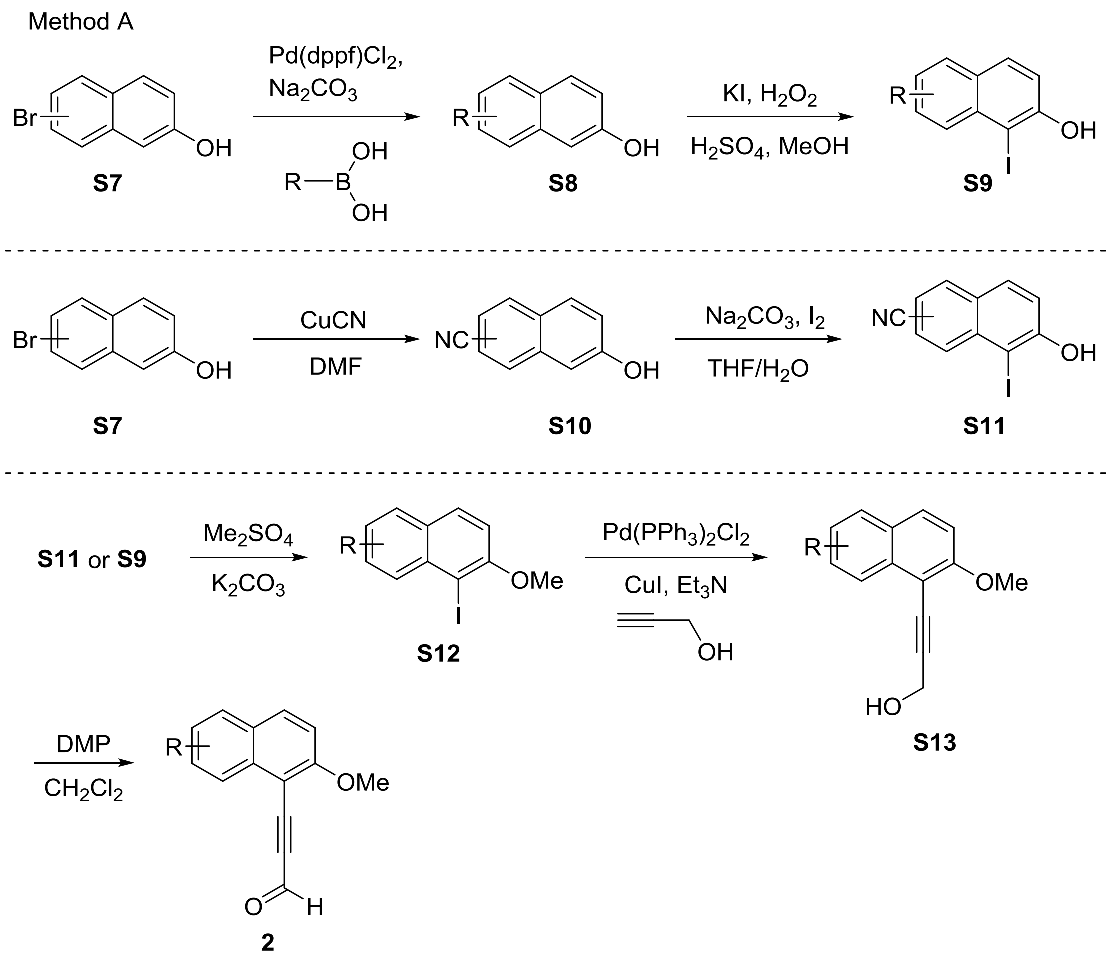

2a, 2b, 2c, 2e, 2f, 2g, 2h, 2i, 2j, 2k, 2l, 2m, 2n, 2o, 2p, 2q, 2r, 2t, and **2u** were synthesized by method A.

Method A:

To a solution of **S7** (10.0 mmol) in 1,4-dioxane (40 mL) was added 2 M aqueous Na_2CO_3 (10 mL, 20 mmol), boric acid (15.0 mmol) and $PdCl_2$(dppf) (183 mg, 0.25 mmol). The flask was flushed with nitrogen gas. The solution was warmed to reflux for 4 h. The reaction mixture was cooled to RT. The 1,4-dioxane was evaporated, and the residue was extracted with EtOAc (3 x 30 mL) and washed with brine, dried over sodium sulfate. The solvent was evaporated, and the residue was purified by column

chromatography to afford **S8**.

To MeOH (50 mL) being stirred at 0 ℃ in a round-bottom flask was added sulfuric acid (95.8%, 0.56 mL, 15.0 mmol), **S7** (10.0 mmol), KI (1.67 g, 10.0 mmol), and H_2O_2 (31.6%, 2.0 mL, 21.0 mmol). The dark mixture was kept stirring at 0 ℃ for 9 h at which time the substrate appeared consumed (TLC). The mixture was quenched by adding excess saturated aqueous Na_2SO_3. The MeOH was evaporated, and the residue was extracted with EtOAc (3 x 30 mL) and washed with brine, dried over sodium sulfate. The solvent was evaporated to yield the product **S9** which was used directly for the next step.

A mixture of bromo-2-naphthol **S7** (10.0 mmol) and CuCN (1.34 g, 15.0 mmol) in DMF (20 mL) was vigorously stirred at 150 ℃ for 4.5 h. After cooling to room temperature, the mixture was added 10% NaOH (30 mL) and stirred for 5 min. The reaction was filtered and the filtrate was washed with water (15 mL). Then, 3 N HCl was used to adjust pH to 2-3. The precipitate was collected by filtration to give **S10** as a brown solid which was used directly for the next step.

To a solution of **S10** (5.0 mmol) in THF: H_2O (50 mL, 1:4) was added Na_2CO_3 (1.06 g, 10.0 mmol), and I_2 (1.27 g, 5.0 mmol) at 0 ℃. The reaction was vigorously stirred at RT for 4 h. at which time TLC indicated complete conumption of the naphthol. The mixture was quenched by adding excess saturated aqueous Na_2SO_3. The mixture was extracted with EtOAc (3 x 30 mL) and washed with brine, dried over sodium sulfate. The solvent was evaporated to yield the product **S11** which was used directly for the next step.

To a solution of **S11** or **S9** (5.0 mmol) in acetone (20 mL) was added K_2CO_3 (1.38 g, 10.0 mmol), dimethyl sulfate (1.0 mL, 10.0 mmol). The solution was warmed to reflux for 4 h at which time TLC indicated complete consumption of the naphthol. The reaction mixture was cooled to RT. Et_3N (5.0 mL) was added, and the reaction was stirred for 1 h. The reaction mixture was filtered through a pad of Celite, the solid was washed with DCM and the solvent was evaporated to yield a yellow solid. The solid was washed with PE for three times to give the product **S12** which was used directly for the next step.

To a solution of **S12** (5.00 mmol) and propargyl alcohol (1.4 g, 25.0 mmol) in triethylamine (20 mL) was added $PdCl_2(PPh_3)_2$ (142 mg, 0.202 mmol) and CuI (76 mg, 0.40 mmol). The flask was flushed with nitrogen gas. The resulting mixture was stirred at room temperature for 40 h. The reaction mixture was filtered and washed with EtOAc. The combined filtrate was concentrated and the residue was purified by a silica gel column chromatography to give **S13**.

To a solution of **S13** (4.0 mmol) in DCM (50 mL) was added $NaHCO_3$ (1.06 g, 10.0 mmol), and Dess-Martin oxidant (2.12 g, 5.0 mmol) at 0 ℃. The reaction was stirred at RT for 2 h. at which time TLC indicated complete conumption of the **S14**. The mixture was quenched by adding excess saturated aqueous Na_2SO_3. The mixture was vigorously stirred at RT for 1 h. The mixture was extracted with EtOAc (3 x 30 mL) and washed with brine, dried over sodium sulfate. The solvent was concentrated and the residue was purified by a silica gel column chromatography to give **2**.

Method B

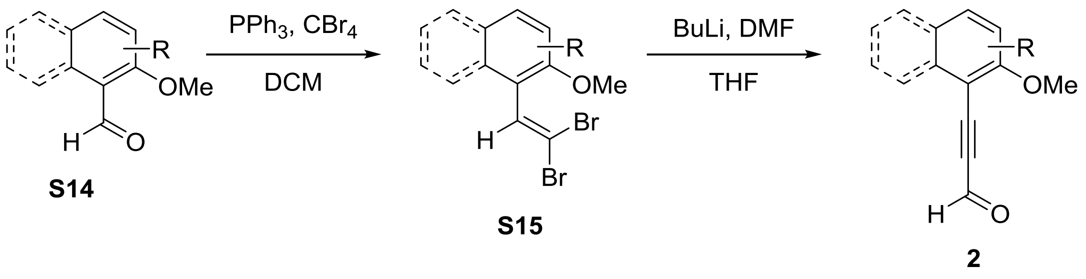

2d and **2s** were synthesized by method B.

Method B:

To a solution of **S14** (10.0 mmol) in DCM (50 mL) was added PPh_3 (13.1 g, 50.0 mmol), CBr_4 (8.3 g, 25.0 mmol) was added slowly at 0 ^{o}C. The solution was stirred for 30 mins. Half of the solvent was evaporated, PE: EtOAc (50 mL, 1:1) was added to the mixture. The mixture was filtered through a pad of silica gel, the solid was washed with PE: EtOAc (50 mL, 1:1) for three times and the solvent was evaporated and the residue was purified by a silica gel column chromatography to give **S15**.

To the above product **S15** (8.0 mmol), in THF (30 mL) was added BuLi (7.0 mL, 2.5 M) at -78 ^{o}C under N_2, the mixture was warmed to -30 ^{o}C and stirred for 1 h. Then DMF (1.19 g, 16.0 mmol) was added to the reaction mixture. The reaction was warmed to RT and stirred for another 2 hours and then quenched by addition of NH_4Cl. The reaction mixture was extracted with EtOAc (3 x 30 mL) and washed with brine, dried over sodium sulfate. The solvent was concentrated and the residue was purified by a silica gel column chromatography to give **2**.

Characterization data of 1 and 2.

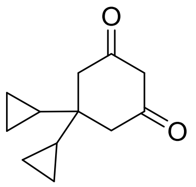

1e: White solid: **^{1}H NMR** (400MHz, $CDCl_3$) δ 3.35 (s, 2H), 2.41 (s, 4H), 0.67 – 0.60 (m, 2H), 0.42 – 0.32 (m, 8H); **^{13}C NMR** (101MHz, $CDCl_3$) δ 204.3, 57.4, 48.9, 40.8, 35.0, 18.8, 0.4; **HRMS** (ESI): *m/z*: calculated for $C_{12}H_{17}O_2^+$: $[M + H]^+$ 193.1223, found: 193.1229.

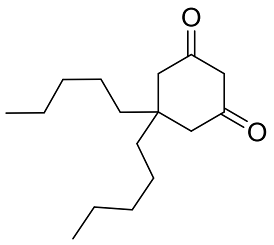

1f: White solid: **^{1}H NMR** (400MHz, $CDCl_3$) δ 3.33 (s, 2H), 2.53 (s, 4H), 1.38 -1.22 (m, 16H), 0.88 (t, *J* = 6.9 Hz, 6H); **^{13}C NMR** (101MHz, $CDCl_3$) δ 204.2, 57.6, 50.9, 43.1, 37.5, 32.1, 22.7, 22.4, 13.9; **HRMS** (ESI): *m/z*: calculated for $C_{16}H_{29}O_2^+$: $[M + H]^+$ 253.2162, found: 253.2170.

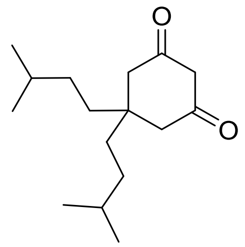

1g: White solid: **^{1}H NMR** (400MHz, $CDCl_3$) δ 3.33 (s, 2H), 2.52 (s, 4H), 1.21 (s, 4H), 1.09 (s, 6H), 0.86 (s, 12H); **^{13}C NMR** (101MHz, $CDCl_3$) δ 204.0, 57.6, 50.9, 35.9, 35.0, 31.9, 28.3, 22.5; **HRMS** (ESI): *m/z*: calculated for $C_{16}H_{29}O_2^+$: $[M + H]^+$ 253.2162, found: 253.2171.

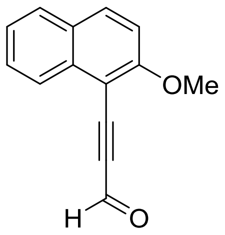

2a: Yellow solid; **^{1}H NMR** (400 MHz, $CDCl_3$) δ 9.59 (s, 1H), 8.22 (d, *J* = 8.5 Hz, 1H), 7.96 (d, *J* = 9.2 Hz, 1H), 7.79 (d, *J* = 8.1 Hz, 1H), 7.62 – 7.57 (m, 1H), 7.44 – 7.40 (m, 1H), 7.24 (d, *J* = 9.6 Hz, 1H), 4.05 (s, 3H); **^{13}C NMR** (100 MHz, $CDCl_3$) δ 176.7, 162.2, 134.9, 134.1, 128.6, 128.5, 128.2, 124.8, 124.7, 112.1, 102.1, 98.5, 91.3, 56.5; **HRMS** (ESI): *m/z*: calculated for $C_{14}H_{10}NaO_2^+$: $[M + Na]^+$ 233.0573, found: 233.0579.

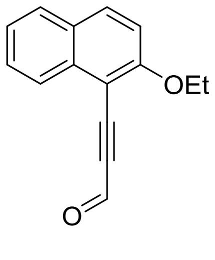

2b: Yellow solid; **^{1}H NMR** (400 MHz, $CDCl_3$) δ 9.59 (s, 1H), 8.21 (d, *J* = 8.5 Hz, 1H), 7.91 (d, *J* = 9.0 Hz, 1H), 7.77 (d, *J* = 8.1 Hz, 1H), 7.58 (t, *J* = 7.4 Hz, 1H), 7.40 (t, *J* = 7.4 Hz, 1H), 7.23 (d, *J* = 9.1 Hz, 1H), 4.28 (q, *J* = 7.0 Hz, 1H), 1.51 (t, *J* = 7.0 Hz, 1H); **^{13}C NMR** (100 MHz, $CDCl_3$) δ 176.7, 161.7, 134.9, 133.8, 128.4, 128.3,

128.1, 124.7, 113.3, 102.5, 98.4, 91.5, 65.2, 14.9; **HRMS** (ESI): *m/z*: calculated for $C_{15}H_{12}NaO_2^+$: [M + Na]$^+$ 247.0730, found: 247.0736.

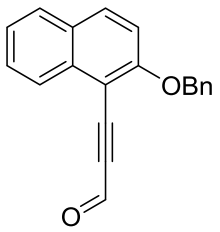

2c: Yellow solid; **^{1}H NMR** (400 MHz, CDCl$_3$) δ **^{1}H NMR** (400MHz, CDCl$_3$) δ 9.56 (s, 1H), 8.22 (d, *J* = 8.4 Hz, 1H), 7.86 (d, *J* = 9.1 Hz, 1H), 7.75 (d, *J* = 8.2 Hz, 1H), 7.59 – 7.55 (m, 1H), 7.48 (d, *J* = 7.3 Hz, 2H), 7.42 – 7.36 (m, 3H), 7.33 – 7.29 (m, 1H), 7.23 – 7.20 (m, 1H), 5.33 (s, 2H); **^{13}C NMR** (101MHz, CDCl$_3$) δ 176.7, 161.4, 136.3, 134.9, 133.9, 128.7, 128.5, 128.5, 128.4, 127.0, 125.0, 124.8, 114.0, 103.3, 98.5, 91.2, 71.2; **HRMS** (ESI): *m/z*: calculated for $C_{20}H_{14}NaO_2^+$: [M + Na]$^+$ 309.0886, found: 309.0892.

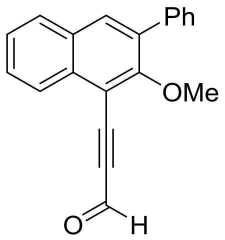

2d: White solid; **^{1}H NMR** (400MHz, CDCl$_3$) δ 9.64 (s, 1H), 8.30 (d, *J* = 8.4 Hz, 1H), 7.97 (s, 1H), 7.88 (d *J* = 8.1 Hz, 1H), 7.68 – 7.63 (m, 3H), 7.56 – 7.43 (m, 4H), 3.78 (s, 3H); **^{13}C NMR** (101MHz, CDCl$_3$) δ 176.4, 161.4, 137.1, 134.7, 134.3, 134.0, 130.1, 129.1, 128.4, 128.4, 128.1, 127.8, 126.2, 124.9, 108.8, 97.4, 90.4, 61.9; **HRMS** (ESI): *m/z*: calculated for $C_{20}H_{14}NaO_2^+$: [M + Na]$^+$ 309.0886, found: 309.0880.

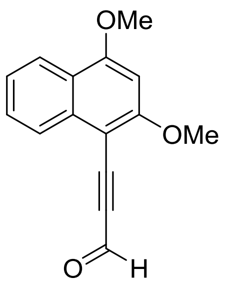

2e: Yellow solid; **^{1}H NMR** (400MHz, $CDCl_3$) δ 9.55 (s, 1H), 8.16 (s, 2H), 7.60 (s, 1H), 7.39 (d, *J* = 6.8 Hz, 1H), 6.53 (s, 1H), 4.05 (s, 6H); **^{13}C NMR** (101MHz, $CDCl_3$) δ 176.5, 164.2, 160.8, 135.4, 129.2, 124.5, 124.1, 122.6, 120.9, 98.9, 94.3, 93.3, 91.9, 56.5, 55.9; **HRMS** (ESI): *m/z*: calculated for $C_{15}H_{12}NaO_3^+$: $[M + Na]^+$ 263.0679, found: 263.0672.

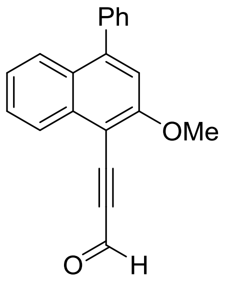

2f: White solid; **^{1}H NMR** (400MHz, $CDCl_3$) δ 9.60 (s, 1H), 8.31 (d, *J* = 8.4 Hz, 1H), 7.80 (d, *J* = 8.4 Hz, 1H), 7.62 – 7.58 (m, 1H), 7.54 – 7.47 (m, 5H), 7.37 – 7.33 (m, 1H), 7.19 (s, 1H), 4.04 (s, 3H); **^{13}C NMR** (101MHz, $CDCl_3$) δ 176.6, 161.4, 146.8, 139.6, 135.4, 129.6, 128.5, 128.4, 128.2, 126.9, 125.1, 124.8, 113.3, 101.5, 98.8, 91.4, 56.5; **HRMS** (ESI): *m/z*: calculated for $C_{20}H_{14}NaO_2^+$: $[M + Na]^+$ 309.0886, found: 309.0882.

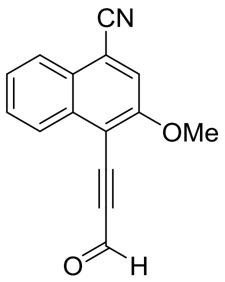

2g: Yellow solid; **^{1}H NMR** (400MHz, DMSO-d^6) δ 9.60 (s, 1H), 8.18 (s, 2H), 8.04 (s,

1H), 7.78 (s, 1H), 7.69 (d, *J* = 5.6 Hz, 1H), 4.06 (s, 3H); **^{13}C NMR** (101MHz, DMSO-D$_6$) δ 179.0, 160.5, 134.0, 130.5, 127.9, 126.9, 125.5, 125.3, 120.3, 117.1, 113.8, 106.8, 99.8, 87.2, 57.8; **HRMS** (ESI): *m/z*: calculated for $C_{15}H_9NNaO_2^+$: [M + Na]$^+$ 258.0525, found: 258.0530.

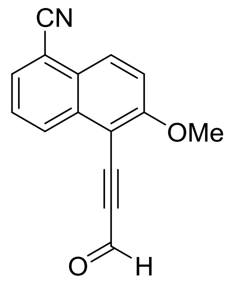

2h: Yellow solid**;** **^{1}H NMR** (400MHz, DMSO-D$_6$) δ 9.59 (s, 1H), 8.42 (s, 1H), 8.28 (s, 1H), 8.11 (s, 1H), 7.81 (s, 2H), 4.08 (s, 3H); **^{13}C NMR** (101MHz, DMSO-D$_6$) δ 178.9, 163.1, 134.3, 132.3, 130.8, 129.9, 128.7, 126.7, 117.7, 116.6, 110.2, 102.3, 99.0, 88.4, 57.4; **HRMS** (ESI): *m/z*: calculated for $C_{15}H_9NNaO_2^+$: [M + Na]$^+$ 258.0525, found: 258.0518.

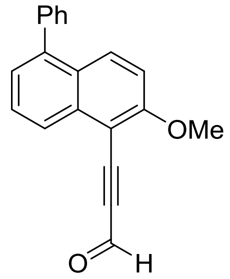

2i: Yellow solid**;** **^{1}H NMR** (400MHz, CDCl$_3$) δ 9.61 (s, 1H), 8.27 (d, *J* = 8.5 Hz, 1H), 8.03 (d, *J* = 9.4 Hz, 1H), 7.65 – 7.61 (m, 1H), 7.51 – 7.43 (m, 5H), 7.35 (d, *J* = 7.1 Hz, 1H), 7.15 (d, *J* = 2.5 Hz, 1H), 4.03 (s, 3H); **^{13}C NMR** (101MHz, CDCl$_3$) δ 176.7, 162.0, 141.1, 140.1, 135.4, 132.5, 130.0, 128.4, 128.2, 127.6, 126.6, 125.9, 124.3, 102.3, 98.6, 91.4, 56.5; **HRMS** (ESI): *m/z*: calculated for $C_{20}H_{14}NaO_2^+$: [M + Na]$^+$ 309.0886, found: 309.0880.

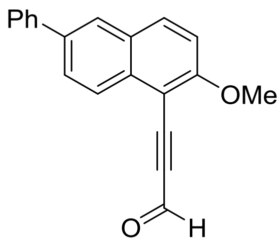

2j: Yellow solid**;** **^{1}H NMR** (400MHz, $CDCl_3$) δ 9.63 (s, 1H), 8.30 – 8.27 (m, 1H), 8.01 – 7.98 (m, 2H), 7.88 – 7.85 (m, 1H), 7.71 (d, *J* = 8.2 Hz, 2H), 7.51 (t, *J* = 8.0 Hz, 2H), 7.28 – 7.25 (m, 1H), 4.07 (s, 3H); **^{13}C NMR** (101MHz, $CDCl_3$) δ 176.6, 162.1, 140.3, 137.5, 134.2, 134.0, 128.9, 128.4, 128.1, 127.5, 127.1, 126.1, 125.2, 112.4, 102.0, 98.4, 91.0, 56.5; **HRMS** (ESI): *m/z*: calculated for $C_{20}H_{14}NaO_2^+$: $[M + Na]^+$ 309.0886, found: 309.0892.

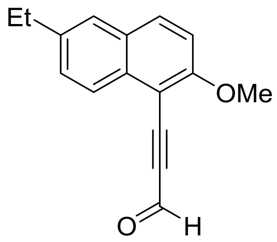

2k: Yellow solid**;** **^{1}H NMR** (400MHz, $CDCl_3$) δ 9.61 (s, 1H), 8.14 (d, *J* = 8.6 Hz, 1H), 7.90 – 7.87 (m, 1H), 7.57 (s, 1H), 7.46 (dd, *J* = 8.6, 1.8 Hz, 1H), 7.22 – 7.19 (m, 1H), 4.04 (dd, *J* = 4.9, 3.6 Hz, 3H), 2.80 (dd, *J* = 15.2, 7.6 Hz, 2H), 1.35 – 1.31 (m, 3H); **^{13}C NMR** (101MHz, $CDCl_3$) δ 176.6, 161.6, 140.6, 133.5, 133.2, 129.7, 128.4, 126.0, 124.5, 111.9, 101.8, 98.3, 91.5, 56.4, 28.5, 15.3; **HRMS** (ESI): *m/z*: calculated for $C_{16}H_{14}NaO_2^+$: $[M + Na]^+$ 261.0886, found: 261.0881.

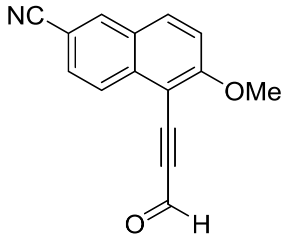

2l: White solid**;** **^{1}H NMR** (400MHz, DMSO-D_6) δ 9.59 (s, 1H), 8.63 (d, *J* = 1.4 Hz, 1H), 8.33 (d, *J* = 9.3 Hz, 1H), 8.23 (d, *J* = 8.7 Hz, 1H), 7.95 (dd, *J* = 8.7, 1.7 Hz, 1H),

7.74 (d, *J* = 9.3 Hz, 1H), 4.10 (s, 3H); **^{13}C NMR** (101MHz, DMSO-D$_6$) 178.9, 164.5, 136.3, 135.6, 135.5, 129.6, 127.3, 125.6, 119.4, 115.5, 107.5, 101.4, 98.7, 88.7, 57.5; **HRMS** (ESI): *m/z*: calculated for $C_{15}H_9NNaO_2^+$: [M + Na]$^+$ 258.0525, found: 258.0531.

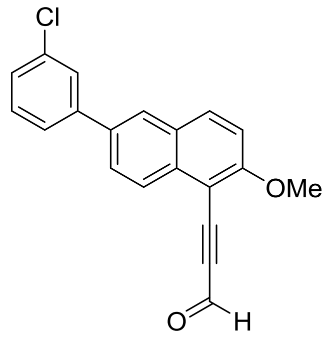

2m: Yellow solid**; ^{1}H NMR** (400MHz, CDCl$_3$) δ 9.60 (s, 1H), 8.23 (d, *J* = 8.7 Hz, 1H), 7.97 (d, *J* = 9.2 Hz, 1H), 7.91 (d, *J* = 1.6 Hz, 1H), 7.76 (dd, *J* = 8.7, 1.8 Hz, 1H), 7.64 (t, *J* = 1.8 Hz, 1H), 7.54 – 7.51 (m, 1H), 7.40 – 7.32 (m, 2H), 7.26 – 7.24 (m, 1H), 4.05 (s, 3H); **^{13}C NMR** (101MHz, CDCl$_3$) δ 176.6, 162.3, 142.1, 136.0, 134.8, 134.3, 134.2, 130.1, 128.3, 127.7, 127.4, 127.2, 126.3, 125.4, 112.6, 102.1, 98.4, 90.8, 56.5; **HRMS** (ESI): *m/z*: calculated for $C_{20}H_{13}ClNaO_2^+$: [M + Na]$^+$ 343.0496, found: 343.0490.

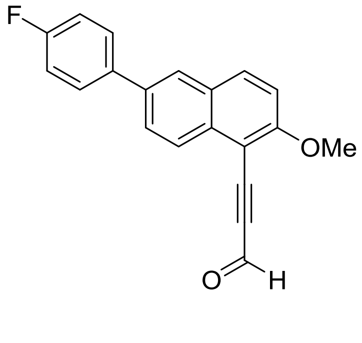

2n: Yellow solid**; ^{1}H NMR** (400MHz, DMSO-D$_6$) δ 9.60 (s, 1H), 8.26 – 8.16 (m, 3H), 7.98 (d, *J* = 7.6 Hz, 1H), 7.84 (d, *J* = 7.2 Hz, 2H), 7.58 (d, *J* = 7.6 Hz, 1H), 7.35 (d, *J* = 7.4 Hz, 2H), 4.06 (s, 3H); **^{13}C NMR** (101MHz, DMSO-D$_6$) δ 178.9, 162.7, 136.3, 135.7, 135.3, 133.7, 129.9 (d, *J* = 8.2 Hz), 128.6, 128.2, 126.6, 128.2, 126.6, 125.0, 116.3 (d, *J* = 2.1 Hz), 114.2, 100.8, 98.7, 90.3, 57.1; **^{19}F NMR** (376 MHz, DMSO-D$_6$)

δ -115.2; **HRMS** (ESI): *m/z*: calculated for $C_{20}H_{13}FNaO_2^+$: [M + Na]$^+$ 327.0792, found: 327.0799.

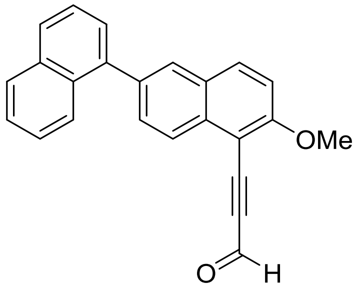

2o: Yellow solid**;** ^{1}**H NMR** (400MHz, $CDCl_3$) δ 9.60 (s, 1H), 8.32 (d, *J* = 7.9 Hz, 1H), 7.98 – 7.88 (m, 5H), 7.74 (d, *J* = 7.7 Hz, 1H), 7.53 – 7.41 (m, 4H), 7.26 (d, *J* = 8.6 Hz, 1H), 4.06 (s, 3H); ^{13}**C NMR** (101MHz, $CDCl_3$) δ 176.7, 162.3, 139.4, 137.4, 134.2, 134.1, 133.9, 131.7, 131.2, 129.2, 128.4, 128.3, 128.0, 127.3, 126.3, 126.0, 125.8, 125.5, 124.6, 112.5, 102.2, 98.5, 91.1, 56.6; **HRMS** (ESI): *m/z*: calculated for $C_{24}H_{16}NaO_2^+$: [M + Na]$^+$ 359.1043, found: 359.1049.

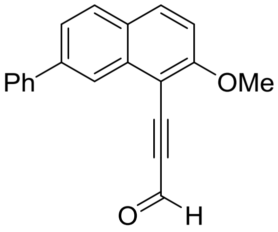

2p: White solid; ^{1}**H NMR** (400MHz, $CDCl_3$) δ 9.59 (s, 1H), 8.36 (s, 1H), 7.92 (d, *J* = 9.1 Hz, 1H), 7.81 (d, *J* = 8.4 Hz, 1H), 7.30 (d, *J* = 8.4 Hz, 2H), 7.64 (dd, *J* = 8.4, 1.6 Hz, 1H), 2.49 (t, *J* = 7.3 Hz, 2H), 7.39 (t, *J* = 7.3 Hz, 1H), 7.19 (d, *J* = 9.1 Hz, 1H), 4.02 (s, 3H); ^{13}**C NMR** (101MHz, $CDCl_3$) δ 176.6, 162.6, 141.3, 140.7, 135.2, 133.8, 129.0, 127.9, 127.6, 127.4, 124.6, 122.5, 112.0, 102.3, 98.8, 91.2, 56.6; **HRMS** (ESI): *m/z*: calculated for $C_{20}H_{14}NaO_2^+$: [M + Na]$^+$ 309.0886, found: 309.0881.

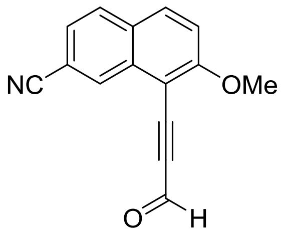

2q: White solid**; ^{1}H NMR** (400MHz, DMSO-D$_6$) δ 9.60 (s, 1H), 8.49 (s, 1H), 8.26 (d, *J* = 6.1 Hz, 1H), 8.12 (d, *J* = 4.2 Hz, 1H), 7.72 (d, *J* = 6.5 Hz, 2H), 4.06 (s, 3H); **^{13}C NMR** (101MHz, DMSO-D$_6$) δ 179.1, 163.3, 134.8, 133.6, 130.8, 130.0, 129.6, 125.8, 119.4, 116.8, 111.5, 101.5, 99.0, 88.4; **HRMS** (ESI): *m/z*: calculated for $C_{15}H_9NNaO_2^+$: [M + Na]$^+$ 258.0525, found: 258.0520.

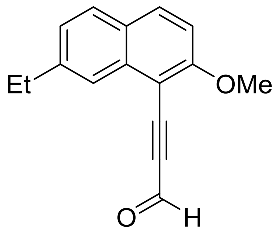

2r: Yellow solid; **^{1}H NMR** (400MHz, CDCl$_3$) δ 9.62 (s, 1H), 7.95 (s, 1H), 7.82 (d, *J* = 9.1 Hz, 1H), 7.65 (d, *J* = 8.4 Hz, 1H), 7.23 (dd, *J* = 8.3, 1.5 Hz, 1H), 7.06 (d, *J* = 9.1 Hz, 1H), 3.95 (s, 3H), 2.81 (dd, *J* = 15.2, 7.6 Hz, 2H), 1.32 (t, *J* = 7.6 Hz, 3H); **^{13}C NMR** (101MHz, CDCl$_3$) δ 176.5, 162.1, 144.8, 134.8, 133.6, 128.2, 126.4, 125.6, 122.1, 110.8, 101.1, 98.4, 91.6, 29.1, 15.3; **HRMS** (ESI): *m/z*: calculated for $C_{16}H_{14}NaO_2^+$: [M + Na]$^+$ 261.0886, found: 261.0891.

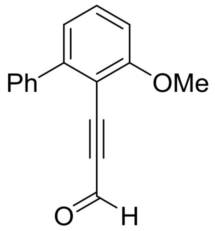

2s: White solid; **^{1}H NMR** (400MHz, CDCl$_3$) δ 9.32 (s, 1H), 7.58 – 7.41 (m, 6H), 7.05 (dd, *J* = 7.7, 0.5 Hz, 1H), 6.95 (d, *J* = 8.4 Hz, 1H), 3.98 (s, 3H), **^{13}C NMR** (101MHz, CDCl$_3$) δ 176.6, 162.4, 148.0, 139.2, 132.3, 129.1, 128.1, 128.1, 122.0, 109.3, 107.4,

95.5, 92.3, 56.1; **HRMS** (ESI): *m/z*: calculated for $C_{16}H_{12}NaO_2^+$: [M + Na]$^+$ 259.0730, found: 259.0737.

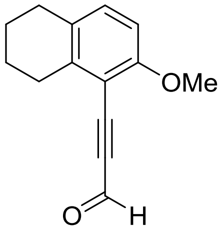

2t: Yellow solid; **^{1}H NMR** (400MHz, $CDCl_3$) δ 9.49 (s, 1H), 7.14 (d, *J* = 8.6 Hz, 1H), 6.69 (d, *J* = 8.6 Hz, 1H), 3.88 (s, 3H), 2.88 (t, *J* = 6.0 Hz, 2H), 2.69 (t, *J* = 6.2 Hz, 2H), 1.82 – 1.74 (m, 4H); **^{13}C NMR** (101MHz, $CDCl_3$) δ 176.8, 160.4, 143.1, 133.4, 129.9, 108.1, 107.9, 97.7, 91.5, 55.8, 28.8, 28.2, 22.7, 22.5; **HRMS** (ESI): *m/z*: calculated for $C_{14}H_{14}NaO_2^+$: [M + Na]$^+$ 237.0886, found: 237.0880.

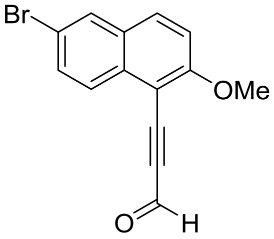

2u: White solid; **^{1}H NMR** (400MHz, DMSO-D_6) δ 9.57 (s, 1H), 8.23 (d, *J* = 2.0 Hz, 1H), 8.14 (d, *J* = 9.2 Hz, 1H), 8.00 (d, *J* = 9.0 Hz, 1H), 7.75 (dd, *J* = 9.0, 2.1 Hz, 1H), 7.58 (d, *J* = 9.3 Hz, 1H), 4.04 (s, 3H); **^{13}C NMR** (101MHz, DMSO-D_6) δ 178.8, 162.7, 134.0, 133.1, 132.0, 131.0, 129.4, 126.4, 118.0, 114.8, 101.1, 98.7, 89.5, 57.2; **HRMS** (ESI): *m/z*: calculated for $C_{14}H_9BrNaO_2^+$: [M + Na]$^+$ 310.9678, 312.9658, found: 310.9685, 312.9663.

Supplementary Table 11. Optimization of the reaction conditions.

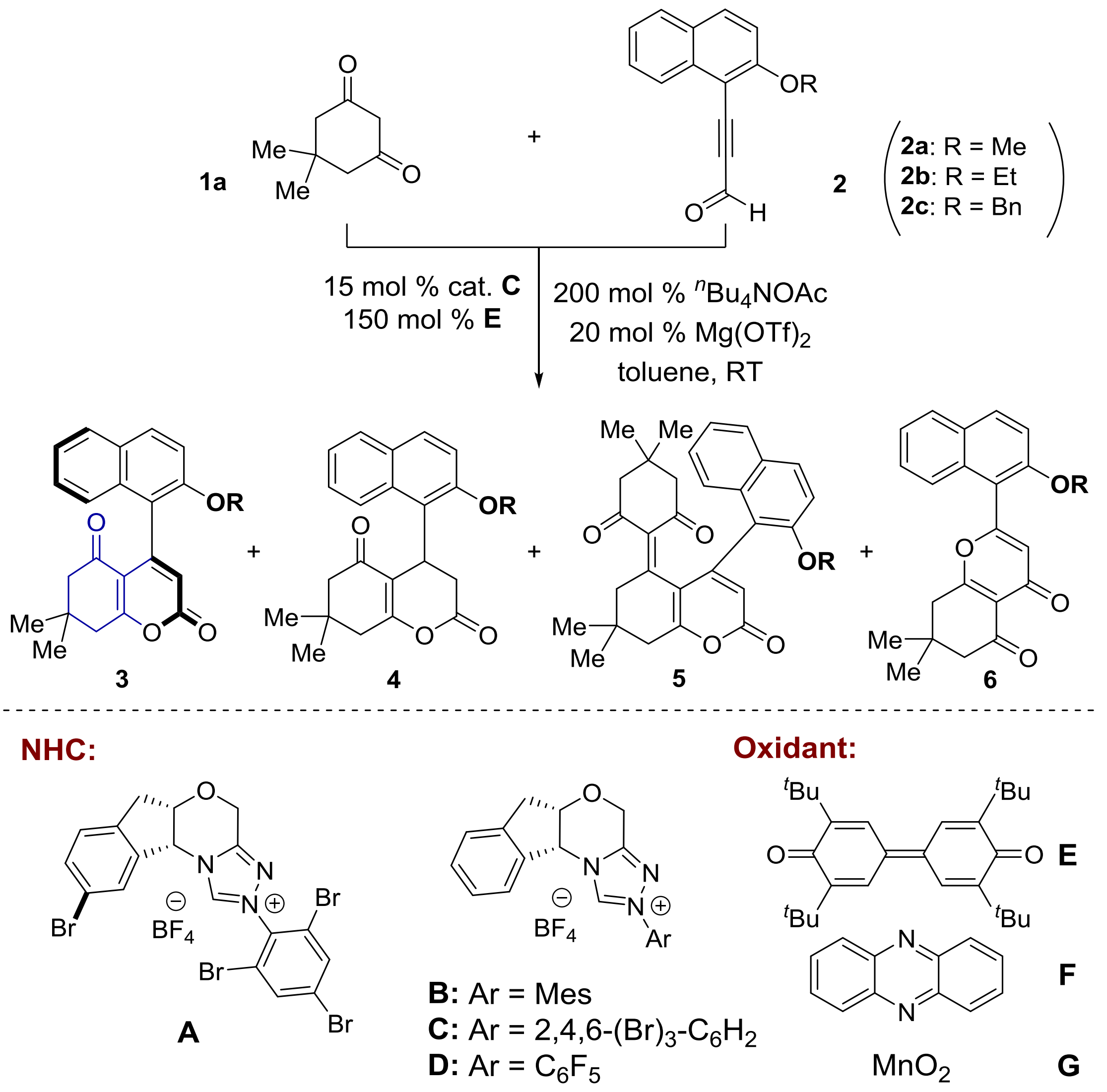

entry	deviation from standard conditions[a]	yield **3** (%)[b]	er **3** (%)[c]	yield **4** (%)[b]	yield **5** (%)[b]	yield **6** (%)[b]
1[d]	**none**	**70**	**91:9**	<5	<5	<5
2	no cat. **C**	0	-	0	0	0
3	**A** instead of **C**	54	81:19	<5	20	18
4	**B** instead of **C**	<5	-	<5	<5	<5
5	**D** instead of **A**	<5	-	<5	<5	60
6	no $Mg(OTf)_2$	60	90:10	<5	<5	18
7	LiCl instead of $Mg(OTf)_2$	58	90:10	<5	<5	<10
8	$In(OTf)_3$ instead of $Mg(OTf)_2$	63	90:10	<5	<5	<10
9	$Sc(OTf)_3$ instead of $Mg(OTf)_2$	60	90:10	<5	<5	<10
10	$Zn(OTf)_2$ instead of $Mg(OTf)_2$	61	90:10	<5	<5	<10
11	$CHCl_3$ as solvent	40	80:20	<5	15	23

12	THF as solvent	54	85:15	<5	<10	19
13	Dioxane as solvent	20	-	<5	<5	50
14	no ^{n}Bu$_4$NOAc	0	-	0	0	0
15	DIPEA as base	45	80:20	<5	17	20
16	Cs_2CO_3 as base	40	75:25	<5	20	22
17	KO^{t}Bu as base	<10	-	<5	<5	<5
18	**F** instead of **E**	<5	-	74	<5	<5
19	**G** instead of **E**	<5	-	70	<5	<5
20	10 mol % cat. **C**	69[e]	91:9	<5	<5	13
21	100 mol % **E**	28	91:9	20	20	10
22	120 mol % **E**	56	91:9	<5	22	10
23	100 mol % base	40	91:9	<5	23	25
24	50 mol % base	30	91:9	<5	32	28
25	150 mol % **1a**	31	91:9	<5	30	28
26	200 mol % **1a**	28	91:9	<5	24	40
27	150 mol % **2a**	<5	-	<5	<5	<5
28	200 mol % **2a**	<5	-	<5	<5	<5
29[e]	**2b** instead of **2a**[f]	60	75:25	<5	<5	18
30[f]	**2c** instead of **2a**[g]	60	72:8	<5	<5	<5

[a]Standard conditions: **1a** (0.11 mmol), **2a** (0.10 mmol, R = Me), ^{n}Bu$_4$NOAc (0.2 mmol), oxidant **E** (0.15 mmol), $Mg(OTf)_2$ (20 mol %), cat. **C** (15 mol %), toluene (2.0 mL), room temperature, N_2, 24 h. [b]Isolated yield. [c]Determined by chiral HPLC. [e] 48 h. [f] **3ab** as major product. [g] **3ac** as major product.

General procedure for NHC-catalyzed reaction of 1 with 2.

In a glovebox, a flame-dried Schlenk reaction tube equipped with a magnetic stir bar, was added the azolium precatalyst (9.2 mg, 0.015 mmol), Bu$_4$NOAc (60.2 mg, 0.20 mmol), DQ (62.0 mg), **1** (0.11 mmol) and **2** (0.10 mmol), and freshly distilled toluene (2.0 mL) was added. The mixture was then stirred at rt for 24 h. The reaction mixture was filtered through a pad of Celite, the solid was washed with DCM and the solvent was evaporated and purified by via column chromatography on silica gel to afford the desired product.

Note: Racemic samples for the standard of chiral HPLC spectra were prepared using racemic NHC precursor as catalyst.

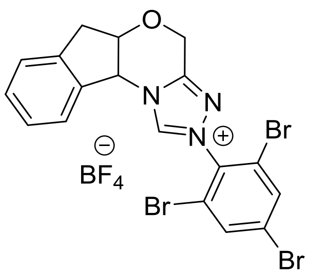

Characterization data of 3, 4, 5, 6, 7, 8, 10.

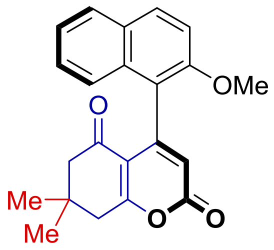

3aa: 70%; white solid; **^{1}H NMR** (400MHz, $CDCl_3$) δ 7.90 (d, J = 9.0 Hz, 1H), 7.82 (d, J = 8.0 Hz, 1H), 7.47 (d, J = 8.3 Hz, 1H), 7.40 – 7.26 (m, 3H), 6.14 (s, 1H), 3.80 (s, 3H), 2.80 (s, 2H), 2.32 (d, J = 15.9 Hz, 1H), 2.22 (d, J = 15.9 Hz, 1H), 1.14 (s, 3H), 1.11 (s, 3H); **^{13}C NMR** (101MHz, $CDCl_3$) δ 192.5, 172.5, 160.1, 152.4, 152.0, 131.2, 130.0, 128.7, 128.2, 126.8, 123.5, 123.4, 120.0, 116.0, 114.5, 112.3, 56.1, 51.6, 42.5, 31.7, 28.0, 27.8; **HRMS** (ESI): *m/z*: calculated for $C_{22}H_{20}NaO_4^+$: $[M + Na]^+$ 371.1254, found: 371.1250; HPLC (Chiralpak IB, *i*-propanol/hexane = 10/90, flow rate 1.0 mL/min, λ = 254 nm): t_R (major) = 25.8 min, t_R (minor) = 32.7 min, e.r. = 90:10; $[\alpha]^{25}_D$ = -214.6 (c = 1.0, $CHCl_3$).

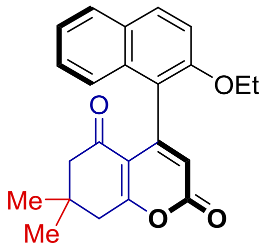

3ab: 60%; yellow oil; **^{1}H NMR** (400MHz, $CDCl_3$) δ 7.87 (d, J = 9.0 Hz, 1H), 7.80 (d, J = 7.6 Hz, 1H), 7.45 (d, J = 8.4 Hz, 1H), 7.39 – 7.32 (m, 2H), 7.27 – 7.24 (m, 1H), 6.14 (s, 1H), 4.14 – 4.08 (m, 2H), 2.84 (s, 2H), 2.30 (dd, J = 28.0, 15.9 Hz, 2H), 1.27

(t, J = 7.0 Hz, 3H), 1.17 (s, 3H), 1.13 (s, 3H); ^{13}**C NMR** (101MHz, $CDCl_3$) δ 192.6, 172.6, 160.3, 152.5, 152.0, 131.5, 130.0, 128.8, 128.3, 126.9, 123.6, 123.6, 120.4, 116.1, 114.6, 113.6, 64.6, 51.8, 42.7, 31.9, 28.4, 28.0, 15.1; **HRMS** (ESI): *m/z*: calculated for $C_{23}H_{23}O_4^+$: $[M + H]^+$ 363.1591, found: 363.1597; HPLC (Chiralpak IB, *i*-propanol/hexane = 10/90, flow rate 1.0 mL/min, λ = 254 nm): t_R (major) = 19.9 min, t_R (minor) = 21.4 min, e.r. = 75:25; $[\alpha]^{25}_D$ = -103.2 (c = 1.0, $CHCl_3$).

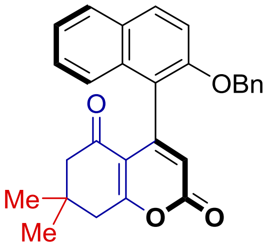

3ac: 60%; yellow oil; ^{1}**H NMR** (400MHz, $CDCl_3$) δ 7.87 (dd, J = 22.8, 9.2 Hz, 2H), 7.52 (d, J = 8.3 Hz, 1H), 7.44 – 7.28 (m, 8H), 6.15 (s, 1H), 5.19 (d, J = 12.0 Hz, 1H), 5.10 (d, J = 12.0 Hz, 1H), 2.78 (dd, J = 23.8, 18.4 Hz, 2H), 2.18 (dd, J = 26.4, 15.8 Hz, 2H), 1.11 (s, 3H), 1.03 (s, 3H); ^{13}**C NMR** (101MHz, $CDCl_3$) δ 192.7, 172.4, 160.2, 152.2, 151.7, 136.9, 131.4, 130.1, 129.1, 128.5, 128.3, 127.9, 127.1, 126.9, 123.8, 123.7, 121.0, 116.0, 114.6, 114.1, 71.2, 51.6, 42.5, 31.7, 28.0, 28.0; **HRMS** (ESI): *m/z*: calculated for $C_{28}H_{25}O_4^+$: $[M + H]^+$ 425.1747, found: 425.1741; HPLC (Chiralpak IB, *i*-propanol/hexane = 10/90, flow rate 1.0 mL/min, λ = 254 nm): t_R (major) = 20.7 min, t_R (minor) = 28.4 min, e.r. = 72.5:8.5; $[\alpha]^{25}_D$ = -83.2 (c = 1.0, $CHCl_3$).

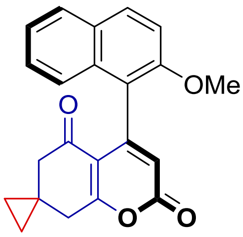

3ba: 61%; yellow oil; ^{1}**H NMR** (400 MHz, $CDCl_3$) δ 7.90 (d, J = 9.0 Hz, 1H), 7.82 (d, J = 7.9 Hz, 1H), 7.50 (d J = 8.4 Hz, 1H), 7.40 (dt, J = 8.4, 1.4 Hz, 1H), 7.34 (dt, J = 6.8, 1.2 Hz, 1H), 7.29 (d, J = 9.1 Hz, 1H), 6.17 (s, 1H), 3.84 (s, 3H) 2.94 (d, J = 18.7

Hz, 1H), 2.76 (d, J = 18.7 Hz, 1H), 2.41 (d, J =15.8 Hz, 1H), 2.18 (d, J = 16.1 Hz, 1H), 0.63 – 0.49 (m, 4H); **^{13}C NMR** (101MHz, $CDCl_3$) δ 192.2, 173.2, 159.9, 152.4, 152.4, 131.4, 130.2, 128.8, 128.3, 127.0, 123.7, 123.5, 120.1, 116.2, 115.6, 112.6, 56.3, 46.9, 38.4, 14.0, 11.4, 11.1; **HRMS** (ESI): *m/z*: calculated for $C_{22}H_{18}NaO_4^+$: $[M + Na]^+$ 369.1097, found: 369.1091; HPLC (Chiralpak IB, *i*-propanol/hexane = 20/80, flow rate 1.0 mL/min, λ = 254 nm): t_R (major) = 15.6 min, t_R (minor) = 22.0 min, e.r. = 85:15; $[\alpha]^{25}_D$ = -129.2 (*c* = 1.0, $CHCl_3$).

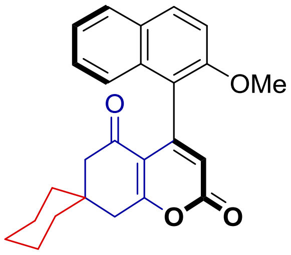

3ca: 69%; yellow oil; **^{1}H NMR** (400 MHz, $CDCl_3$) δ 7.90 (d, J = 9.0 Hz, 1H), 7.81 (d, J = 7.5 Hz, 1H), 7.48 (d, J = 8.4 Hz, 1H), 7.40 – 7.31 (m 2H), 7.26 (d, J = 1.7 Hz, 1H), 6.14 (s, 1H), 3.82 (s, 3H), 2.89 (t, J = 19.5 Hz, 2H), 2.37 (dd, J = 41.6, 15.9 Hz, 2H), 1.55 – 1.42 (m, 10H); **^{13}C NMR** (101MHz, $CDCl_3$) δ 192.7, 172.3, 160.4, 152.5, 152.0, 131.3, 130.2, 128.8, 128.3, 127.0, 123.6, 123.5, 120.0, 116.0, 114.8, 112.5, 56.2, 49.7, 40.3, 36.4, 36.3, 34.6, 31.6, 25.8, 22.6, 21.5, 21.4; **HRMS** (ESI): *m/z*: calculated for $C_{25}H_{25}O_4^+$: $[M + H]^+$ 389.1747, found: 389.1742; HPLC (Chiralpak IB, *i*-propanol/hexane = 15/85, flow rate 1.0 mL/min, λ = 254 nm): t_R (major) = 14.5 min, t_R (minor) = 16.0 min, e.r. = 88:12; $[\alpha]^{25}_D$ = -133.6 (*c* = 1.0, $CHCl_3$).

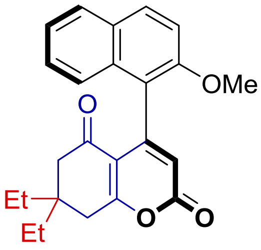

3da: 68%; yellow oil; **^{1}H NMR** (400MHz, $CDCl_3$) δ 7.92 (d, *J* = 9.0 Hz, 1H), 7.84 (d, *J* = 8.0 Hz, 1H), 7.51 (d, *J* = 8.4 Hz, 1H), 7.43 – 7.29 (m, 3H), 6.17 (s, 1H), 3.85 (s,

3H), 2.85 (dd, J = 22.9, 18.7 Hz, 2H), 2.38 (d. J = 15.8 Hz, 1H), 2.29 (d, J = 15.8 Hz, 1H), 1.60 – 1.45 (m, 4H), 0.92 – 0.84 (m, 6H); **^{13}C NMR** (101MHz, $CDCl_3$) δ 193.0, 172.6, 160.4,152.5, 152.0, 131.3, 130.2, 128.9, 128.4, 127.0, 123.7, 123.6, 120.0, 116.0, 114.9, 112.5, 56.2, 47.9, 38.9, 37.2, 29.1, 28.9, 7.7, 7.6; **HRMS** (ESI): *m/z*: calculated for $C_{24}H_{25}O_4^+$: $[M + H]^+$ 377.1747, found: 377.1753; HPLC (Chiralpak ID, *i*-propanol/hexane = 15/85, flow rate 1.0 mL/min, λ = 254 nm): t_R (major) = 15.9 min, t_R (minor) = 19.5 min, e.r. = 90:10; $[\alpha]^{25}_D$ = -163.4 (c = 1.0, $CHCl_3$).

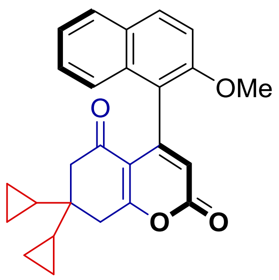

3ea: 72%; yellow oil; **^{1}H NMR** (400MHz, $CDCl_3$) δ 7.93 (d, J = 9.0 Hz, 1H), 7.84 (d, J = 8.0 Hz, 1H), 7.48 (d, J = 8.2 Hz, 1H), 7.42 – 7.29 (m, 3H), 6.16 (s, 1H), 3.85 (s, 3H), 2.76 (dd, J = 21.4, 19.0 Hz, 2H), 2.24 (d, J = 15.9 Hz, 1H), 2.15 (d, J = 15.9 Hz, 1H), 0.92 – 0.77 (m, 2H), 0.47 – 0.35 (m, 8H); **^{13}C NMR** (101MHz, $CDCl_3$) δ 192.5, 172.6, 160.2, 152.5, 152.0, 131.3, 130.1, 128.8, 128.3, 126.9, 123.6, 123.5, 120.0, 115.9, 115.0, 112.5, 56.2, 46.0, 37.7, 35.2, 18.4, 18.4, 0.7, 0.6, 0.6, 0.5; **HRMS** (ESI): *m/z*: calculated for $C_{26}H_{25}O_4^+$: $[M + H]^+$ 401.1747, found: 401.1752; HPLC (Chiralpak IB, *i*-propanol/hexane = 20/80, flow rate 1.0 mL/min, λ = 190 nm): t_R (major) = 15.9 min, t_R (minor) = 19.0 min, e.r. = 93:7; $[\alpha]^{25}_D$ = -177.2 (c = 1.0, $CHCl_3$).

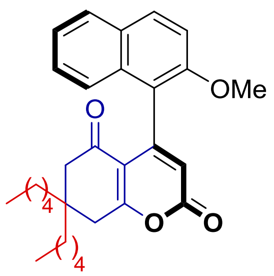

3fa: 71%; yellow oil; **^{1}H NMR** (400MHz, $CDCl_3$) δ 7.93 (d, J = 9.0 Hz, 1H), 7.84 (d,

J = 8.0 Hz, 1H), 7.49 (d, *J* = 8.3 Hz, 1H), 7.42 – 7.28 (m, 3H), 6.17 (s, 1H), 3.86 (d, *J* = 6.4 Hz, 3H), 2.86 (dd, *J* = 23.4, 22.6 Hz, 2H), 2.38 (d, *J* = 15.9 Hz, 1H), 2.29 (d, *J* = 15.9 Hz, 1H), 1.46 – 1.25 (m, 18H), 0.94 – 0.88 (m, 6H); **^{13}C NMR** (101MHz, $CDCl_3$) δ 192.9, 172.7, 160.4, 152.5, 152.0, 131.4, 130.2, 128.8, 128.4, 127.0, 123.7, 123.5, 120.0, 116.0, 114.8, 112.5, 56.3, 48.7, 39.7, 37.3, 37.2, 37.0, 32.4, 32.3, 23.0, 22.9, 22.5, 22.5, 14.1, 14.0; **HRMS** (ESI): *m/z*: calculated for $C_{30}H_{37}O_4^+$: $[M + H]^+$ 461.2686, found: 461.2680; HPLC (Chiralpak IB, *i*-propanol/hexane = 20/80, flow rate 1.0 mL/min, λ = 190 nm): t_R (major) = 11.0 min, t_R (minor) = 13.2 min, e.r. = 94:6; $[\alpha]^{25}_D$ = -106.8 (*c* = 1.0, $CHCl_3$).

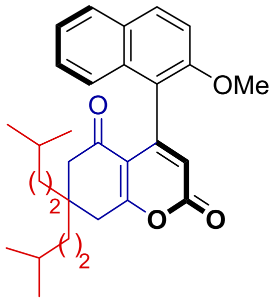

3ga: 70%; yellow oil; **^{1}H NMR** (400MHz, $CDCl_3$) δ 7.90 (d, *J* = 9.0 Hz, 1H), 7.81 (d, *J* = 7.5 Hz, 1H), 7.44 (d, *J* = 8.1 Hz, 1H), 7.38 – 7.26 (m, 3H), 6.13 (s, 1H), 3.82 (s, 3H), 2.83 (dd, *J* = 23.4, 18.6 Hz, 2H), 2.31 (dd, *J* = 33.2, 15.9 Hz, 2H), 1.50 – 1.36 (m, 6H), 1.14 – 1.06 (m, 4H), 0.91 – 0.85 (m, 12H); **^{13}C NMR** (101MHz, $CDCl_3$) δ 192.7, 172.7, 160.3, 152.5, 152.0, 131.4, 130.1, 128.8, 128.3, 126.9, 123.6, 123.5, 120.1, 116.0, 114.7, 112.5, 56.2, 48.7, 39.7, 36.8, 34.7, 34.7, 32.1, 32.1, 28.5, 28.4, 22.5; **HRMS** (ESI): *m/z*: calculated for $C_{30}H_{37}O_4^+$: $[M + H]^+$ 461.2686, found: 461.2691; HPLC (Chiralpak IB, *i*-propanol/hexane = 20/80, flow rate 1.0 mL/min, λ = 190 nm): t_R (major) = 9.9 min, t_R (minor) = 14.7 min, e.r. = 95:5; $[\alpha]^{25}_D$ = -189.8 (*c* = 1.0, $CHCl_3$).

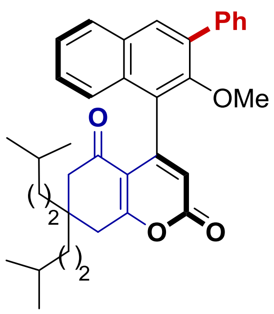

3gd: 57%; yellow oil; **^{1}H NMR** (400 MHz, $CDCl_3$) δ 7.86 – 7.84 (m, 2H), 7.68-7.66 (m, 2H), 7.47 – 7.36 (m, 6H), 6.23 (s, 1H), 3.21 (s, 3H), 2.86 (s, 2H) 3.34 (dd, J = 24.0, 16.0 Hz, 2H), 1.47 – 1.39 (m, 6H), 1.14 – 1.08 (m, 4H), 0.90 – 0.85 (m, 12H) **^{13}C NMR** (101MHz, $CDCl_3$) δ 192.8, 173.0, 160.1, 152.2, 151.3, 138.3, 134.2, 130.9, 130.5, 129.1, 128.4, 128.4, 127.4, 126.6, 125.1, 123.8, 115.7, 114.7, 60.4, 48.6, 39.9, 36.9, 34.8, 34.7, 32.1, 28.5, 28.4, 22.6, 22.5, 22.5; **HRMS** (ESI): *m/z*: calculated for $C_{36}H_{41}O_4^+$: [M + H]$^+$ 537.2999, found: 537.2993; HPLC (Chiralpak IA, *i*-propanol/hexane = 10/90, flow rate 1.0 mL/min, λ = 300 nm): t_R (major) = 5.3 min, t_R (minor) = 6.2 min, e.r. = 90:10; $[\alpha]^{25}_D$ = -173.2 (*c* = 1.0, $CHCl_3$).

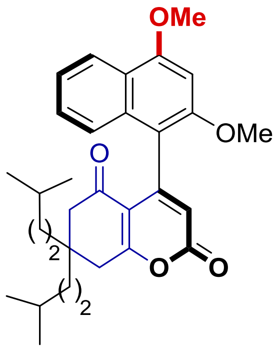

3ge: 76%; yellow oil; **^{1}H NMR** (400MHz, $CDCl_3$) δ 8.20 (d, *J* = 8.2 Hz, 1H), 7.42 – 7.26 (m, 3H), 6.64 (s, 1H), 6.14 (s, 1H), 4.04 (s, 3H), 3.82 (s, 3H), 2.82 (dd, *J* = 23.2, 18.8 Hz, 2H), 2.31 (dd, *J* = 31.8, 15.8 Hz, 2H), 1.51 – 1.35 (m, 6H), 1.13 – 1.10 (m, 4H), 0.90 – 0.85 (m, 12H); **^{13}C NMR** (101MHz, $CDCl_3$) δ 193.0, 172.5, 160.5, 157.4, 153.1, 152.2, 132.0, 127.6, 123.2, 123.0, 122.5, 121.1, 116.3, 115.0, 112.4, 92.6, 56.3, 55.6, 48.9, 39.8, 36.8, 34.7, 34.7, 32.2, 32.1, 28.5, 28.5, 22.6, 22.6; **HRMS** (ESI): *m/z*: calculated for $C_{31}H_{39}O_5^+$: [M + H]$^+$ 491.2792, found: 491.2798; HPLC (Chiralpak IB,

i-propanol/hexane = 20/80, flow rate 1.0 mL/min, λ = 190 nm): t_R (major) = 6.3 min, t_R (minor) = 8.8 min, e.r. = 93:7; $[\alpha]^{25}_D$ = -165.4 (*c* = 1.0, $CHCl_3$).

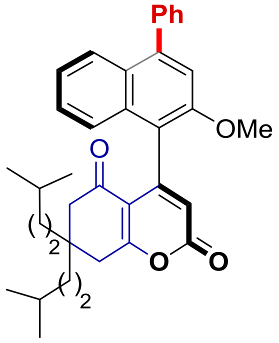

3gf: 74%; yellow oil; **^{1}H NMR** (400MHz, $CDCl_3$) δ 7.86 (d, *J* = 8.4 Hz, 1H), 7.58 – 7.48 (m, 6H), 7.41 – 7.38 (m, 1H), 7.31 – 7.25 (m, 2H), 6.22 (s, 1H), 3.86 (s, 3H), 2.89 (dd, *J* = 23.7, 18.6 Hz, 2H), 2.44 – 2,32 (m, 2H), 1.54 – 1.42 (m, 6H), 1.20 – 1.11 (m, 4H), 0.95 – 0.90 (m, 12H); **^{13}C NMR** (101MHz, $CDCl_3$) δ 193.0, 172.8, 160.4, 152.1, 151.8, 142.6, 140.5, 132.0, 130.1, 128.3, 127.6, 127.2, 126.8, 126.6, 123.9, 123.7, 119.6, 116.2, 114.9, 113.8, 56.3, 48.8, 39.8, 36.9, 34.8, 34.8, 32.2, 32.2, 28.6, 28.5, 22.6; **HRMS** (ESI): *m/z*: calculated for $C_{36}H_{41}O_4^+$: $[M + H]^+$ 537.2999, found: 537.2991; HPLC (Chiralpak IB, *i*-propanol/hexane = 20/80, flow rate 1.0 mL/min, λ = 190 nm): t_R (major) = 5.4 min, t_R (minor) = 4.9 min, e.r. = 95:5; $[\alpha]^{25}_D$ = -173.9 (*c* = 1.0, $CHCl_3$).

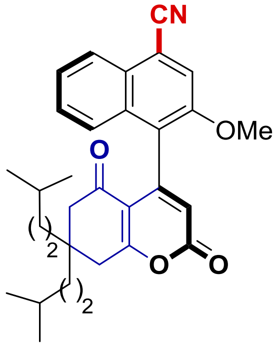

3gg: 60%; yellow oil; **^{1}H NMR** (400MHz, $CDCl_3$) δ 8.24 (d, *J* = 8.2 Hz, 1H), 7.69 (s, 1H), 7.60 – 7.50 (m, 3H), 6.10 (s, 1H), 3.88 (s, 3H), 2.87 (dd, *J* = 21.8, 18.9 Hz, 2H), 2.39 – 2.27 (m, 2H), 1.52 – 1.38 (m, 6H), 1.18 – 1.04 (m, 4H), 0.93 – 0.87 (m, 12H);

^{13}C NMR (101MHz, $CDCl_3$) δ 192.8, 173.5, 160.0, 151.5, 150.1, 131.3, 128.3, 128.3, 126.3, 126.3, 125.6, 124.3, 118.2, 117.5, 115.7, 114.1, 111.4, 56.5, 48.4, 39.7, 36.9, 34.8, 34.6, 32.1, 32.1, 28.5, 28.4, 22.5, 22.4; **HRMS** (ESI): *m/z*: calculated for $C_{31}H_{36}NO_4^+$: $[M + H]^+$ 486.2639, found: 486.2634; HPLC (Chiralpak IB, *i*-propanol/hexane = 20/80, flow rate 1.0 mL/min, λ = 365 nm): t_R (major) = 8.6 min, t_R (minor) = 7.0 min, e.r. = 93:7; $[\alpha]^{25}_D$ = -145.8 (*c* = 1.0, $CHCl_3$).

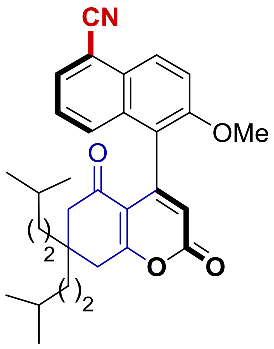

3gh: 70%; yellow oil; **^{1}H NMR** (400MHz, $CDCl_3$) δ 8.34 (d, *J* = 9.2 Hz, 1H), 7.78 (d, *J* = 7.0 Hz, 1H), 7.70 (d, *J* = 8.6 Hz, 1H), 7.50 (d, *J* = 9.2 Hz, 1H), 7.42 (dd, *J* = 8.6, 7.2 Hz, 1H), 6.10 (s, 1H), 3.89 (s, 3H), 2.86 (dd, *J* = 26.4, 18.7 Hz, 2H), 2.34 (dd, *J* = 37.2, 16.0 Hz, 2H), 1.54 – 1.37 (m, 6H), 1.17 – 1.07 (m, 4H), 0.93 – 0.88 (m, 12H); **^{13}C NMR** (101MHz, $CDCl_3$) δ 192.9, 173.3, 159.9, 153.7, 150.6, 131.2, 130.3, 128.8, 127.3, 125.8, 121.0, 117.8, 116.1, 114.8, 114.3, 110.6, 56.3, 48.6, 39.7, 36.8, 34.8, 34.6, 32.1, 32.1, 28.5, 28.4, 22.5, 22.4; **HRMS** (ESI): *m/z*: calculated for $C_{31}H_{36}NO_4^+$: $[M + H]^+$ 486.2639, found: 486.2644; HPLC (Chiralpak IB, *i*-propanol/hexane = 20/80, flow rate 1.0 mL/min, λ = 190 nm): t_R (major) = 19.1 min, t_R (minor) = 17.5 min, e.r. = 95.5:4.5; $[\alpha]^{25}_D$ = -198.6 (*c* = 1.0, $CHCl_3$).

Additional experiment:

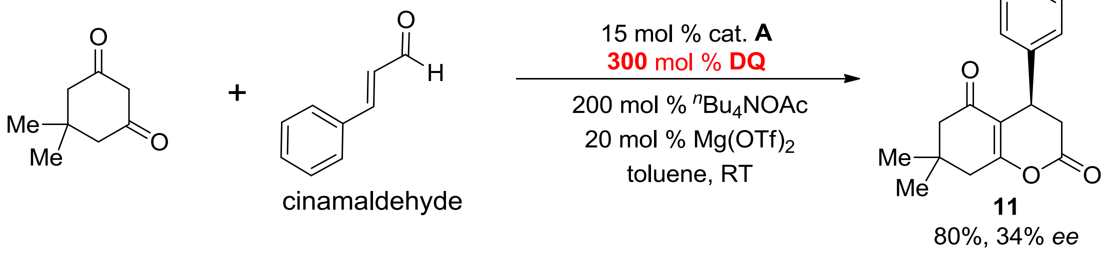

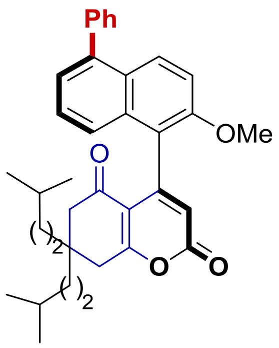

3gi: 71%; yellow oil; **^{1}H NMR** (400MHz, $CDCl_3$) δ 7.97 (d, *J* = 9.4 Hz, 1H), 7.53 – 7.41 (m, 7H), 7.30 (d, *J* = 1.16 Hz, 1H), 7.23 (d, *J* = 9.4 Hz 1H), 6.20 (s, 1H), 3.83 (s, 3H), 2.89 (dd, *J* = 22.5, 18.6 Hz, 2H), 2.37 (dd, *J* = 30.4, 15.9 Hz, 2H), 1.54 – 1.41 (m, 6H), 1.19 – 1.13 (m, 4H), 0.95 – 0.90 (m, 12H); **^{13}C NMR** (101MHz, $CDCl_3$) δ 192.9, 172.8, 160.3, 152.4, 152.3, 140.8, 140.7, 131.8, 130.1, 128.4, 128.2, 127.2, 127.0, 126.5, 124.8, 123.1, 120.2, 116.0, 114.8, 112.4, 56.2, 48.7, 39.8, 36.8, 34.7, 34.7, 32.1, 28.5, 28.4, 22.5; **HRMS** (ESI): *m/z*: calculated for $C_{36}H_{41}O_4^+$: $[M + H]^+$ 537.2999, found: 537.2993; HPLC (Chiralpak IB, *i*-propanol/hexane = 20/80, flow rate 1.0 mL/min, λ = 300 nm): t_R (major) = 6.4 min, t_R (minor) = 5.7 min, e.r. = 93:7; $[\alpha]^{25}_D$ = -167.4 (*c* = 1.0, $CHCl_3$).

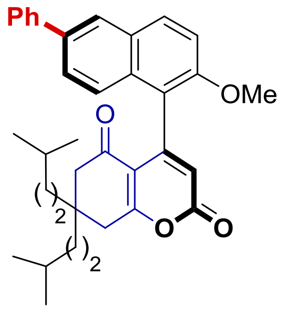

3gj: 68%; yellow oil; **^{1}H NMR** (400MHz, $CDCl_3$) δ 8.01 (s, 1H), 7.95 (d, *J* = 9.0 Hz, 1H), 7.67 – 7.53 (m, 3H), 7.53 – 7.44 (m, 3H), 7.37 – 7.29 (m, 2H), 6.16 (s, 1H), 3.83 (s, 3H), 2.84 (dd, *J* = 23.1, 18.9 Hz, 2H), 2.32 (dd, *J* = 33.5, 15.8 Hz, 2H), 1.50 – 1.37 (m, 6H), 1.11 (d, *J* = 6.4 Hz, 4H), 0.90 – 0.85 (m, 12H); **^{13}C NMR** (101MHz, $CDCl_3$) δ 192.8, 172.8, 160.3, 152.6, 151.9, 140.9, 136.4, 130.5, 130.4, 129.0, 128.8, 127.2, 127.1, 126.7, 126.2, 124.0, 120.0, 115.9, 114.7, 112.9, 56.2, 48.7, 39.7, 36.8, 34.7,

34.6, 32.1, 32.1, 28.5, 28.4, 22.5; **HRMS** (ESI): *m/z*: calculated for $C_{34}H_{37}O_4^+$: [M + H]$^+$ 537.2999, found: 537.2992; HPLC (Chiralpak IB, *i*-propanol/hexane = 20/80, flow rate 1.0 mL/min, λ = 190 nm): t_R (major) = 7.1 min, t_R (minor) = 8.9 min, e.r. = 96:4; $[\alpha]^{25}_D$ = -178.6 (*c* = 1.0, $CHCl_3$).

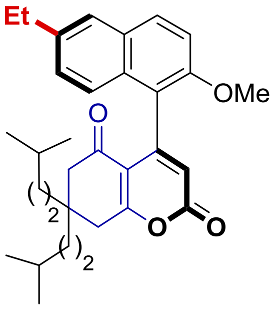

3gk: 66%; yellow oil; ^{1}**H NMR** (400MHz, $CDCl_3$) δ 7.83 (d, *J* = 9.0 Hz, 1H), 7.59 (s, 1H), 7.37 (d, *J* = 8.6 Hz, 1H), 7.24 (d, *J* = 9.0 Hz, 2H), 6.13 (s, 1H), 3.80 (s, 3H), 2.88 – 2.73 (m, 4H), 2.31 (dd, *J* = 34.3, 15.8 Hz, 2H), 1.50 – 1.36 (m, 6H), 1.31 – 1.27 (m, 3H), 1.27 -1.11 (m, 4H), 0.90 – 0.85 (m, 12H); ^{13}**C NMR** (101MHz, $CDCl_3$) δ 192.7, 172.6, 160.4, 152.2, 152.0, 139.4, 129.8, 129.5, 129.1, 128.2, 125.9, 123.4, 120.0, 115.9, 114.8, 112.6, 56.3, 48.7, 39.7, 36.8, 34.7, 34.7, 32.1, 32.1, 28.6, 28.5, 28.4, 22.5, 22.5, 15.4; **HRMS** (ESI): *m/z*: calculated for $C_{32}H_{41}O_4^+$: [M + H]$^+$ 489.2999, found: 489.2992; HPLC (Chiralpak IB, *i*-propanol/hexane = 20/80, flow rate 1.0 mL/min, λ = 190 nm): t_R (major) = 6.1 min, t_R (minor) = 8.1 min, e.r. = 95:5; $[\alpha]^{25}_D$ = -165.9 (*c* = 1.0, $CHCl_3$).

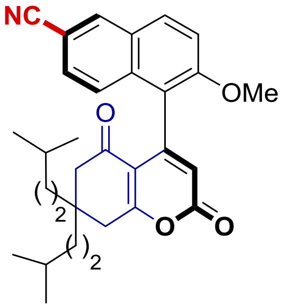

3gl: 72%; white solid; ^{1}**H NMR** (400MHz, $CDCl_3$) δ 8.20 (s, 1H), 7.96 (d, *J* = 9.1 Hz, 1H), 7.49 (dd, *J* = 13.4, 8.8 Hz, 2H), 7.39 (d, *J* = 9.1 Hz, 1H), 6.08 (s, 1H), 3.87 (s,

3H), 2.85 (dd, J = 22.6, 18.9 Hz, 2H), 2.31 (dd, J = 32.4, 16.0 Hz, 2H), 1.51 – 1.36 (m, 6H), 1.15 – 1.08 (m, 4H), 0.91 – 0.85 (m, 12H); **^{13}C NMR** (101MHz, $CDCl_3$) δ 193.0, 173.4, 159.9, 155.0, 150.6, 134.7, 132.9, 130.9, 127.4, 127.3, 124.8, 120.6, 119.3, 116.1, 114.3, 114.0, 107.0, 56.2, 48.6, 39.8, 36.9, 34.8, 34.6, 32.1, 32.1, 28.5, 28.4, 22.6, 22.5; **HRMS** (ESI): *m/z*: calculated for $C_{31}H_{36}NO_4^+$: $[M + H]^+$ 486.2639, found: 486.2634; HPLC (Chiralpak IA, *i*-propanol/hexane = 20/80, flow rate 1.0 mL/min, λ = 190 nm): t_R (major) = 8.1 min, t_R (minor) = 11.7 min, e.r. = 96.5:3.5; $[\alpha]^{25}_D$ = -187.0 (c = 1.0, $CHCl_3$).

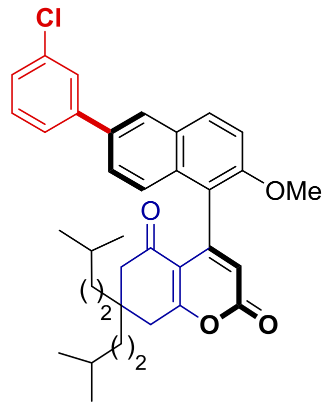

3gm: 70%; yellow oil; **^{1}H NMR** (400MHz, $CDCl_3$) δ 7.99 – 7.95 (m, 2H), 7.65 (s, 1H), 7.94 (d, J = 9.0 Hz, 1H), 7.59 – 7.51 (m, 3H), 7.40 – 7.31 (m, 3H), 6.16 (s, 1H), 3.84 (s, 3H), 2.85 (dd, J = 22.8, 18.8 Hz, 2H), 2.32 (dd, J = 33.8, 15.8 Hz, 2H), 1.50 – 1.37 (m, 6H), 1.12 – 1.11 (m, 4H), 0.91 – 0.86 (m, 12H); **^{13}C NMR** (101MHz, $CDCl_3$) δ 192.9, 172.9, 160.3, 152.9, 151.8, 142.8, 135.0, 134.7, 130.9, 130.5, 130.1, 128.9, 127.3, 127.2, 126.5, 126.3, 125.4, 124.3, 120.1, 116.0, 114.7, 113.1, 56.3, 48.7, 39.8, 36.9, 34.8, 34.7, 32.2, 32.1, 28.5, 28.5, 22.6; **HRMS** (ESI): *m/z*: calculated for $C_{36}H_{39}ClNaO_4^+$: $[M + Na]^+$ 593.2429, found: 593.2424; HPLC (Chiralpak IB, *i*-propanol/hexane = 20/80, flow rate 1.0 mL/min, λ = 190 nm): t_R (major) = 7.6 min, t_R (minor) = 9.0 min, e.r. = 95:5; $[\alpha]^{25}_D$ = -173.6 (c = 1.0, $CHCl_3$).

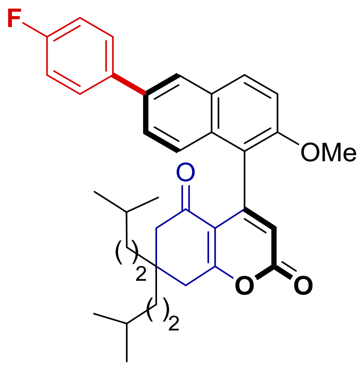

3gn: 71%; yellow oil; **^{1}H NMR** (400MHz, $CDCl_3$) δ 7.94 (d, *J* = 9.0 Hz, 2H), 7.63 – 7.56 (m, 3H), 7.51 (d, *J* = 8.8 Hz, 1H), 7.31 (d, *J* = 9.0 Hz, 1H), 7.14 (t, *J* = 8.6 Hz, 2H), 6.16 (s, 1H), 3.83 (s, 3H), 2.85 (dd, *J* = 23.7, 18.8 Hz, 2H), 2.32 (dd, *J* = 34.6, 15.8 Hz, 2H), 1.50 – 1.37 (m, 6H), 1.14 – 1.07 (m, 4H), 0.91 – 0.85 (m, 12H); **^{13}C NMR** (101MHz, $CDCl_3$) δ 192.9, 172.8, 162.4 (d, *J* = 244.7 Hz), 160.3, 152.7, 151.8, 137.0 (d, *J* = 3.1 Hz), 135.4, 130.5, 130.3, 128.9, 128.7 (d, *J* = 7.9 Hz), 126.5, 126.0, 134.2, 120.0, 116.0, 115.7 (d, *J* = 21.3 Hz), 114.7, 113.1, 76.7, 56.2, 48.7, 39.7, 36.8, 34.7, 34.7, 32.1, 32.1, 28.5, 28.4, 22.5; **^{19}F NMR** (376 MHz, $CDCl_3$) δ -115.8; **HRMS** (ESI): *m/z*: calculated for $C_{36}H_{39}FNaO_4^+$: $[M + Na]^+$ 577.2725, found: 577.2730; HPLC (Chiralpak IB, *i*-propanol/hexane = 20/80, flow rate 1.0 mL/min, λ = 190 nm): t_R (major) = 7.5 min, t_R (minor) = 10.8 min, e.r. = 95:5; $[\alpha]^{25}_D$ = -165.4 (*c* = 1.0, $CHCl_3$).

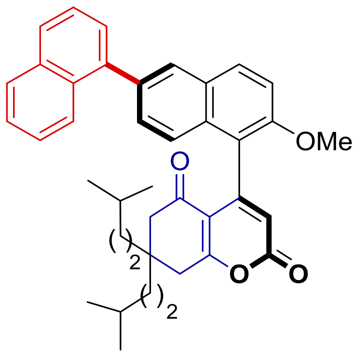

3go: 67%; yellow oil; **^{1}H NMR** (400MHz, $CDCl_3$) δ 7.96 – 7.86 (m, 5H), 7.56 – 7.48 (m, 5H), 7.43 (d, *J* = 7.6 Hz, 1H), 7.33 (d, *J* = 9.0 Hz, 1H), 6.22 (s, 1H), 3.86 (s, 3H), 2.85 (dd, *J* = 25.2, 18.8 Hz, 2H), 2.36 (dd, *J* = 32,3, 15.9 Hz, 2H), 1.51 – 1.38 (m, 6H), 1.15 – 1.07 (m, 4H), 0.92 – 0.85 (m, 12H); **^{13}C NMR** (101MHz, $CDCl_3$) δ 193.0,

172.9, 160.4, 152.8, 152.0, 140.0, 136.1, 133.9, 131.8, 130.6, 130.4, 129.6, 129.2, 128.9, 128.3, 127.7, 127.2, 126.2, 126.1, 125.8, 125.5, 123.4, 120.2, 116.1, 114.8, 113.0, 56.4, 48.8, 39.8, 36.9, 34.8, 34.7, 32.2, 32.2, 28.6, 28.5, 22.6, 22.6; **HRMS** (ESI): *m/z*: calculated for $C_{40}H_{43}O_4^+$: $[M + H]^+$ 587.3156, found: 587.3151; HPLC (Chiralpak IC, *i*-propanol/hexane = 20/80, flow rate 1.0 mL/min, λ = 190 nm): t_R (major) = 9.4 min, t_R (minor) = 11.4 min, e.r. = 94:6; $[\alpha]^{25}_D$ = -179.4 (*c* = 1.0, $CHCl_3$).

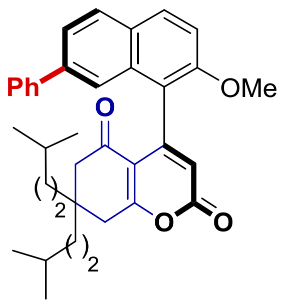

3gp: 72%; yellow oil; **^{1}H NMR** (400MHz, $CDCl_3$) δ 7.90 (dd, *J* = 14.2, 9.0 Hz, 2H), 7.57 (t, 7.1 Hz, 4H), 7.42 (t, *J* = 7.3 Hz, 2H), 7.36 – 7.26 (m, 2H), 6.19 (s, 1H), 3.83 (s, 3H), 2.83 (dd, *J* = 21.6, 19.0 Hz, 2H), 2.31 (dd, *J* = 37.5, 15.8 Hz, 2H), 1.51 – 1.33 (m, 6H), 1.13 – 1.03 (m, 4H), 0.91 – 0.77 (m, 12H); **^{13}C NMR** (101MHz, $CDCl_3$) δ 192.9, 172.9, 160.4, 153.0, 152.2, 141.4, 139.9, 131.6, 129.9, 128.9, 128.0, 127.6, 127.4, 123.7, 121.6, 120.3, 116.0, 114.7, 112.5, 56.3, 48.8, 39.8, 36.8, 34.8, 32.2, 32.1, 28.5, 28.4, 22.6, 22.5; **HRMS** (ESI): *m/z*: calculated for $C_{34}H_{37}O_4^+$: $[M + H]^+$ 537.2999, found: 537.2993; HPLC (Chiralpak IB, *i*-propanol/hexane = 20/80, flow rate 1.0 mL/min, λ = 190 nm): t_R (major) = 8.0 min, t_R (minor) = 10.2 min, e.r. = 96:4; $[\alpha]^{25}_D$ = -198.6 (*c* = 1.0, $CHCl_3$).

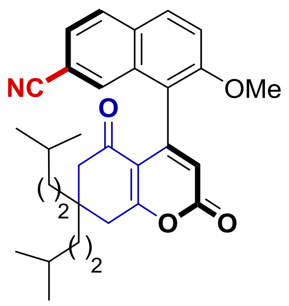

3gq: 60%; yellow oil; **^{1}H NMR** (400MHz, $CDCl_3$) δ 7.95 (d, *J* = 9.0 Hz, 1H), 7.90 (d, *J* = 8.4 Hz, 1H), 7.45 (t, *J* = 10.5 Hz, 2H), 6.08 (s, 1H), 3.85 (s, 3H), 2.87 (s, 2H), 2.32 (dd, *J* = 24.5, 15.9 Hz, 2H), 1.51 – 1.37 (m, 6H), 1.13 – 1.09 (m, 4H), 0.90 – 0.86 (m, 12H); **^{13}C NMR** (101MHz, $CDCl_3$) δ 193.1, 173.5, 159.7, 153.8, 150.3, 130.5, 130.2, 129.9, 129.7, 129.6, 124.0, 120.8, 119.2, 116.1, 115.4, 114.2, 110.5, 56.3, 48.6, 39.8, 36.9, 35.0, 34.4, 32.1, 32.1, 28.5, 28.4, 22.6, 22.5; **HRMS** (ESI): *m/z*: calculated for $C_{31}H_{36}NO_4^+$: $[M + H]^+$ 486.2639, found: 486.2644; HPLC (Chiralpak IB, *i*-propanol/hexane = 20/80, flow rate 1.0 mL/min, λ = 190 nm): t_R (major) = 9.4 min, t_R (minor) = 10.8 min, e.r. = 93:7; $[\alpha]^{25}_D$ = -143.7 (*c* = 1.0, $CHCl_3$).

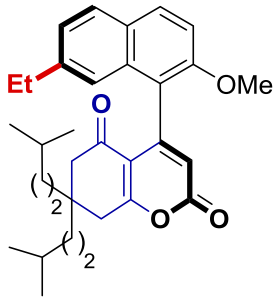

3gr: 71%; yellow oil; **^{1}H NMR** (400MHz, $CDCl_3$) δ 7.87 (d, *J* = 9.0 Hz, 1H), 7.76 (d, *J* = 8.2 Hz, 1H), 7.28 (s, H), 7.23 (d, *J* = 9.8 Hz, 1H), 6.16 (s, 1H), 3.83 (s, 3H), 2.87 (t, *J* = 19.5 Hz, 2H), 2.71 (dd, *J* = 14.8, 7.3 Hz, 2H), 2.34 (dd, *J* = 36.5, 15.8 Hz, 2H), 1.52 – 1.38 (m, 6H), 1.25 (t, *J* = 7.7 Hz, 3H), 1.14 – 1.13 (m, 4H), 0.93 – 0.86 (m, 12H); **^{13}C NMR** (101MHz, $CDCl_3$) δ 192.8, 172.6, 160.5, 152.6, 152.3, 143.0, 131.6, 129.8, 128.3, 127.4, 124.8, 121.3, 119.6, 115.9, 114.8, 111.6, 56.2, 48.8, 39.7, 36.8, 34.7, 34.6, 32.1, 32.1, 29.4, 28.5, 28.4, 22.5, 15.6; **HRMS** (ESI): *m/z*: calculated for $C_{32}H_{41}O_4^+$: $[M + H]^+$ 489.2999, found: 489.2993; HPLC (Chiralpak IB, *i*-propanol/hexane = 20/80, flow rate 1.0 mL/min, λ = 254 nm): t_R (major) = 6.8 min, t_R (minor) = 8.8 min, e.r. = 97:3; $[\alpha]^{25}_D$ = -189.6 (*c* = 1.0, $CHCl_3$).

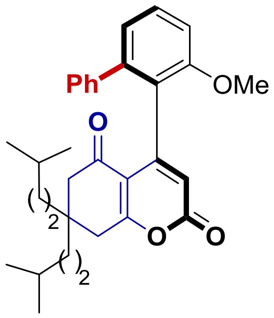

3gs: 60%; yellow oil; **^{1}H NMR** (400MHz, $CDCl_3$) δ 7.39 (t, *J* = 7.9 Hz, 1H), 7.26 – 7.20 (m, 3H), 7.12 – 7.10 (m, 2H), 6.99 (d, *J* = 37.8 Hz, 1H), 6.88 (d, *J* = 8.3 Hz, 1H), 5.70 (s, 1H), 3.72 (s, 3H), 2.76 (d, *J* = 18.5 Hz, 1H), 2.64 (d, *J* = 18.5 Hz, 1H), 2.39 (d, *J* = 15.5 Hz, 1H), 2.25 (d, *J* = 15.5 Hz, 1H), 1.49 – 1.26 (m, 6H), 1.15 – 1.05 (m, 4H), 0.89 (d, *J* = 6.6 Hz, 12H); **^{13}C NMR** (101MHz, $CDCl_3$) δ 193.5, 171.4, 160.2, 155.6, 152.8, 140.7, 140.6, 129.1, 128.0, 127.1, 125.6, 122.5, 115.6, 115.2, 109.1, 55.7, 48.7, 39.5, 37.0, 35.0, 34.1, 32.1, 32.0, 28.5, 28.5, 22.6, 22.6, 22.5, 22.5; **HRMS** (ESI): *m/z*: calculated for $C_{32}H_{39}O_4^+$: $[M + H]^+$ 487.2843, found: 487.2844; HPLC (Chiralpak IB, *i*-propanol/hexane = 20/80, flow rate 1.0 mL/min, λ = 254 nm): t_R (major) = 7.5 min, t_R (minor) = 7.1 min, e.r. = 96:4; $[\alpha]^{25}_D$ = -176.8 (*c* = 1.0, $CHCl_3$).

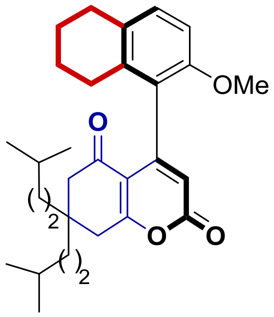

3gt: 61%; yellow oil; **^{1}H NMR** (400MHz, $CDCl_3$) δ 7.05 (d, *J* = 8.4Hz, 1H), 6.69 (d, *J* = 8.2 Hz, 1H), 5.97 (s, 1H), 3.64 (s, 3H), 2.84 – 2.72 (m, 4H), 2.54 (d, *J* = 16.9 Hz, 1H), 2.33 (dd, *J* = 24.2, 15.9 Hz, 2H), 2.18 (d, *J* = 16.4 Hz, 1H), 1.71 – 1.63 (m, 4H), 1.48 – 1.36 (m, 6H), 1.11 – 1.10 (m, 4H), 0.89 – 0.86 (m, 12H); **^{13}C NMR** (101MHz, $CDCl_3$) δ 192.9, 172.6, 160.5, 153.4, 153.2, 133.8, 129.6, 129.4, 125.8, 114.7, 114.2, 108.1, 55.6, 48.7, 39.7, 36.8, 34.8, 34.5, 32.1, 29.0, 28.5, 28.4, 27.6, 22.9, 22.5, 22.5, 22.4; **HRMS** (ESI): *m/z*: calculated for $C_{30}H_{41}O_4^+$: $[M + H]^+$ 465.2999, found:

465.2993; HPLC (Chiralpak IB, *i*-propanol/hexane = 10/90, flow rate 1.0 mL/min, λ = 190 nm): t_R (major) = 6.8 min, t_R (minor) = 7.3 min, e.r = 92:8; $[\alpha]^{25}_D$ = -143.2 (*c* = 1.0, $CHCl_3$).

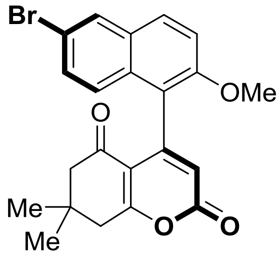

3au: 68%; white solid; **^{1}H NMR** (400MHz, $CDCl_3$) δ 7.97 (d, *J* = 1.4 Hz, 1H), 7.81 (d, *J* = 9.1 Hz, 1H), 7.44 (dd, *J* = 9.0, 1.8 Hz, 1H), 7.35 – 7.26 (m, 2H), 6.11 (s, 1H), 3.82 (s, 3H), 2.84 (s, 2H), 2.30 (dd, *J* = 36.1, 15.9 Hz, 2H), 1.17 (s, 3H), 1.13 (s, 1H); **^{13}C NMR** (101MHz, $CDCl_3$) δ 192.7, 172.8, 160.0, 152.7, 151.4, 130.2, 129.9, 129.8, 129.2, 125.3, 120.3, 117.4, 116.1, 114.4, 113.5, 56.2, 51.6, 42.6, 31.8, 28.2, 28.0; **HRMS** (ESI): *m/z*: calculated for $C_{20}H_{20}BrO_4^+$: $[M + H]^+$ 427.0539, 429.0519, found: 427.0534, 429.0513; HPLC (Chiralpak IB, *i*-propanol/hexane = 20/80, flow rate 1.0 mL/min, λ = 254 nm): t_R (major) = 10.4 min, t_R (minor) = 12.8 min, e.r. = 91:9; $[\alpha]^{25}_D$ = -193.2 (*c* = 1.0, $CHCl_3$).

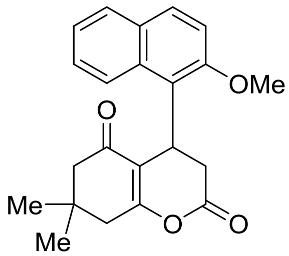

4aa: 74%; White solid; **^{1}H NMR** (400MHz, $CDCl_3$) δ 8.48 (d, *J* = 8.7 Hz, 1H), 7.75 (d, *J* = 8.6 Hz, 2H), 7.58 (t, *J* = 7.4 Hz, 1H), 7.35 (t, *J* = 7.3 Hz, 1H), 7.18 (d, *J* = 9.0 Hz, 1H), 5.12 (d, *J* = 10.1 Hz, 1H), 3.81 (s, 3H), 3.03 (dd, *J* = 16.9, 10.3 Hz, 1H), 2.69 (d, *J* = 16.9 Hz, 1H), 2.49 (dd, *J* = 28.6, 17.8 Hz, 2H), 2.17 (dd, *J* = 21.7, 16.4 Hz, 2H), 1.11 (s, 3H), 0.98 (s, 3H); **^{13}C NMR** (101MHz, $CDCl_3$) δ 197.0, 165.6, 165.0, 155.0, 132.2, 129.5, 129.2, 128.4, 126.7, 123.6, 123.4, 122.5, 112.7, 112.0,

54.4, 50.7, 41.5, 33.9, 32.3, 28.4, 27.7, 26.8; **HRMS** (ESI): *m/z*: calculated for $C_{22}H_{23}O_4^+$: [M + H]$^+$ 351.1591, found: 351.1596; HPLC (Chiralpak AD-H, *i*-propanol/hexane = 20/80, flow rate 1.0 mL/min, λ = 190 nm): t_R (major) = 7.7 min, t_R (minor) = 6.1 min, e.r. = 67:33; $[\alpha]^{25}_D$ = -53.2 (*c* = 1.0, $CHCl_3$).

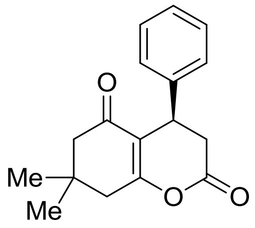

11: 80%; white solid; **^{1}H NMR** (400 MHz, $CDCl_3$) δ 7.30 – 7.20 (m, 3H), 7.15 (d, *J* = 7.2 Hz, 2H), 4.30 (t, *J* = 4.6 Hz, 1H), 2.94 (dd, *J* = 21.8, 16.2 Hz, 1H), 2.93 (s, 1H) 2.54 (s, 2H), 2.32 (s, 2H) 1.15 (s, 3H), 1.10 (s, 3H); **^{13}C NMR** (101MHz, $CDCl_3$) δ 196.1, 166.0, 165.7, 140.5, 129.1, 127.5, 126.5, 116.1, 50.6, 41.0, 36.3, 33.8, 32.5, 28.5, 28.1; **HRMS** (ESI): m/z: calculated for $C_{17}H_{19}O_3^+$: [M + H]$^+$ 271.1329, found: 271.1327; HPLC (Chiralpak AD-H, *i*-propanol/hexane = 20/80, flow rate 1.0 mL/min, λ = 254 nm): t_R (major) = 7.8 min, t_R (minor) = 5.2 min, e.r. = 67:33; $[\alpha]^{25}_D$ = -110.2 (c = 1.0, $CHCl_3$).

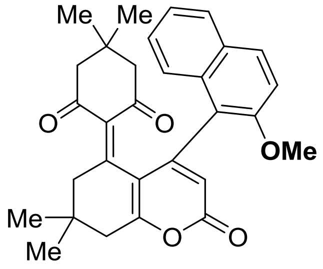

5aa: Yellow oil; **^{1}H NMR** (400MHz, $CDCl_3$) δ 7.90 (d, *J* = 9.1 Hz, 1H), 7.80 (d, *J* = 7.3 Hz, 1H), 7.41 – 7.26 (m, 4H), 5.79 (s, 1H), 3.83 (s, 3H), 2.96 (s, 2H), 2.38 (dd, *J* = 18.3, 16.3 Hz, 2H), 2.04 (d, *J* = 7.6 Hz, 2H), 1.58 (dd, *J* = 22.9, 18.0 Hz, 2H), 1.20 (d, *J* = 3.5 Hz, 6H), 0.81 (s, 6H); **^{13}C NMR** (101MHz, $CDCl_3$) δ 199.2, 191.9, 169.6, 166.9, 154.7, 154.2, 140.3, 132.5, 130.4, 128.7, 128.1, 128.1, 126.7, 123.8, 123.5, 121.5, 114.8, 113.2, 112.9, 56.4, 52.6, 50.4, 41.1, 37.8, 34.8, 32.4, 28.5, 28.3, 27.8,

27.7; **HRMS** (ESI): *m/z*: calculated for $C_{30}H_{31}O_5^+$: $[M + H]^+$ 471.2166, found: 471.2161.

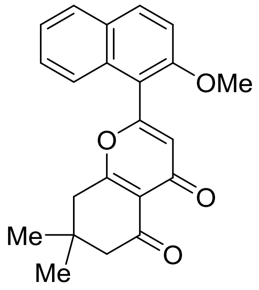

6aa: Yellow oil; **^{1}H NMR** (400MHz, $CDCl_3$) δ 7.93 (d, *J* = 9.1 Hz, 1H), 7.81 (d, *J* = 8.1 Hz, 1H), 7.72 (d, *J* = 8.5 Hz, 1H), 7.51 - 7.47 (m, 2H), 7.38 (t, *J* = 7.6 Hz, 1H), 7.30 (d, *J* = 9.1 Hz, 1H), 3.97 (s, 3H), 2.62 (s, 2H), 2.38 (s, 2H), 1.14 (s, 6H); **^{13}C NMR** (101MHz, $CDCl_3$) δ 191.7, 190.5, 179.3, 156.2, 147.2, 132.1, 131.8, 128.7, 128.4, 127.2, 124.3, 124.0, 114.8, 113.3, 112.6, 110.6, 56.2, 51.8, 39.0, 33.8, 28.4; **HRMS** (ESI): *m/z*: calculated for $C_{22}H_{21}O_4^+$: $[M + H]^+$ 349.1434, found: 349.1439.

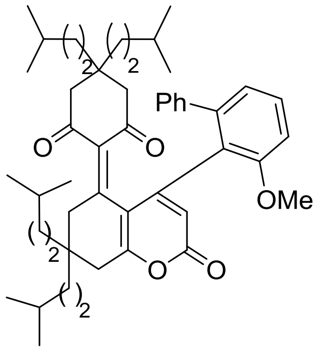

7: 20%; yellow oil; **^{1}H NMR** (400MHz, $CDCl_3$) δ 7.93 (t, *J* = 8.0 Hz, 1H), 7.19 - 7.09 (m, 5H), 7.00 (d, *J* = 8.2 Hz, 1H), 6.96 (d, *J* = 8.2 Hz, 1H), 5.93 (s, 1H), 3.76 (s, 3H), 2.80 (s, 2H), 2.39 (d, *J* = 16.1 Hz, 1H), 2.30 (d, *J* = 16.1 Hz, 1H), 2.25 (s, 2H), 2.07 (s, 2H), 2.48 – 2.10 (m, 20H), 0.96 – 0.88 (m, 24H); **^{13}C NMR** (101MHz, $CDCl_3$) δ 199.6, 192.3, 169.0, 167.1, 156.7, 154.7, 143.1, 141.0, 139.5, 129.6, 129.4, 129.1, 127.6, 127.0, 122.1, 121.9, 118.3, 115.5, 109.5, 55.9, 49.6, 47.7, 40.1, 38.8, 37.8, 35.5, 34.5, 34.4, 33.7, 32.4, 32.3, 32.2, 29.7, 28.5, 22.7, 22.6, 22.6; **HRMS** (ESI): *m/z*: calculated for $C_{48}H_{65}O_5^+$: $[M + H]^+$ 721.4827, found: 721.4821; HPLC (Chiralpak IC,

i-propanol/hexane = 10/90, flow rate 1.0 mL/min, λ = 254 nm): t_R (major) = 6.7 min, t_R (minor) = 4.6 min, e.r. = 59:41; $[\alpha]^{25}_D$ = -11.2 (*c* = 1.0, $CHCl_3$).

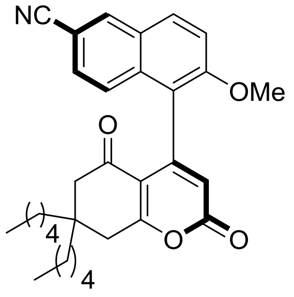

8: 71%; yellow oil; **^{1}H NMR** (400MHz, $CDCl_3$) δ 8.22 (t, *J* = 7.9 Hz, 1H), 7.98 (d, *J* = 4.4 Hz, 1H), 7.56 – 7.40 (m, 2H), 7.41 (d, *J* = 4.4 Hz, 1H), 6.10 (s, 1H), 3.88 (s, 3H), 2.87 (dd, *J* = 22.0 Hz, 18.8 Hz, 2H), 2.33 (dd, *J* = 33.2 Hz, 16.0 Hz, 2H), 1.45 – 1.24 (m, 18H), 0.90 (dd, *J* = 14.8 Hz, 6.8 Hz, 6H); **^{13}C NMR** (101MHz, $CDCl_3$) δ 193.1, 173.3, 159.9, 155.0, 134.6, 132.8, 130.9. 127.4, 127.3, 120.5, 119.2, 116.1, 114.3, 113.9, 106.9, 56.2, 48.6, 39.6, 37.4, 37.1, 32.3, 32.2, 22.9, 22.8, 22.5, 14.0, 13.9; **HRMS** (ESI): *m/z*: calculated for $C_{31}H_{38}NO_4^+$: $[M + H]^+$ 488.2801, found: 488.2808; HPLC (Chiralpak IA, *i*-propanol/hexane = 20/80, flow rate 1.0 mL/min, λ = 254 nm): t_R (major) = 9.6 min, t_R (minor) = 17.5 min, e.r. = 96:4; $[\alpha]^{25}_D$ = -17.6 (*c* = 1.0, $CHCl_3$).

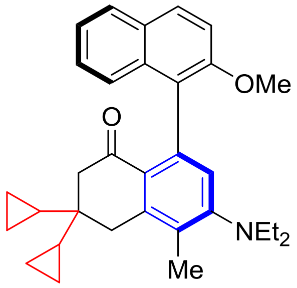

10a: 52%; White solid; **^{1}H NMR** (400 MHz, $CDCl_3$) δ 7.84 – 7.78 (m, 2H), 7.32 (d, *J* = 9.0 Hz, 1H), 7.27 – 7.21 (m, 3H), 6.75 (s, 1H), 3.75 (s, 3H), 3.03 (dd, *J* = 14.1, 7.0 Hz, 4H), 2.73 (dd, *J* = 21.0, 17.1 Hz, 2H), 2.31 (s, 3H), 2.23 (d, *J* = 15.1 Hz, 1H), 2.12 (d, *J* = 15.2 Hz, 1H), 1.02 (t, *J* = 7.1 Hz, 6H), 0.85 – 0.76 (m, 2H), 0.37 – 0.30 (m, 8H); **^{13}C NMR** (101MHz, $CDCl_3$) δ 197.4, 153.8, 152.8, 142.7, 134.5, 133.3,

130.9, 129.1, 127.9, 127.8, 127.4, 127.3, 125.7, 124.8, 123.9, 123.1, 114.0, 56.7, 46.8, 46.4, 37.0, 35.6, 18.8, 18.5, 14.6, 12.2, 1.0, 0.6, 0.3, 0.2, 0.0; **HRMS** (ESI): m/z: calculated for $C_{32}H_{38}NO_2^+$: $[M + H]^+$ 468.2897, found: 468.2897; HPLC (Chiralpak IB, *i*-propanol/hexane = 5/95, flow rate 1.0 mL/min, λ = 241 nm): t_R (major) = 9.6 min, t_R (minor) = 16.5 min, e.r. = 93:7; $[\alpha]^{25}_D$ = -140.2 (c = 1.0, $CHCl_3$).

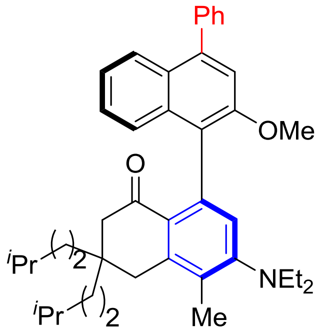

10b: 52%; yellow oil; **^{1}H NMR** (400 MHz, $CDCl_3$) δ 7.83 – 7.81 (m, 1H), 7.60 – 7.57 (m, 2H), 7.50 (t, *J* = 7.4 Hz, 2H), 7.45 – 7.41 (m, 1H), 7.29 – 7.26 (m, 2H), 7.21 – 7.19 (m, 2H), 6.80 (s, 1H), 3.76 (s, 3H), 3.04 (dd, *J* = 14.1, 7.0 Hz, 4H), 2.81 (s, 2H), 2.35 (dd, *J* = 28.4, 16.0 Hz, 2H), 2.34 (s, 3H), 1.44 – 1.36 (m, 6H), 1.20 – 1.17 (m, 2H), 1.10 – 1.06 (m, 2H), 1.02 (t, *J* = 7.0 Hz, 6H), 0.88 – 0.84 (m, 12H); **^{13}C NMR** (101MHz, $CDCl_3$) δ 197.8, 153.8, 152.1, 142.8, 141.2, 140.1, 134.7, 133.7, 131.1, 130.3, 128.2, 127.4, 127.1, 127.0, 126.1, 125.6, 125.1, 124.2, 123.2, 115.3, 56.7, 49.9, 46.5, 38.9, 37.3, 35.2, 34.5, 32.2, 28.6, 28.6, 22.7, 22.7, 22.5, 22.5, 14.6, 12.2; **HRMS** (ESI): m/z: calculated for C30H37O4$^+$: $[M + H]^+$ 604.4149, found: 604.4147; HPLC (Chiralpak IE, *i*-propanol/hexane = 10/90, flow rate 1.0 mL/min, λ = 254 nm): t_R (major) = 6.7 min, t_R (minor) = 8.0 min, e.r. = 95:5; $[\alpha]^{25}_D$ = -122.0 (c = 1.0, $CHCl_3$).

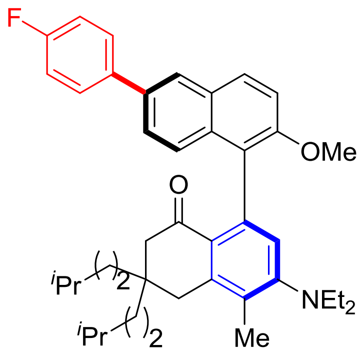

10c: 64%; yellow oil; **^{1}H NMR** (400 MHz, $CDCl_3$) δ 7.93 (d, *J* = 1.4 Hz, 1H), 7.87 (d, *J* = 9.0 Hz, 1H), 7.62 – 7.58 (m, 2H), 7.41 (dd, *J* = 8.8, 1.8 Hz, 1H), 7.35 (d, *J* = 9.0 Hz, 1H), 7.28 (d, *J* = 8.8 Hz, 1H) 7.12 (t, *J* = 8.7 Hz, 2H), 6.77 (s, 1H), 3.76 (s, 3H), 3.04 (dd, *J* = 14.2, 7.0 Hz, 4H), 2.81 (s, 2H), 2.34 (s, 3H), 2.32 (dd, *J* = 30.0, 16.0 Hz, 2H), 1.45 – 1.35 (m, 6H), 1.18 – 1.12 (m, 2H), 1.10 – 1.05 (m, 2H), 1.02 (t, *J* = 7.02 Hz, 6H), 0.87 – 0.83 (m, 12H); **^{19}F NMR** (376 MHz, $CDCl_3$) δ -116.47; **^{13}C NMR** (101MHz, $CDCl_3$) δ 197.7, 162.2 (*J* = 245.8 Hz), 153.8, 153.0, 142.8, 137.6 (*J* = 3.1 Hz), 134.8, 134.5, 132.4, 131.2, 129.2, 128.7 (*J* = 7.9 Hz), 128.1, 127.4, 127.2, 125.8, 125.4, 125.3, 124.0, 115.5 (*J* = 21.4 Hz), 114.6, 56.7, 49.9, 46.5, 38.8, 37.3, 35.1, 34.5, 32.2, 28.6, 28.6, 22.7, 22.5, 14.6, 12.2; **HRMS** (ESI): m/z: calculated for $C_{42}H_{53}FNO_2^+$: [M + H]$^+$ 622.4055, found: 622.4059; HPLC (Chiralpak IB, *i*-propanol/hexane = 5/95, flow rate 1.0 mL/min, λ = 254 nm): t_R (major) = 5.8 min, t_R (minor) = 6.9 min, e.r. = 93:7; $[\alpha]^{25}_D$ = -120.0 (c = 1.0, $CHCl_3$).

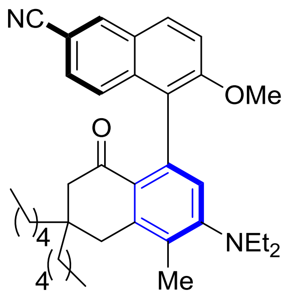

10d: 78%; yellow oil; **^{1}H NMR** (400MHz, $CDCl_3$) δ 8.22 (s, 1H), 7.91 (d, *J* = 9.2 Hz, 1H), 7.45 (d, *J* = 8.8 Hz, 4H), 7.35 – 7.28 (m, 2H), 6.71 (s, 1H), 3.83 (s, 3H), 3.07 (dd, *J* = 14.4 Hz, 7.2 Hz, 2H), 2.85 (s, 1H), 2.42 -2.29 (m, 5H), 1.30 – 1.26 (m, 18H), 1.06

(t, J = 6.8 Hz, 6H), 0.89 (dd, J = 10.8 Hz, 6.8 Hz, 6H), ; **^{13}C NMR** (101MHz, $CDCl_3$) δ 197.9, 155.4, 154.1, 143.2, 134.4, 133.3, 131.6, 128.6, 127.7, 126.1, 126.0, 123.6, 115.0, 106.2, 56.4, 49.7, 46.4,38.9, 37.7, 37.6, 32.5, 32.5, 22.5, 22.4, 14.7, 14.0, 12.2; **HRMS** (ESI): *m/z*: calculated for $C_{37}H_{49}N_2O_2^+$: [M + H]$^+$ 553.3794, found: 553.3795; HPLC (Chiralpak IA, *i*-propanol/hexane = 10/90, flow rate 1.0 mL/min, λ = 254 nm): t_R (major) = 4.5 min, t_R (minor) = 7.6 min, e.r. = 96:4; $[\alpha]^{25}_D$ = -16.8 (c = 1.0, $CHCl_3$).

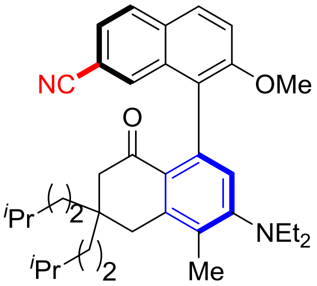

10e: 80%; yellow oil; **^{1}H NMR** (400 MHz, $CDCl_3$) δ 7.87 (d, J = 3.6 Hz, 1H), 7.85 (d, J = 3.0 Hz, 1H), 7.61 (s, 1H), 7.45 (d, J = 9.0 Hz, 1H), 7.38 (dd, J = 8.5, 1.6 Hz, 1H), 6.66 (s, 1H), 3.77 (s, 3H), 3.05 (dd, J = 14.1, 7.1 Hz, 4H), 2.82 (s, 2H), 2.35 (s, 3H), 2.32 (dd, J = 24.7, 15.8 Hz, 2H), 1.47 – 1.35 (m, 6H), 1.16 – 1.08 (m, 4H), 1.03 (t, J = 7.0 Hz, 6H), 0.86 – 0.83 (m, 12H); **^{13}C NMR** (101MHz, $CDCl_3$) δ 197.9, 154.2, 154.0, 143.3, 133.0, 132.5, 131.8, 131.4, 130.0, 129.1, 128.3, 127.9, 126.8, 123.5, 123.4, 119.7, 116.6, 109.1, 56.5, 49.8, 46.3, 39.0, 37.3, 34.8, 34.8, 32.2, 28.6, 28.5, 22.7, 22.6, 22.5, 14.7, 12.2; **HRMS** (ESI): m/z: calculated for $C_{37}H_{49}N_2O_2^+$: [M + H]$^+$ 553.3789, found: 553.3790; HPLC (Chiralpak AD-H, *i*-propanol/hexane = 10/90, flow rate 1.0 mL/min, λ = 254 nm): t_R (major) = 3.4 min, t_R (minor) = 5.1 min, e.r. = 90.5:9.5; $[\alpha]^{25}_D$ = -60.0 (c = 1.0, $CHCl_3$).

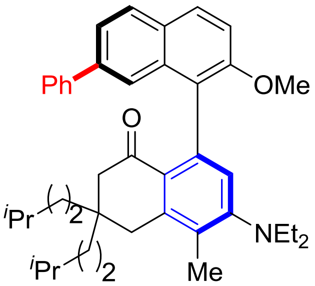

10f: 48%; yellow oil; **^{1}H NMR** (400 MHz, $CDCl_3$) δ 7.86 (t, *J* = 8.0 Hz, 2H), 7.52 (dd, *J* = 8.5 Hz, 1.8 Hz, 1H), 7.46 – 7.44 (m, 3H), 7.34 – 7.30 (m, 3H), 7.27 – 7.23 (m, 2H), 6.82 (s, 1H), 3.75 (s, 3H), 3.07 – 3.02 (m, 4H), 2.79 (s, 2H), 2.34 (s, 3H), 2.31 (dd, *J* = 39.9 Hz, 15.8 Hz, 2H), 1.44 – 1.24 (m, 6H), 1.17 – 1.07 (m, 2H), 1.01 (t, *J* = 7.0 Hz, 6H), 0.84 (dd, *J* = 11.0, 6.6 Hz, 6H), 0.73 (d, *J* = 6.6 Hz, 6H); **^{13}C NMR** (101MHz, $CDCl_3$) δ 197.7, 153.7, 153.2, 142.8, 141.9, 138.6, 134.5, 133.4, 131.3, 128.4, 128.4, 127.7, 127.5, 127.5, 127.4, 126.8, 124.1, 123.1, 123.0, 114.0, 56.6, 50.0, 46.6, 38.8, 37.3, 35.0, 34.6, 32.2, 28.6, 28.4, 22.7, 22.6, 22.5, 14.6, 12.2; **HRMS** (ESI): m/z: calculated for $C_{42}H_{54}NO_2^+$: $[M + H]^+$ 604.4149, found: 604.4147; HPLC (Chiralpak IC, *i*-propanol/hexane = 10/90, flow rate 1.0 mL/min, λ = 254 nm): t_R (major) = 5.5 min, t_R (minor) = 7.4 min, e.r. = 95:5; $[\alpha]^{25}_D$ = -72.7 (c = 1.0, $CHCl_3$).

Supplementary References:

1. Shriner, R. L.; Todd; H. R. *Organic Syntheses*, **1935**, *XV*, 14-16.
2. Jin X, Xu W, Yang J, et al. Short and tandem syntheses of spiro [2.5] octane-5, 7-dione and spiro [3.5] nonane-6, 8-dione via diethyl acetonedicarboxylate. *Tetrahedron Lett*. **56**, 6287-6289 (2015).
